# Design, synthetic approach, *in silico* molecular docking and antibacterial activity of quinazolin-2,4-dione hybrids bearing bioactive scaffolds†

**DOI:** 10.1039/d2ra06527d

**Published:** 2022-12-21

**Authors:** Aboubakr H. Abdelmonsef, Mohamed Omar, Huda R. M. Rashdan, Mohamed M. Taha, Ahmed M. Abobakr

**Affiliations:** Chemistry Department, Faculty of Science, South Valley University Qena 83523 Egypt aboubakr.ahmed@sci.svu.edu.eg; Chemistry of Natural and Microbial Products Department, Pharmaceutical and Drug Industries Research Institute, National Research Centre Dokki Cairo 12622 Egypt

## Abstract

Antimicrobial resistance (AMR) is one of ten global public health threats facing humanity. This created the need to identify and develop effective inhibitors as antimicrobial agents. In this respect, quinazolin-2,4-dione hybrids bearing N-heterocyclic cores such as pyrrolidine-2,5-dione, pyrazole and oxadiazole and/or bioactive scaffolds such as hydrazone, amide, sulfonamide, azomethine, and thiourea linkage are described for design, synthesis, antibacterial investigation, and *in silico* studies. The characterization of the target compounds was accomplished by elemental analysis and various spectroscopic data like FT-IR, ^1^H-NMR, ^13^C-NMR and MS. The antibacterial evaluation was achieved for the newly synthesized compounds using two G −ve bacteria (*Escherichia coli* ATCC 25955 and *Pseudomonas aeruginosa* ATCC 10145), and two G +ve bacteria (*Bacillus subtilis* ATCC 6633 and *Staphylococcus aureus* NRRL B-767). Synthesized compounds exhibited various activities against the tested pathogens, the results revealed that compound 3c exhibited a characteristic antimicrobial efficacy against all the tested pathogenic strains at a concentration lower than the tested standard drug ranging from 2.5 to 10 μg ml^−1^. Moreover, the molecular docking study against the target *S. aureus* tyrosyl-tRNA synthetase (PDB ID: 1JIJ) was carried out to investigate the mechanism of action of the prepared compounds, which is in line with an *in vitro* study. Most new compounds exhibited zero violation of Lipinski's rule (Ro5). These candidate molecules have shown promising antibacterial activity. Among these molecules, compound 3c with di-hydroxyl groups on two phenyl rings at position-4 exhibited a promising potent antibacterial inhibitory effect. Further SAR analysis reveals that a greater number of hydroxyl groups in an organic compound might be crucial for antibacterial efficacy. These findings demonstrate the potential activity of compound 3c as an antibacterial agent.

## Abstract 2

Antimicrobial resistance (AMR) is one of ten global public health threats facing humanity.

## Graphical abstract

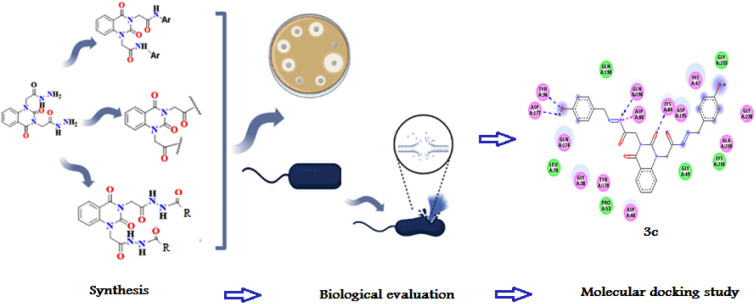

## 1. Introduction

Despite the profound success achieved to increase the number of antibiotics used to treat bacterial infections, the bacterial resistance to antibiotics is still an internationally-recognized problem.^1^ There is an urgent need to identify new bioactive molecules with high efficacy against bacterial diseases.

**Fig. 1:**
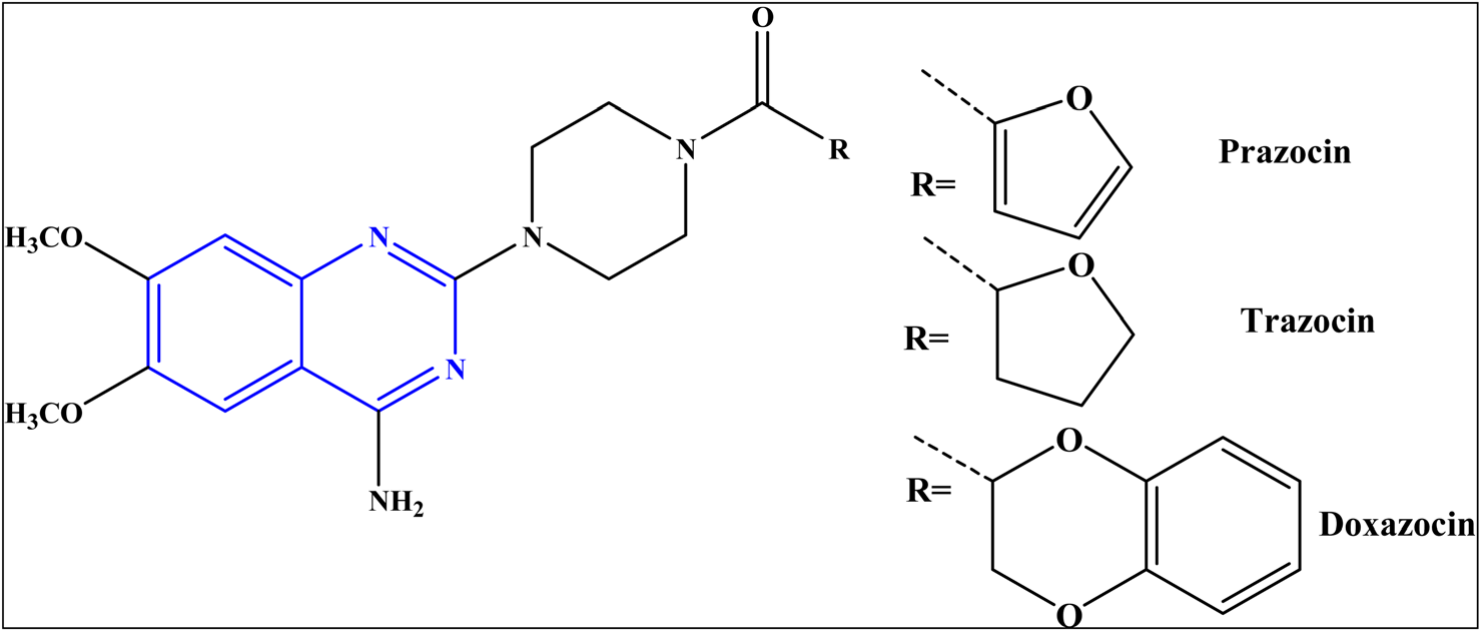
Approved marketed drugs containing quinazoline skeleton.

Quinazolines represent an important class of nitrogen-heterocycles that participate in various pharmacological and biological activities, such as anti-cancer,^2,3^ anti-malarial,^4^ anti-inflammation,^5^ anti-microbial,^6^ anti-cholera^7^ and anti-covid19 ^8^. In addition, the quinazoline derivatives are known to have drawn significant attention of in light of their biopharmaceutical significance.^8^ Currently, there are various approved drugs containing the quinazoline skeleton^9^ such as prazosin HCl, doxazosin, and terazosin HCl, as represented in Fig. 1.

On the other hand, compounds containing hydrazone, amide, sulfonamide, azomethine, and thiourea motifs are recognized for their high biological activity.^10–12^

As a result of the aforementioned facts and in continuation to our work on the development of a new class of heterocyclic molecules,^2–5,7,13–21^ we have strategically synthesized a new series of hybrid quinazolin-2,4-dione analogues having N-heterocyclic cores and/or bioactive scaffolds for the evaluation of antibacterial efficacy by *in vitro* studies. The newly synthesized products were structurally elucidated by means of elemental and spectroscopic analyses. Additionally, all of them were examined as antibacterial agents against G +ve and G −ve strains. Finally, the molecular docking studies^22–24^ were accomplished using PyRx software^25^ to examine the binding affinities of herein reported quinazolin-2,4-dione hybrid molecules toward *S. aureus* tyrosyl-tRNA synthetase. The pharmacokinetics and toxicity of these compounds were investigated using AdmetSAR, SwissADME, and mol inspiration.

**Fig. 2:**
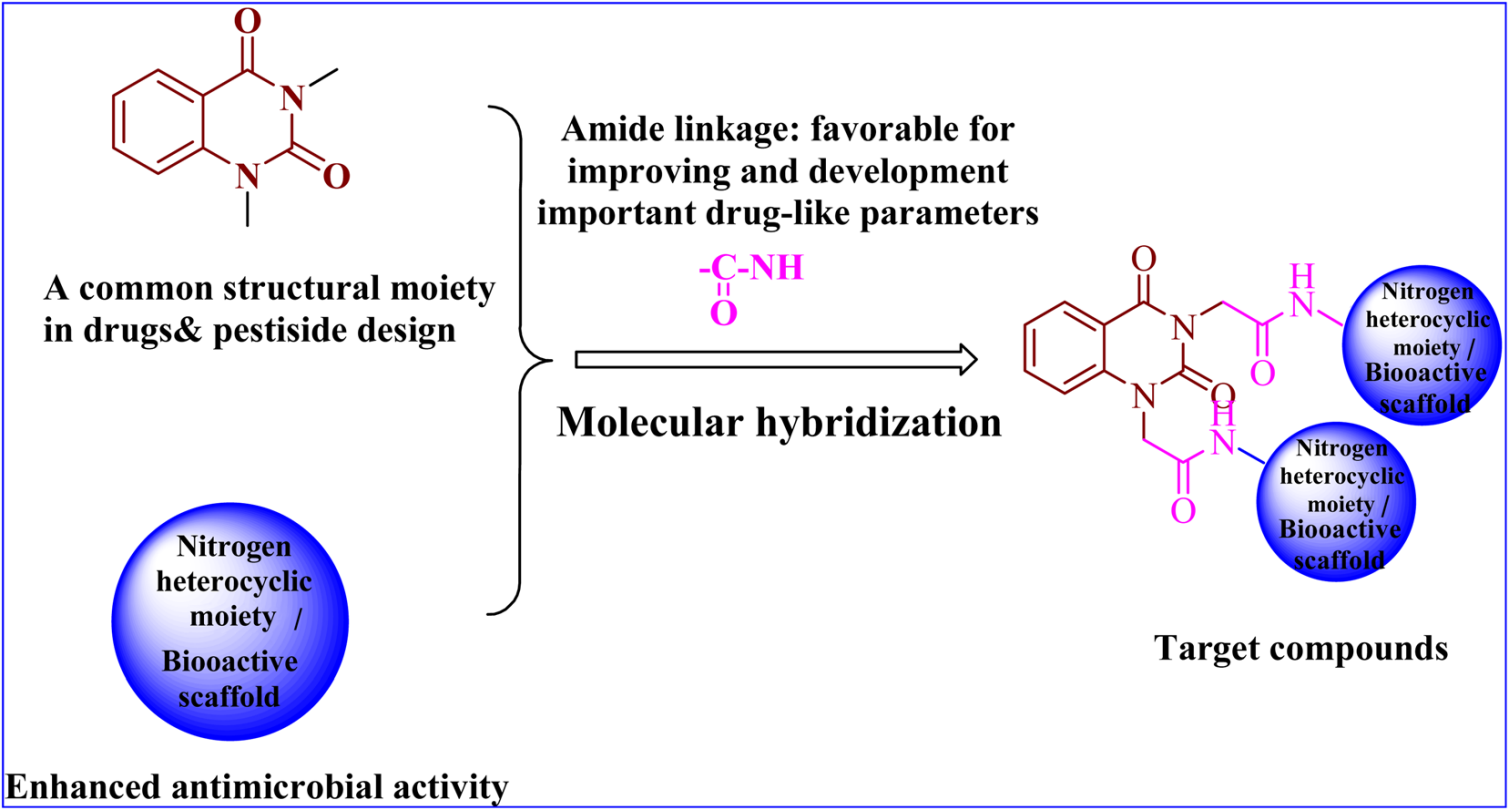
Design of the target compounds.

In conclusion, the SAR study exhibited that the quinazoline skeleton was found to be essential for antibacterial activity as shown in Fig. 2. In addition, nitrogen heterocyclic scaffolds such as pyrrolidine-2,5-dione, pyrazole and oxadiazole enhanced the antimicrobial activity. Further, bioactive scaffolds such as hydrazone, amide, sulfonamide, azomethine, and thiourea linkage were found to possess promising antibacterial efficacy. Taken together, these quinazolindione skeletons attached to N-heterocyclic moieties and/or bioactive scaffolds were found to be unique templates for development of antibacterial agents.

## 2. Experimental

### 2.1 Chemistry

All chemicals and solvents were commercially available. Melting points (°C, uncorrected) of newly compounds were checked in capillary tubes by using MEL-TEMP II. Thin Layer Chromatography (TLC) technique was used for following reactions. The techniques FT-IR, NMR and MS spectra were recorded on Shimadzu 408, Bruker Vect. 22 and Mass 5988 Mass spectrometer, respectively. Elemental analyses were carried out at Cairo University, Egypt.

#### 2,2′-(2,4-Dioxoquinazolin-1,3(2*H*,4*H*)-diyl)diacetate (1)

A mixture of ethyl 2-(2,4-dioxo-1,4-dihydroquinazolin-3(2*H*)-yl) acetate 26 (5 g, 0.02 mol) and ethyl chloroacetate (2.47 g, 0.02 mol) in a solution of dry acetone (50 ml) was refluxed for 8 h, in presence of anhydrous potassium carbonate (2.76 g, 0.02 mol). Left to cool, the reaction mixture was added to crushed ice bath; the formed solid was collected by filtration, then dried and recrystallized from benzene to obtain white crystals of compound 1. Yield 86%; mp: 120–122 °C; FT-IR (KBr, *ν*, cm^−1^) = 3125, 3088 (C–H aromatic), 2971, 2912 (C–H stretch of CH_3_ and CH_2_), 1709, 1668 (C=O); ^1^H-NMR (400 MHz, DMSO-d_6_, *δ*, ppm) = 1.27–1.32 (t, 6H, 2CH_3_), 4.22–4.29 (q, 4H, 2CH_2_), 6.99–8.26 (m, 4H, Ar-H); ^13^C-NMR (100 MHz, DMSO-d_6_, *δ*, ppm) = 14.43, 42.91, 45.32, 61.56, 61.74, 115.17, 123.98, 128.60, 136.33, 168.10, 168.30; MS (electron ionization [EI]): *m*/*z* (%) = 334 [M^+^]; anal. calcd for C_16_H_18_N_2_O_6_: C, 57.48%; H, 5.43%; N, 8.38%. Found: C, 57.78%; H, 5.53%; N, 8.08%.

#### 2,2′-(2,4-Dioxoquinazolin-1,3(2*H*,4*H*)-diyl)di-(acetohydrazide) (2)

To a solution of 1 (0.009 mol, 3 g) in 25 ml of absolute ethanol, hydrazine hydrate (0.04 mol, 2 g) was added. The reaction mixture was refluxed for 6 h, a white precipitate is formed during reflux, then the solid formed collected by filtration and recrystallized from methanol to furnish 2, as white crystals. Yield: 88%; mp: 286–288 °C; FT-IR (KBr, *ν*, cm^−1^) = 3292, 3193 (N–H stretch of NH_2_ and NH), 1671, 1610 (C=O); ^1^H-NMR (400 MHz, DMSO-d_6_, *δ*, ppm) = 4.25 and 4.31 (s, 4H, 2NH_2_), 4.56 and 4.73 (s, 4H, 2CH_2_), 7.24–8.10 (m, 4H, Ar-H), 9.25 and 9.32 (s, 2H, 2NH); ^13^C-NMR (100 MHz, DMSO-d_6_, *δ*, ppm) = 42.74, 45.54, 114.94, 115.12, 115.58, 123.47, 128.28, 140.36, 150.29, 161.20, 166.56; MS (EI): *m*/*z* (%) = 306.28 [M^+^]; anal. calcd for C_12_H_14_N_6_O_4_: C, 47.06%; H, 4.85%; N, 4.61%. Found: C, 47.16%; H, 4.95%; N, 4.21%.

#### General methods for preparation of compounds (3a–e)

To a solution of di acetohydrazide 2 (1 mmol) in glacial acetic acid (20 ml), aromatic aldehydes (2 mmol) namely, benzaldehyde, 4-chlorobenzaldehyde, 4-hydroxybenzaldehyde, 4-nitrobenzaldehyde, and furfural, respectively were added. The reaction mixture was refluxed for 10–12 h, then allowed to cool. The formed precipitate was filtrated off and recrystallized from the appropriate solvent.

#### 2,2′-(2,4-Dioxoquinazolin-1,3(2*H*,4*H*)-diyl)bis(*N*′-((*E*)-benzylidene)aceto hydrazide) (3a)

Yield: 75%; mp: >300 °C; FT-IR (KBr, *ν*, cm^−1^) = 3481 (N–H), 3190, 3093 (C–H aromatic), 2966, 2854 (C–H aliphatic), 1688, 1662 (C=O's), 1483 (C=N); ^1^H-NMR (400 MHz, DMSO-d_6_, *δ*, ppm) = 5.13 and 5.39 (s, 4H, 2CH_2_), 7.34–8.26 (m, 16H, Ar-H + 2N=CH), 11.75 and 11.87 (s, 2H, 2NH); ^13^C-NMR (100 MHz, DMSO-d_6_, *δ*, ppm) = 42.44, 45.26, 114.8, 115.12, 123.24, 127.36, 128.35, 128.97, 130.49, 134.62, 136, 140.67, 144.75, 150.87, 161.43, 168.34; MS (EI): *m*/*z* (%) = 482.5 [M^+^]; anal. calcd for C_26_H_22_N_6_O_4_: C, 64.72%; H, 4.60%; N, 17.42%. Found: C, 64.75%; H, 4.63%; N, 17.38%.

#### 2,2′-(2,4-Dioxoquinazolin-1,3(2*H*,4*H*)-diyl)bis(*N*′-((*E*)-4-chlorobenzylidene) acetohydrazide) (3b)

Yield: 81%; mp: >300 °C; FT-IR (KBr, *ν*, cm^−1^) = 3476 (N–H), 3185, 3091 (C–H aromatic), 2961, 2853 (C–H aliphatic), 1682, 1613 (C=O's), 1483 (C=N), 844 (C–Cl stretching); ^1^H-NMR (400 MHz, TFA + CDCl_3_, *δ*, ppm) = 4.92 and 5.44 (s, 4H, 2CH_2_), 7.03–8.19 (m, 14H, Ar-H + 2N=CH), 9.8 (s, 2H, 2NH); ^13^C-NMR (100 MHz, DMSO-d_6_, *δ*, ppm) = 42.11, 45.21, 114.8, 115.12, 123.24, 127.36, 128.35, 128.97, 130.49, 134.62, 136, 140.67, 144.75, 150.87, 161.43, 168.34; MS (EI): *m*/*z* (%) = 550.09 [M^+^] and 552 ([M^+^] + 2) due to the presence of two chlorine atoms; anal. calcd for C_26_H_20_Cl_2_N_6_O_4_: C, 56.64%; H, 3.66%; Cl, 12.86; N, 15.24%. Found: C, 56.63%; H, 3.67%; Cl, 12.89; N, 15.20%.

#### 2,2′-(2,4-Dioxoquinazoline-1,3(2*H*,4*H*)-diyl)bis(*N*′-((*E*)-4-hydroxybenzylidene) acetohydrazide) (3c)

Yield: 66%; mp: >300 °C; FT-IR (KBr, *ν*, cm^−1^) = 3402, 3339 (N–H), 3185, 3091 (O–H, broad), 3211, 3069 (C–H aromatic), 2967, 2883 (C–H aliphatic), 1657, 1607 (C=O's), 1421 (C=N), 1963 (O–H bending); ^1^H-NMR (400 MHz DMSO-d_6_, *δ*, ppm) = 5.09 and 5.43 (s, 4H, 2CH_2_), 6.83 and 6.85 (s, 2H, N=CH), 7.33–8.12 (m, 12H, Ar-H), 9.95 (s, 2H, 2OH), 11.54 and 11.64 (s, 2H, 2NH); ^13^C-NMR (100 MHz, DMSO-d_6_, *δ*, ppm) = 42.12, 45.33, 114.8, 115.12, 123.24, 127.36, 128.35, 128.97, 130.49, 134.62, 136, 140.67, 144.75, 150.87, 161.43, 168.34; MS (EI): *m*/*z* (%) = 514.5 [M^+^]; anal. calcd for C_26_H_22_N_6_O_6_: C, 60.70%; H, 4.31%; N, 16.33%. Found: C, 60.74%; H, 4.34%; N, 16.30%.

#### 2,2′-(2,4-Dioxoquinazoline-1,3(2*H*,4*H*)-diyl)bis(*N*′-((*E*)-4-nitrobenzylidene)aceto hydrazide) (3d)

Yield: 66%; mp: >300 °C; FT-IR (KBr, *ν*, cm^−1^) = 3472 (N–H), 3187, 3067 (C–H aromatic), 2958, 2851 (C–H aliphatic), 1683, 1613 (C=O), 1483 (C=N); ^1^H-NMR (400 MHz, DMSO-d_6_, *δ*, ppm) = 5.17 and 5.42 (s, 4H, 2CH_2_), 7.35 and 7.98 (s, 2H, 2N=CH), 8.00–8.28 (m, 12H, Ar-H), 11.90 (s, 2H, 2NH); MS (EI): *m*/*z* (%) = 572.5 [M^+^]; anal. calcd for C_26_H_20_N_8_O_8_: C, 54.55%; H, 3.52%; N, 19.57%. Found: C, 54.58%; H, 3.56%; N, 19.53%.

#### 2,2′-(2,4-Dioxoquinazoline-1,3(2*H*,4*H*)-diyl)bis(*N*′-((*E*)-furan-2-ylmethylene) acetohydrazide) (3e)

Yield: 76%; mp: >300 °C; FT-IR (KBr, *ν*, cm^−1^) = 3456, 3221 (N–H) 3140, 3083 (C–H aromatic), 2975, 2922 (C–H aliphatic), 1710, 1610 (C=O), 1565, 1483 (C=N); ^1^H-NMR (400 MHz, DMSO-d_6_, *δ*, ppm) = 5.13 and 5.37 (s, 4H, 2CH_2_), 7.35–8.12 (m, 12H, Ar-H + 2N=CH), 11.69 and 11.79 (s, 2H, 2NH); MS (EI): *m*/*z* (%) = 462 [M^+^]; anal. calcd for C_22_H_18_N_6_O_6_: C, 57.14%; H, 3.92%; N, 18.17%. Found: C, 57.15%; H, 3.96%; N, 18.13%.

#### 2,2′-(2,4-Dioxoquinazolin-1,3(2*H*,4*H*)-diyl)bis(*N*′-cyclopentylidene acetohydrazide) (4a)

A solution of 2 (0.001 mol, 0.4 g) in 20 ml ethanol and few drops of TEA, was heated at 100 °C for 1/2 h. Cyclopentanone (0.002 mol, 0.2 g) was added to the hot solution in one portion then the resulting reaction mixture was refluxed for 12 h. The reaction mixture was allowed to cool and filtered to afford a pale-yellow precipitate. Yield: 71%; mp: 240–242 °C; FT-IR (KBr, *ν*, cm^−1^) = 3506, 3431 (NH stretching of NH groups), 3049 (C–H aromatic), 2971 (C–H aromatic), 1694 (C=O); ^1^H-NMR (400 MHz, DMSO-d_6_, *δ*, ppm) = 1.71–1.92 (m, 16H, 8CH_2_), 4.94 and 5.17 (s, 4H, 2CH_2_), 7.12–8.10 (m, 4H, Ar-H), 10.31 and 10.43 (s, 2H, 2NH); ^13^C-NMR (100 MHz, DMSO-d_6_, *δ*, ppm) = 24.39, 28.67, 34.12, 43.20, 45.33, 114.24, 114.96, 115.29, 123.38, 127.77, 135.89, 140.82, 150.94, 161.40, 163.07, 164.38, 167.76; MS (EI): *m*/*z*(%) = 438 [M^+^]; anal. calcd for C_22_H_26_N_6_O_4_: C, 60.26%; H, 5.98%; N, 19.17%. Found: C, 60.31%; H, 5.99%; N, 19.14%.

#### 2,2′-(2,4-Dioxoquinazolin-1,3(2*H*,4*H*)-diyl)bis(*N*′-cyclohexylidene acetohydrazide) (4b)

To a solution of 2 (0.001 mol, 0.4 g) in (30 ml) ethanol containing few drops of TEA, cyclohexanone (0.002, 0.26 ml) was added. The resulting reaction mixture was refluxed for 8 h. After cooling, the white precipitate was filtered off and recrystallized from ethanol/acetic acid to 4b. Yield: 64%; mp: 280–282 °C; FT-IR (KBr, *ν*, cm^−1^) = 3500 (N–H stretching of NH groups), 2956 (C–H aliphatic), 1649 (C=O's); ^1^H-NMR (400 MHz, DMSO-d_6_, *δ*, ppm) = 1.60–1.66 (m, 10H, 5CH_2_), 2.25–2.42 (m, 10H, 5CH_2_), 4.93, 5.20 (s, 4H, 2CH_2_), 7.28–7.36 (m, 4H, Ar-H), 10.62, 10.77 (s, 2H, 2NH); MS (EI): *m*/*z* (%) = 466 [M^+^]; anal. calcd for C_24_H_30_N_6_O_4_: C, 61.79%; H, 6.48%; N, 18.01%. Found: C, 61.82%; H, 6.51%; N, 17.98%.

#### 2,2′-(2,4-Dioxoquinazolin-1,3(2*H*,4*H*)-diyl)bis(*N*′-((*E*)-1-(phenyl)ethylidene) acetohydrazide) (4c)

A mixture of 2 (0.001 mol, 0.4 g) and acetophenone (0.002 mol, 0.24 g) were dissolved in absolute ethanol (15 ml) then drops of triethylamine were added, the reaction mixture was refluxed for 6 h. The formed solid was filtered off, dried and further purified by crystallization from ethanol to afford compound 4c, as white crystals. Yield: 78%; mp: 292–294 °C; FT-IR (KBr, *ν*, cm^−1^): 3226 (NH stretching of NH groups), 2986 (C–H aliphatic), 1661 (C=O's); ^1^H-NMR (400 MHz, DMSO-d_6_, *δ*, ppm) = 2.80 and 2.82 (s, 6H, 2CH_3_), 4.75 and 4.82 (s, 4H, 2CH_2_), 7.31–8.08 (m, 14H, Ar-H), 10.43 and 10.57 (s, 2H, 2NH); MS (EI): *m*/*z*(%) = 510 [M^+^]; anal. calcd for C_28_H_26_N_6_O_4_: C, 65.87%; H, 5.13%; N, 16.46%. Found: C, 65.90%; H, 5.17%; N, 16.42%.

#### 2,2′-(2,4-Dioxoquinazolin-1,3(2*H*,4*H*)-diyl)bis(*N*′-((*E*)-1-(*p*-tolyl)ethylidene) acetohydrazide) (4d)

Compound 2 (0.001 mol, 0.4 g) and 4-methyl acetophenone (0.002 mol, 0.36 g) were heated in ethanol (30 ml) and drops of TEA for 8 h under reflux. Then the solid products formed was filtered off and recrystallized from ethanol to afford compound 4d, as white crystals. Yield: 77%; mp 278–280 °C; FT-IR (KBr, *ν*, cm^−1^) = 3455, 3221 (N–H stretching), 3060, 3035 (C–H aromatic), 2963, 2920 (C–H aliphatic), 1706, 1671 (C=O's), 1542, 1483 (C=N); ^1^H-NMR (400 MHz, DMSO-d_6_, *δ*, ppm) = 2.29 (s, 6H, 2CH_3_), 2.34 (s, 6H, 2CH_3_), 4.96 and 5.38 (s, 4H, 2CH_2_), 7.24–8.11 (m, 12H, Ar-H), 10.94 and 10.31 (s, 2H, 2NH); ^13^C-NMR (100 MHz, DMSO-d_6_, *δ*, ppm) = 14.01, 42.84, 45.06, 114.38, 114.98, 123.47, 126.51, 129.43, 135.41, 139.31, 140.83, 149.15, 150.9, 161.83, 169; anal. calcd for C_30_H_30_N_6_O_4_: C, 66.9%; H, 5.61%; N, 15.6%. Found: C, 67.02%; H, 5.64%; N, 15.3%.

#### *N*′,*N*′′′-(2,2′-(2,4-Dioxoquinazolin-1,3(2*H*,4*H*)-diyl)bis(acetyl))bis(thiophene-2-carbohydrazide) (4e)

To an ethanolic solution of compound 2 (0.001 mol, 0.4 g), 2-acetyl thiophene (0.002 mol, 0.3 g) was added in presence of TEA (0.3 ml). The reaction mixture was heated under reflux for 6 h, then left to cool. The precipitated solid product was filtered off, dried and finally recrystallized from ethanol to afford 4e, as brown crystals. Yield: 67%; mp: 222–224 °C; FT-IR (KBr, *ν*, cm^−1^) = 3450, 3342 (N–H stretching), 3213, 3044 (C–H aromatic), 2962, 2924 (C–H aliphatic), 1660, 1610 (C=O's), 1558, 1485 (C=N); ^1^H-NMR (400 MHz, DMSO-d_6_, *δ*, ppm) = 2.27 and 2.37 (s, 6H, 2CH_3_), 4.82 and 5.30 (s, 4H, 2CH_2_), 7.09–8.12 (m, 10H, Ar-H), 11.02 and 11.11 (s, 2H, 2NH); MS (EI): *m*/*z* (%) = 522 [M^+^]; anal. calcd for C_24_H_22_N_6_O_4_S_2_: C, 55.16%; H, 4.24%; N, 16.08%, S, 12.27%. Found: C, 55.19%; H, 4.25%; N, 16.03%, S, 12.17%.

#### 2,2′-(2,4-Dioxoquinazolin-1,3(2*H*,4*H*)-diyl)bis(*N*′-((*E*)-2-oxoindolin-3-ylidene) acetohydrazide) (4f)

The compound 2 (0.001 mol, 0.4 g) was condensed under reflux for 10 h with Isatin (0.002 mol, 0.4 g) in ethanol (20 ml) and triethyl amine. After cooling the formed solid product was filtered off, dried and recrystallized from benzene to give 4f, as yellow crystals. Yield: 68%; mp: 206–208 °C; FT-IR (KBr, *ν*, cm^−1^) = 3221 (N–H stretching), 3069 (C–H aromatic), 2965, 2815 (C–H aliphatic), 1701, 1665 (C=O's), 1484, 1466 (C=N); ^1^H-NMR (400 MHz, DMSO-d_6_, *δ*, ppm) = 5.52 and 5.68 (s, 4H, 2CH_2_), 6.96–8.13 (m, 12H, Ar-H), 11.31 and 12.73 (s, 4H, 4NH); MS (EI): *m*/*z* (%) = 564 [M^+^]; anal. calcd for C_28_H_20_N_8_O_6_: C, 59.57%; H, 3.57%; N, 19.85%, Found: C, 59.61%; H, 3.59%; N, 19.81%.

#### 2,2′-(2,4-Dioxoquinazolin-1,3(2*H*,4*H*)-diyl)bis(*N*-(1,3-dioxoisoindolin-2-yl) acetamide) (5a)

A solution of phthalic anhydride (0.002 mol, 0.6 g) in glacial acetic acid (5 ml) was added to a solution of 2 (0.001 mol, 0.4 g) in glacial acetic acid (5 ml). The mixture was refluxed for 4 h till observation a white precipitate is formed during the reaction. Then the product was filtrated off and recrystallized from ethanol to yield the corresponding compound 5a, as white crystals. Yield: 80%; mp: >300 °C; FT-IR (KBr, *ν*, cm^−1^) = 3523 (N–H stretching), 3101 (C–H aromatic), 1746, 1674 (C=O^'^s); ^1^H-NMR (400 MHz, DMSO-d_6_, *δ*, ppm) = 4.89 and 5.15 (s, 4H, 2CH_2_), 7.33–8.13, (m, 12H, Ar-H), 11.11 and 11.23 (s, 4H, 4NH); ^13^C-NMR (100 MHz, DMSO-d_6_, *δ*, ppm) = 42.68, 44.73, 115.1, 124.23, 128.51, 129.92, 135.76, 140.06, 150.8, 160.98, 164.94, 166; MS (EI): *m*/*z* (%) = 566.49 [M]^+^; anal. calcd for C_28_H_18_N_6_O_8_: C, 59.37%; H, 3.20%; N, 14.84%. Found: C, 59.34%; H, 3.16%; N, 14.80%.

#### 2,2′-(2,4-Dioxoquinazolin-1,3(2*H*,4*H*)-diyl)bis(*n*-(1,3-dioxo-1,3,3*a*,4,7,7*a*-hexahydro-2*h*-isoindol-2-yl)acetamide) (5b)

Compound 2 (0.001 mol, 0.4 g) was dissolved in (10 ml) glacial acetic acid then 1,2,3,6-tetrahydrophthalic anhydride (0.002 mol, 0.3 g) was added, the mixture was heated under reflux for 4 h. The solid formed was filtered off, dried and recrystallized from methanol to give 5b as white crystals. Yield: 81%; mp: >300 °C; FT-IR (KBr, *ν*, cm^−1^): 3507, 3292 (N–H), 3095, 3010 (C–H aromatic), 2958, 2852 (C–H aliphatic), 1727, 1631 (stretching anhydride C=O^'^s); ^1^H-NMR (400 MHz, DMSO-d_6_, *δ*, ppm) = 2.21 and 2.25 (m, 8H, 4CH_2_), 3.27 (m, 4H, 4CH (sp^3^)), 4.76, 5.01 (s, 4H, 2CH_2_), 5.85 (m, 4H, 4CH (sp^2^)), 7.24–8.10 (m, 4H, Ar-H), 10.93, 11.03 (s, 2H, 2NH); MS (EI): *m*/*z* (%) = 574.55 [M^+^]; anal. calcd for C_28_H_26_N_6_O_8_: C, 58.53%; H, 4.56%; N, 14.63%; Found: C, 58.57%; H, 4.58%; N, 14.59%.

#### 2,2′-(2,4-Dioxoquinazolin-1,3(2*H*,4*H*)-diyl)bis(*N*-(12,14-dioxo-4*a*,9,9*a*,10-tetrahydro-9,10-[3,4]epipyrroloanthracen-13-yl)acetamide) (5c)

Refluxing a mixture of compound 2 (0.001 mol, 0.4 g) and anthracene maleic anhydride (0.002 mol, 0.55 g) in glacial acetic acid (15 ml) for 3 h. After cooling; the resulting solid was separated and recrystallized from ethanol/acetic acid to yield compound (5c) as pale-yellow crystals. Yield: 77%; mp: >300 °C; FT-IR (KBr, *ν*, cm^−1^): 3511 (N–H), 3022, 2963 (sp^3^ C–H), 1792, 1662 (stretching C=O^'^s); ^1^H-NMR (400 MHz, DMSO-d_6_, *δ*, ppm) = 3.27 (d, 4H, 4CH), 4.83 (d, 4H, 4CH), 7.14–7.48 (m, 20H, Ar-H), 10.81 (s, 2H, 2NH); MS (EI): *m*/*z* (%) = 822.28 [M^+^]; anal. calcd for C_48_H_38_N_6_O_8_: C, 69.72%; H, 4.63%; N, 10.16%. Found: C, 69.76%; H, 4.67%; N, 10.13%.

#### 2,2′-(2,4-Dioxoquinazolin-1,3(2*H*,4*H*)-diyl)bis(*N*′-(acetyl)acetohydrazide) (6a)

To a stirred solution of compound 2 (0.002 mol, 0.04 g) in DMF (5 ml) at 0–5 °C acetylchloride (0.004 mol) was added. The reaction mixture was stirred for 2 h; then poured onto ice water. The solid product was collected by filtration to give compound 6a that was further purified by crystallization from ethanol. Yield: 77%; mp: >300 °C; FT-IR (KBr, *ν*, cm^−1^) = 3456, 3223 (N–H stretching), 3019 (C–H aromatic) 2928, 2852 (C–H aliphatic), 1705, 1658 (C=O's); ^1^H-NMR (400 MHz, DMSO-d_6_, *δ*, ppm) = 1.85 (s, 6H, 2CH_3_), 4.64 and 4.88 (s, 4H, 2CH_2_) 7.15–8.09, (m, 4H, Ar-H), 10.21, 10.25, 10.31 and 10.35 (s, 4H, 4NH); anal. calcd for C_16_H_18_N_6_O_6_: C, 49.23%; H, 4.65%; N, 21.53%. Found: C, 49.25%; H, 4.66%; N, 21.51%.

#### 2,2′-(2,4-Dioxoquinazolin-1,3(2*H*,4*H*)-diyl)bis(*N*′-(2-chloroacetyl)aceto hydrazide) (6b)

To a stirred solution of 2 (0.001 mol, 0.4 g) in DMF (10 ml) at 0 °C, chloroacetyl chloride (0.002 mol, 0.3 g) was added dropwise. The completion of the reaction was observed after 2 h of stirring (monitored by TLC). Then the mixture was neutralized with 10% sodium bicarbonate. The precipitate was filtered off, washed with ice-cold water, dried and recrystallized from ethanol to yield 6b, as white crystals. Yield: 75%. mp: >300 °C; FT-IR (KBr, *ν*, cm^−1^) = 3190 (N–H stretching) 3140, 3053 (C–H aromatic), 2962 (C–H aliphatic), 1717, 1675 (C=O's), 856 (C–Cl stretching);^1^H-NMR (400 MHz, DMSO-d_6_, *δ*, ppm) = 4.14 and 4.16 (s, 4H, 2CH_2_), 4.67 and 4.91 (s, 4H, 2CH_2_), 7.15–8.11, (m, 4H, Ar-H), 10.49 (s, 4H, 4NH); ^13^C-NMR (100 MHz, DMSO-d_6_, *δ*, ppm) = 41.25, 42.35, 44.49, 114.79, 123.32, 123.69, 128.57, 135.66, 140.1, 140.45, 150.83, 160.89, 164.72, 165.98; MS (EI): *m*/*z* (%) = 458.08 [M^+^] and 460.08 [M^+^ + 2], due to the presence of two chlorine atoms; anal. calcd for C_16_H_16_N_6_O_6_: C, 41.85%; H, 3.51, Cl, 15, 44%; N, 18.30%. Found: C, 41.88%; H, 3.56, Cl, 15, 44%; N, 18.25%.

#### *N*′,*N*′′′-(2,2′-(2,4-Dioxoquinazolin-1,3(2*H*,4*H*)-diyl)bis(acetyl))di(benzo hydrazide) (6c)

To a cold stirred solution of di acetohydrazide 2 (0.001 mol, 0.4 g) in DMF (10 ml), benzoyl chloride (0.002 mol, 0.2 g) was added gradually, the reaction mixture was stirred at room temperature for 4 h, then the mixture was poured gradually with stirring into a cold sodium bicarbonate solution. The separated product was filtered off, washed with water, dried, and finally recrystallized from ethanol to give 6c as white crystals. Yield: 63%; mp: 224–226 °C; FT-IR (KBr, *ν*, cm^−1^) = 3470 (N–H stretching), 3207 (C–H aromatic), 3003 (C–H aliphatic), 1702, 1658 (C=O's); ^1^H-NMR (400 MHz, DMSO-d_6_, *δ*, ppm) = 4.14 and 4.16 (s, 4H, 2CH_2_), 4.67 and 4.91 (s, 4H, 2CH_2_), 7.15–8.11 (m, 4H, Ar-H), 10.49 (s, 4H, 4NH); MS (EI): *m*/*z* (%) = 514 [M^+^]; anal. calcd for C_26_H_22_N_6_O_6_: C, 60.70%; H, 4.31; N, 16.33%. Found: C, 60.70%; H, 4.34; N, 16.29%.

#### *N*′,*N*′′′-(2,2′-(2,4-Dioxoquinazolin-1,3(2*H*,4*H*)-diyl)bis(acetyl))bis(thiophene-2-carbohydrazide) (6d)

Thiophene-2-carbonyl chloride (0.0025 mol, 0.6 g) was added to a cold solution of 2 (0.001 mol, 0.4 g) in 10 ml of DMF, the addition was carried out portion wise with stirring at 0–5 °C over a period of 5 min. After complete addition, the reaction mixture was stirred for further 4 hours, then kept in an ice-chest for 12 h, and finally diluted with water. The precipitated solid was collected, washed with water, dried and recrystallized from ethanol/DMF to afford the corresponding 6d, as white crystals. Yield: 68%; mp: 254–256 °C; FT-IR (KBr, *ν*, cm^−1^) = 3440, 3232 (N–H stretching), 3091, 3030 (C–H aromatic), 2961, 2926 (C–H aliphatic), 1710, 1674 (C=O's); ^1^H-NMR (400 MHz, DMSO-d_6_, *δ*, ppm) = 4.65 and 4.90 (s, 4H, 2CH_2_), 7.32–8.10 (m, 14H, Ar-H), 10.10, 10.13, 10.34 and 10.43 (s, 4H, 4NH); MS (EI): *m*/*z* (%) = 526 [M^+^]; anal. calcd for C_22_H_18_N_8_O_8_: C, 53.44%; H, 3.67; N, 17.00%. Found: C, 53.40%; H, 3.69; N, 16.96%.

#### *N*′,*N*′′′-(2,2′-(2,4-Dioxoquinazolin-1,3(2*H*,4*H*)-diyl)bis(acetyl))dibenzene sulphonohydrazide (6e)

To a cold solution of 2 (0.001 mol, 0.4 g) in DMF (10 ml), benzenesulphonyl chloride (0.002 mmol) was added dropwise. The reaction mixture was stirred at room temperature for 4 hours, then the mixture was poured on ice cold water, and the solid formed was collected by filtration and recrystallized from ethanol/benzene to give compounds 6e, as white powder. Yield 74%; mp: 240–242 °C; FT-IR (KBr, *ν*, cm^−1^) = 3434, 3224 (N–H stretching), 3017 (C–H aromatic), 2926, 2852 (C–H aliphatic), 1705, 1658 (C=O's), 1070 (S=O stretching); ^1^H-NMR (400 MHz, DMSO-d_6_, *δ*, ppm) = 4.75 and 4.76 (s, 4H, 2CH_2_), 7.32–8.09 (m, 14H, Ar-H), 10.43, 10.44, 10.56 and 10.57 (s, 4H, 4NH); MS (EI): *m*/*z*(%) = 586 [M^+^]; anal. calcd for C_24_H_22_N_6_O_8_S_2_: C, 49.14%; H, 3.78; N, 14.33; S, 10.93%. Found: C, 49.17%; H, 3.83; N, 14.24; S, 10.91%.

#### 1,3-Bis(2-(3,5-dimethyl-1*H*-pyrazol-1-yl)-2-oxoethyl)quinazolin-2,4(1*H*,3*H*)-dione (7a)

Heating of compound 2 (0.001 mol, 0.4 g) with acetyl acetone (0.003 mol, 0.39 g), in ethanol (30 ml) and few drops of glacial acetic acid. After cooling; the obtained solid was filtered off then recrystallized from benzene to obtain the scaffold of compound 7a, as yellow crystals. Yield: 68%; mp: 198–200 °C; FT-IR (KBr, *ν*, cm^−1^) = 3090, 3067 (C–H aromatic), 2926, 2853 (C–H aliphatic), 1712, 1669 (C=O's), 1484, 1439 (C=N stretching); ^1^H-NMR (400 MHz, DMSO-d_6_, *δ*, ppm) = 1.75 and 1.756 (s, 6H, 2CH_3_), 2.03 and 2.06 (s, 6H, 2CH_3_), 4.92 (s, 2H, CH_2_), 5.16 (s, 2H, CH_2_), 6.5 (s, 2H, sp^2^(CH)), 7.21–8.10 (m, 4H, Ar-H); ^13^C-NMR (100 MHz, DMSO-d_6_, *δ*, ppm) = 16.38, 25.81, 44.27, 46.08, 52.01, 90.8, 114.2, 114.81, 123.36, 128.04, 128.77, 135.33, 140.59, 151.38, 156.42, 161.2, 163.46; MS(EI): *m*/*z* (%) = 434.17 [M^+^]; anal. calcd for C_22_H_22_N_6_O_4_: C, 60.82%; H, 5.10; N, 19.34%. Found: C, 60.86%; H, 5.14; N, 19.29%.

#### 1,3-Bis(2-(3-methyl-5-oxo-4,5-dihydro-1*H*-pyrazol-1-yl)-2-oxoethyl)quinazolin-2,4(1*H*,3*H*)-dione (7b)

A mixture of di acetohydrazide 2 (0.001 mol, 0.4 g) and ethyl acetoacetate (0.003 mol, 0.4 g) in ethanol (30 ml) that containing few drops of glacial acetic acid as a catalyst was refluxed for 14 h to afford 7b. The produced precipitate was filtered off and recrystallized from benzene, as white crystals. Yield: 60%; mp: 138–140 °C; FT-IR (KBr, *ν*, cm^−1^) = 3126, 3088 (C–H aromatic), 2970, 2914 (C–H aliphatic), 1753, 1658 (C=O's), 1482, 1425 (C=N stretching); ^1^H-NMR (400 MHz, CDCl_3_, *δ*, ppm) = 1.27 and 1.30 (s, 6H, 2CH_3_), 4.24 (s, 4H, 2CH_2_), 4.80 (s, 4H, 2CH_2_), 6.99–8.24 (m, 4H, Ar-H); ^13^C-NMR (100 MHz, DMSO-d_6_, *δ*, ppm) = 14.14, 42.76, 45.03, 61.68, 62.1, 113.2, 115.35, 123.59, 129.41, 135.58, 139.73, 150.72, 161.21, 167.60, 167.80, 217.55; MS (EI): *m*/*z* (%) = 438.13 [M^+^]; anal. calcd for C_20_H_18_N_6_O_6_: C, 54.49%; H, 4.14; N, 19.17%. Found: C, 54.63%; H, 4.19; N, 19.13%.

#### 2,2′-(2,4-Dioxoquinazolin-1,3(2*H*,4*H*)-diyl)bis(*N*′-(2-cyanoacetyl)aceto hydrazide) (8)

Ethyl cyanoacetate (0.002 mol, 0.23 g) was added to a refluxed solution of 2 (0.001 mol, 0.4 g) in glacial acetic acid (20 ml). The reaction mixture was heated under reflux for 2 h. A white precipitate was formed during reflux; then the solid product was filtered off, washed with hot ethanol and recrystallized from dioxane to yield the target compound 8, as white crystals. Yield: 84%; mp: >300 °C; FT-IR (KBr, *ν*, cm^−1^) = 3460, 3217 (*N*–H stretching), 3062, 3002 (C–H aromatic), 2966, 2855 (C–H aliphatic), 2337 (C≡N) 1688 (C=O's); ^1^H-NMR (400 MHz, DMSO-d_6_, *δ*, ppm) = 4.12 and 4.16 (s, 4H, 2CH_2_), 5.37 and 5.64 (s, 4H, 2CH_2_), 8.00–8.85 (m, 4H, Ar-H); MS (EI): *m*/*z* (%) = 440.12 [M^+^]; anal. calcd for C_18_H_16_N_8_O_6_: C, 49.09%; H, 3.66; N, 25.45%. Found: C, 49.13%; H, 3.69; N, 25.41%.

#### 2,2′-(2,4-Dioxoquinazolin-1,3(2*H*,4*H*)-diyl)bis(*N*′-((*E*)-2-oxo-1,2-diphenyl ethylidene)acetohydrazide) (9a)

To a cold solution of 3a (0.001 mol) in dry pyridine (10 ml), benzoylchloride (0.002 mmol) was added dropwise; the reaction mixture was stirred at room temperature for 1 h, then poured into diluted HCl. The solid formed was filtered off and recrystallized from ethanol to yield compounds 9a, as white powders. Yield 71%; mp: 284–286 °C; FT-IR (KBr, *ν*, cm^−1^) = 3460, 3217 (N–H stretching), 3062, 3031 (C–H aromatic), 2966, 2855 (C–H aliphatic), 1688, 1665 (C=O's), 1483 (C=N); ^1^H-NMR (400 MHz, DMSO-d_6_, *δ*, ppm) = 5.14 and 5.39 (s, 4H, 2CH_2_), 7.34–8.49 (m, 24H, Ar-H), 11.75 to 11.84 (s, 2H, 2NH); ^13^C-NMR (100 MHz, DMSO-d_6_, *δ*, ppm) = 44.59, 47.23, 114.80, 115.12, 123.24, 127.36, 128.35, 128.97, 130.49, 134.62, 136, 140.67, 144.75, 150.87, 161.43, 168.34; MS (EI): *m*/*z*(%) = 690 [M^+^]; anal. calcd for C_40_H_30_N_6_O_6_: C, 69.56%; H, 4.38; N, 12.17. Found: C, 69.56%; H, 4.39; N, 12.13.

### 2.2 *In vitro* antibacterial activity

Antimicrobial susceptibility and MIC of the newly molecules were determined against two G −ve and two G +ve bacteria. The studied pathogens were collected from Al-Azhar University, Egypt. They were cultivated in Mueller Hinton broth at 35 ± 2 °C for 24 h. The antimicrobial activity and MIC were performed as reported by Qader *et al.* (2021).^2,27–36^

### 2.3 Docking study

Docking studies^37^ were achieved on the new synthesized molecules 2–9a towards *S. aureus* tyrosyl-tRNA synthetase. The 3D structure of the target (PDB ID: 1JIJ) was retrieved from the Protein Data Bank (PDB).^38^ The 2D structures of the ligand molecules were sketched using ChemDraw Ultra 7.0, then converted to 3D structures using OpenBabel GUI tool.^39^ Before performing the docking approach, the 3D files of the ligand molecules and target were subjected to energy minimization by using AMBER Force Field^40^ in Open Babel and CHARMm Force Field^41^ in Discovery Studio 3.5, respectively, to obtain more stable confirmations. The molecular docking approach was performed by PyRx software tool.^25^ The 2D orientations of the docked molecules with the enzyme were generated by Discovery Studio visualizer software. Finally, the pharmacokinetics and ADMET properties of molecules were anticipated using AdmetSAR, SwissADME, and mol inspiration tools.

## 3. Results and discussion

### 3.1 Chemistry

**Scheme 1:**
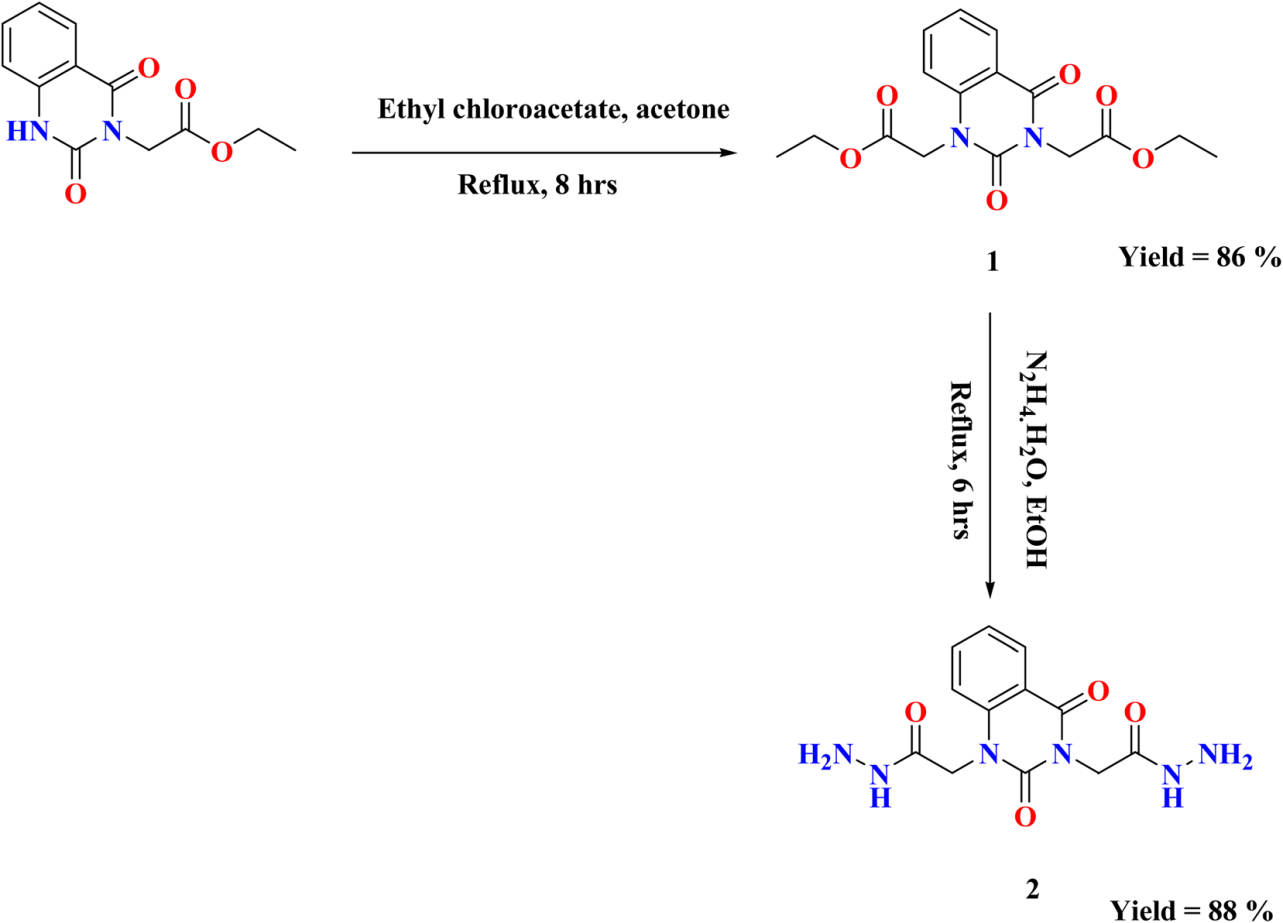
Synthesis of starting material 2.

**Scheme 2:**
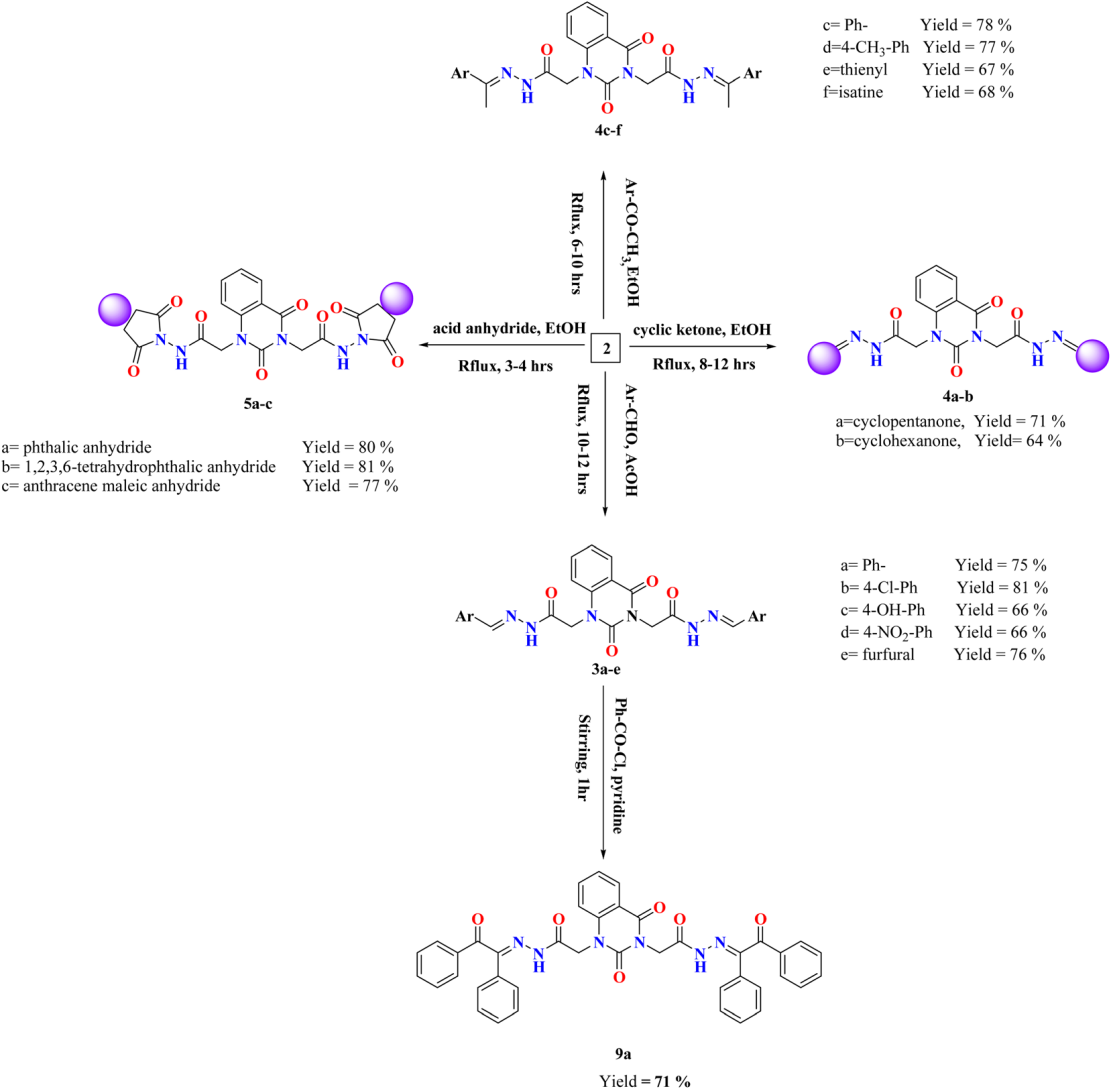
Synthesis of compounds 3–5 and 9a.

**Scheme 3:**
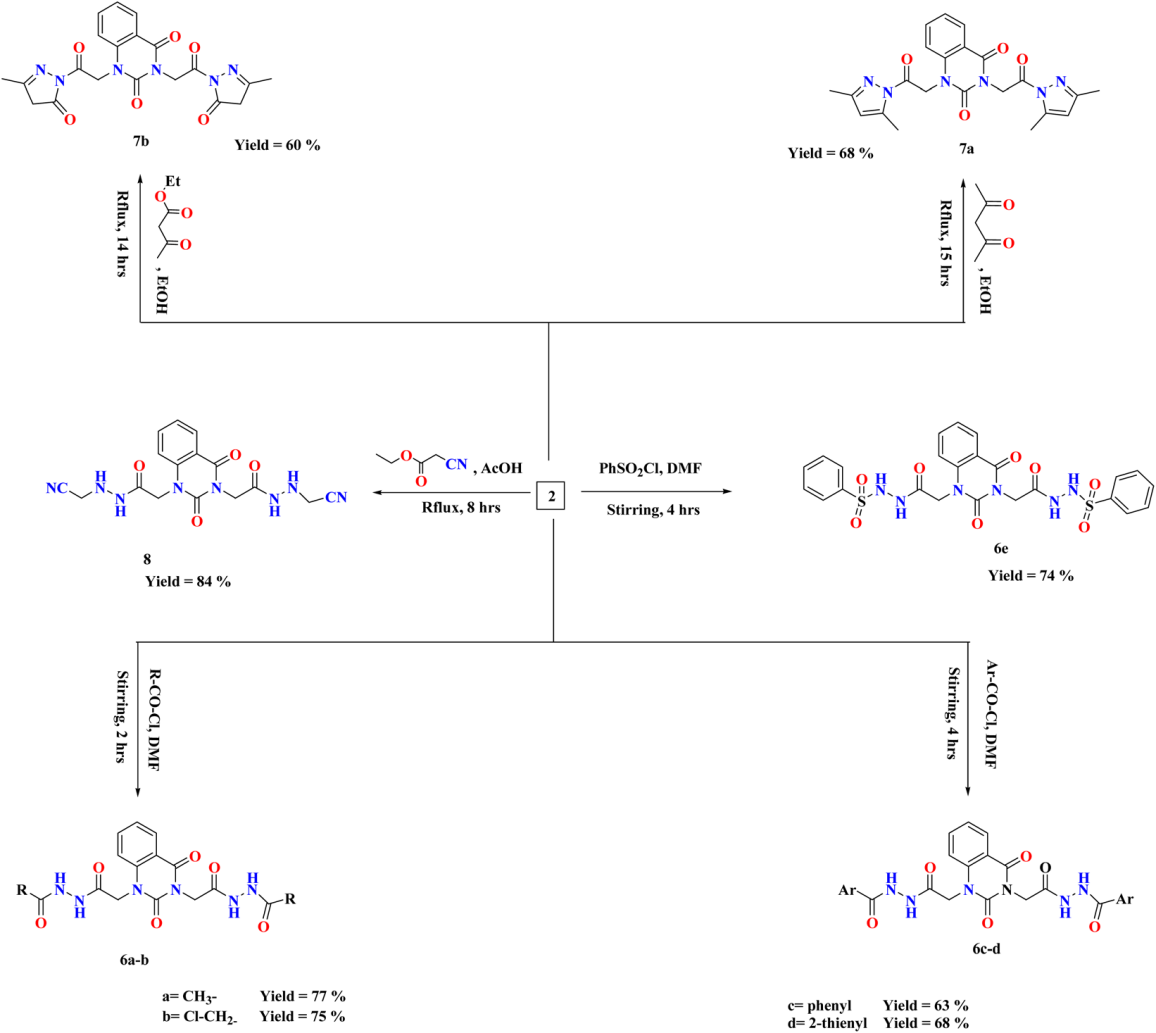
Synthesis of compounds 6–8.

On the basis of our previous findings, and in demanding to develop new antibacterial drug candidates, this context deals with the design, synthesis and antibacterial assessment of the quinazolin-2,4-dione analogues 2–9a. In the present study, 2,2′-(2,4-dioxoquinazoline-1,3(2*H*,4*H*)-diyl)di(aceto-hydrazide) 2 was utilized as a versatile precursor starting for preparation of some new heterocyclic moieties attached to quinazolindione scaffold 3–9a. The structural elucidation of the compounds was ascertained from spectral and elemental analyses. The overall pathway adopted for the preparation of new class of quinazolin-2,4-dione analogues linked to N-heterocycles and/or bioactive scaffolds is represented in Schemes 1–3.

As represented in Scheme 1, our starting 2 was synthesized through the reaction of the synthesized ester diethyl-2,2′-(2,4-dioxoquinazoline-1,3(2*H*,4*H*)-diyl)-diacetate 1 (ref. 26) with hydrazine hydrate in absolute ethanol, and its structure was confirmed by elemental and spectral analyses. The proton nuclear magnetic resonance (^1^H-NMR) spectrum of the ester 1 exhibited the characteristic triplet and quartet signals due to the ethoxy function of the ester groups at *δ* 1.28, 1.30, 4.22, and 4.29 ppm. Additionally, mass spectrum showed molecular ion peak at *m*/*z* = 334 corresponding to the chemical formula (C_16_H_18_N_2_O_6_). The fragment at *m*/*z* 162 referred to the quinazolin-2,4-dione ring.

The infrared (IR) spectrum of compound 2 showed strong to medium intensity bands appearing at *ν* 3250 and 1700 cm^−1^ which attributed to presence of NH_2_ and C=O groups. In addition, ^1^H-NMR spectrum revealed the disappearance of triplet and quartet signals related to the two ethyl groups of the starting di ester 1, in addition to existence of new characteristic singlet signals for two NH groups at *δ* 9.25 and 9.32 ppm, respectively. Furthermore, two singlet signals for two NH_2_ groups at *δ* 4.25 and 4.31 ppm, while the two methylene groups attached to quinazoline moiety appeared as singlet at *δ* 4.56 and 4.73 ppm and the four aromatic protons appeared multiplet in the range from *δ* 7.24–8.10 ppm. Mass spectrum recorded mass at *m*/*z* 306 which is corresponding to the chemical formula C_12_H_14_N_6_O_4_.

Various arylidene hydrazide derivatives (Schiff bases) 3a–e were synthesized in good to excellent yields by treatment of 2,2′-(2,4-dioxoquinazoline-1,3(2*H*,4*H*)-diyl)di(aceto hydrazide) 2 with various aromatic aldehydes such as, benzaldehyde, 4-hydroxybenzaldehyde, 4-chlorobenzaldehyde, 4-nitrobenzaldehyde and furfural, respectively (Scheme 2). The chemical structures of 3a–f were concluded from their IR, and ^1^H-NMR spectra, and elemental analysis. For instance, IR spectra of 3a–e represented characteristic absorption bands at 1570 cm^−1^ assigned for azomethine linkage. ^1^H-NMR spectra of all Schiff bases 3a–e showed a characteristic signal for azomethine proton (–NH–N=CH–) appeared at *δ* 6.83–8.23 ppm, which is in interference with aromatic protons. Moreover, the value appeared at *m*/*z* 482 showed the mass of the synthesized molecules 3a.

The treatment of di-hydrazide 2 with cycloaliphatic ketones such as cyclo-pentanone and/or cyclohexanone produced the relevant hydrazones (–NH–N=C-alicyclic ring) 4a–b, respectively.

Chemical shifts at the ranges of *δ* 1.71–2.39 and 1.60–2.52 ppm confirmed the presence of aliphatic CH_2_ groups in compounds 4a–b, respectively.

Reaction of (un)substituted acetophenones with of di-hydrazide 2 afforded hydrazone derivatives 4c–d, respectively. ^1^H-NMR of 4c, for example, showed new peaks at *δ* 2.80, 2.82 and 7.31–8.08 ppm related to two methyl groups and aromatic protons, respectively.

Condensation of the compound 2 with 2-acetylthiophene and Isatin yielded the comparable hydrazone derivatives 4e–f, respectively. IR spectrum of 4f, as an example, showed the presence of bands at *ν* 3051 and 1625 cm^−1^ characteristic for functional groups Ar-H and C=N, respectively. ^1^H-NMR of 4f exhibited characteristic signals at *δ* 6.96–8.13 and 12.73 ppm assignable to the aromatic protons and NH group, respectively.

Upon condensation of 2 with acid anhydrides such as, phthalic anhydride, tetrachlorophthalic anhydride and/or anthracene maleic anhydride in glacial AcOH, the imide derivatives 5a–c, respectively, were afforded in good yields. ^1^H-NMR of 5a, for example, indicated the disappearance of two singlet peaks related to two amino groups of compound 2 and appearance of characteristic peak at the region *δ* 7.33–8.13 assigned for aromatic protons of 5a. Further, the mass spectral data of 5a exhibited a molecular ion peak at *m*/*z* 566 which agreed with the molecular formula C_28_H_18_N_6_O_8_.

The reactions of compound 2 with aliphatic/aromatic acid chlorides and/or benzene sulphonyl chloride in DMF afforded amide derivatives 6a–e, respectively. Compound 6a was structurally elucidated by spectral and elemental analyses. ^1^H-NMR of 6a displayed characteristic peaks at *δ* 2.78 ppm for two new methyl groups. For compound 6d, its ^1^H-NMR exhibited the characteristic (AMX) signals for thiophene ring at *δ* 7.32–8.09 ppm, which are in interference with aromatic protons.

**Scheme 4:**
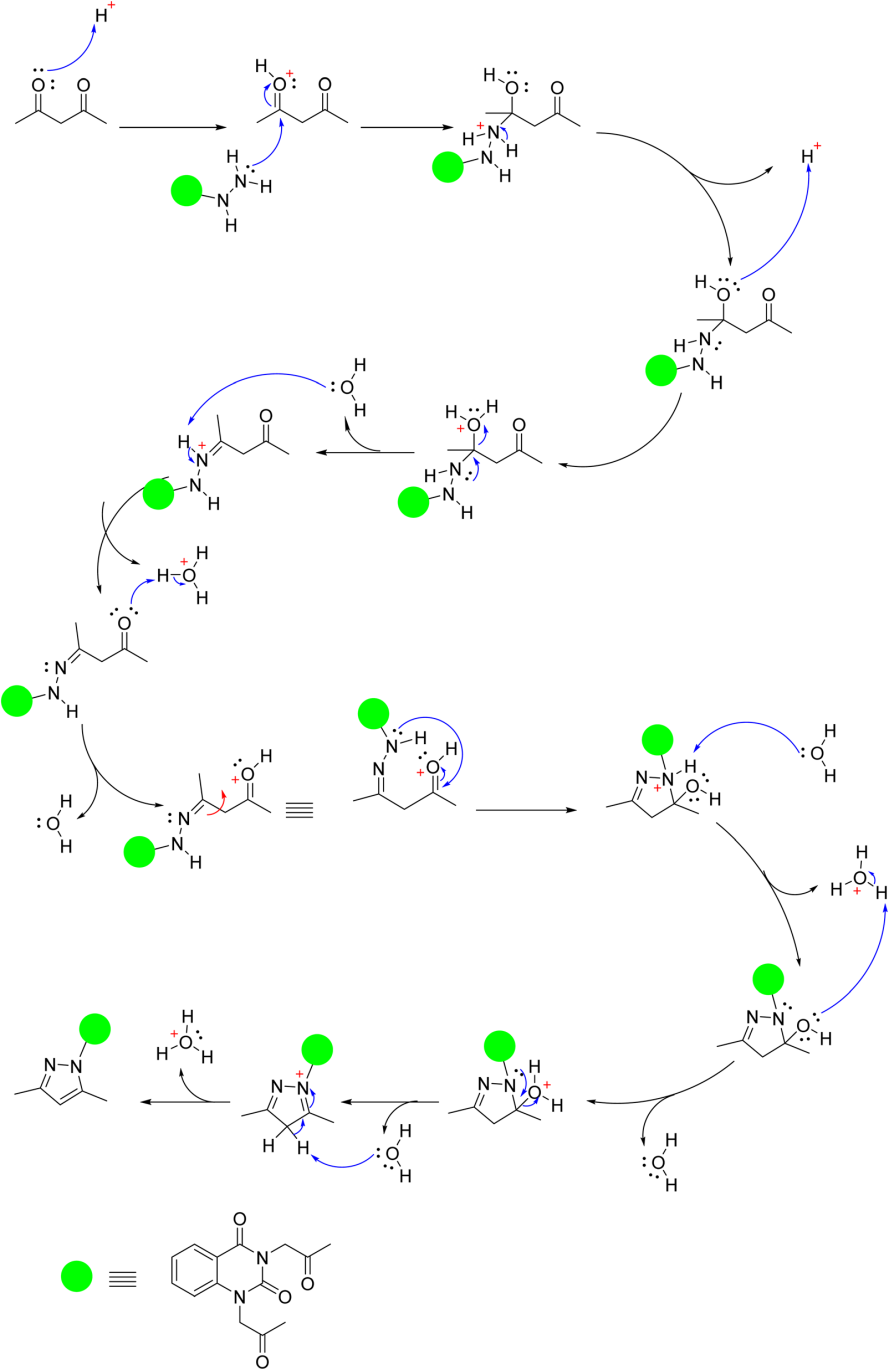
Plausible mechanism for the synthesis of compound 7a.

Moreover, quinazolin-2,4-dione containing pyrazole and pyrazolone moieties 7a–b were synthesized based on condensation of 2 with active methylene compounds such as acetylacetone and ethyl acetoacetate *via* Knorr pyrazole reaction, as declared in Scheme 3. Cyclization of 7a–b was confirmed by spectral and microanalysis. Presence of various functional groups in the newly synthesized compound 7a–b was interpreted by IR and NMR spectra. For compound 7a, as example, IR spectrum showed band appearing at *ν* 1625 cm^−1^ which attributed to presence of functional group C=N, along with the disappearance of absorption band assigned for NH_2_ group. On the other hand, ^1^H-NMR spectrum exhibited four singlet peaks at *δ* 1.75, 1.76, 2.03, 2.06 and ppm attributed to new four methyl groups in pyrazole rings, along with, two singlet peaks at *δ* 6.51 and 6.56 ppm assigned for two methine groups in pyrazole rings. In addition, the mass spectral data showed [M^+^] at *m*/*z* 434 which agreed with the molecular formula of the synthesized molecules 7a. A mechanism for the synthesis of 7a from compound 2 and 1,3-dicarbonyl compound (acetyl acetone) catalyzed by acetic acid is represented in Scheme 4. Initially, this mechanism involved a protonation of acetyl acetone, the produced carbanion then underwent a nucleophilic attack of the free doublet of nitrogen to yield the imine. On the other hand, the other nitrogen atom attacks the second carbonyl group of acetyl acetone which was protonated by the acid to furnish a second imine group. Finally, the diimine was deprotonated to yield the pyrazole product.

Treatment of 2 with ethyl cyanoacetate in glacial AcOH afforded the desired compound 8, which was structurally confirmed by existence of sharp band at 2337 cm^−1^ assigned for cyano group in its IR spectrum. Further, the value appeared at *m*/*z* 440 showed the expected mass of product 8.

Else way, Schiff base 3a was then allowed to react with benzoyl chloride in pyridine to yield compound 9a. The latter product was structurally confirmed by ^1^H-NMR spectrum which exhibited the absence of a characteristic peak for azomethine proton (–NH–N=CH–), along with increasing the signals of aromatic area according to presence of extra two phenyl rings.

The spectral data of all synthesized compounds are given as a ESI.† In addition, InChI code, SMILES and solubility of all compounds are also provided in ESI.†

### 3.2 Antibacterial activity

The spread of the microbial chemotherapeutic resistance is recognized as the most serious global health problem, which minimize the potency of the antibiotics. Therefore, herein, the antibacterial efficacy of a new series of heterocyclic compounds against various pathogenic microbes was determined. The minimum inhibitory concentration (MIC) of the tested molecules was mentioned in Table 1.

**Table 1:** Antibacterial efficacy and MIC of the molecules 1–9a and ciprofloxacin as control, ND: not determined

Sample no.	Minimum Inhibitory Concentration (MIC, μg mL^−1^)			
	*Escherichia coli*	*Pseudomonas aeruginosa*	*Bacillus subtilis*	*Staphylococcus aureus*
1	120	80	ND	40
2	40	20	120	40
3a	ND	ND	ND	ND
3b	40	60	ND	ND
3c	2.5	5	10	5
3d	ND	ND	ND	ND
3e	20	40	ND	ND
4a	ND	ND	ND	ND
4b	120	40	80	80
4c	20	20	ND	ND
4d	ND	ND	ND	ND
4e	10	40	80	120
4f	ND	ND	ND	ND
5a	20	15	ND	ND
5b	ND	ND	20	15
5c	ND	ND	ND	ND
6a	ND	ND	ND	ND
6b	10	15	7	12
6c	ND	ND	ND	ND
6d	40	80	40	40
6e	120	40	80	80
7a	140	160	ND	80
7b	20	40	10	15
8	10	20	20	15
9a	10	40	80	120
Ciprofloxacin	5	7	2.5	1.25

The lowest concentration with no observation of pathogenic microbial growth is known as MIC. It was noticed from the results that compound 3c revealed significant antimicrobial efficacy against all the tested pathogenic strains at low concentration compared to ciprofloxacin ranging from 2.5 to 10 μg ml^−1^. The results represented also, that compounds 6b and 8 exhibited high antibacterial potency towards all strains of tested pathogenic bacteria with low concentrations ranged from 5 to 20 μg ml^−1^. Meanwhile, compounds 4c, 5a declared significant antimicrobial effect at low concentrations against the two tested G −ve bacteria. Additionally, compound 5b showed strong antibacterial effect at relatively low concentrations against the two tested G +ve bacteria.

### 3.3 *In silico* docking study

**Fig. 3:**
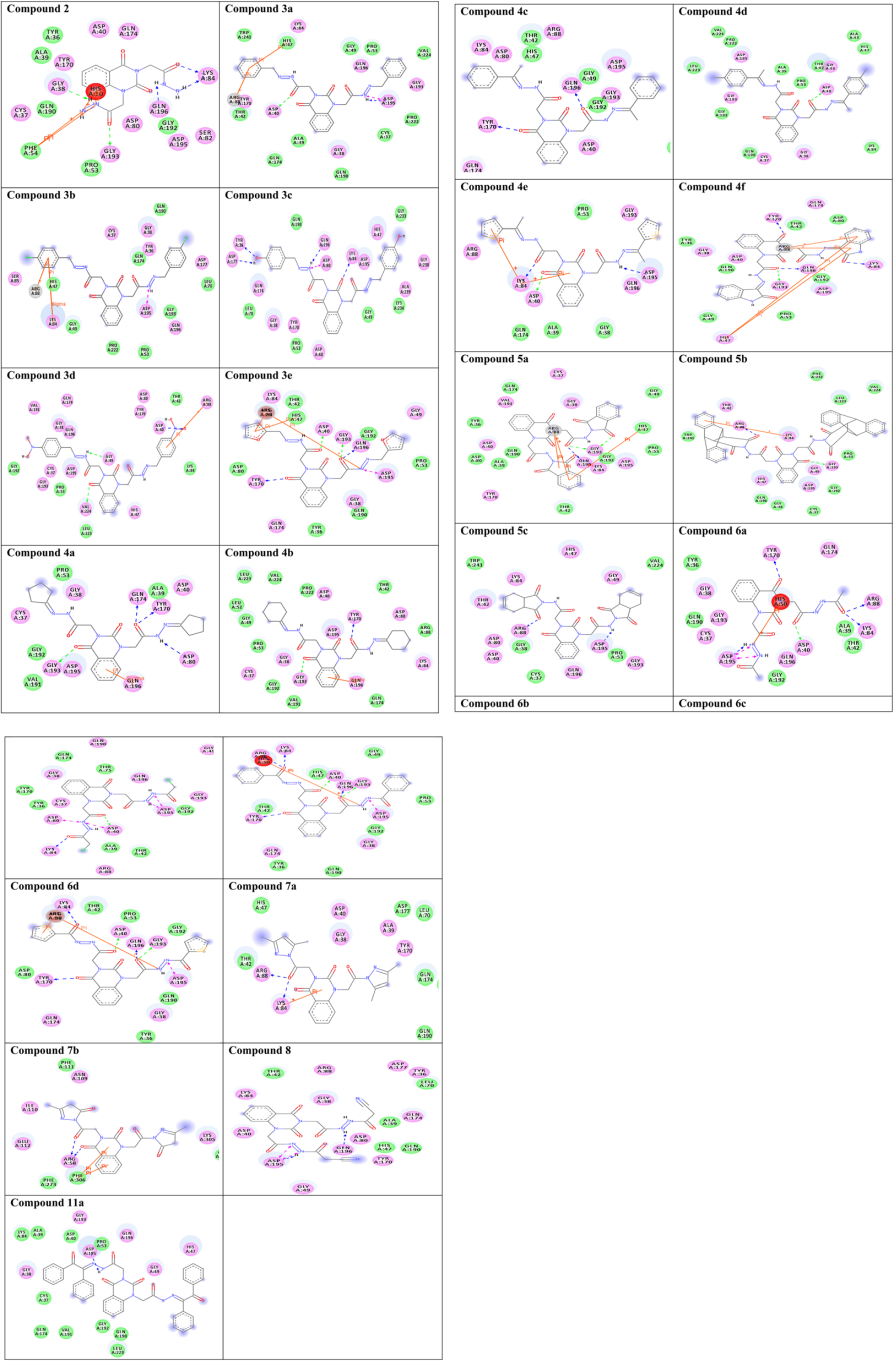
Two-dimensional (2D) orientation of complexes. H-bond interactions are represented in green, blue and pink dotted lines. Pi-stacking are represented in orange lines.

To gain insight into molecular interactions and docking scores, the molecular docking study^36,42,43^ was carried out between the newly ligand molecules and the target enzyme. Herein, *S. aureus* tyrosyl-tRNA synthetase is a well-recognized attractive therapeutic target for antibacterial drug design.^44^ In the present study, the docking approach used *S. aureus* tyrosyl-tRNA synthetase to figure out the action mode of the ligand molecules as antibacterial agents. Target compounds 2–9a exhibited similar fitness to co-crystalized ligand and reference drug into active site of the target enzyme. The docking pattern was interestingly achieved similar to the reported position completed by the co-crystalized ligand with the active site of the target. The results showed that compound 3c exhibited the most promising bacterial inhibitory effect and the best binding affinity against the target (Δ*G* = −14.2 kcal mol^−1^). It engaged in five hydrogen bonds *via* its hydroxyl, azomethine, and carbonyl groups with the essential residues TYR36, ASP80, LYS84, ASP177, and GLN196. Considering the structure within this series, the SAR analysis demonstrated that the presence of two hydroxyl groups on *para*-positions of two phenyl rings (compound 3c) exhibited significantly greater inhibition effect. From the results tabulated in Table 2, compound 3c is considered as a highly antibacterial potency and might be act as an antibiotic. The 2D molecular interaction network between all ligand molecules 2–9a and *S. aureus* tyrosyl-tRNA synthetase are shown in Fig. 3.

**Table 2:** The binding energies (kcal mol^−1^) for all docked compounds 2–9a, reference drug, and co-crystalized ligand against the target

No.	Binding energy (kcal mol^−1^)	Docked complex (amino acid–ligand) interactions	Distance (Å)
2	−8.6	**H-bonds**	
		GLY38:O–compound 2	2.24
		LYS84:NZ–compound 2	2.98
		LYS84:NZ–compound 2	2.95
		GLY193:N–compound 2	2.90
		GLN196:OE1–compound 2	2.32
		**Arene-cation**	
		PHE54–compound 2	5.12
		PHE54–compound 2	4.28
		HIS50–compound 2	5.65
		HIS50–compound 2	4.92

3a	−10.6	**H-bonds**	
		ASP40:N–compound 3a	2.99
		ASP195:OD1–compound 3a	2.32
		ASP195:OD2–compound 3a	2.27
		**Arene–arene**	
		HIS47–compound 3a	4.12
		**Arene-cation**	
		ARG88:NH1–compound 3a	5.59

3b	−9.1	**H-bonds**	
		ASP195:OD1–compound 3b	2.52
		**Arene-cation**	
		LYS84:NZ–compound 3b	5.28
		ARG88:NH1–compound 3b	5.90
		**Arene-sigma**	
		LYS84:CG–compound 3b	3.76

3c	−14.2	**H-bonds**	
		TYR36:OH–compound 3c	2.73
		ASP80:OD2–compound 3c	2.25
		LYS84:NZ–compound 3c	3.00
		ASP177:OD1–compound 3c	2.18
		GLN196:OE1–compound 3c	2.38

3d	−8.8	**H-bonds**	
		GLY49:O–compound 3d	2.32
		ASP40:OD1–compound 3d	3.04
		VAL224:N–compound 3d	2.88
		**arene-cation**	
		ARG88:NH1–compound 3d	5.44

3e	−9.9	**H-bonds**	
		ASP40:N–compound 3e	3.10
		TYR170:OH–compound 3e	2.92
		GLY193:N–compound 3e	2.72
		GLN196:NE2–compound 3e	2.85
		**Arene–arene**	
		HIS47–compound 3e	4.59
		**Arene-cation**	
		HIS50–compound 3e	5.12
		ARG88:NH1–compound 3e	5.07

4a	−10.0	**H-bonds**	
		TYR170:OH–compound 4a	2.80
		GLN174:NE2–compound 4a	3.06
		GLY193:N–compound 4a	3.10
		ASP80:OD2–compound 4a	2.25
		**Arene-sigma**	
		GLN196:CG–compound 4a	3.26

4b	−10.7	**H-bonds**	
		TYR170:OH–compound 4b	2.99
		GLY193:N–compound 4b	2.98
		**Arene-sigma**	
		GLN196:CG–compound 4b	3.36

4c	−10.8	**H-bonds**	
		TYR170:OH–compound 4c	2.85
		GLY193:N–compound 4c	2.76
		GLN196:NE2–compound 4c	2.89

4d	−10.3	**H-bonds**	
		ASP40:N–compound 4d	

4e	−9.1	**H-bonds**	
		ASP40:N–compound 4e	2.96
		LYS84:NZ–compound 4e	3.10
		ASP195:OD1–compound 4e	2.18
		**Arene-cation**	
		LYS84:NZ–compound 4e	5.26
		LYS84:NZ–compound 4e	4.77

4f	−12.0	**H-bonds**	
		LYS84:NZ–compound 4f	3.00
		TYR170:OH–compound 4f	2.92
		GLY193:N–compound 4f	2.77
		GLN196:NE2–compound 4f	2.95
		**Arene–arene**	
		HIS47–compound 4f	5.82
		HIS47–compound 4f	4.52
		**Arene-cation**	
		ARG88:NH1–compound 4f	5.29
		ARG88:NH1–compound 4f	5.12

5a	−11.9	**H-bonds**	
		LYS84:NZ–compound 5a	3.14
		GLY193:N–compound 5a	2.77
		GLY193:N–compound 5a	3.02
		GLN196:NE2–compound 5a	2.99
		**Arene–arene**	
		HIS47–compound 5a	5.62
		**Arene-cation**	
		ARG88:NH1–compound 5a	5.11
		ARG88:NH1–compound 5a	5.16

5b	−12.3	**H-bonds**	
		ARG88:NH1–compound 5b	2.65
		GLY193:N–compound 5b	1.99
		**Arene-cation**	
		LYS84:NZ–compound 5b	5.19

5c	−11.6	**H-bonds**	
		ARG88:NH1–compound 5c	2.39
		ASP195:OD2–compound 5c	2.58

6a	−9.6	**H-bonds**	
		ASP40:N–compound 6a	2.91
		LYS84:NZ–compound 6a	2.80
		ARG88:NH1–compound 6a	3.05
		TYR170:OH–compound 6a	3.10
		ASP195:OD1–compound 6a	2.39
		**Arene-cation**	
		HIS50–compound 6a	

6b	−9.3	**H-bonds**	
		ASP40:N–compound 6b	2.82
		LYS84:NZ–compound 6b	3.19

6c	−10.7	**H-bonds**	
		ASP40:N–compound 6c	3.04
		LYS84:NZ–compound 6c	2.85
		ARG88:NH1–compound 6c	3.00
		TYR170:OH–compound 6c	3.00
		GLY193:N–compound 6c	2.80
		GLN196:NE2–compound 6c	2.93
		**Arene-cation**	
		HIS50–compound 6c	5.16

6d	−10.2	**H-bonds**	
		ASP40:N–compound 6d	3.00
		LYS84:NZ–compound 6d	2.84
		TYR170:OH–compound 6d	2.91
		GLY193:N–compound 6d	2.79
		GLN196:NE2–compound 6d	2.89
		**Arene-cation**	
		ARG88:NH2–compound 6d	5.79

7a	−10.3	**H-bonds**	
		LYS84:NZ–compound 7a	2.80
		ARG88:NH1–compound 7a	3.05
		**Arene-cation**	
		LYS84:NZ–compound 7a	5.95

7b	−8.5	**H-bonds**	
		ARG58:NH1–compound 7b	2.55
		ARG58:NH1–compound 7b	2.37
		**Arene–arene**	
		PHE306–compound 7b	5.24
		PHE306–compound 7b	5.27

8	−8.6	**H-bonds**	
		ASP195:OD2–compound 8	2.31
		GLN196:OE1–compound 8	2.30

9a	−9.6	**H-bonds**	
		ASP195:OD1–compound 11a	2.30

Reference drug	−9.0	**H-bonds**	
		GLY193:N–reference drug	2.11

Co-crystalized ligand	−8.2	**H-bonds**	
		GLY38:O–co-crystalized ligand	2.37
		ASP40:N co-crystalized ligand	2.79
		GLY193:N–co-crystalized ligand	3.07
		ASP195:OD1–co-crystalized ligand	3.11
		TYR170:OH–co-crystalized ligand	2.94
		ASP195:OD1–co-crystalized ligand	2.23
		GLN196:NE2–co-crystalized ligand	2.55
		VAL224:N–co-crystalized ligand	

**Fig. 4:**
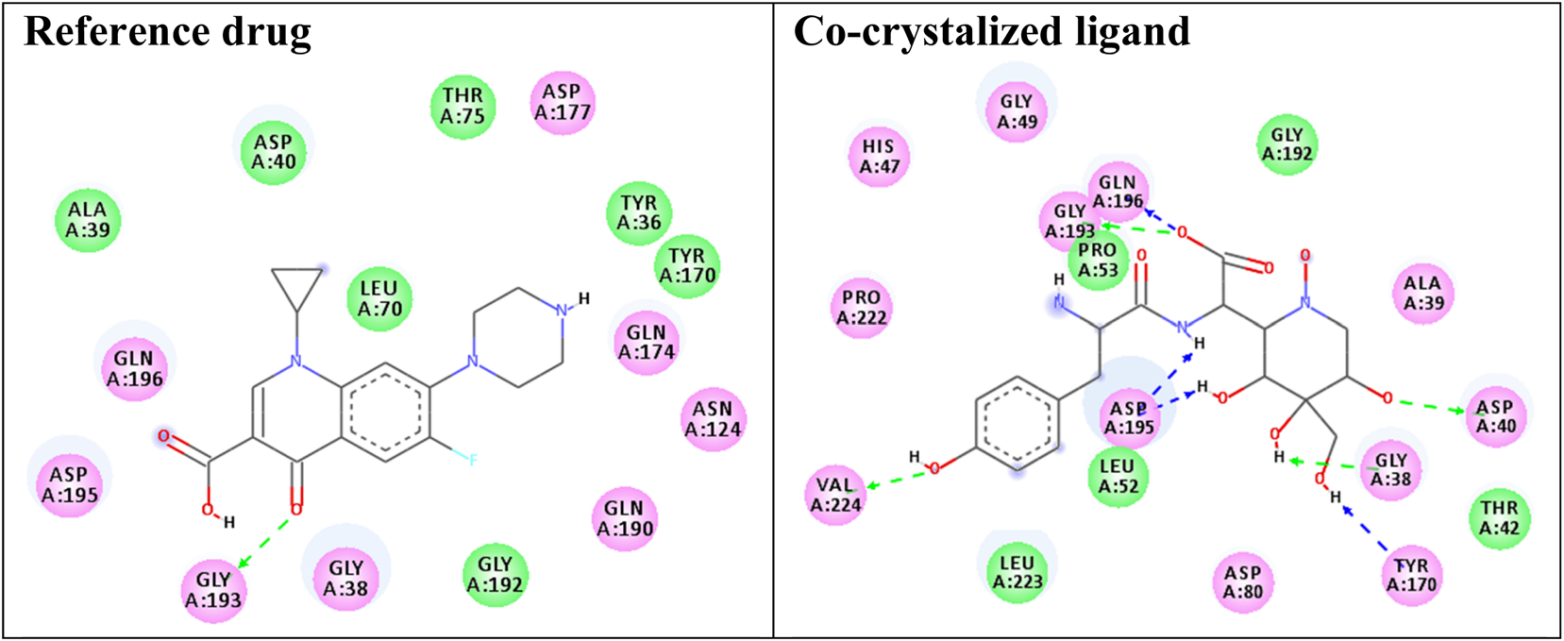
Two-dimensional (2D) orientation of the reference drug and co-crystalized ligand with the target enzyme *S. aureus* tyrosyl-tRNA synthetase. H-bond interactions are shown in green, and blue dotted lines. Pi-stacking interactions are represented in orange lines.

Redocking of the co-crystalized ligand [2-amino-3-(4-hydroxy-phenyl)-propionylamino]-(1,3,4,5-tetrahydroxy-4-hydroxymethyl-piperidin-2-yl)-acetic acid was carried out to validate the docking process (RMSD < 2 Å) and the results showed similar fitness to the docked compounds. The co-crystalized ligand exhibited binding affinity of −8.2 kcal mol^−1^, and it showed H-bond interactions with the residues GLY38, ASP40, TYR170, GLY193, ASP195, GLN196, and VAL224, as shown in Fig. 4. In addition, the reference drug ciprofloxacin (binding affinity = −9.0 kcal mol^−1^) docked to the target though H-bond interaction with the residue GLY193 at the distance 2.11 Å, as presented in Fig. 4.

**Fig. 5:**
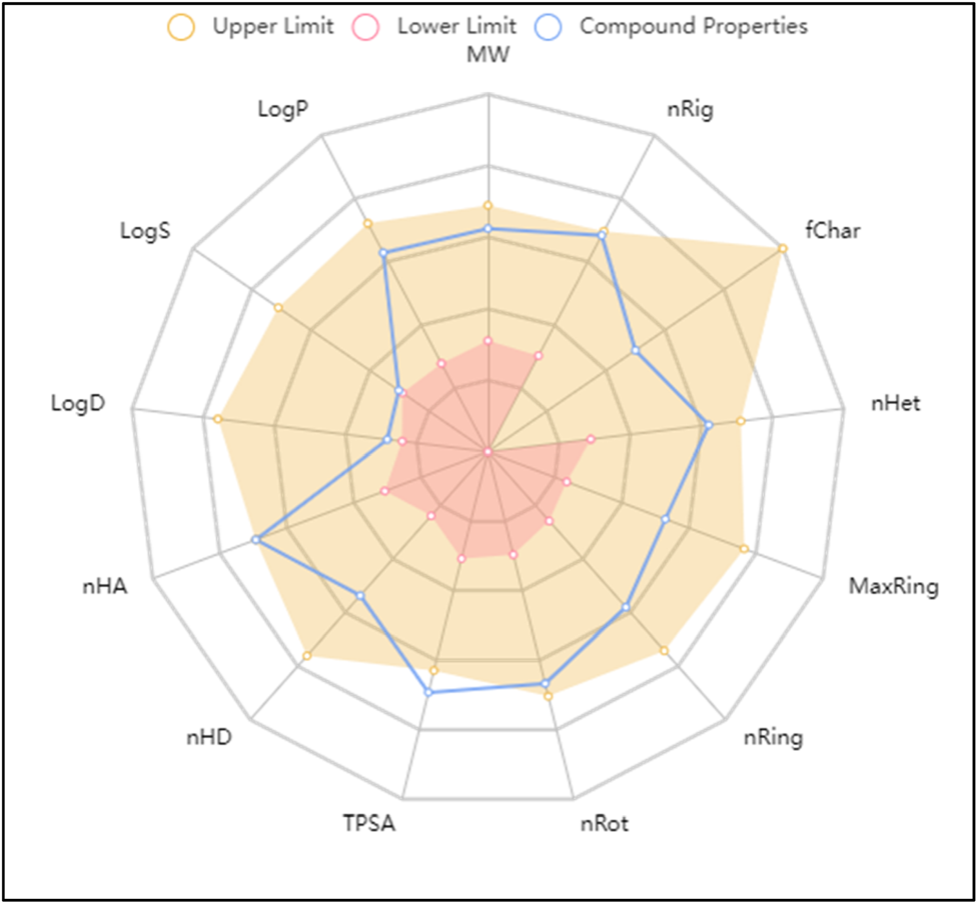
*In silico* ADMET properties of compound 3c predicted by ADMETlab tool.

The molecular and pharmacokinetics properties of the most active compound 3c and the known antibiotic, ciprofloxacin were calculated by SwissADME, admetSAR, and mol inspiration web servers. The bioavailability and physicochemical properties of 3c were evaluated using ADMETlab tool, by plotting radar showing 13 properties (Fig. 5). According to Lipinski's rule, most of the tested compounds satisfied with the Ro5 (no. of violations ≤ 1) and meet all criteria for good permeability and acceptable oral bioavailability, displayed rotatable bonds number in the range <10, which means they are flexible. Their HBA and HBD values were in the satisfied range, gave them higher solubility in cellular membranes. The log *p* values less than 5, reflected good lipophilicity character, as tabulated in Table 3. Furthermore, the ADMET parameters exhibited that the molecules had better Human Intestinal Absorption (% HIA) scores; indicating that they could be better absorbed by the human intestine. The target compounds 2–9a does not pass blood–brain barrier that indicating their good CNS safety profile. Finally, all showed negative AMES toxicity and carcinogenicity test; indicating their safety.

**Table 3:** ADME and drug-likeness properties of the docked ligand molecules 2–9a, and reference drug. log *p*, logarithm ratio of partition coefficient between *n*-octanol and water; TPSA, topological polar surface area; MW, molecular weight; HBA, number of hydrogen bond acceptors; HBD, number of hydrogen bond donors; *N* rotatable, number of rotatable bonds

	Molecular weight (g mol^−1^)	BBB permeant	GI absorption	% Human Intestinal Absorption (HIA+)	log *p*	TPSA A^2^	HBA	HBD	*N* rotatable	*N* violations	Bioavailability score	AMES toxicity	Carcino-genicity
**Ref. range**	**130–500**			**<25 poor**	**≤5**	**≤140**	**2.0–20.0**	**0.0–6.0**	**≤10**	**≤1**		**Nontoxic**	**Non-carcinogenic**
				**>80 high**									
2	306.28	No	Low	98.50	−3.92	154.25	6	4	6	0	0.55	Nontoxic	Non-carcinogenic
3a	482.49	No	High	92.12	2.91	126.93	6	2	8	0	0.55	Nontoxic	Non-carcinogenic
3b	551.39	No	High	93.28	4.27	126.93	10	2	8	1	0.55	Nontoxic	Non-carcinogenic
3c	514.50	No	Low	91.71	1.95	137.39	8	4	10	1	0.17	Nontoxic	Non-carcinogenic
3d	572.49	No	Low	76.14	2.38	218.58	10	2	12	2	0.17	Nontoxic	Non-carcinogenic
3e	462.41	No	Low	96.93	1.43	153.20	8	2	10	0	0.55	Nontoxic	Non-carcinogenic
4a	438.48	No	High	86.38	1.62	126.93	6	2	8	0	0.55	Nontoxic	Non-carcinogenic
4b	466.53	No	High	79.46	2.63	126.93	6	2	8	0	0.55	Nontoxic	Non-carcinogenic
4c	510.54	No	High	93.88	2.74	126.93	6	2	10	1	0.55	Nontoxic	Non-carcinogenic
4d	538.60	No	High	93.32	3.64	126.93	6	2	10	1	0.55	Nontoxic	Non-carcinogenic
4e	522.60	No	High	96.80	2.54	126.93	6	2	10	1	0.55	Nontoxic	Non-carcinogenic
4f	564.51	No	High	85.53	1.38	185.12	8	4	8	2	0.55	Nontoxic	Non-carcinogenic
5a	566.48	No	Low	96.96	1.35	180.36	8	2	8	2	0.17	Nontoxic	Non-carcinogenic
5b	822.83	No	Low	96.96	2.98	176.97	8	2	8	2	0.17	Nontoxic	Non-carcinogenic
5c	574.54	No	Low	97.50	−0.68	176.97	8	2	8	2	0.17	Nontoxic	Non-carcinogenic
6a	390.35	No	Low	98.68	−2.78	160.40	6	4	10	1	0.55	Nontoxic	Non-carcinogenic
6b	459.24	No	Low	99.50	−1.65	160.40	6	4	12	1	0.55	Nontoxic	Non-carcinogenic
6c	514.49	No	Low	98.28	0.56	160.40	6	4	12	2	0.17	Nontoxic	Non-carcinogenic
6d	526.54	No	Low	99.12	0.36	160.40	6	4	12	2	0.17	Nontoxic	Non-carcinogenic
7a	434.45	No	High	99.29	1.55	113.78	6	0	6	0	0.55	Nontoxic	Non-carcinogenic
7b	438.39	No	Low	99.40	−1.05	143.50	8	0	6	1	0.55	Nontoxic	Non-carcinogenic
8	440.37	No	Low	98.05	−3.95	207.99	8	4	12	1	0.55	Nontoxic	Non-carcinogenic
9a	690.72	No	Low	96.54	4.28	161.08	8	2	14	2	0.17	Nontoxic	Non-carcinogenic
Ref. drug	331.35	No	High	97.95	−70.0	74.57	5	2	3	0	0.55	Nontoxic	Non-carcinogenic

## 4. Conclusion

In summary, a series of hybrid structures 2–9a containing quinazolin-2,4-dione analogue attached to N-heterocyclic cores such as pyrrolidine-2,5-dione, pyrazole and oxadiazole and/or bioactive scaffolds such as hydrazone, amide, sulfonamide, azomethine, and thiourea linkage was designed as potential antibacterial agents, synthesized, and investigated for their antibacterial activity. Predominantly, the *in vitro* studies revealed that the compound 3c exhibited strong significant antibacterial efficacy against all the tested pathogenic strains at low concentrations comparing with the tested standard drug. The findings were also connected with the molecular docking studies which concluded that compound 3c showed good inhibitory activity against the target *S. aureus* tyrosyl-tRNA synthetase. The presence of two hydroxyl groups on phenyl rings at positions-4 in the latter compound 3c seems to be essential for antibacterial activity.

## Data availability

The data that support the findings of this study are included within the article and ESI.†

## Author contributions

A. H. A., M. O., H. R. M. R., M. M. T. and A. M. A. made a significant contribution to the work reported, whether that is in the study design, analysis, and interpretation. A. H. A. and M. O. are responsible for the synthesis of products. Finally, A. H. A., M. O. and H. R. M. R. took part in writing, revising or critically reviewing the article. All authors gave final approval of the version to be published; have agreed on the journal to which the article has been submitted.

## Conflicts of interest

The authors declare no conflict of interest.

## Supplementary Material

RA-013-D2RA06527D-s001
