# Supplementary material for: Design, synthetic approach, in silico molecular docking and antibacterial activity of quinazolin-2,4-dione hybrids bearing bioactive scaffolds
Source: RSC Adv. 2022 Dec 21;13(1):292–308. doi: 10.1039/d2ra06527d (PMC9769111; doi:10.1039/d2ra06527d)
Supplement: RA-013-D2RA06527D-s001 [file RA-013-D2RA06527D-s001.pdf]

Electronic Supplementary Material (ESI) for RSC Advances.
This journal is © The Royal Society of Chemistry 2022

-

Supplementary data file

Design, Synthetic Approach, In silico Molecular Docking and Antibacterial Activity of Quinazolin-2,4-dione Hybrids Bearing Bioactive Scaffolds

-Compound (1):

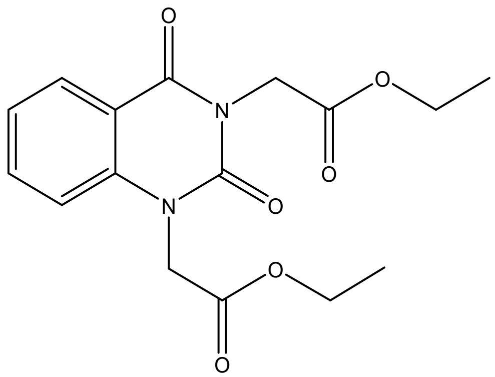

1

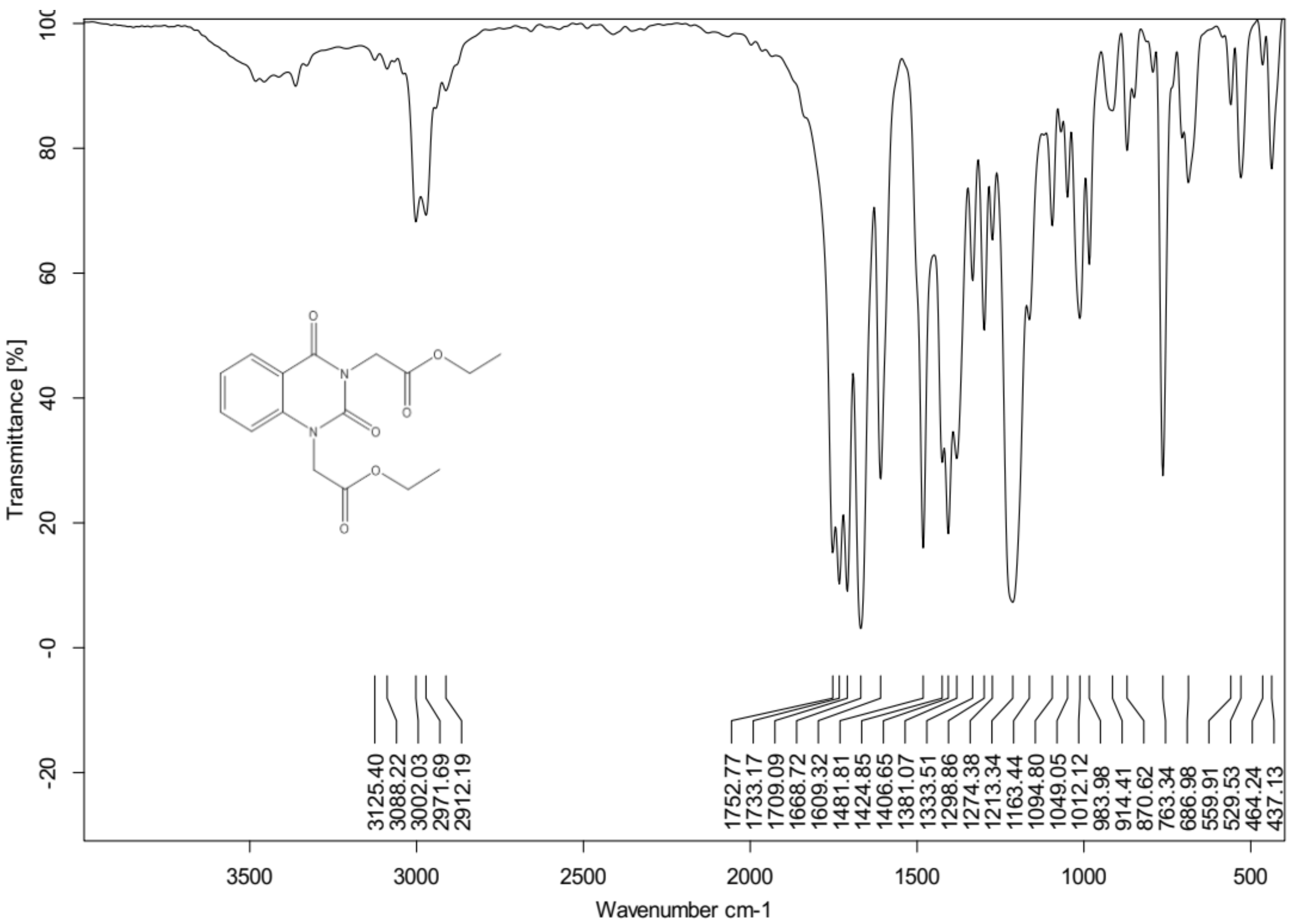

Figure . ^{1}H-NMR of compound 1 .

-

-

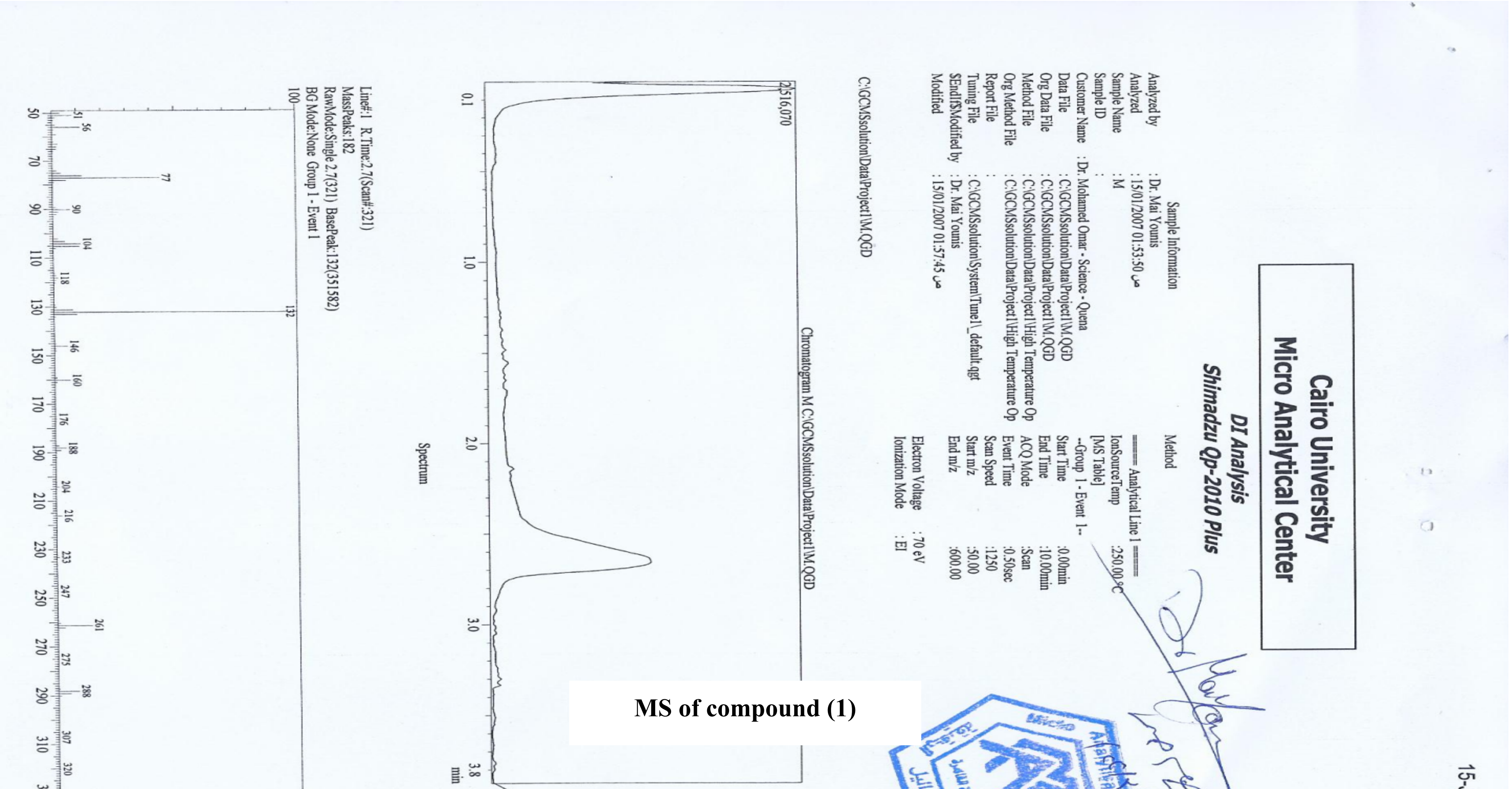

MS of compound (1)

-

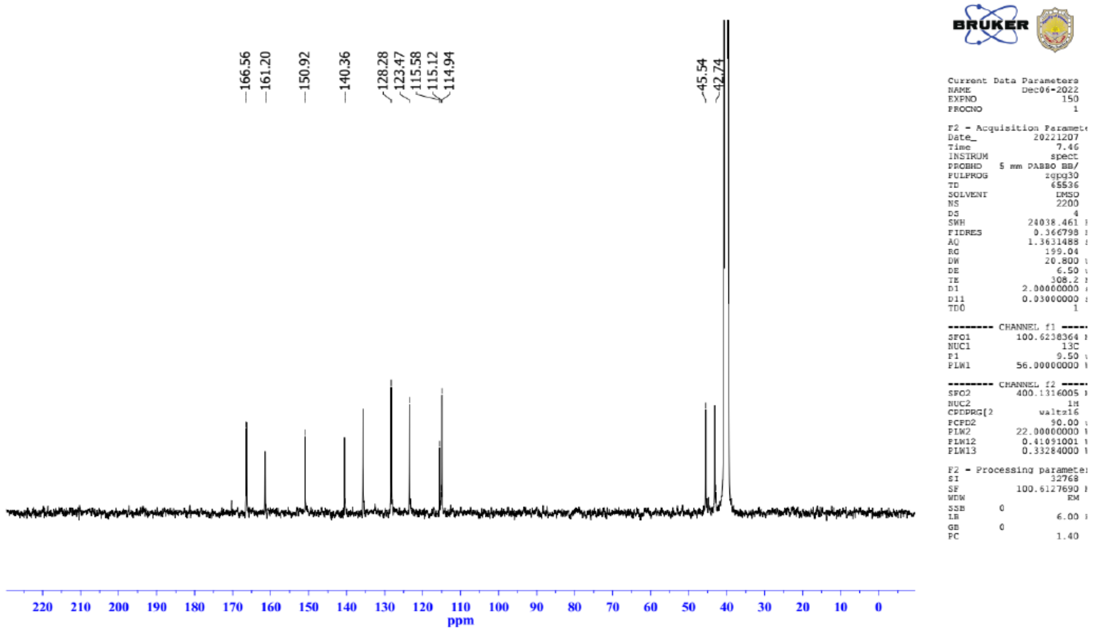

^{13}C-NMR (100 MHz, DMSO) of compound (1)

-

-Compound (2):

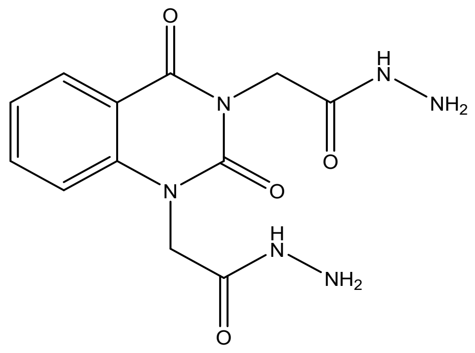

2

2

-

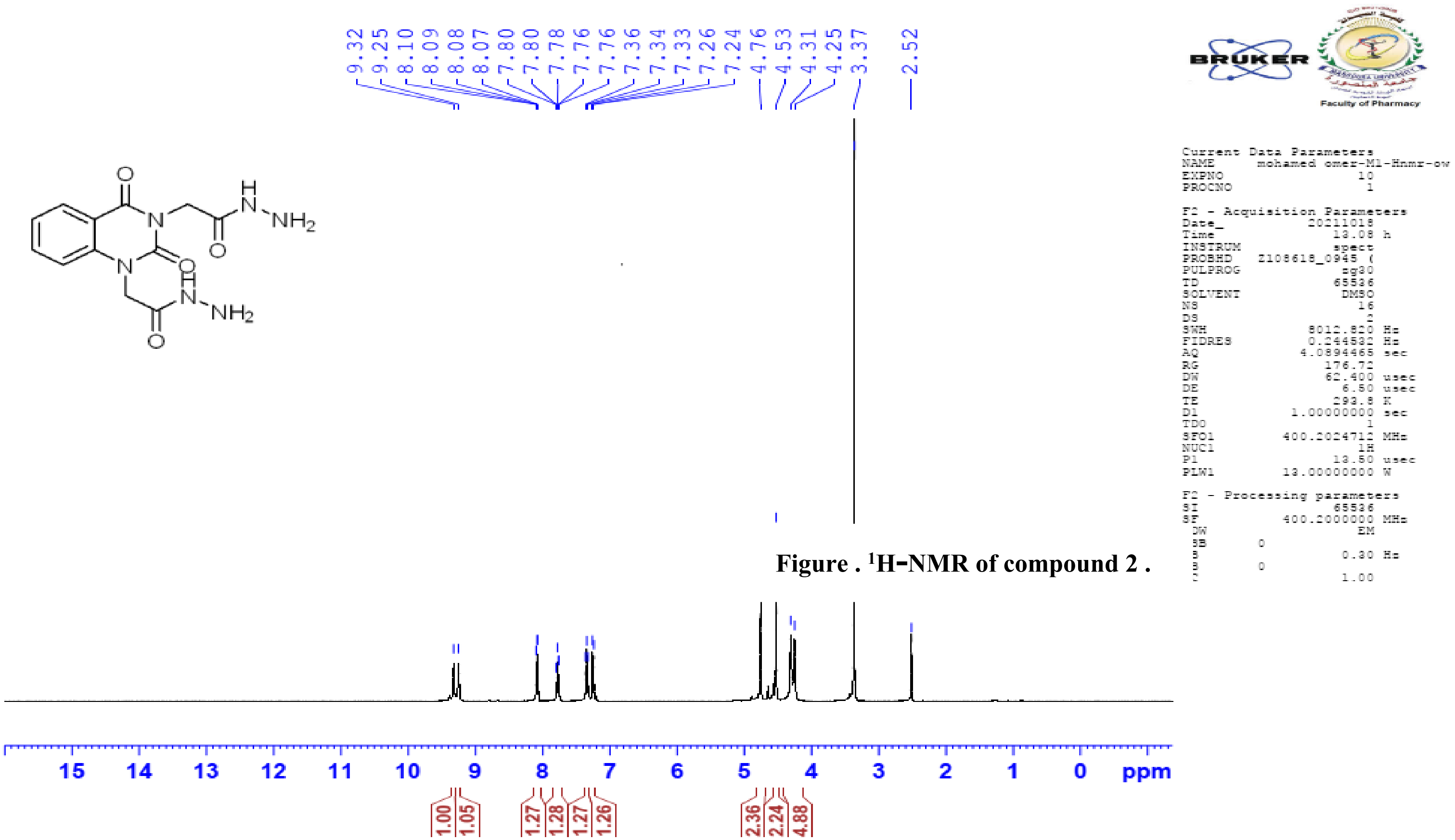

Figure . ^{1}H-NMR of compound 2 .

-

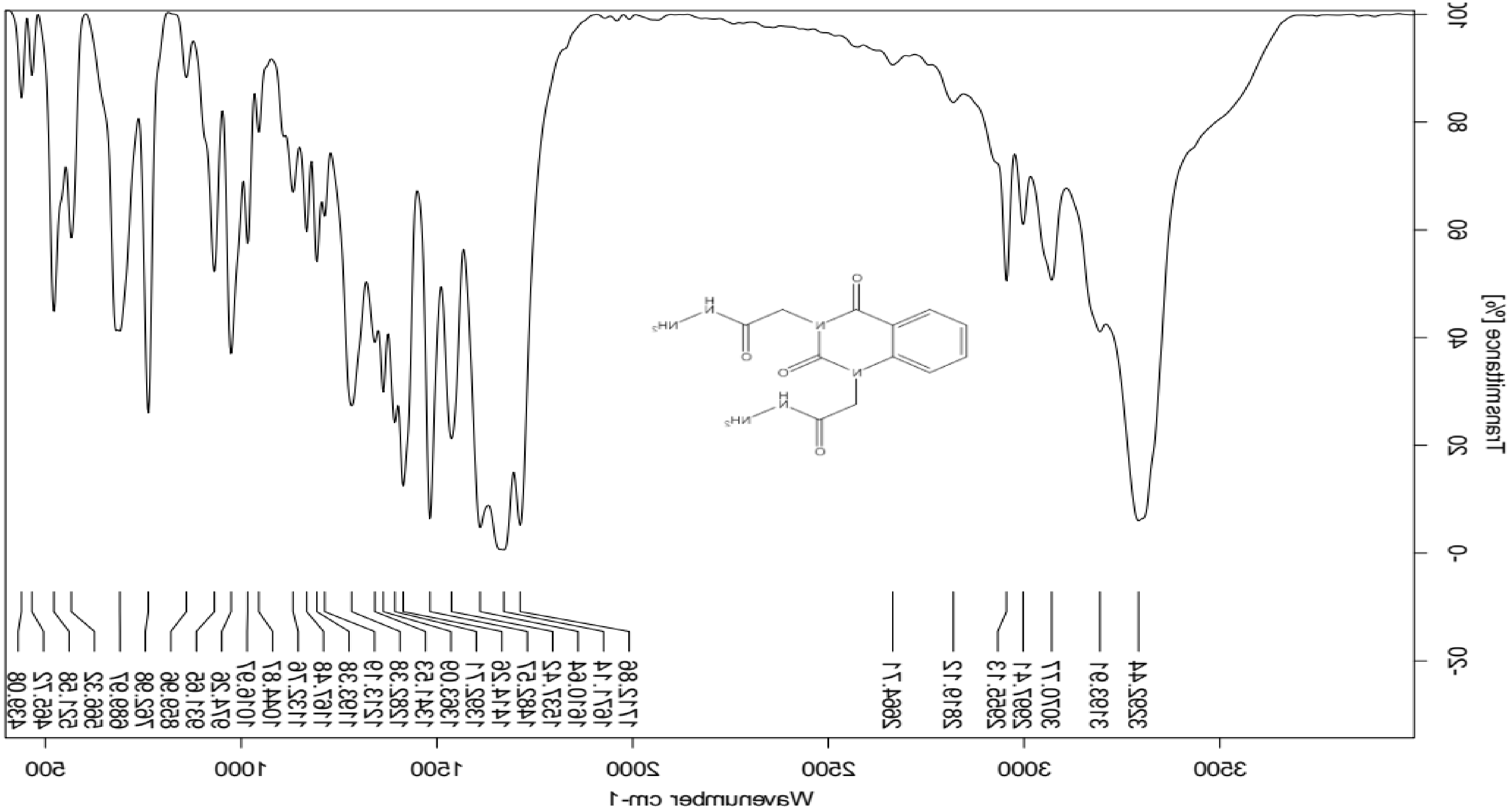

FT-IR of compound (2)

-

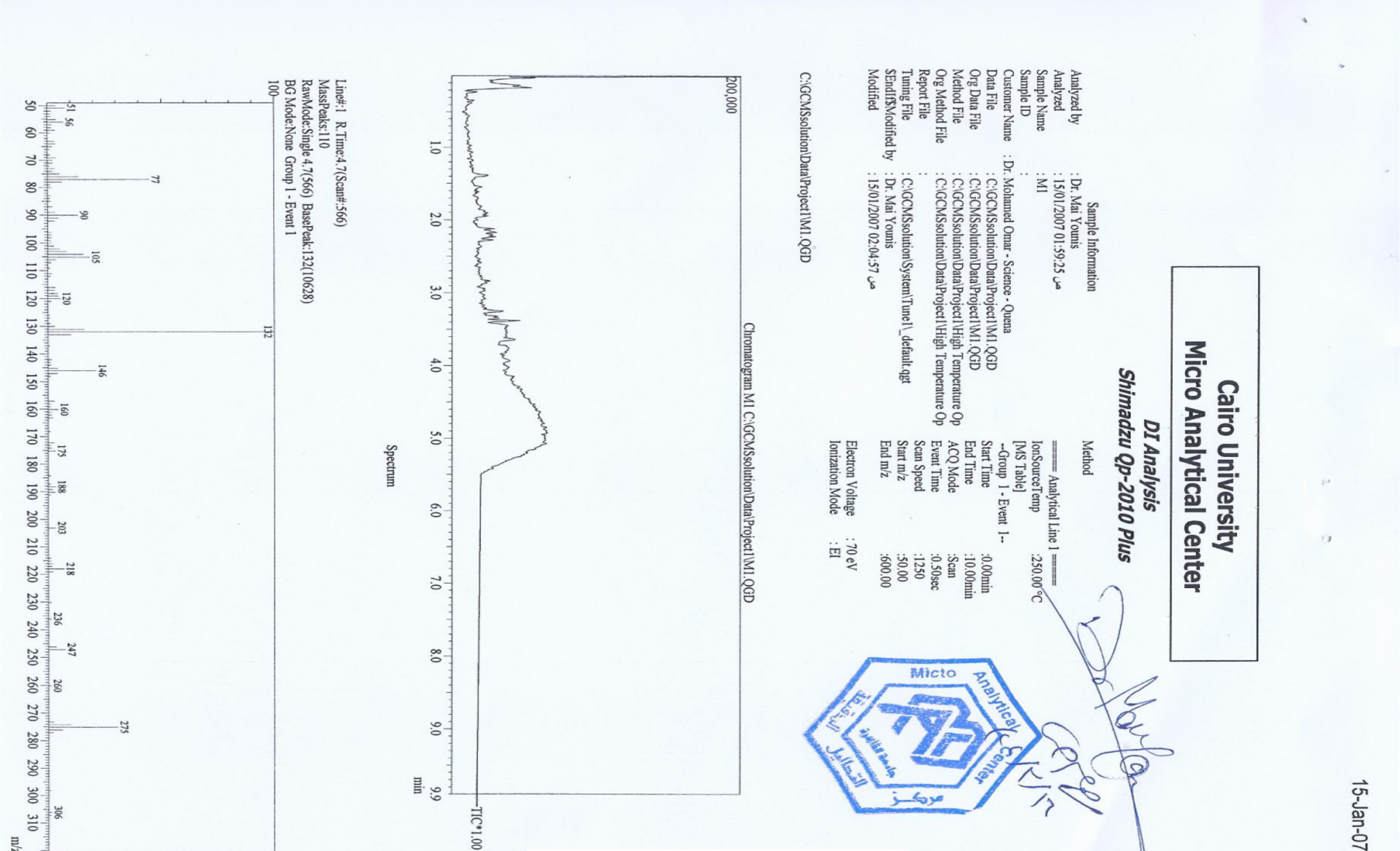

MS of compound (2)

-

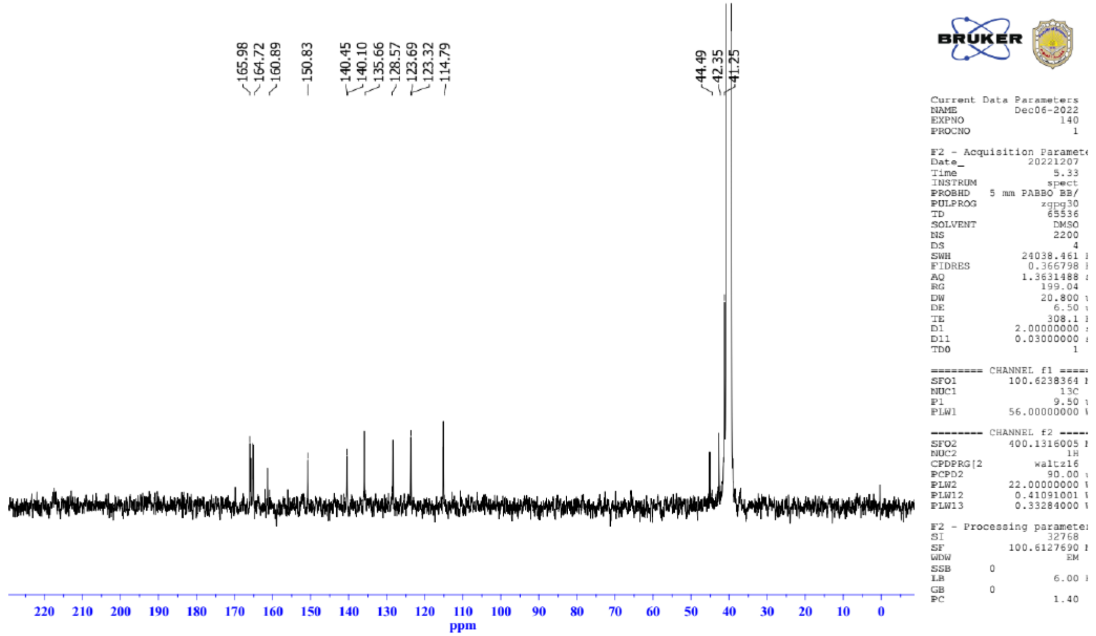

^{13}C-NMR (100 MHz, DMSO) of compound (2)

-

-Compound (3a)

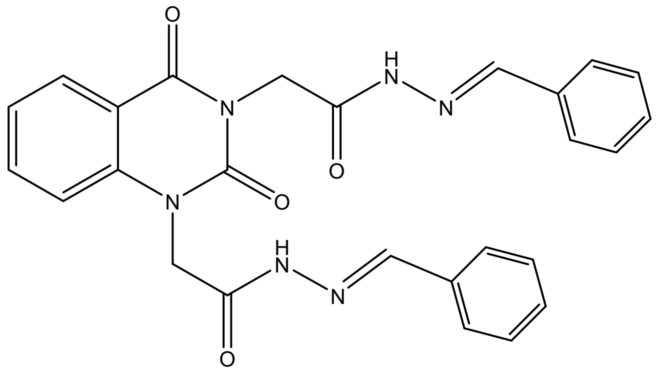

3a

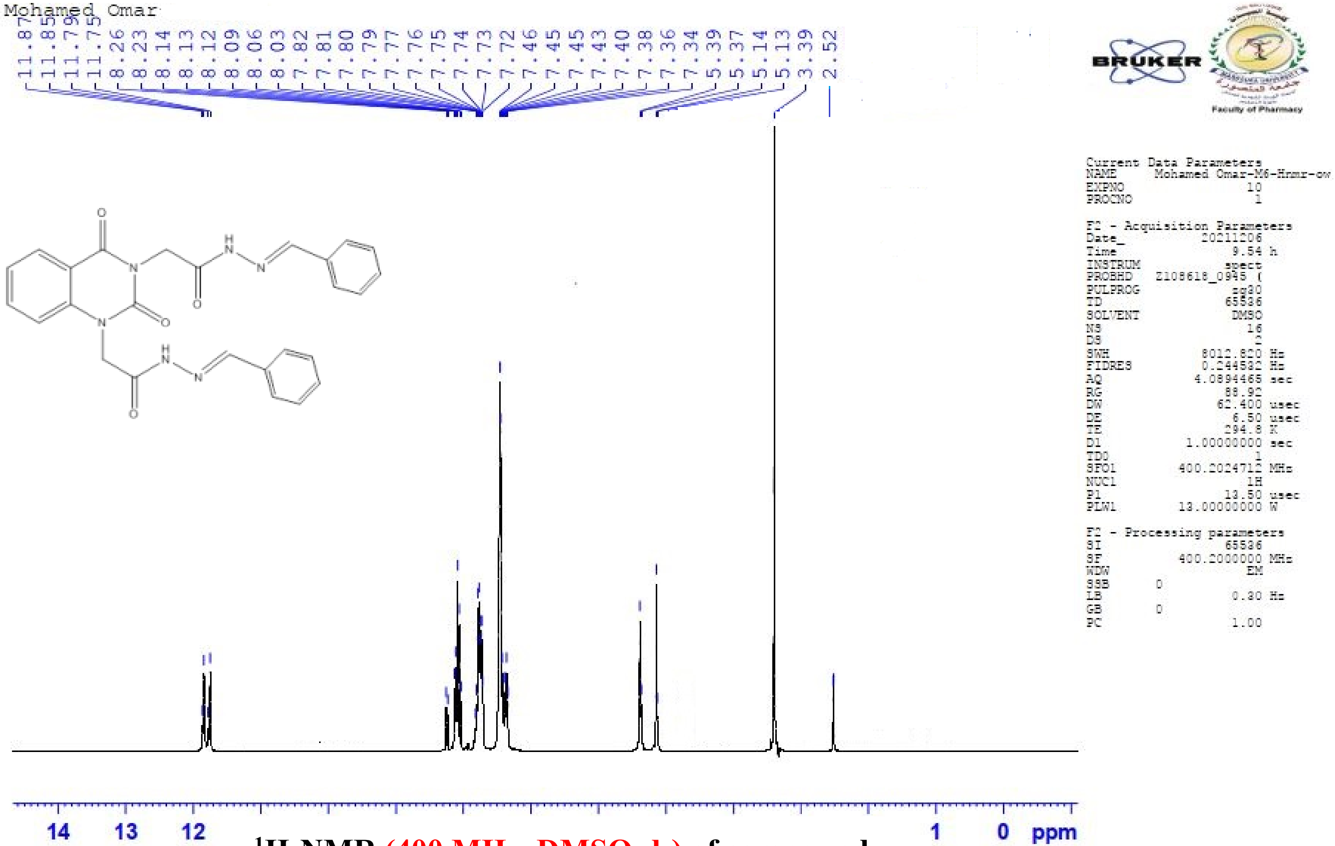

^{1}H-NMR (400 MHz, DMSO-d$_6$) of compound

-

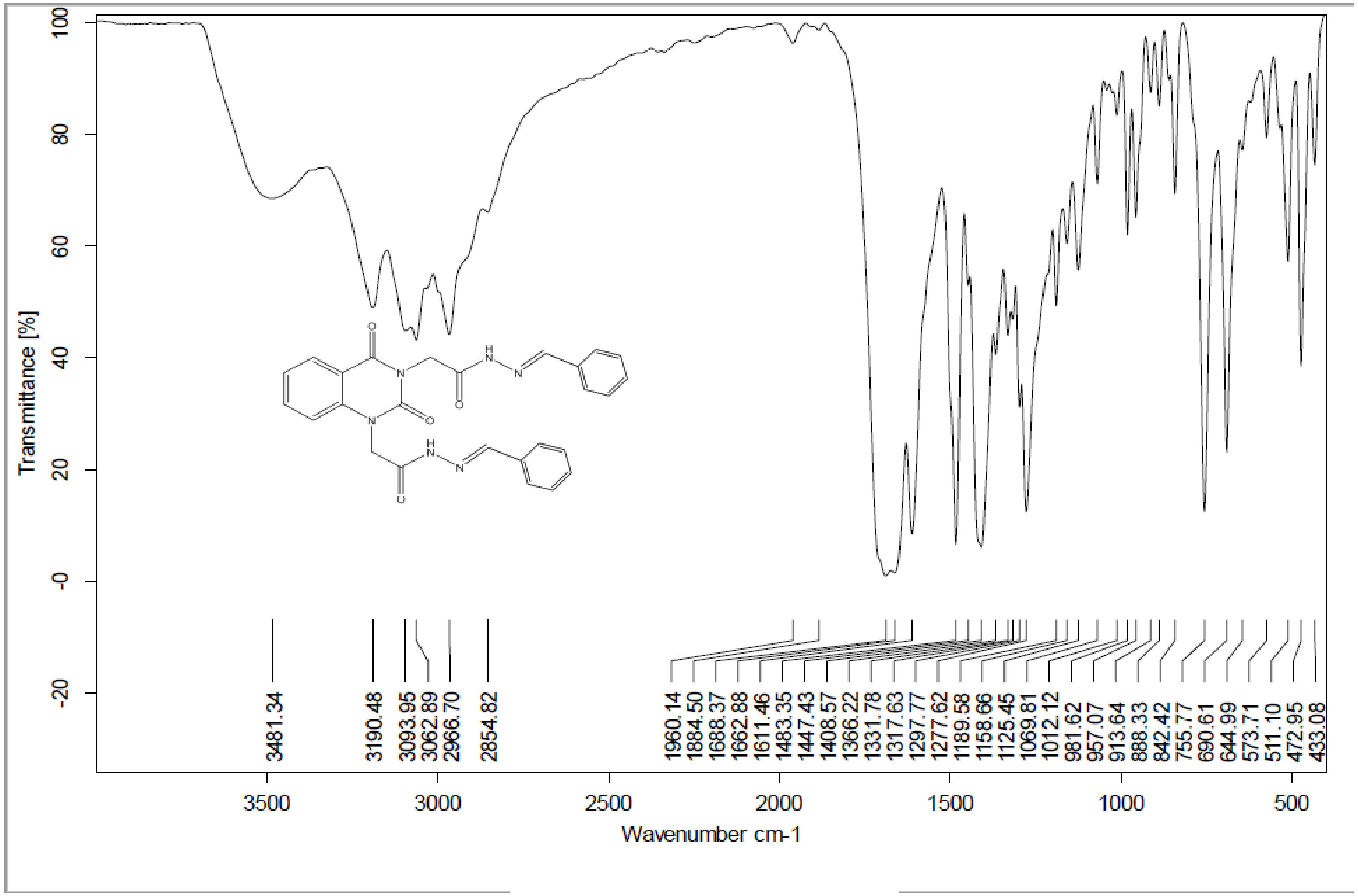

FT-IR of compound (3a)

-

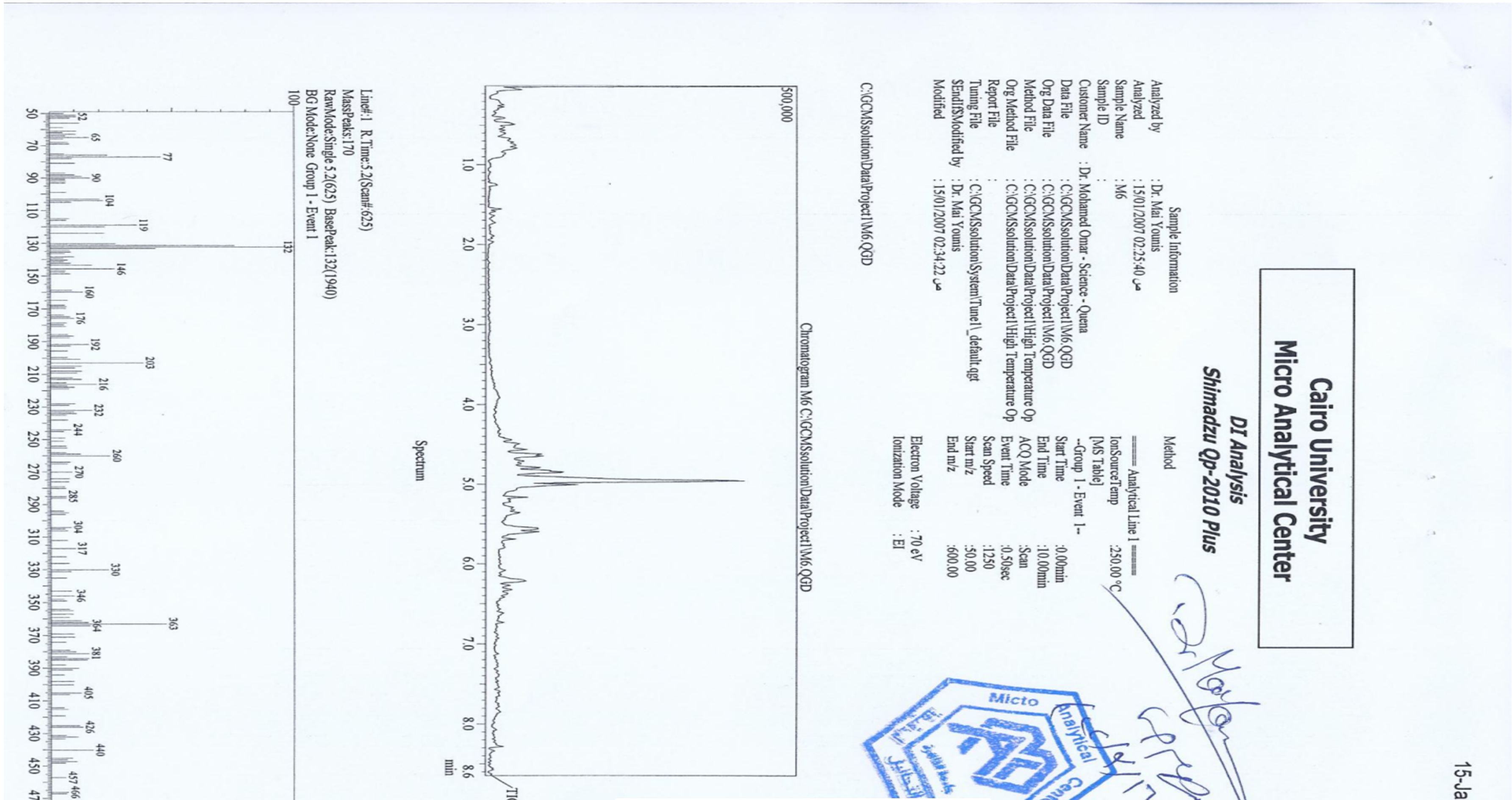

MS of compound (3a)

-

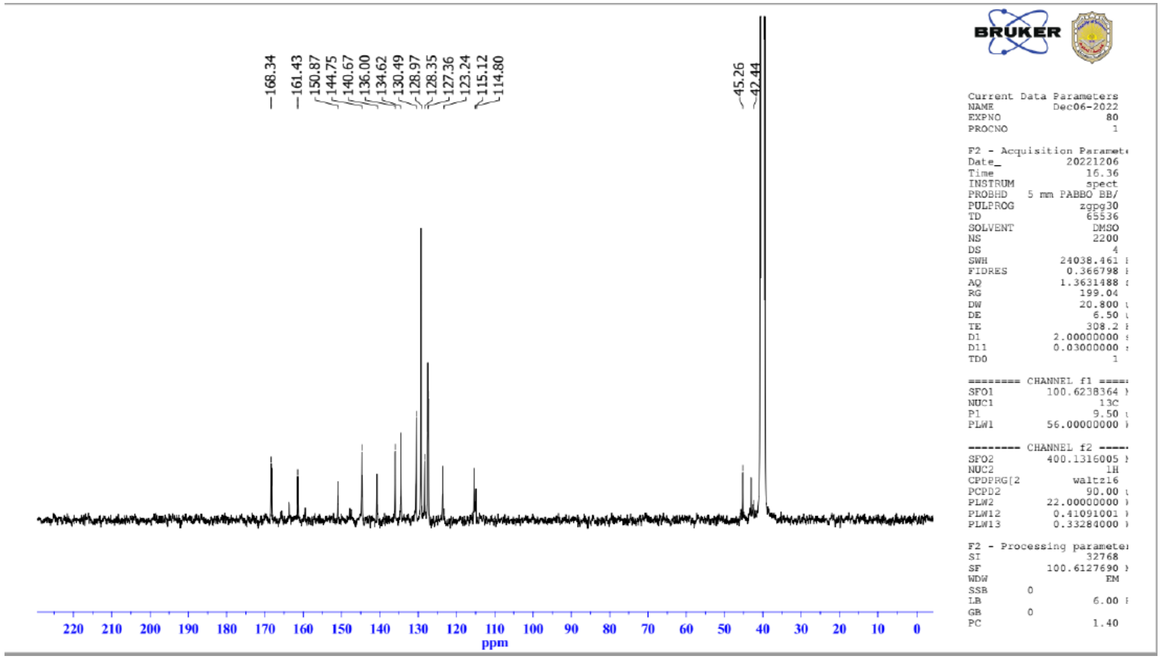

^{13}C-NMR (100 MHz, DMSO) of compound (3a)

-

Compound (3b): -

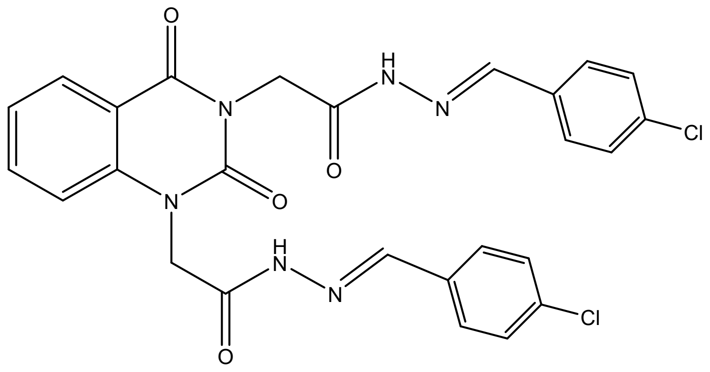

3b

-

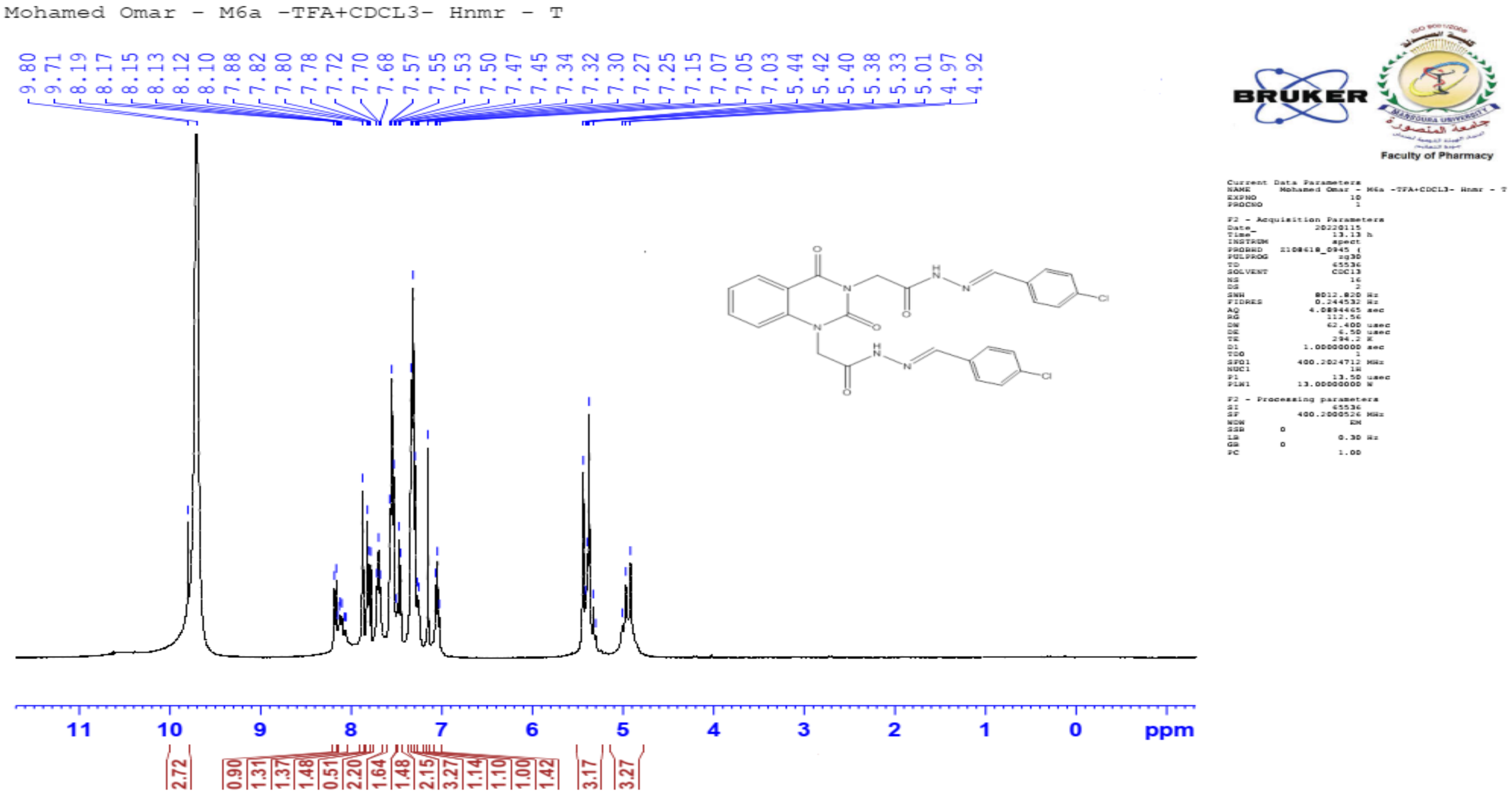

^{1}H-NMR (400 MHz, TFA) of compound (3b)

-

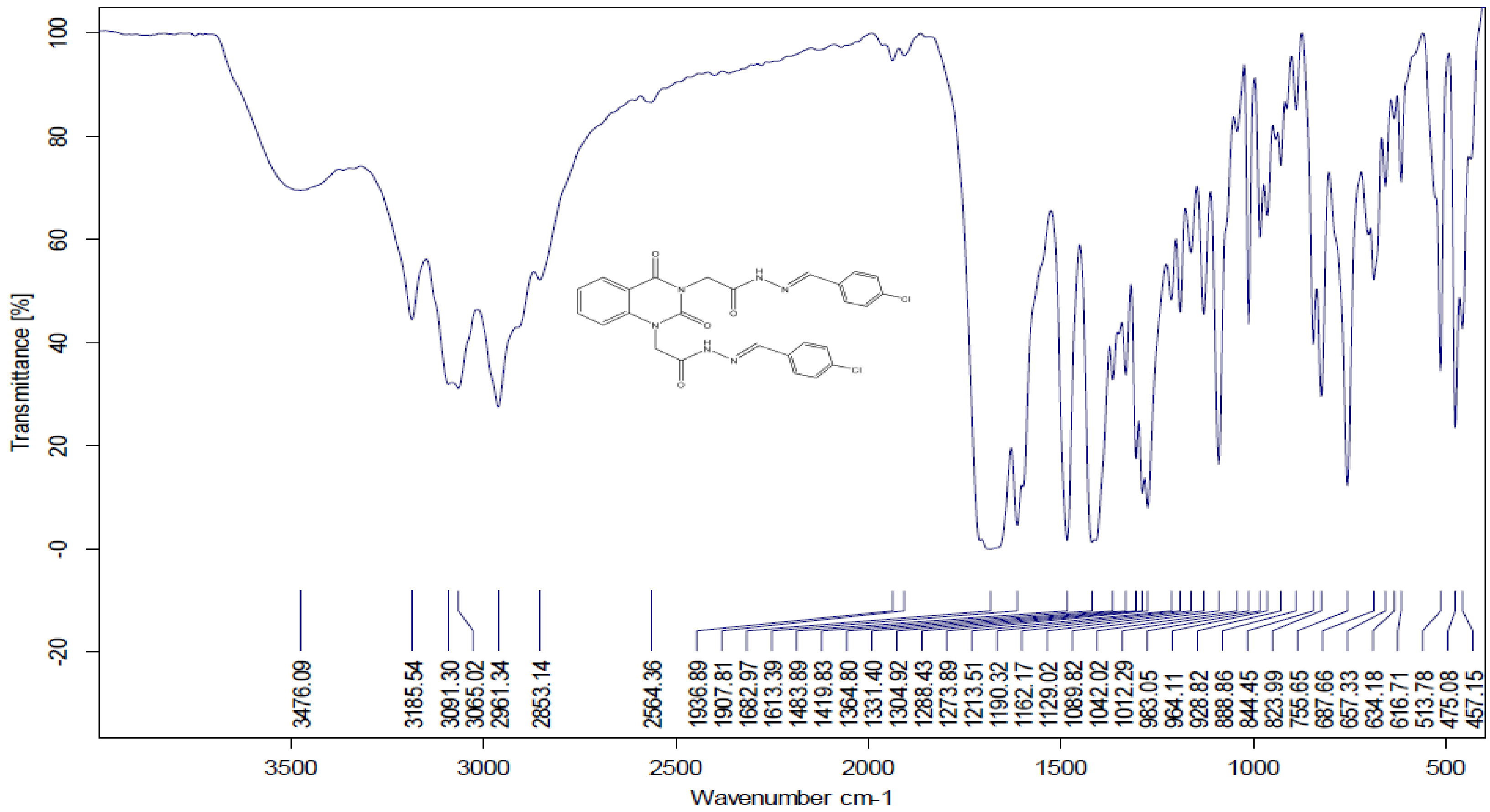

FT-IR of compound (3b)

15-Jan-07 04:21:51

Cairo University
Micro Analytical Center

DI Analysis
Shimadzu Qp-2010 Plus

Sample Information

Analyzed by	: Dr. Mai Younis
Analyzed	: 15/01/2007 04:07:21 ص
Sample Name	: 6a
Sample ID	:
Customer Name	: Dr. Mohamed Omar - Science - Quena
Data File	: C:\GCMSsolution\Data\Project1\6a.QGD
Org Data File	: C:\GCMSsolution\Data\Project1\6a.QGD
Method File	: C:\GCMSsolution\Data\Project1\High Temperature Op
Org Method File	: C:\GCMSsolution\Data\Project1\High Temperature Op
Report File	:
Tuning File	: C:\GCMSsolution\System\Tune1_default.qgt
$EndIf$Modified by	: Dr. Mai Younis
Modified	: 15/01/2007 04:14:59 ص

Method

===== Analytical Line 1 =====

IonSourceTemp	:250.00 °C
[MS Table]	
--Group 1 - Event 1--	
Start Time	:0.00min
End Time	:10.00min
ACQ Mode	:Scan
Event Time	:0.50sec
Scan Speed	:2000
Start m/z	:50.00
End m/z	:900.00
Electron Voltage	: 70 eV
Ionization Mode	: EI

C:\GCMSsolution\Data\Project1\6a.QGD

Chromatogram 6a C:\GCMSsolution\Data\Project1\6a.QGD

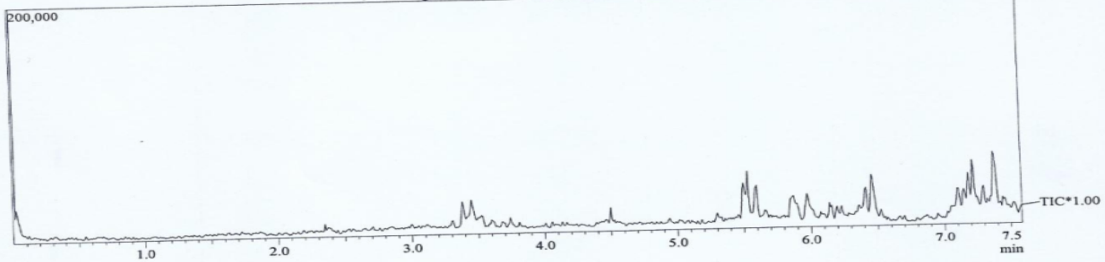

Spectrum

Line#:1 R.Time:6.0(Scan#:715)
MassPeaks:33
RawMode:Single 6.0(715) BasePeak:132(466)
BG Mode:None Group 1 - Event 1

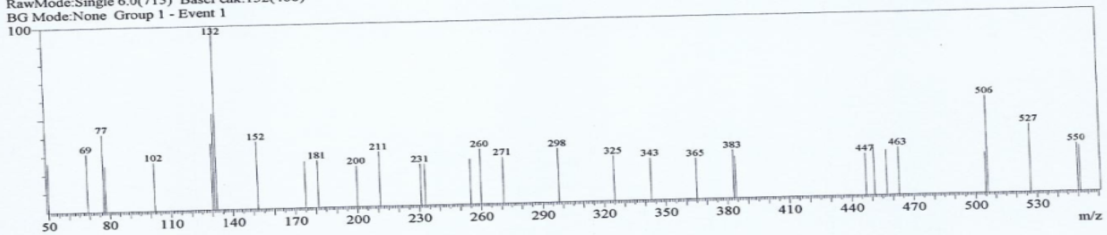

MS of compound (3b)

-

-Compound (3c):

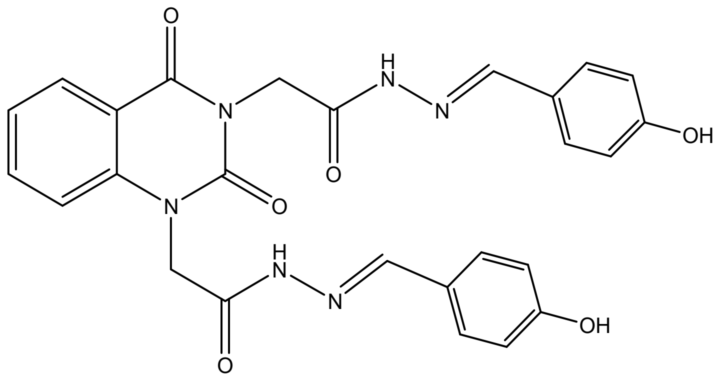

3c

-

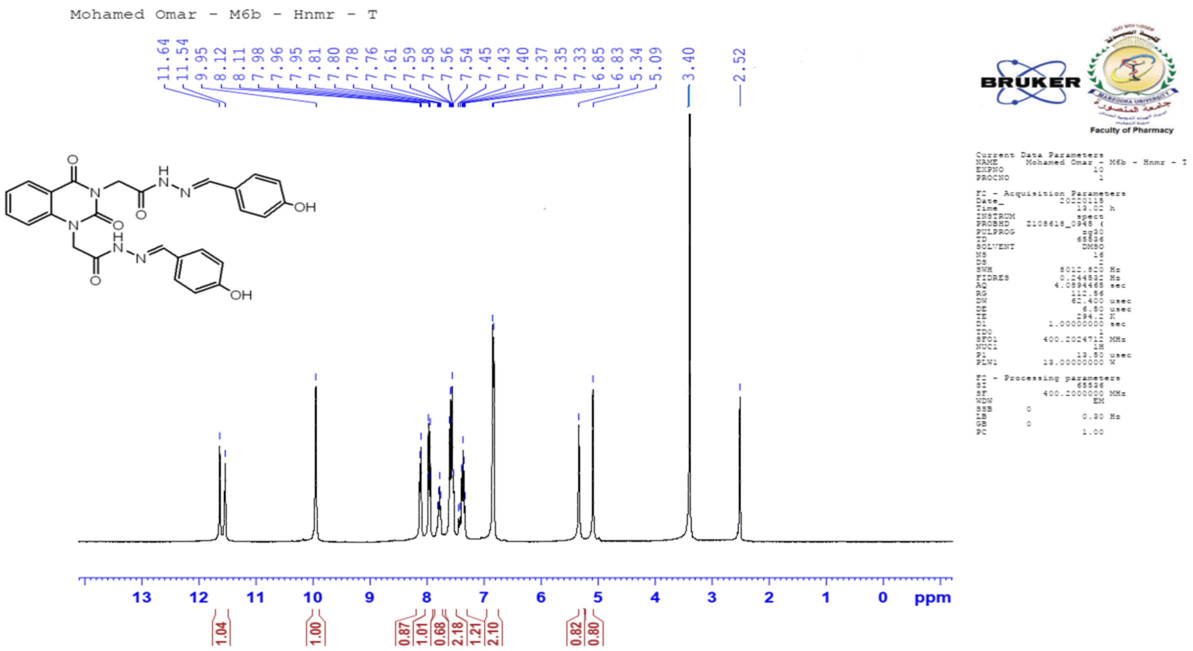

^{1}H-NMR (400 MHz, DMSO-d_6) of compound (3c)

-

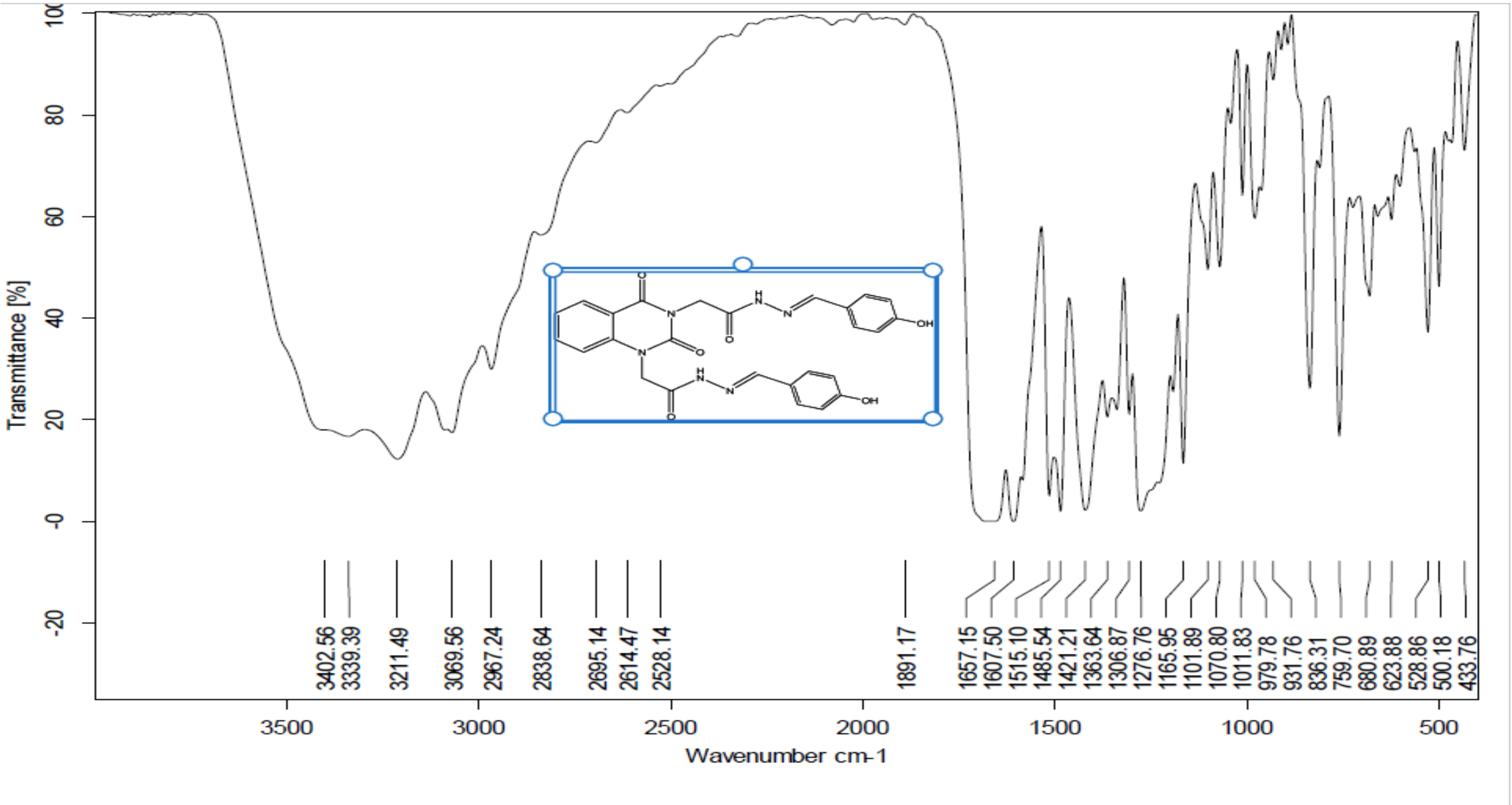

FT-IR of compound (3c)

-

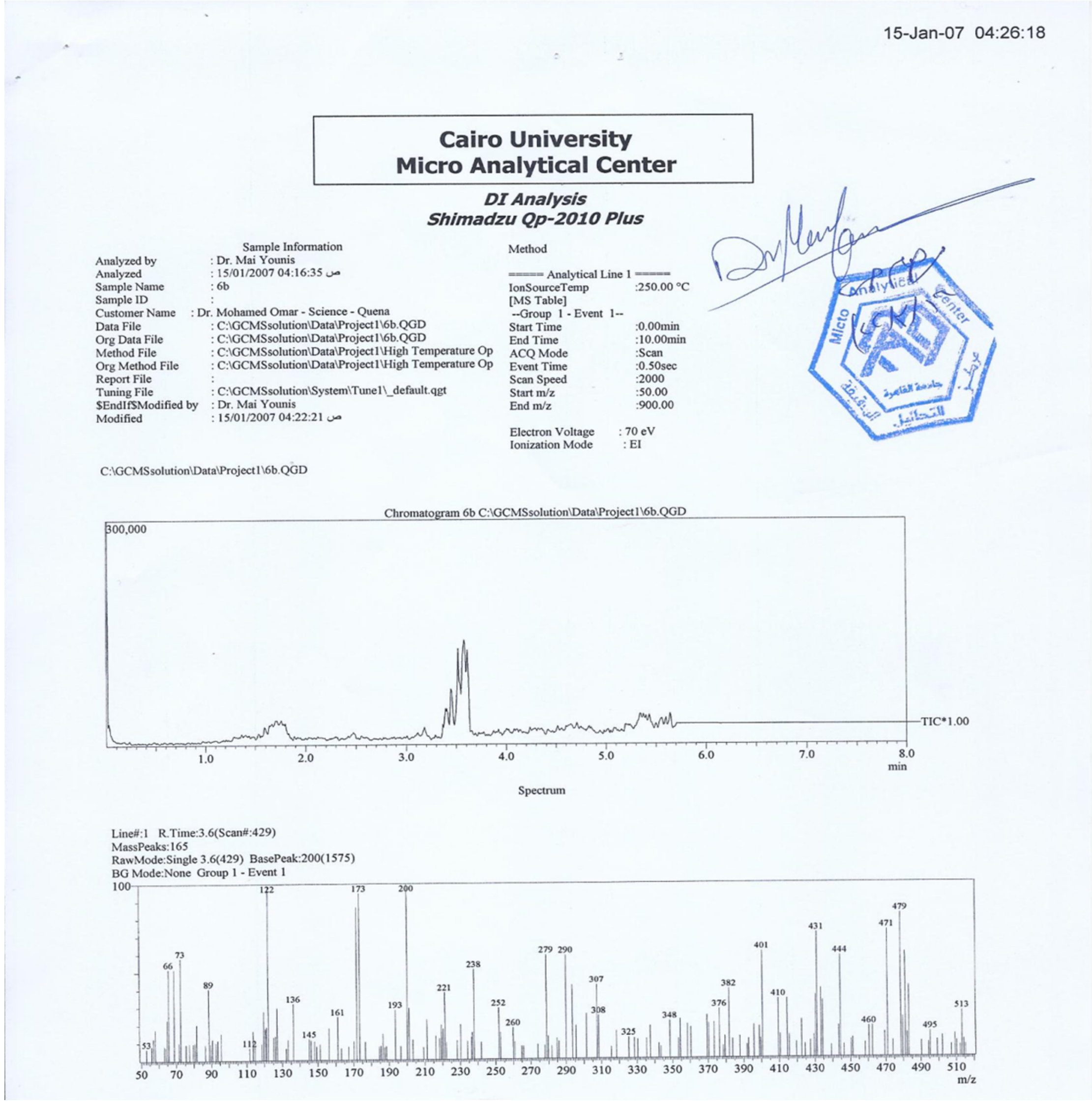
15-Jan-07 04:26:18

Cairo University
Micro Analytical Center

DI Analysis
Shimadzu Qp-2010 Plus

Sample Information

Analyzed by	: Dr. Mai Younis
Analyzed	: 15/01/2007 04:16:35 ص
Sample Name	: 6b
Sample ID	:
Customer Name	: Dr. Mohamed Omar - Science - Quena
Data File	: C:\GCMSsolution\Data\Project1\6b.QGD
Org Data File	: C:\GCMSsolution\Data\Project1\6b.QGD
Method File	: C:\GCMSsolution\Data\Project1\High Temperature Op
Org Method File	: C:\GCMSsolution\Data\Project1\High Temperature Op
Report File	:
Tuning File	: C:\GCMSsolution\System\Tune1_default.qgt
$EndIf$Modified by	: Dr. Mai Younis
Modified	: 15/01/2007 04:22:21 ص

Method

===== Analytical Line 1 =====

IonSourceTemp	:250.00 °C
[MS Table]	
--Group 1 - Event 1--	
Start Time	:0.00min
End Time	:10.00min
ACQ Mode	:Scan
Event Time	:0.50sec
Scan Speed	:2000
Start m/z	:50.00
End m/z	:900.00
Electron Voltage	: 70 eV
Ionization Mode	: EI

C:\GCMSsolution\Data\Project1\6b.QGD

MS of compound (3c)

-

-Compound (3d):

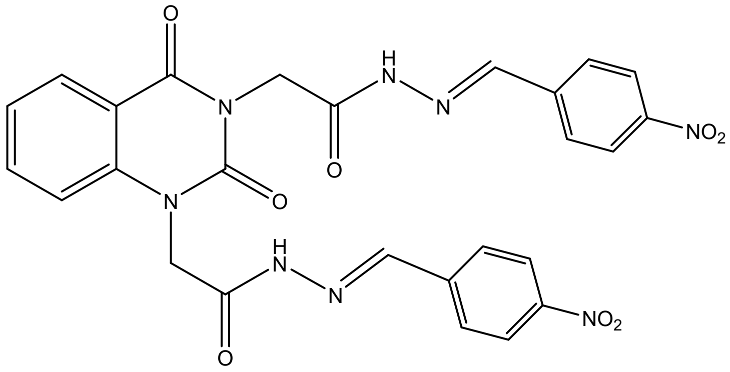

3d

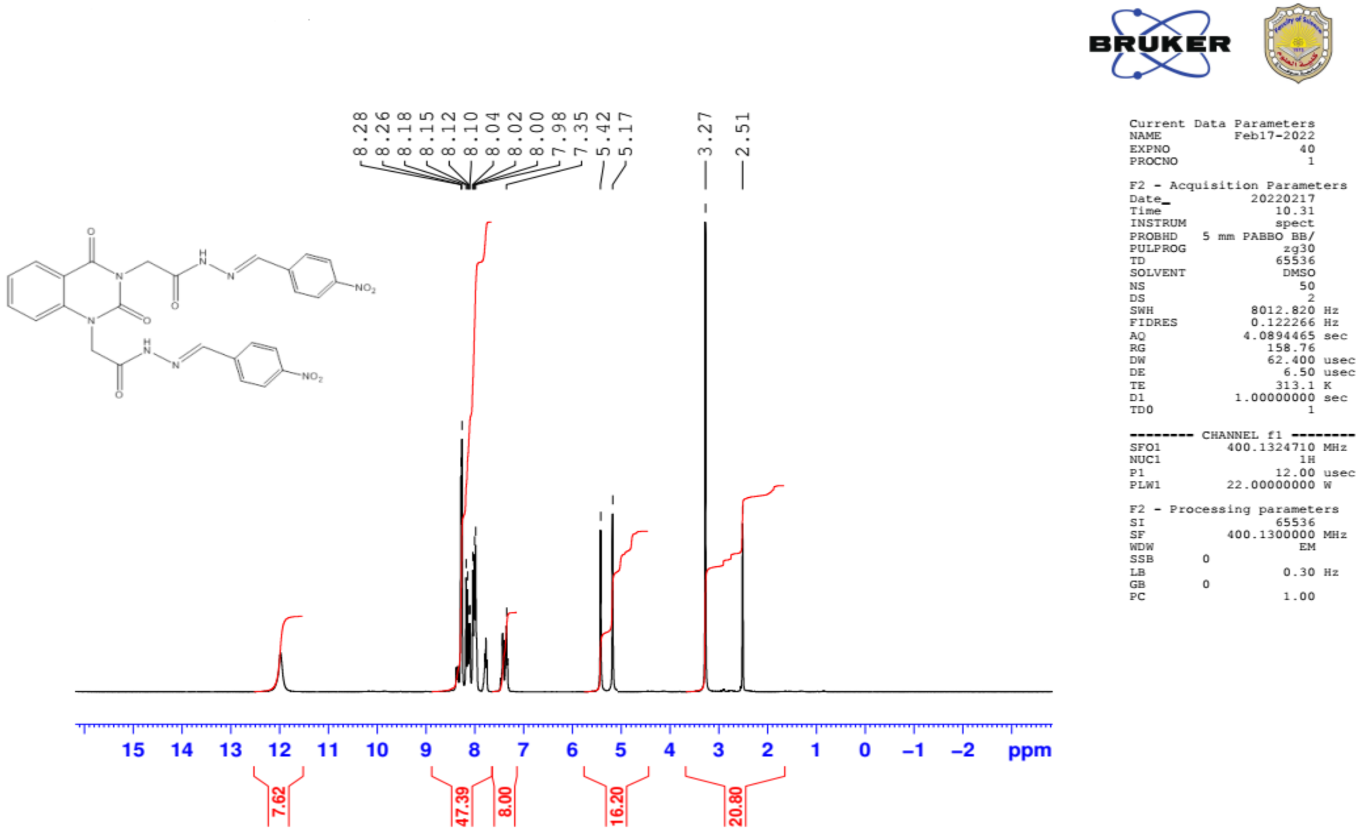

^{1}H-NMR (400 MHz, DMSO-d_6) of compound (3d)

-

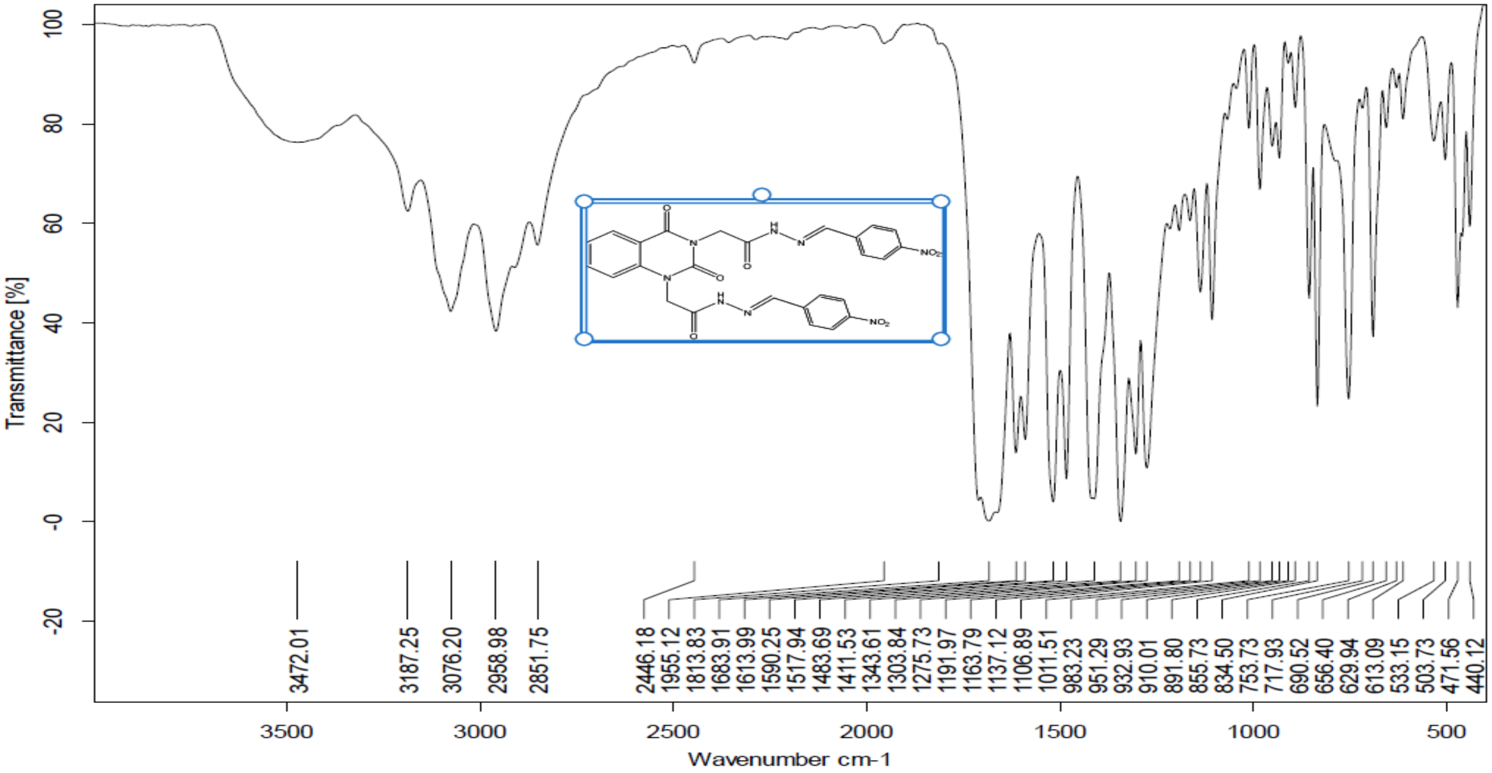

FT-IR of compound (3d)

-

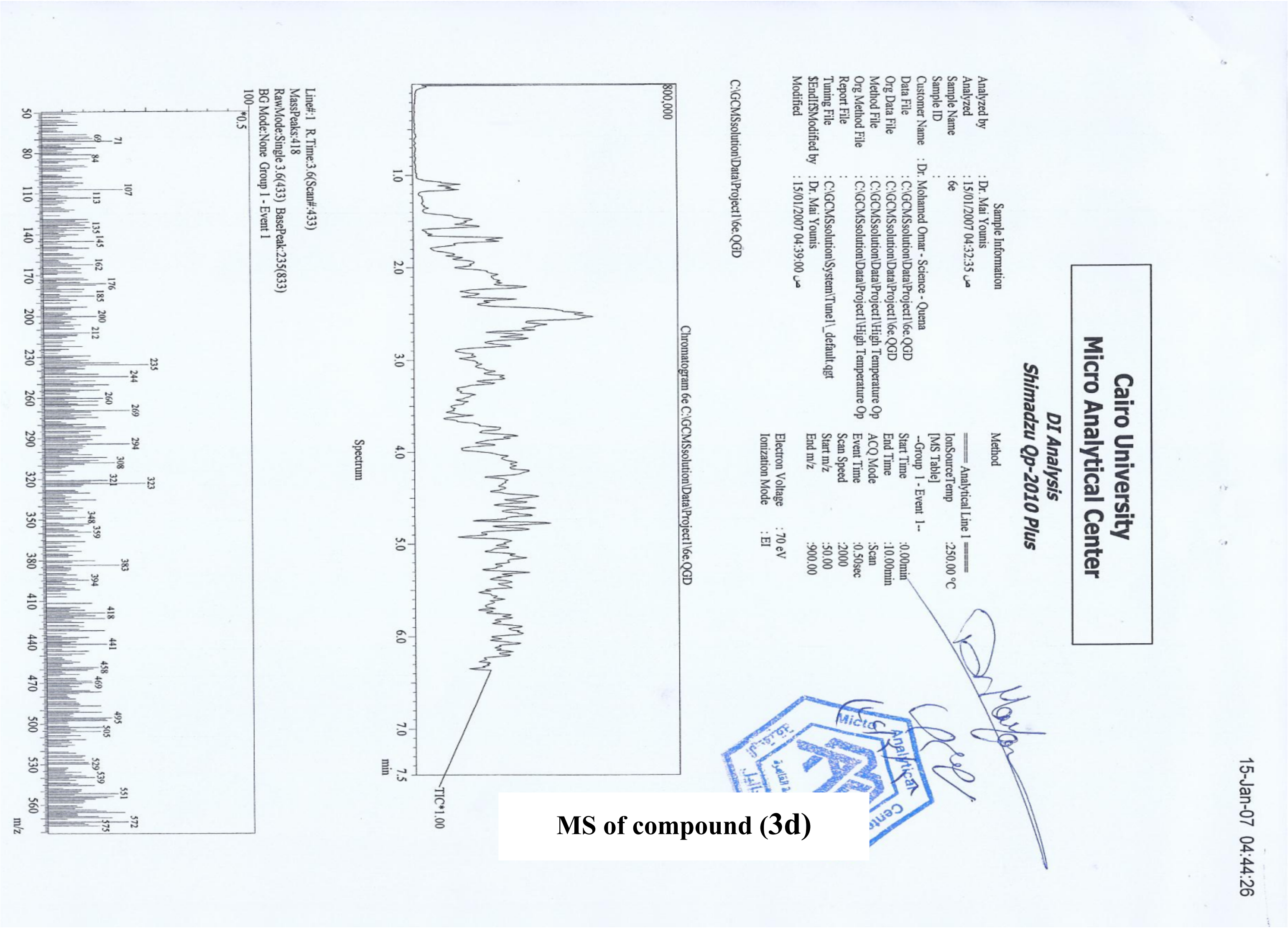

MS of compound (3d)

-

-Compound (3e):

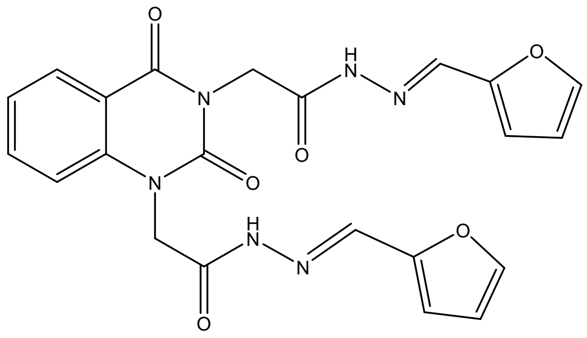

3e

-

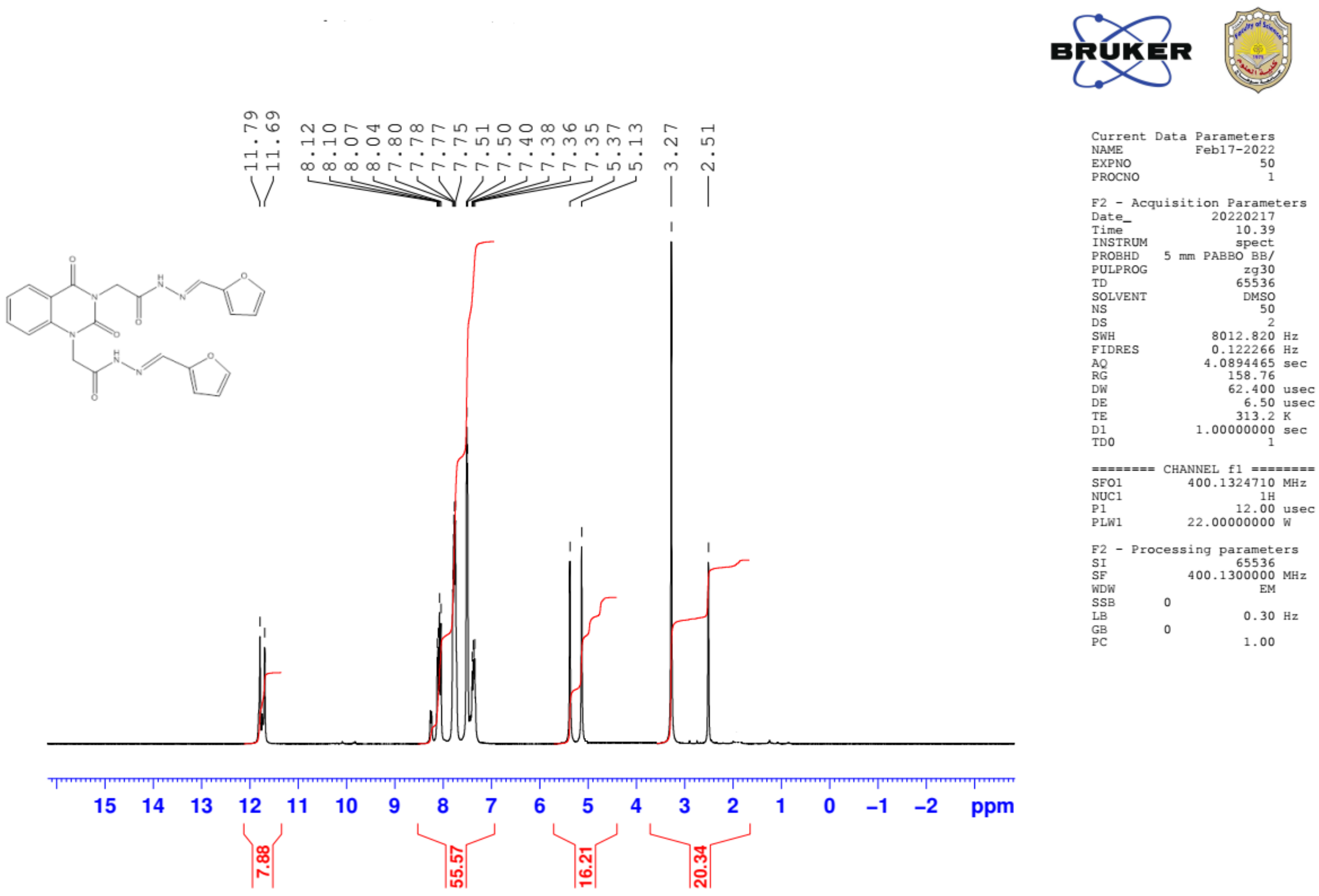

^{1}H-NMR (400 MHz, DMSO-d_6) of compound (3e)

-

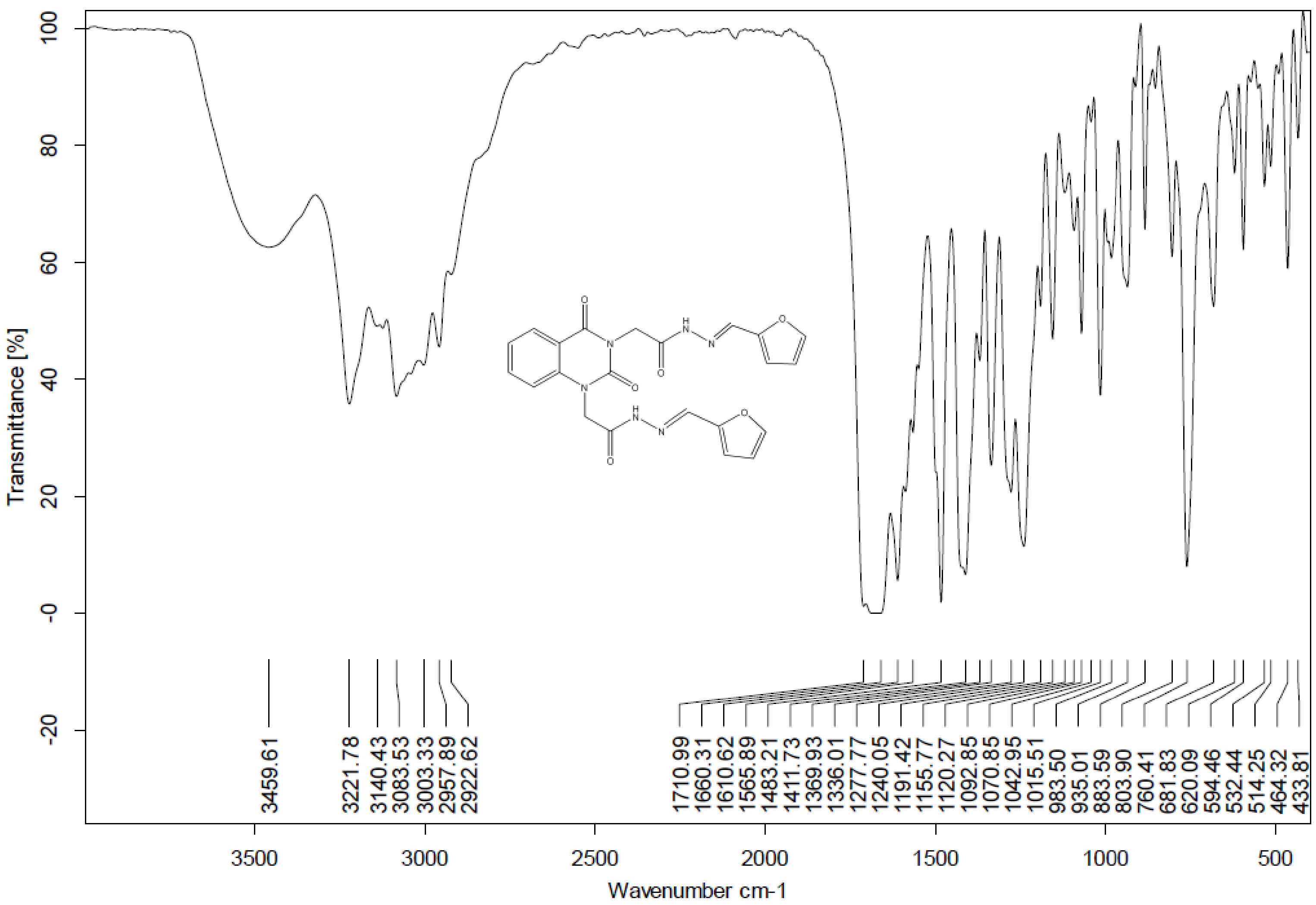

FT-IR of compound (3e)

-

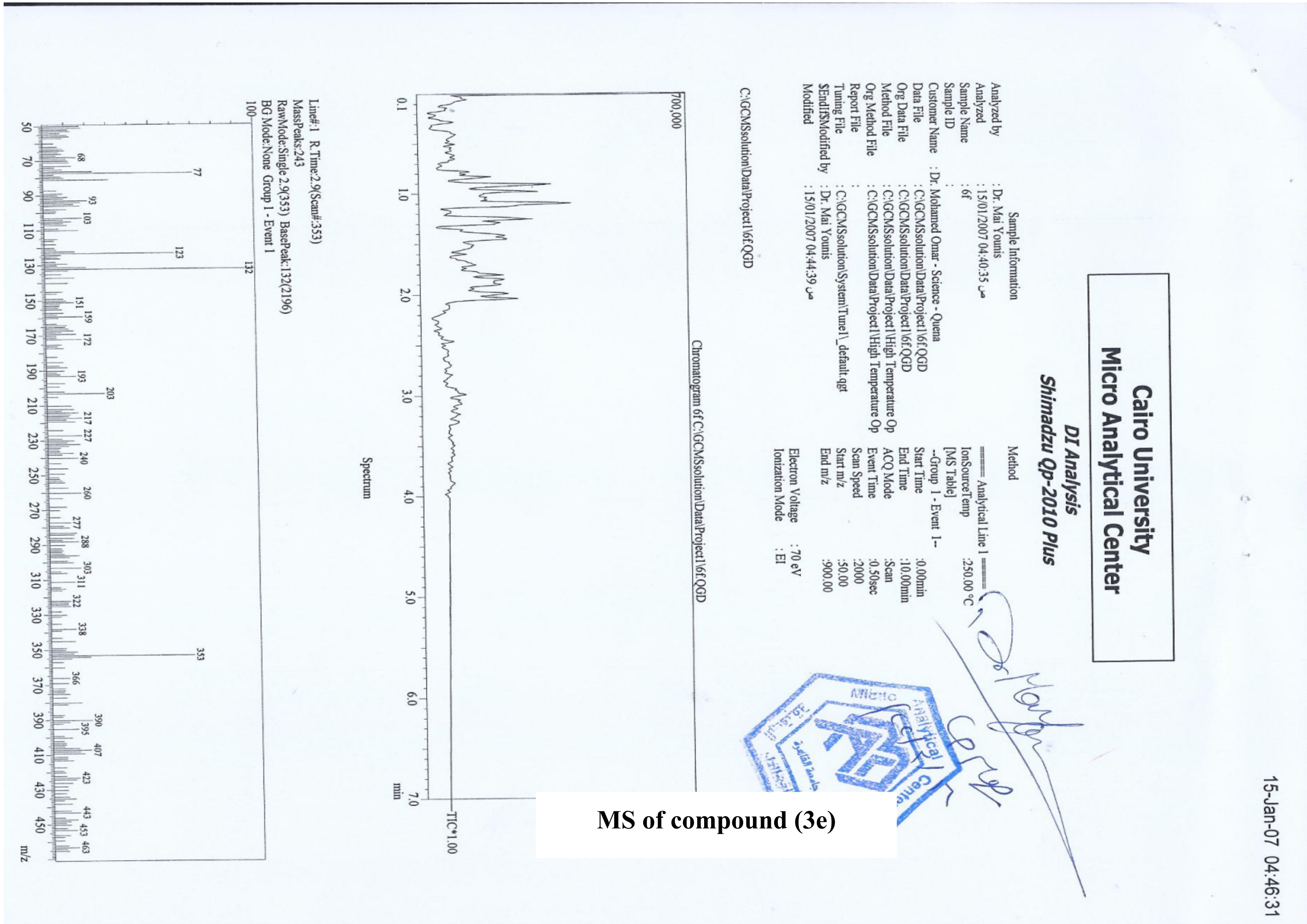

MS of compound (3e)

-

-Compound (4a): -

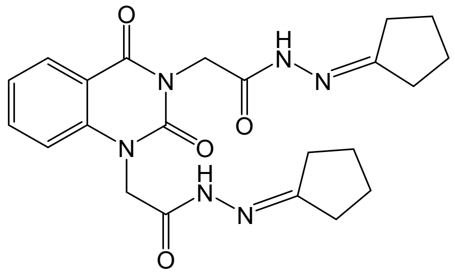

4a

-

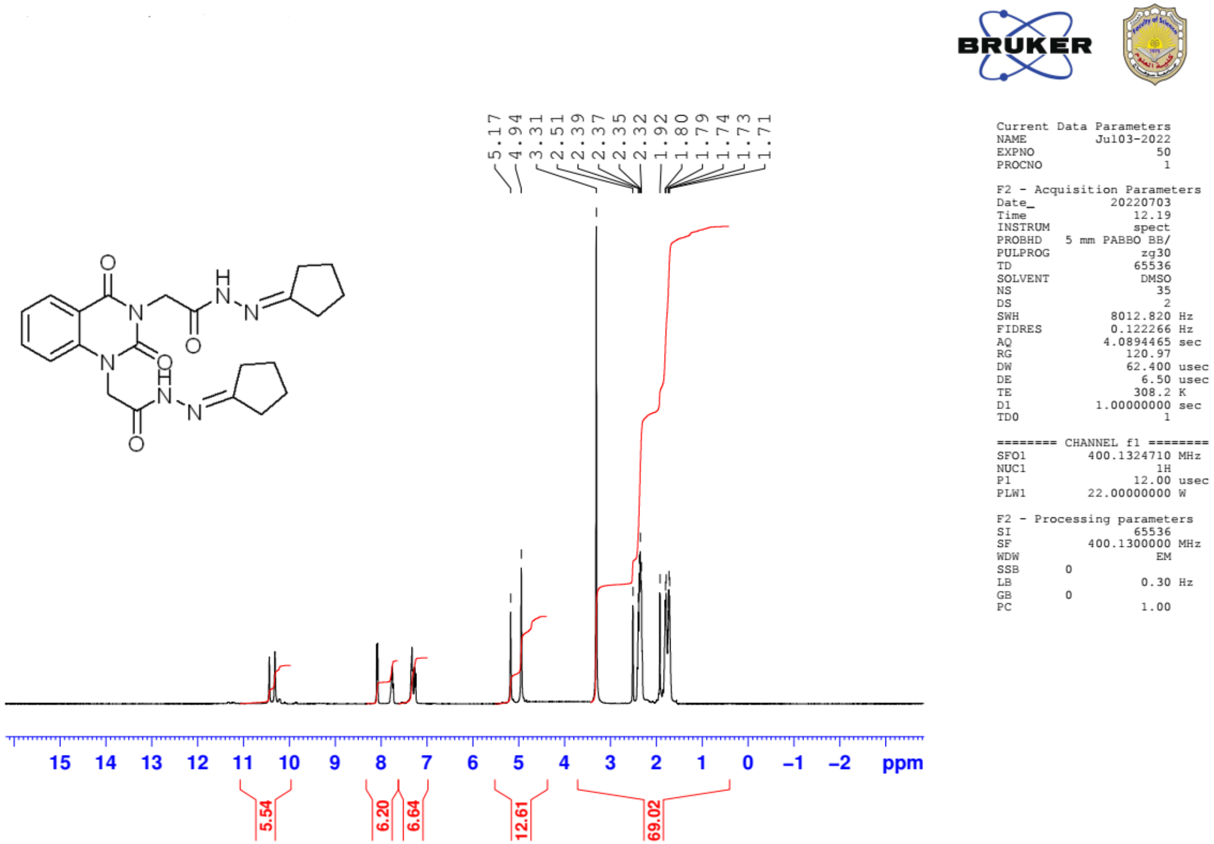

^{1}H-NMR (400 MHz, DMSO-d_6) of compound (4a)

-

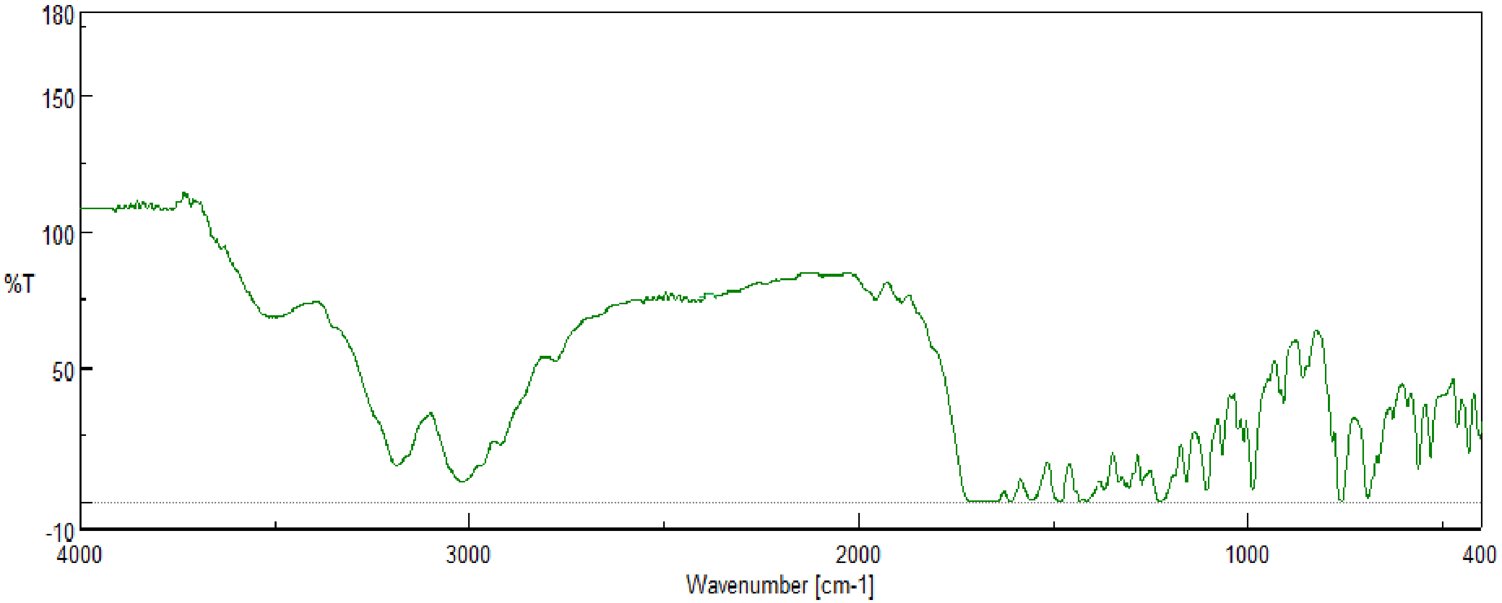

FT-IR of compound (4a)

-

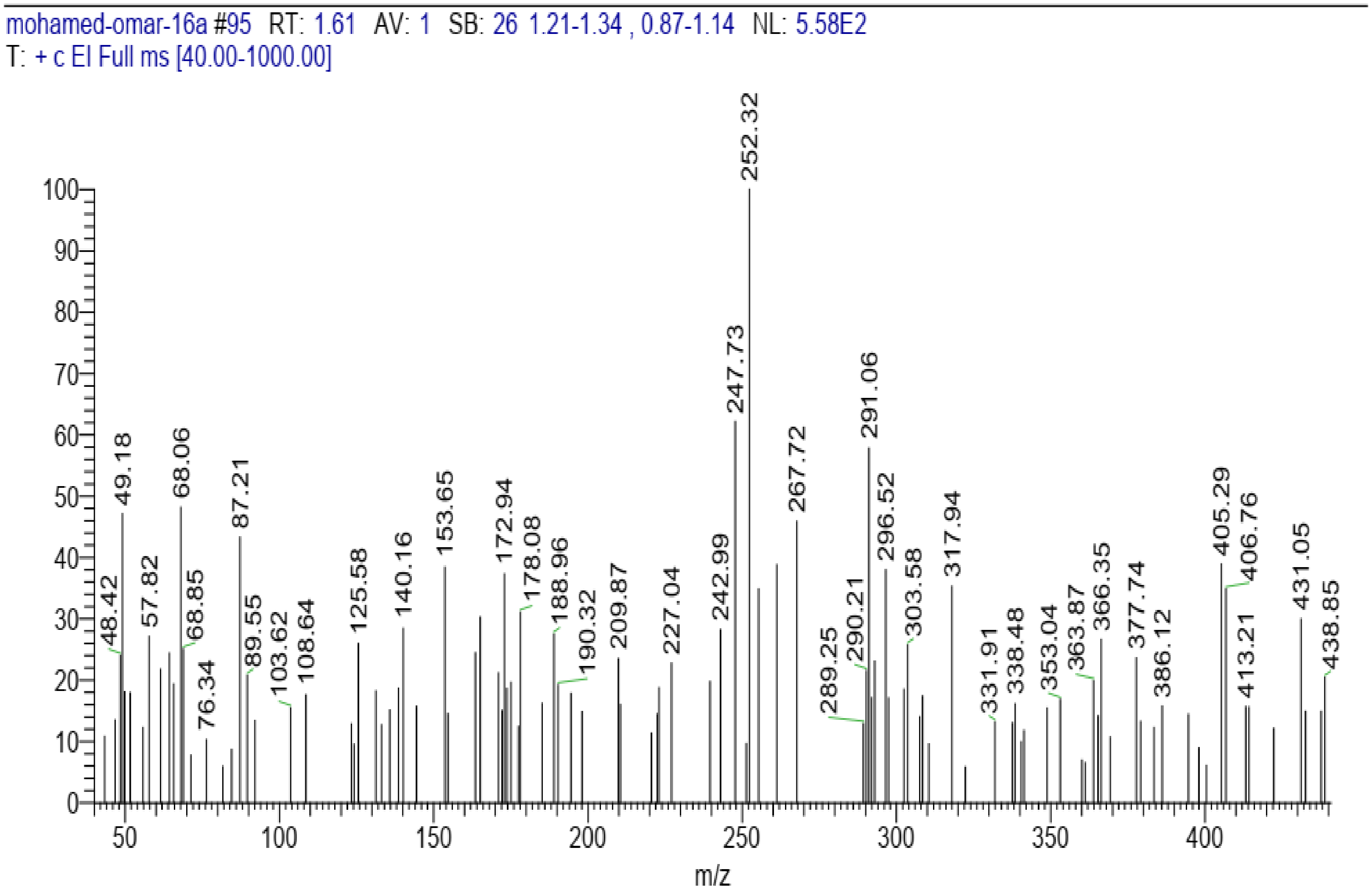

m/z Intensity Relative

MS of compound (4a)

-

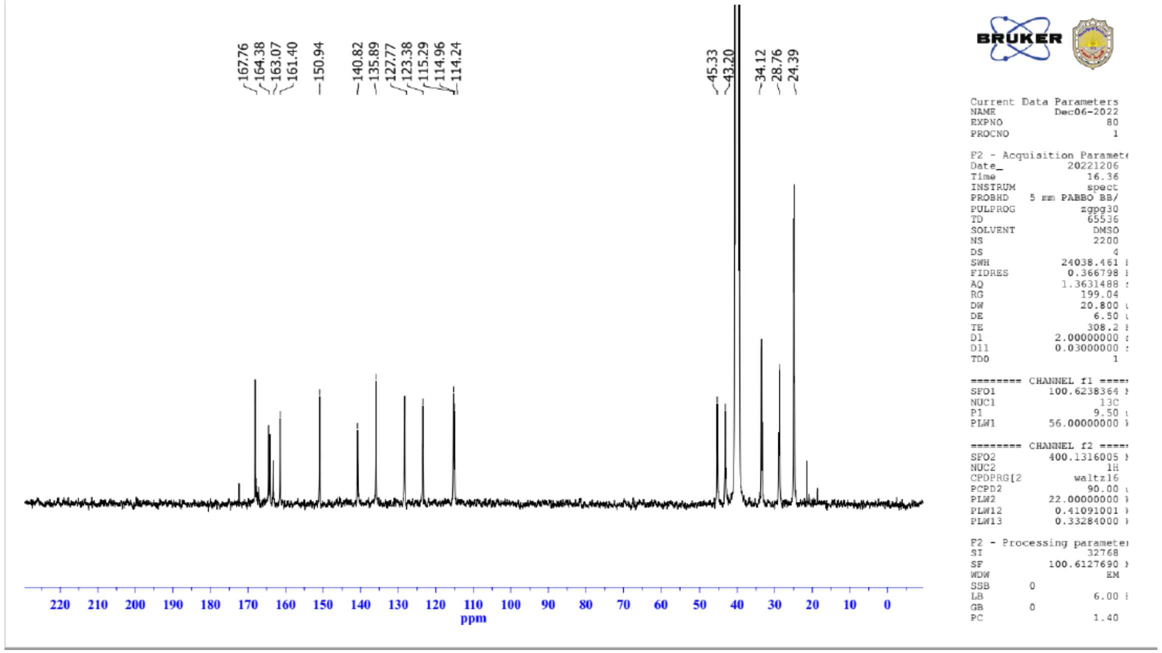

^{13}C-NMR (100 MHz, DMSO) of compound (4a)

-

-Compound (4b): -

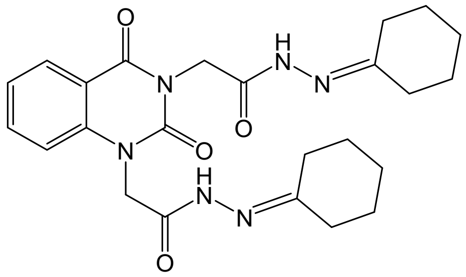

4b

-

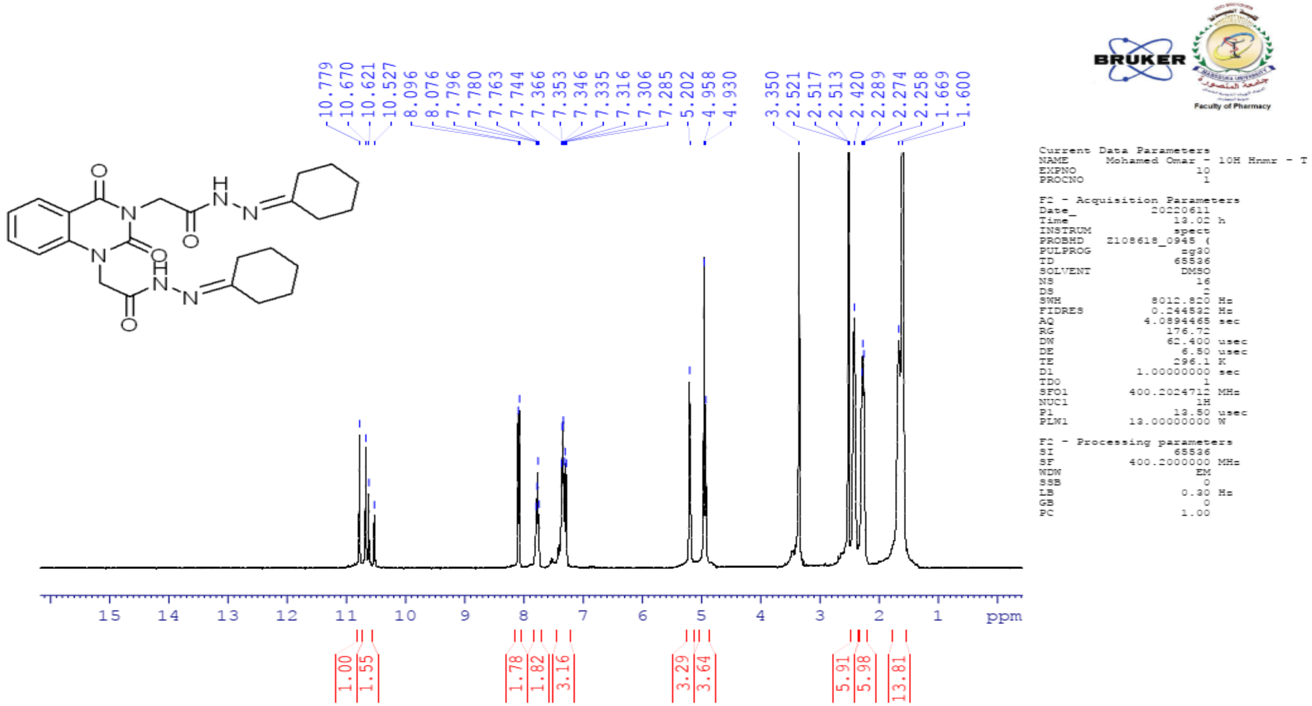

^{1}H-NMR (400 MHz, DMSO-d_6) of compound (4b)

-

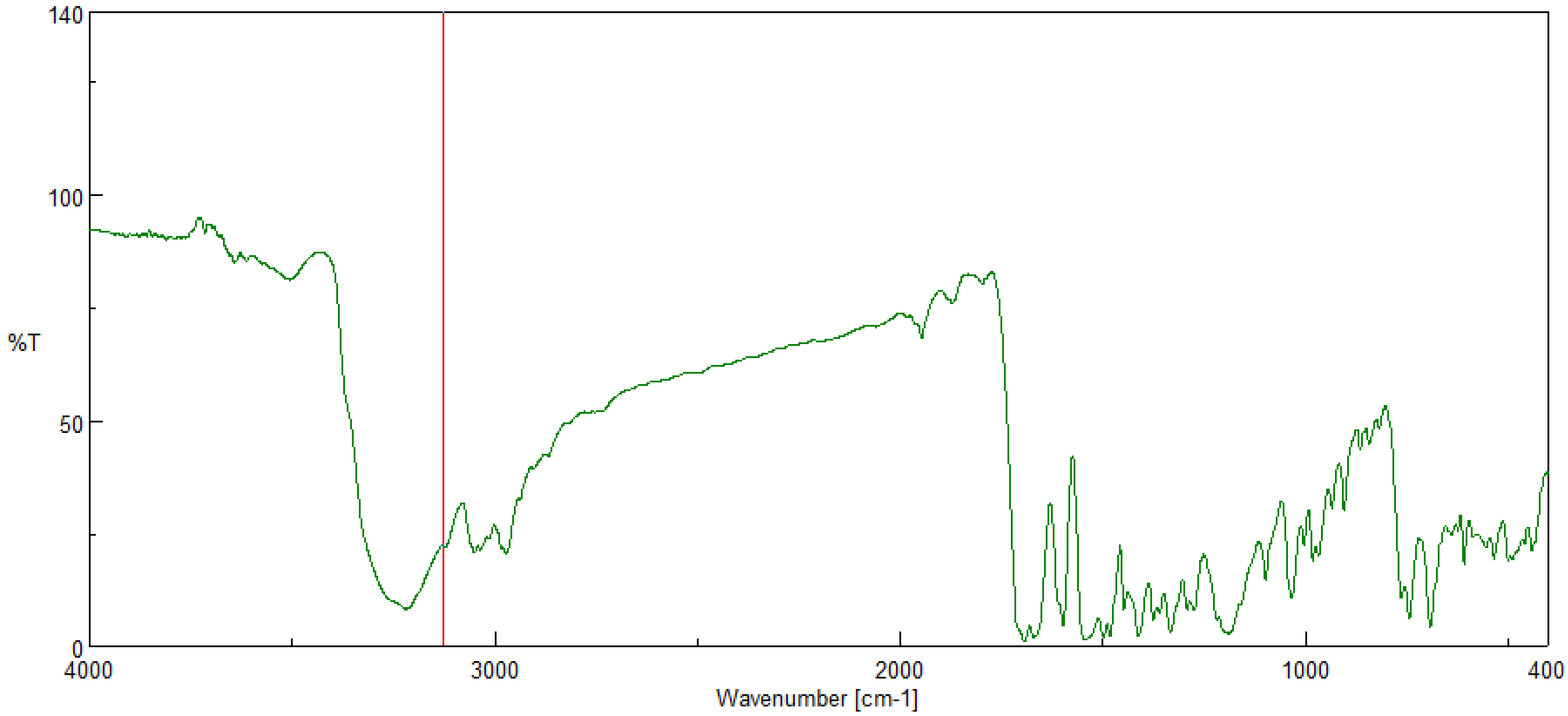

FT-IR of compound (4b)

-

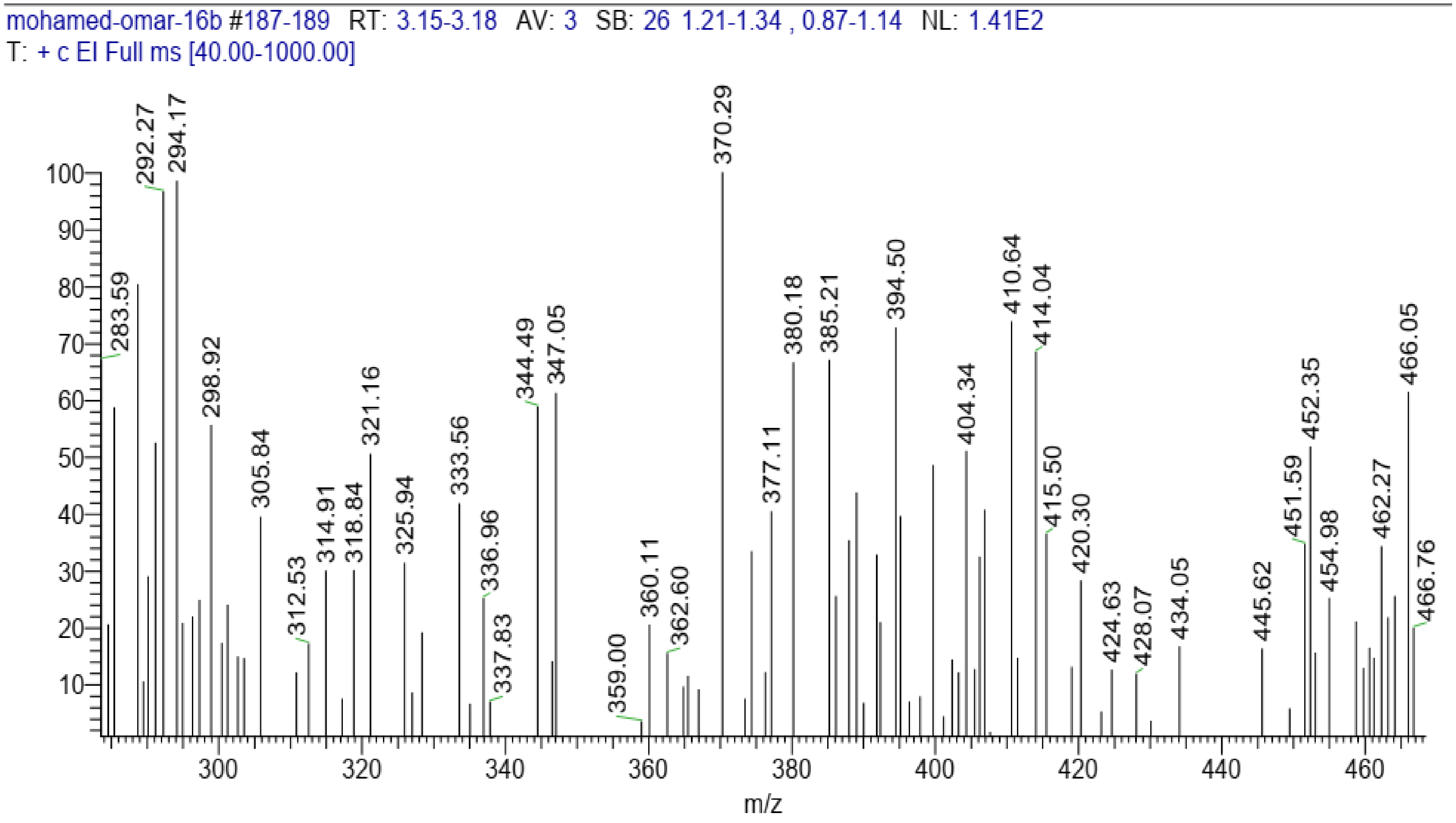

m/z Intensity Relative

MS of compound (4b)

-

- Compound (4c):

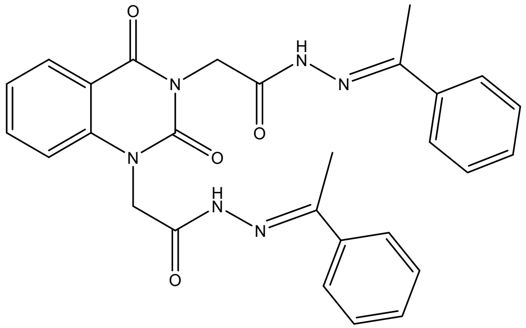

4c

-

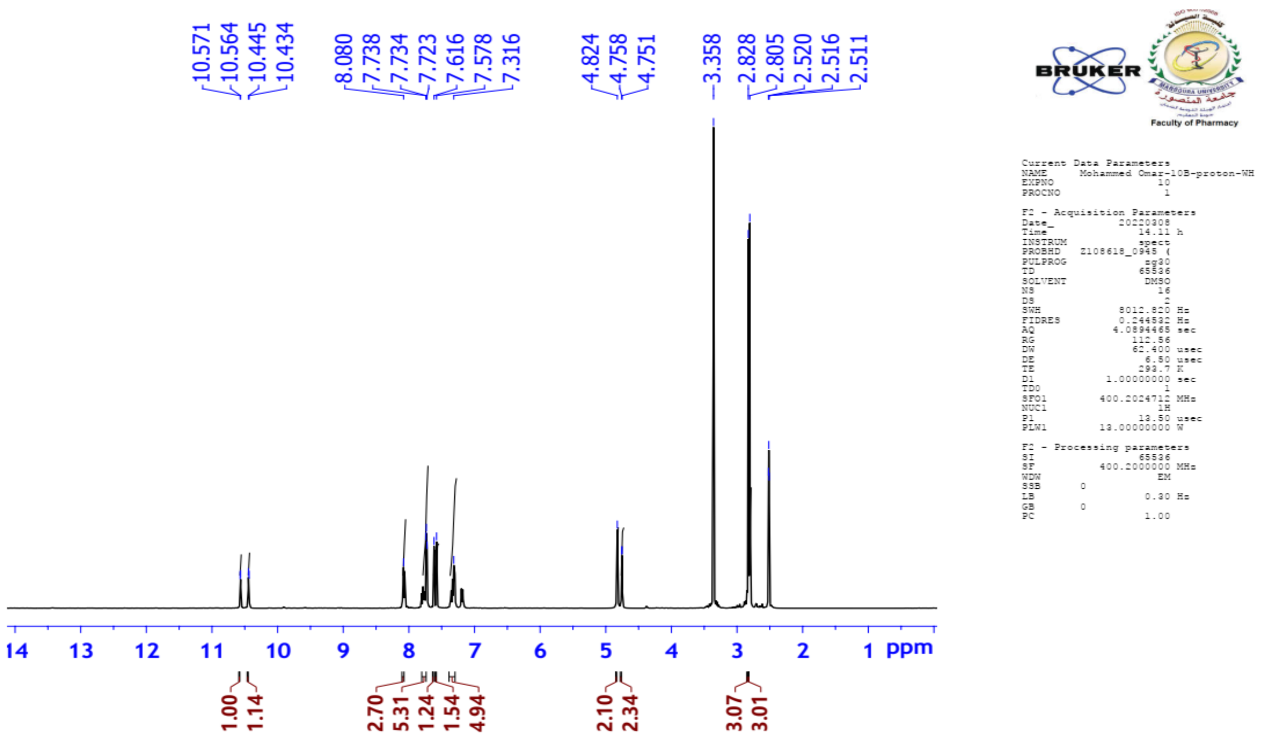

^{1}H-NMR (400 MHz, DMSO-d$_6$) of compound (4c)

-

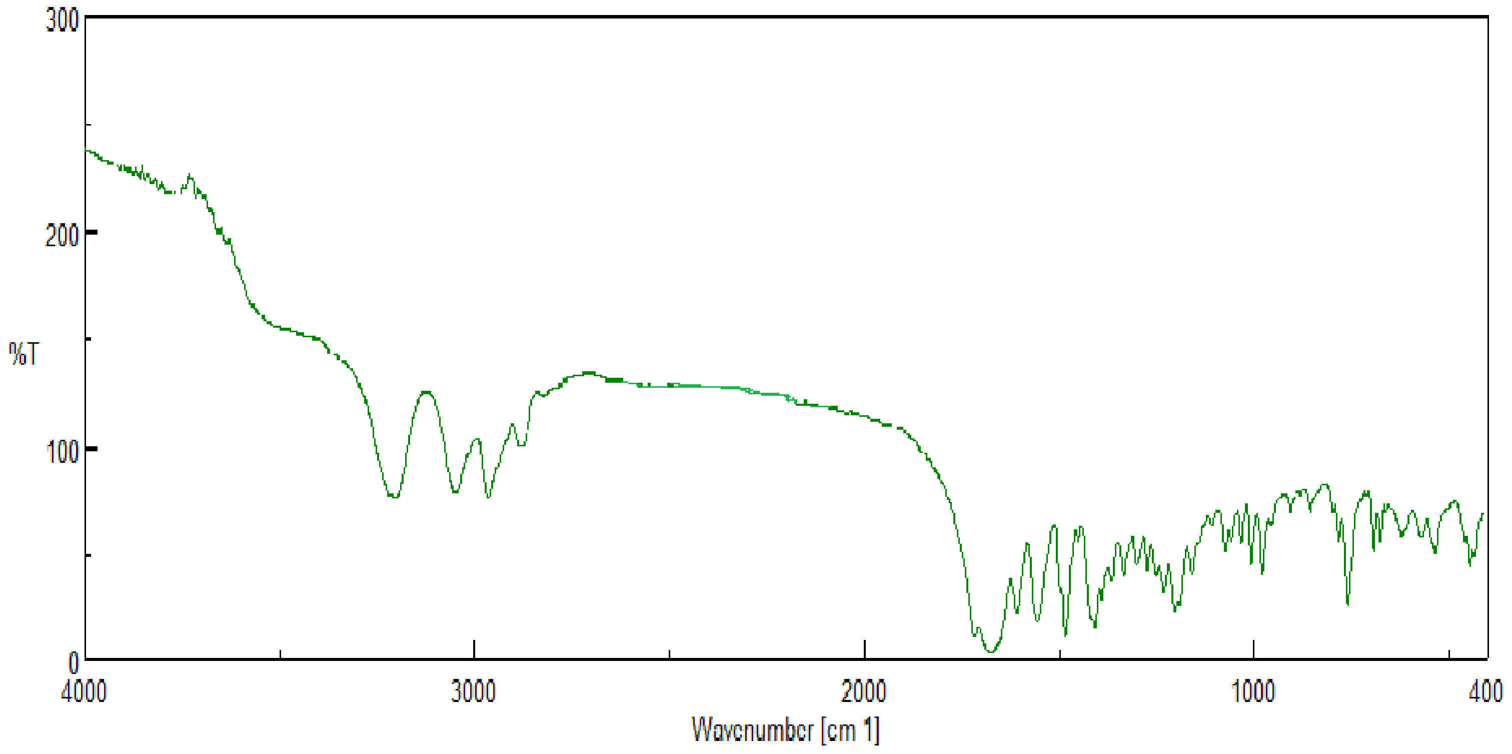

FT-IR of compound (4c)

-

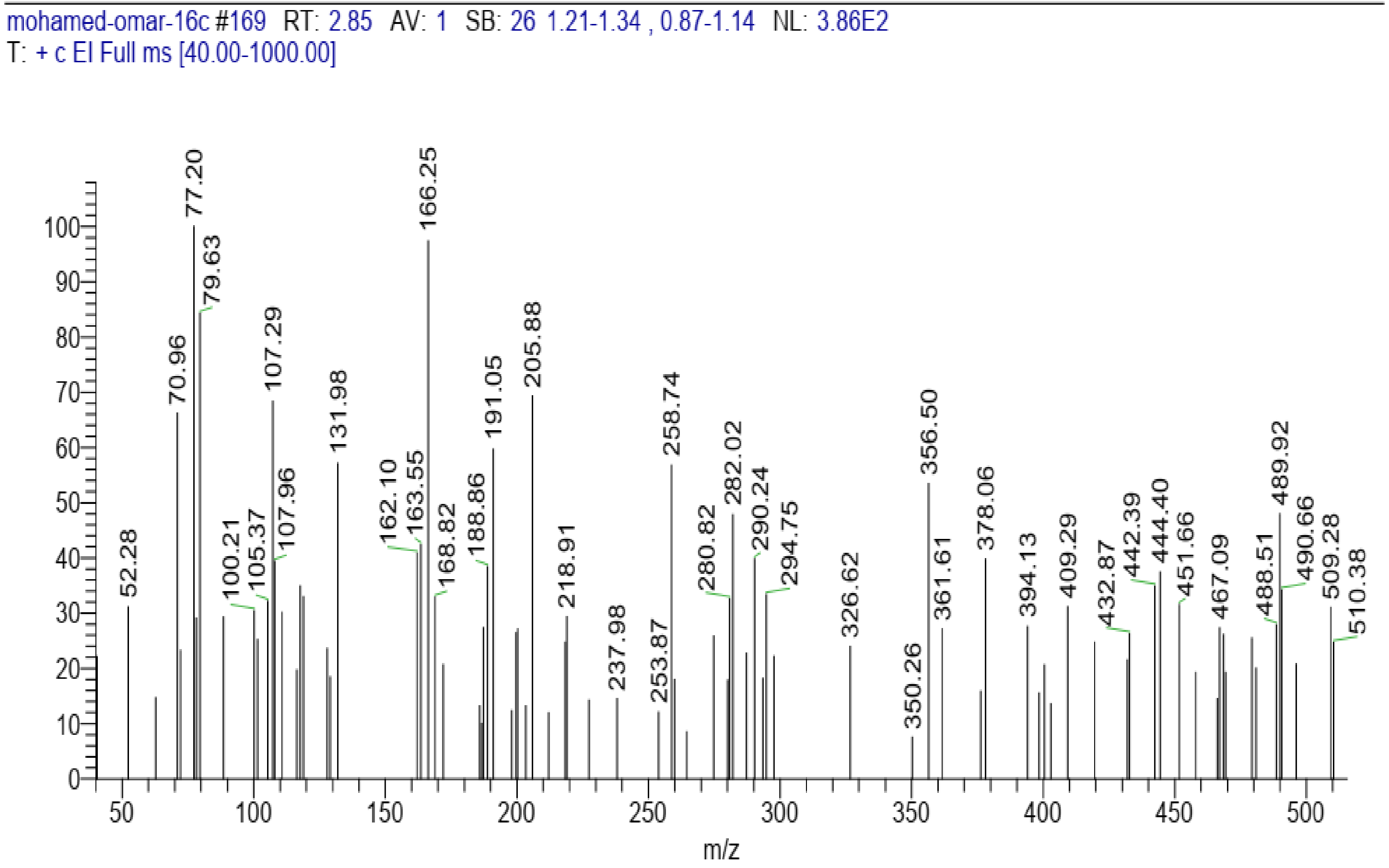

m/z Intensity Relative

MS of compound (4c)

-

-Compound (4d): -

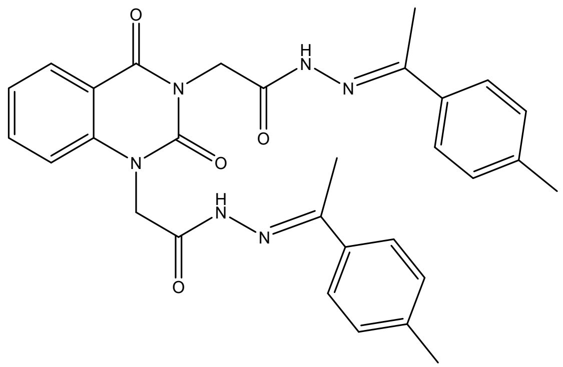

4d

-

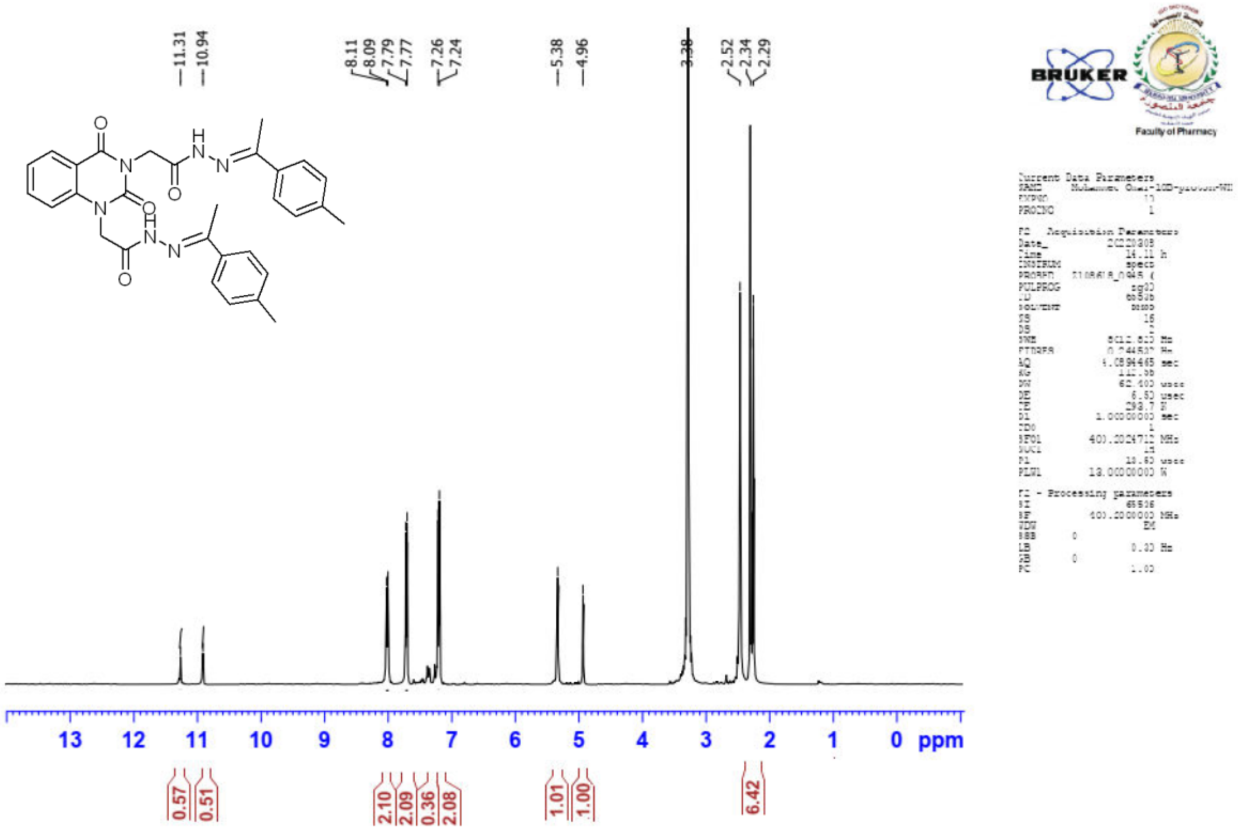

^{1}H-NMR (400 MHz, DMSO-d_6) of compound (4d)

-

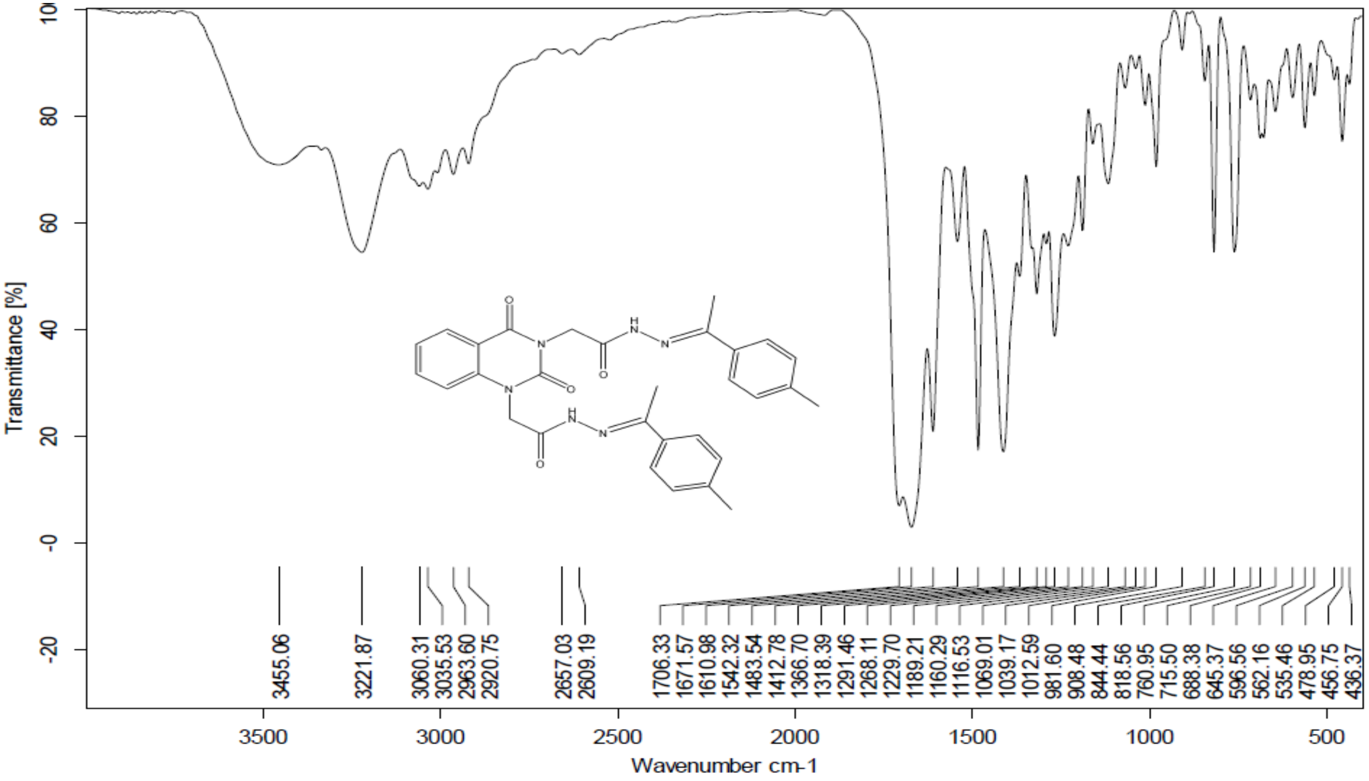

FT-IR of compound (4d)

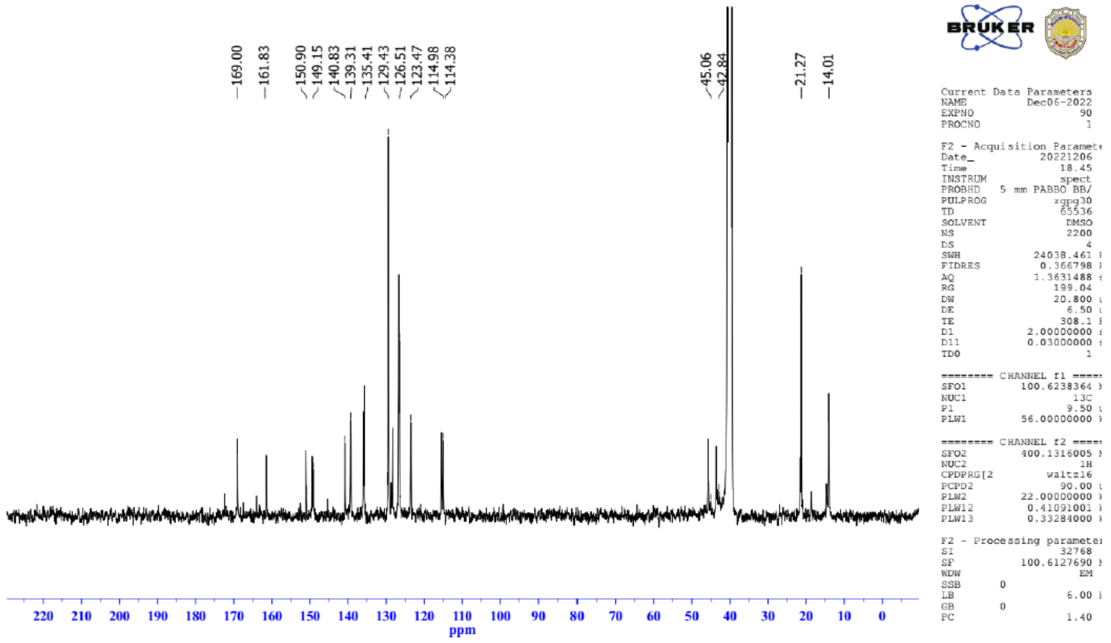

^{13}C-NMR (100 MHz, DMSO) of compound (4d)

-

-Compound (4e):-

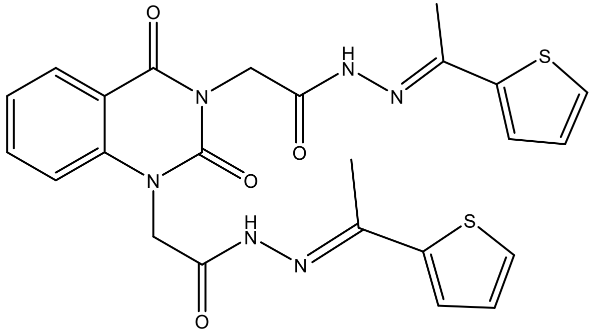

4e

-

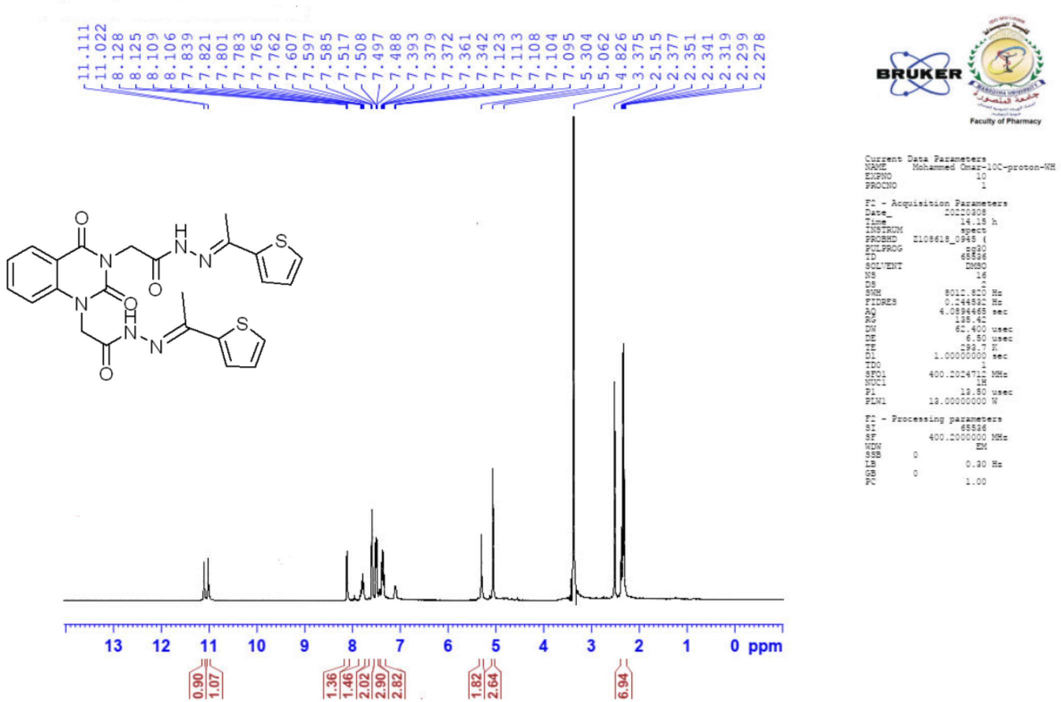

^{1}H-NMR (400 MHz, DMSO-d_6) of compound (4e)

-

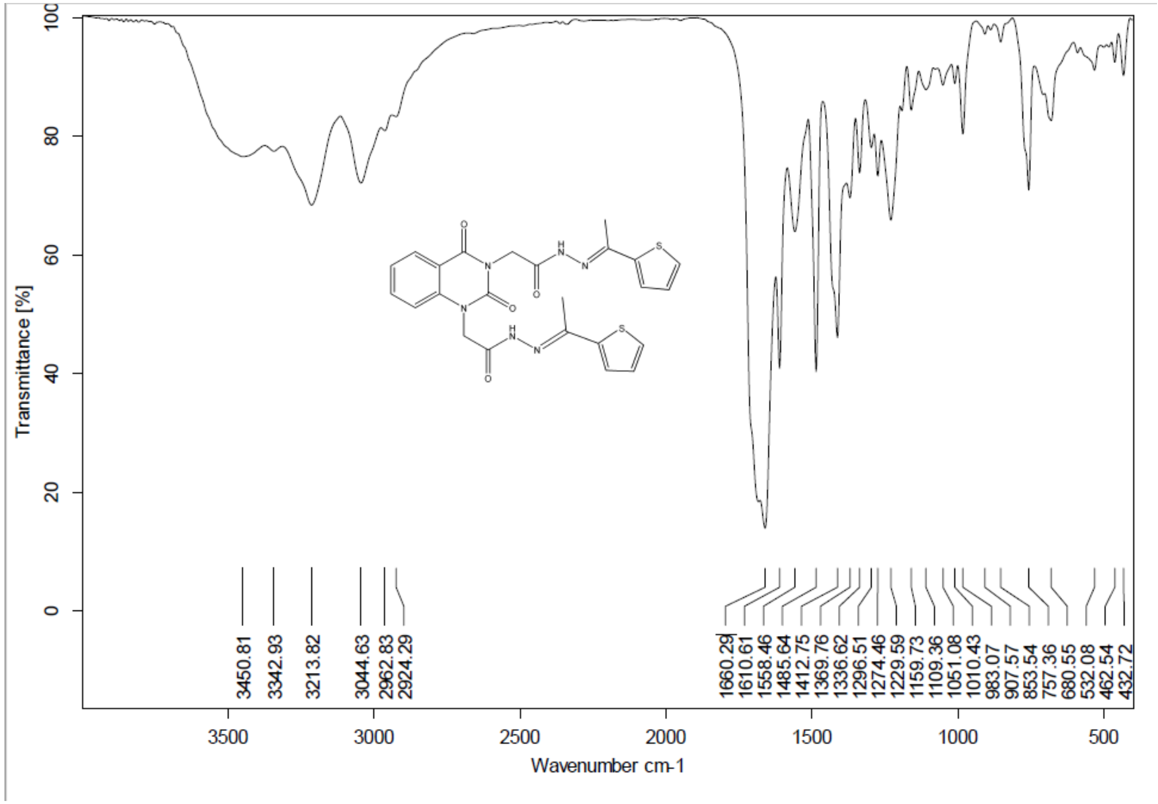

FT-IR of compound (4e)

-

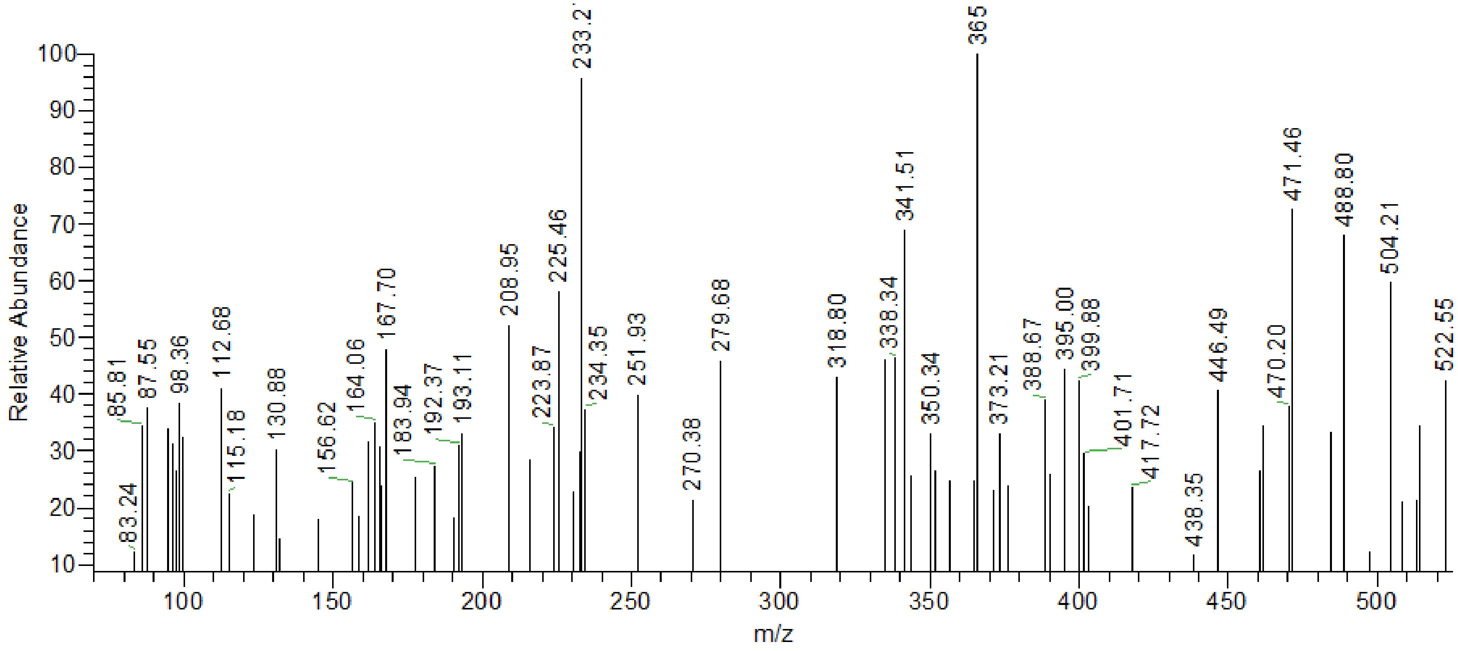

m/z Intensity Relative

MS of compound (4e)

-

-Compound (4f)

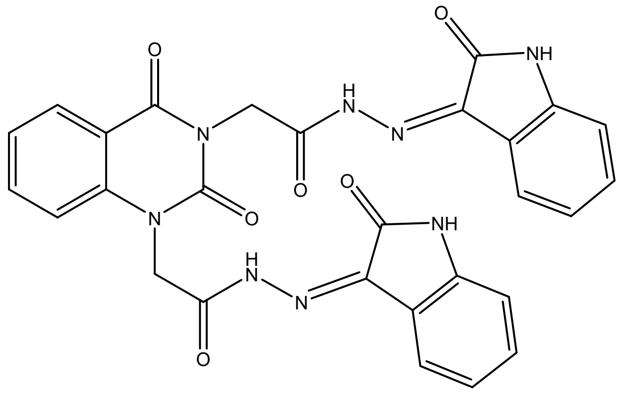

4f

-

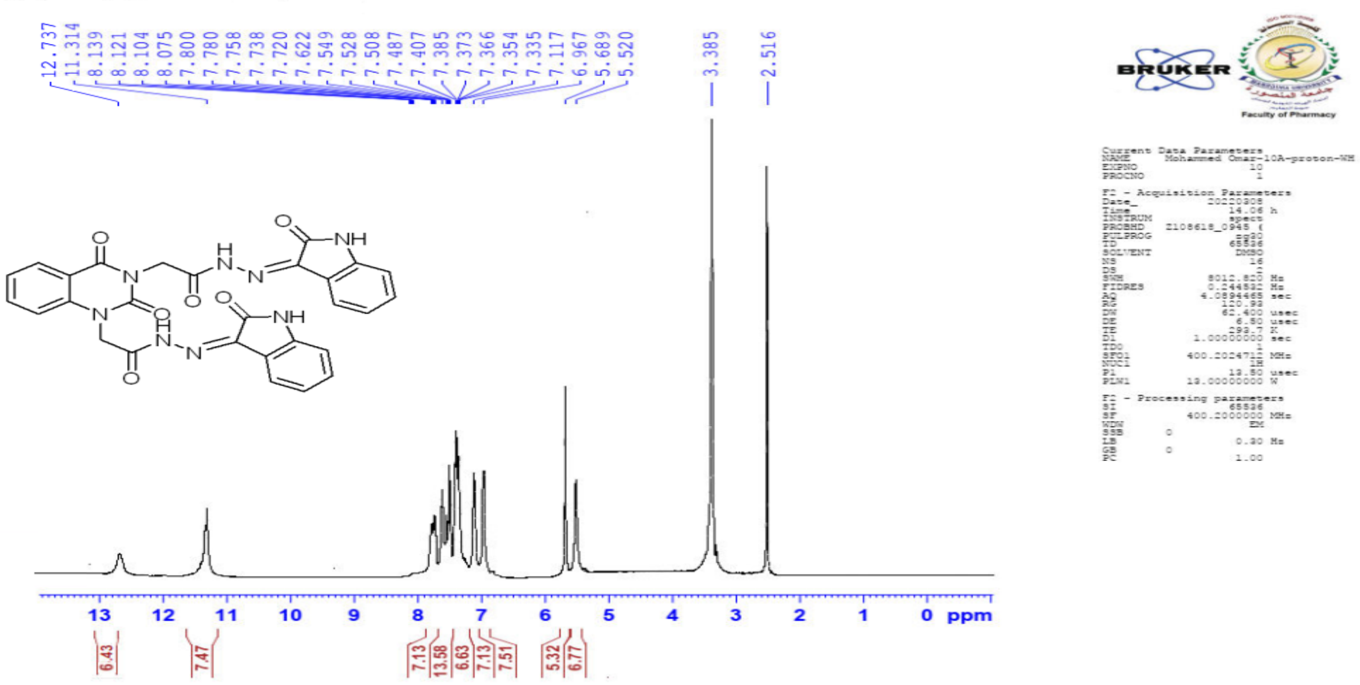

^{1}H-NMR (400 MHz, DMSO-d$_6$) of compound (4f)

-

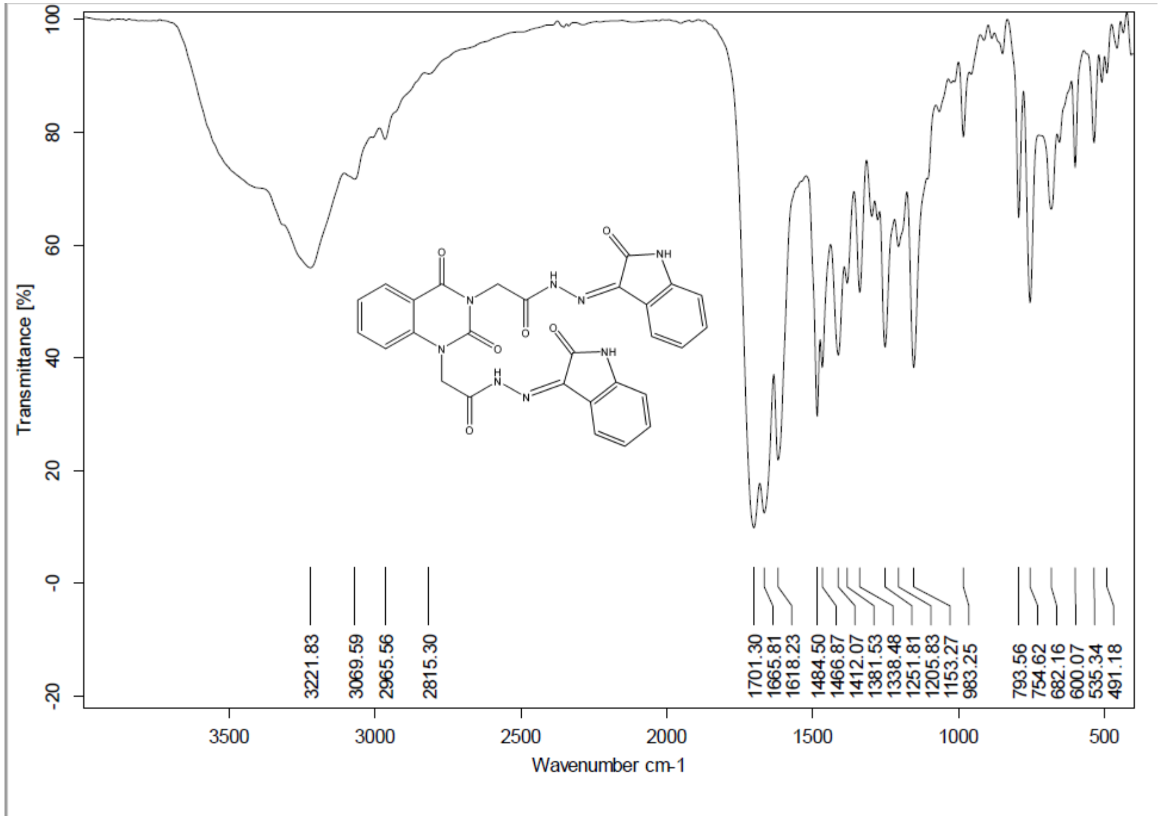

FT-IR of compound (4f)

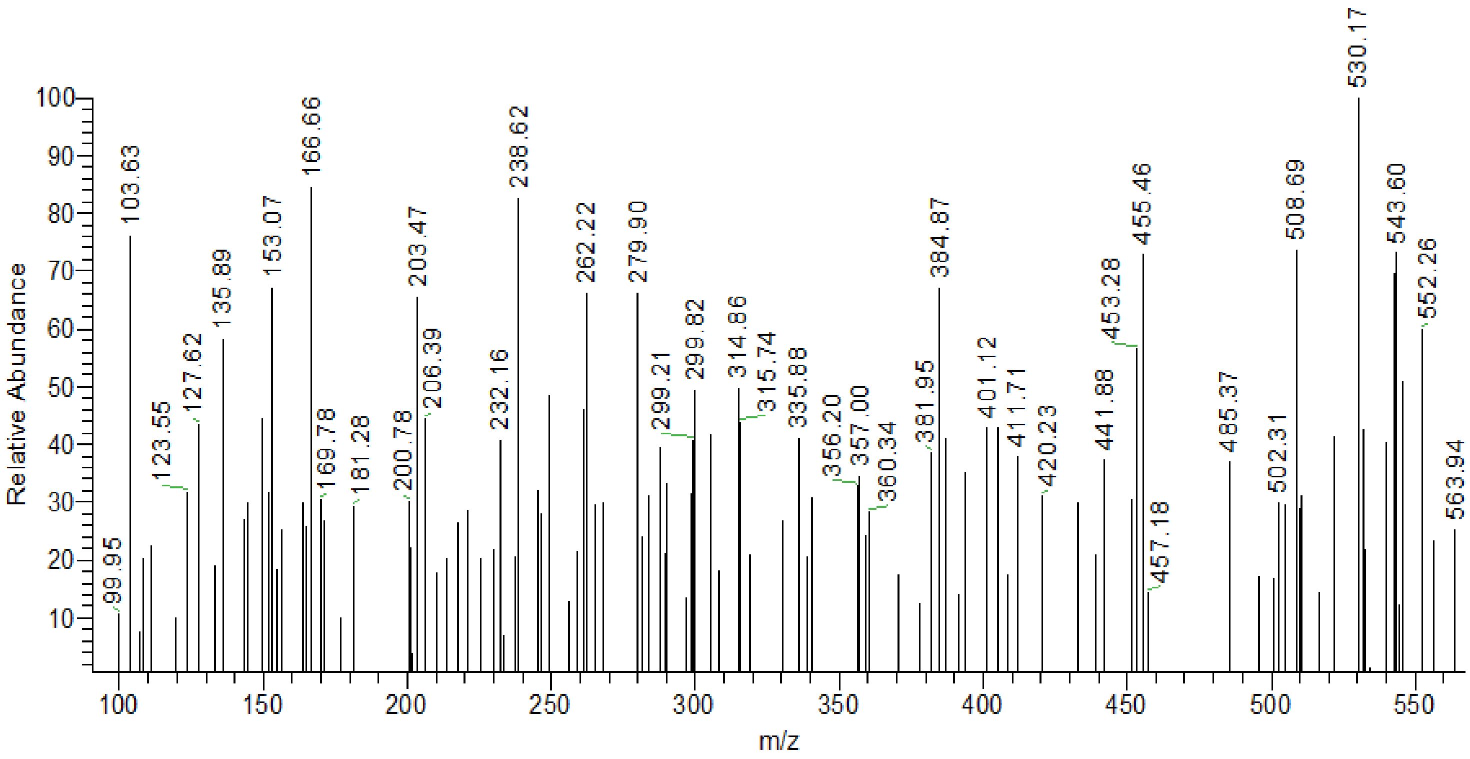

m/z Intensity Relative

MS of compound (4f)

-

-Compound (5a):

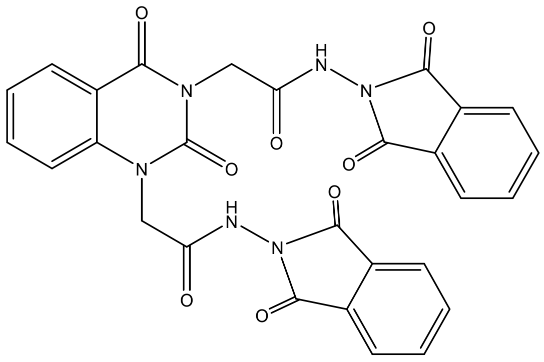

5a

-

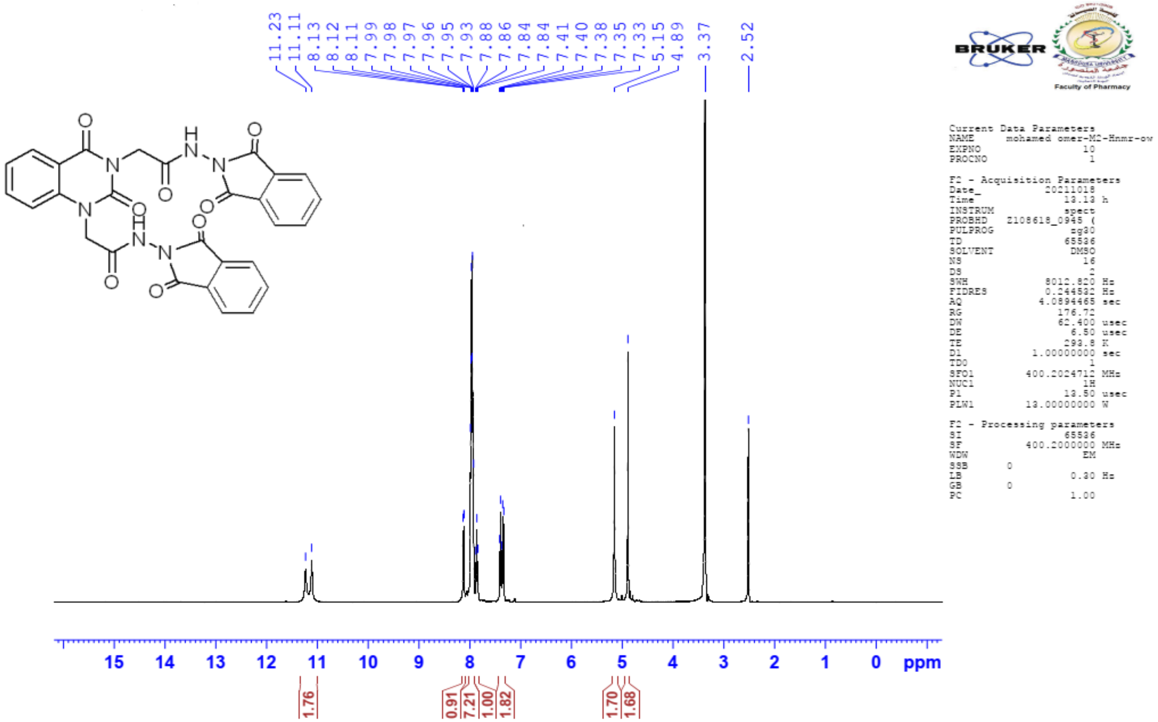

11.23
11.11
8.13
8.12
8.11
7.99
7.98
7.97
7.96
7.95
7.93
7.88
7.86
7.84
7.84
7.41
7.40
7.38
7.35
7.33
5.15
4.89
3.37
2.52
BRUKER
Faculty of Pharmacy
15 14 13 12 11 10 9 8 7 6 5 4 3 2 1 0 ppm
1.76
0.91
7.21
1.00
1.82
1.70
1.68

-

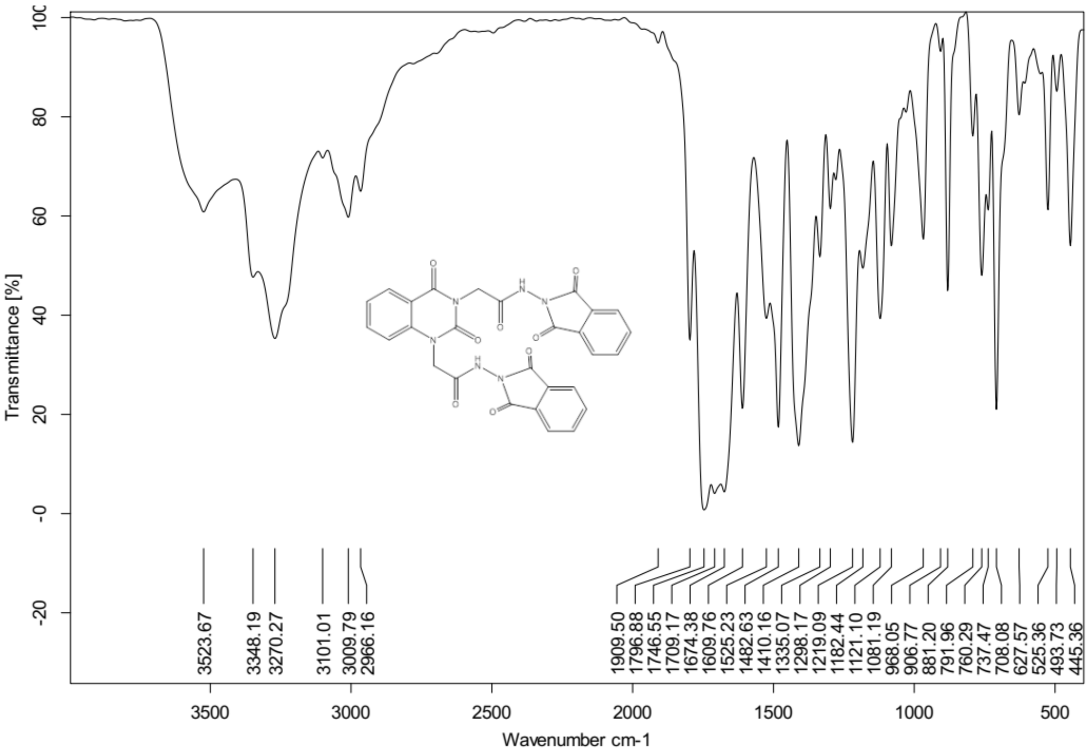

FT-IR of compound (5a)

15-Jan-07 04:04:06

Cairo University
Micro Analytical Center

DI Analysis
Shimadzu Qp-2010 Plus

Sample Information

Analyzed by : Dr. Mai Younis
Analyzed : 15/01/2007 03:55:58 ص
Sample Name : 2a
Sample ID :
Customer Name : Dr. Mohamed Omar - Science - Quena
Data File : C:\GCMSsolution\Data\Project1\2a.QGD
Org Data File : C:\GCMSsolution\Data\Project1\2a.QGD
Method File : C:\GCMSsolution\Data\Project1\High Temperature Op
Org Method File : C:\GCMSsolution\Data\Project1\High Temperature Op
Report File :
Tuning File : C:\GCMSsolution\System\Tune1_default.qgt
$EndIf$Modified by : Dr. Mai Younis
Modified : 15/01/2007 04:00:33 ص

Method

===== Analytical Line 1 =====
IonSourceTemp :250.00 °C
[MS Table]
--Group 1 - Event 1--
Start Time :0.00min
End Time :10.00min
ACQ Mode :Scan
Event Time :0.50sec
Scan Speed :2000
Start m/z :50.00
End m/z :900.00

Electron Voltage : 70 eV
Ionization Mode : EI

C:\GCMSsolution\Data\Project1\2a.QGD

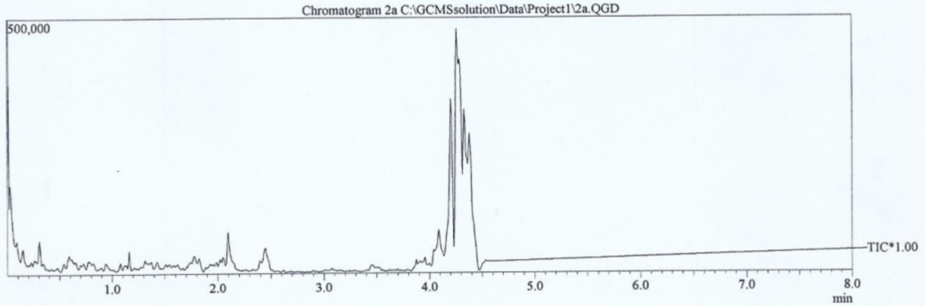

Spectrum

Line#:1 R.Time:4.4(Scan#:526)
MassPeaks:554
RawMode:Single 4.4(526) BasePeak:736(1159)
BG Mode:None Group 1 - Event 1

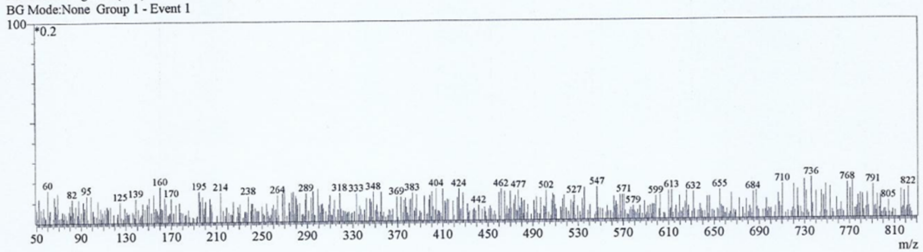

MS of compound (5a)

-

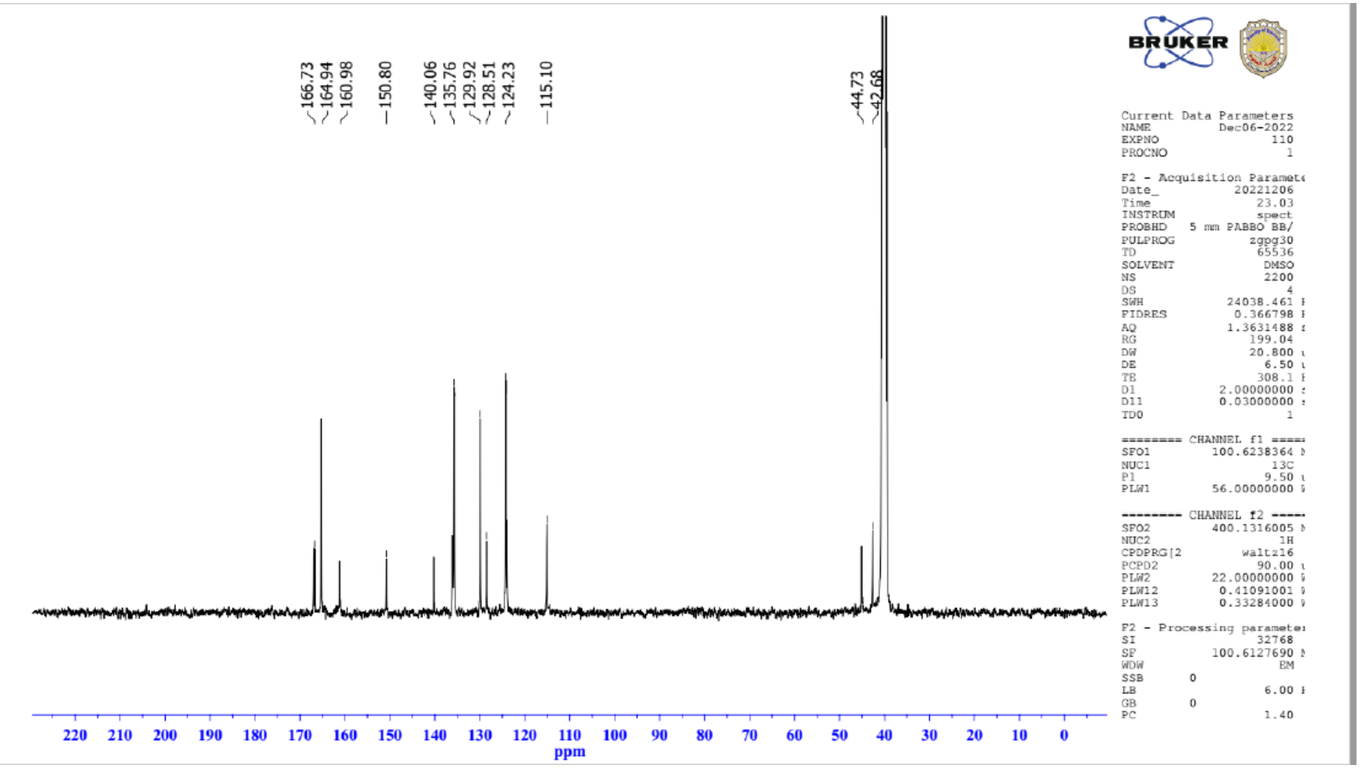

^{13}C-NMR (100 MHz, DMSO) of compound (5a)

-

-Compound (5b):-

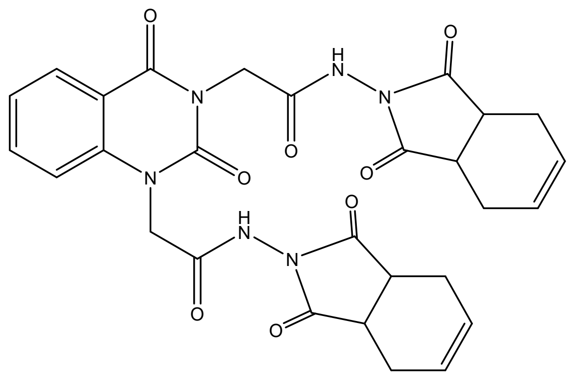

5b

-

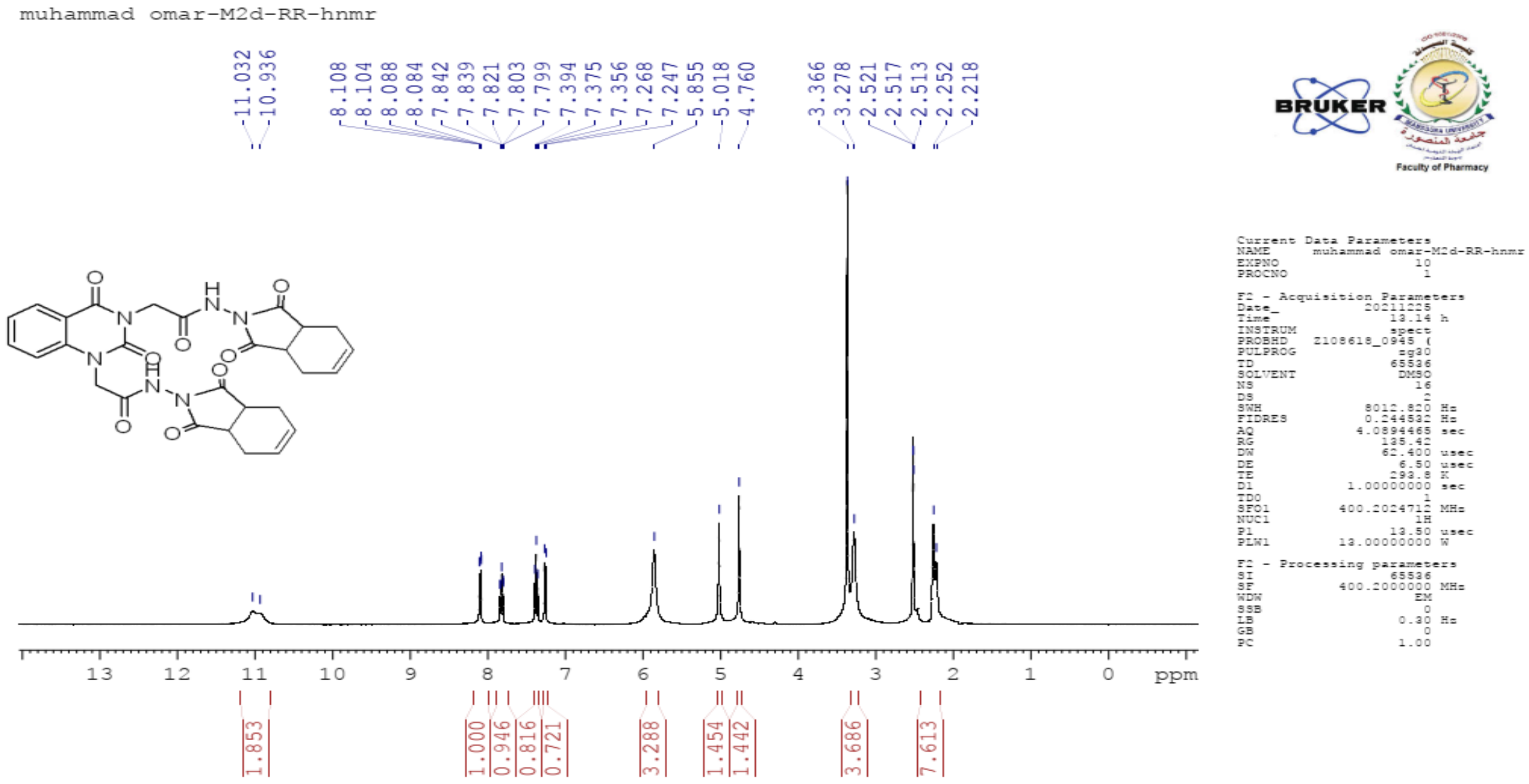

^{1}H-NMR (400 MHz, DMSO-d_6) of compound (5b)

-

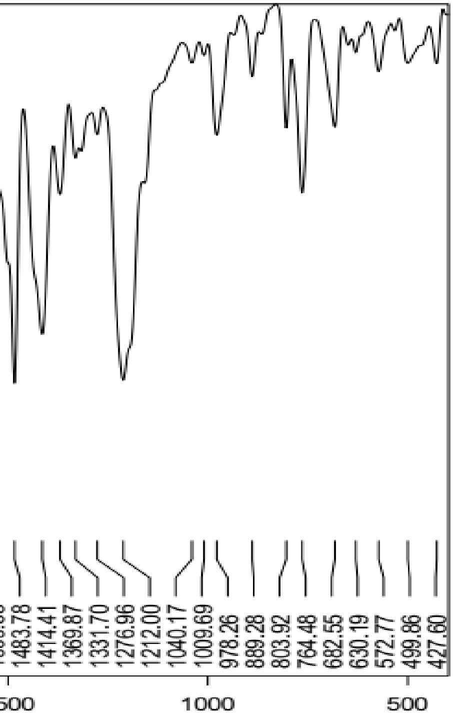

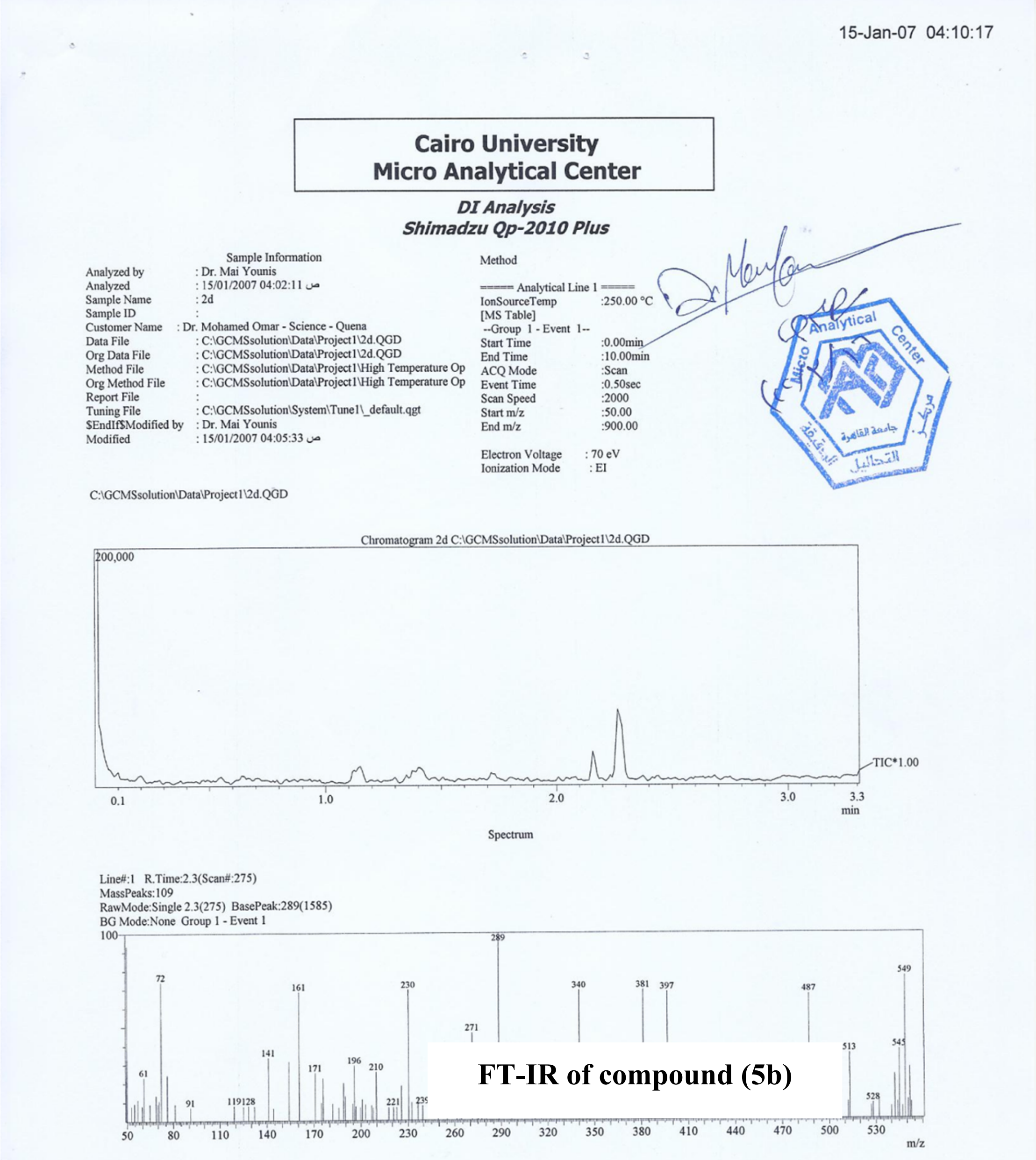

15-Jan-07 04:10:17

Cairo University
Micro Analytical Center

DI Analysis
Shimadzu Qp-2010 Plus

Sample Information

Analyzed by : Dr. Mai Younis
Analyzed : 15/01/2007 04:02:11 ص
Sample Name : 2d
Sample ID :
Customer Name : Dr. Mohamed Omar - Science - Quena
Data File : C:\GCMSsolution\Data\Project1\2d.QGD
Org Data File : C:\GCMSsolution\Data\Project1\2d.QGD
Method File : C:\GCMSsolution\Data\Project1\High Temperature Op
Org Method File : C:\GCMSsolution\Data\Project1\High Temperature Op
Report File :
Tuning File : C:\GCMSsolution\System\Tune1_default.qgt
$EndIf$Modified by : Dr. Mai Younis
Modified : 15/01/2007 04:05:33 ص

Method

===== Analytical Line 1 =====
IonSourceTemp :250.00 °C
[MS Table]
--Group 1 - Event 1--
Start Time :0.00min
End Time :10.00min
ACQ Mode :Scan
Event Time :0.50sec
Scan Speed :2000
Start m/z :50.00
End m/z :900.00

Electron Voltage : 70 eV
Ionization Mode : EI

C:\GCMSsolution\Data\Project1\2d.QGD

Chromatogram 2d C:\GCMSsolution\Data\Project1\2d.QGD

Spectrum

Line#:1 R.Time:2.3(Scan#:275)
MassPeaks:109
RawMode:Single 2.3(275) BasePeak:289(1585)
BG Mode:None Group 1 - Event 1

FT-IR of compound (5b)

-

-Compound (5c)

MS of compound (5b)

-

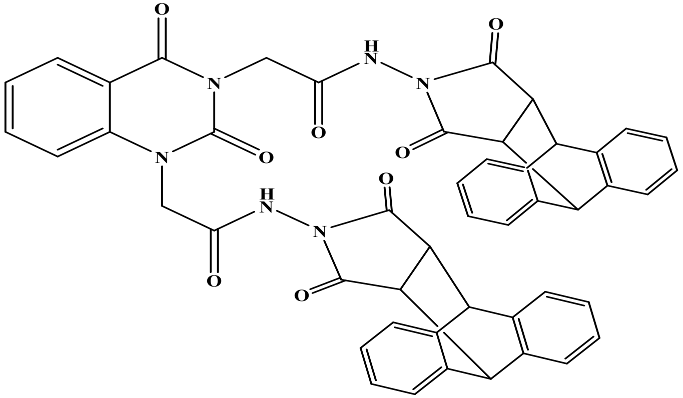

5c

-

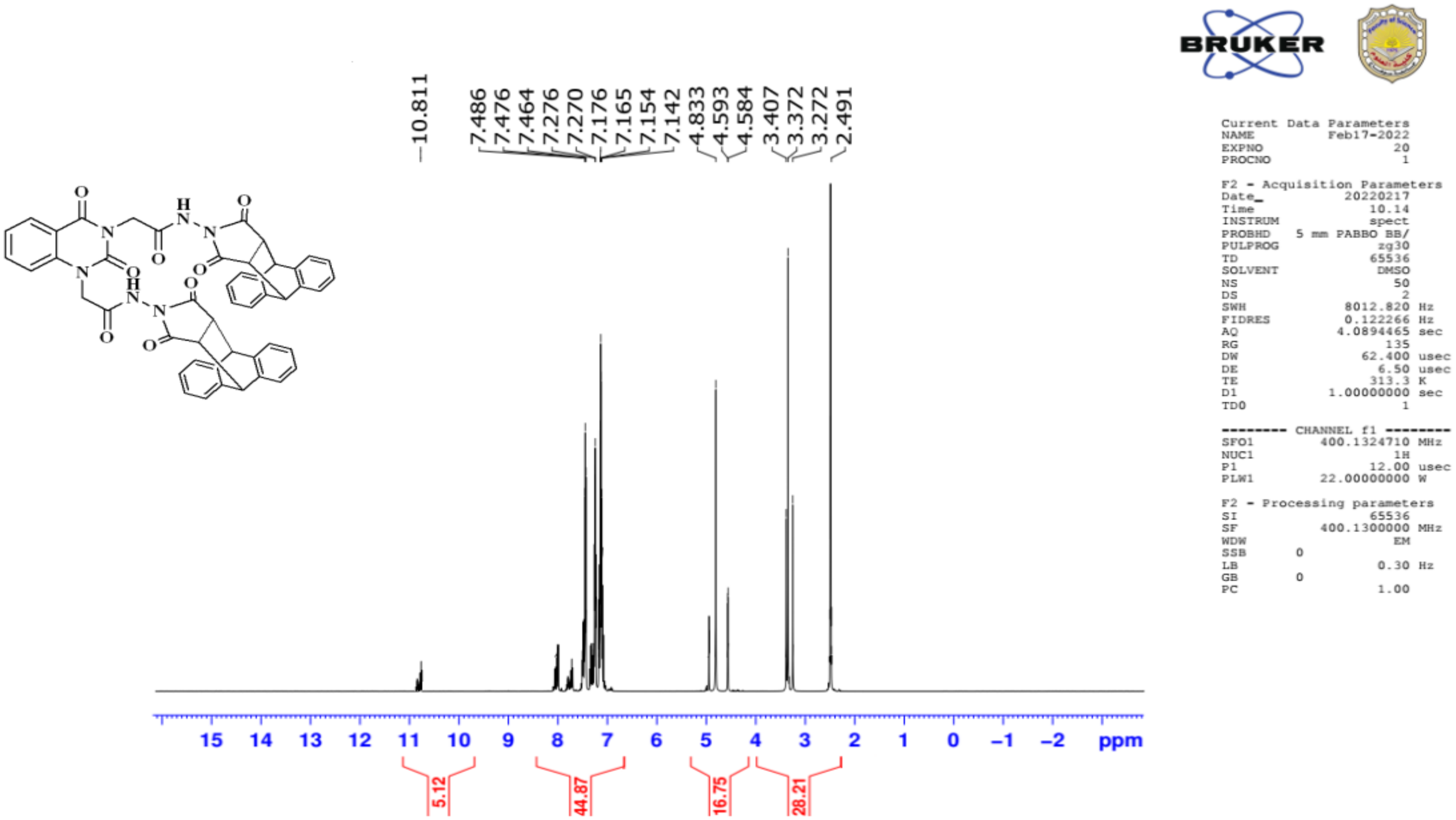

^{1}H-NMR (400 MHz, DMSO-d_6) of compound (5c)

-

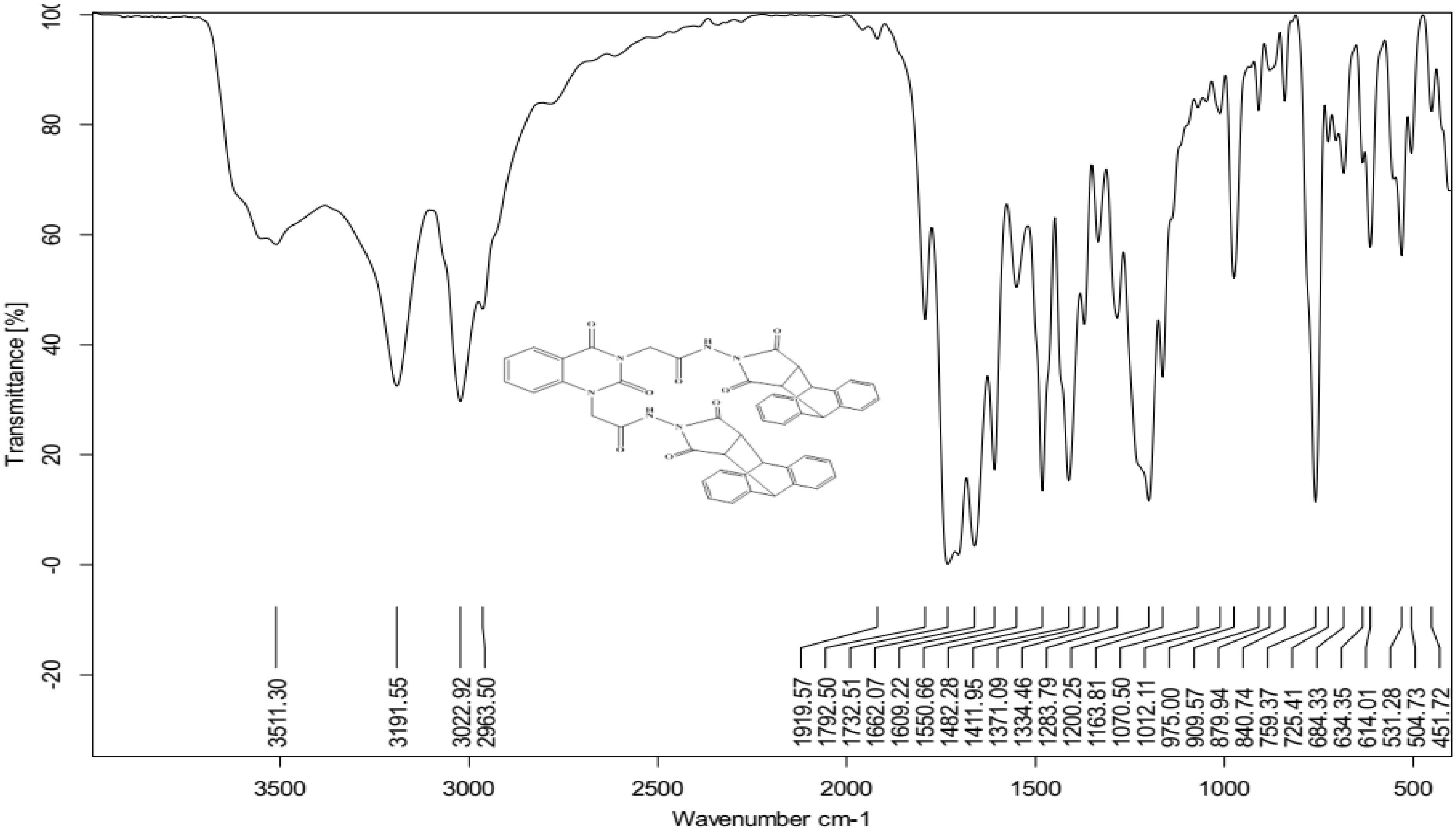

FT-IR of compound (5c)

-

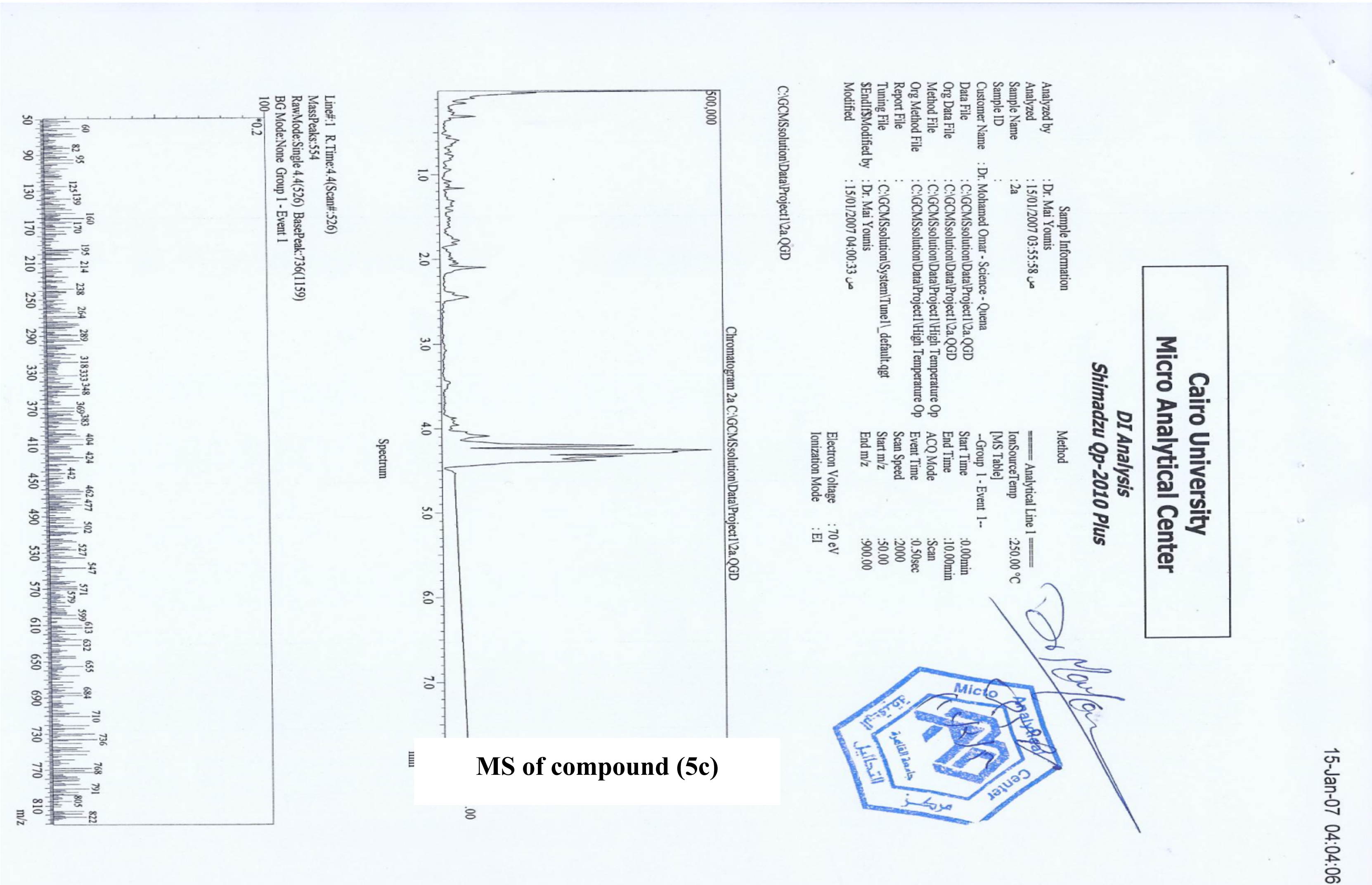

MS of compound (5c)

-

-Compound (6a)

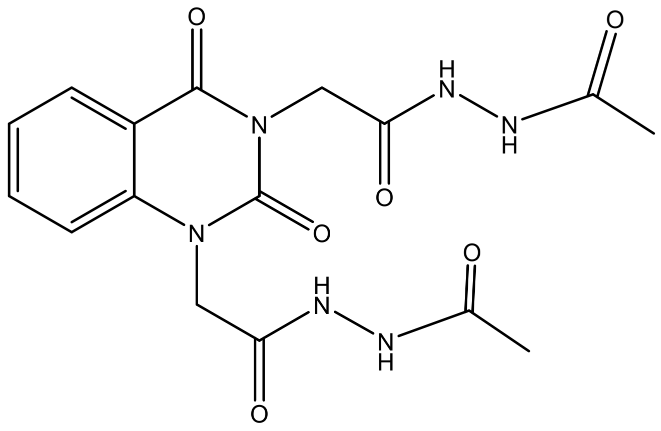

6a

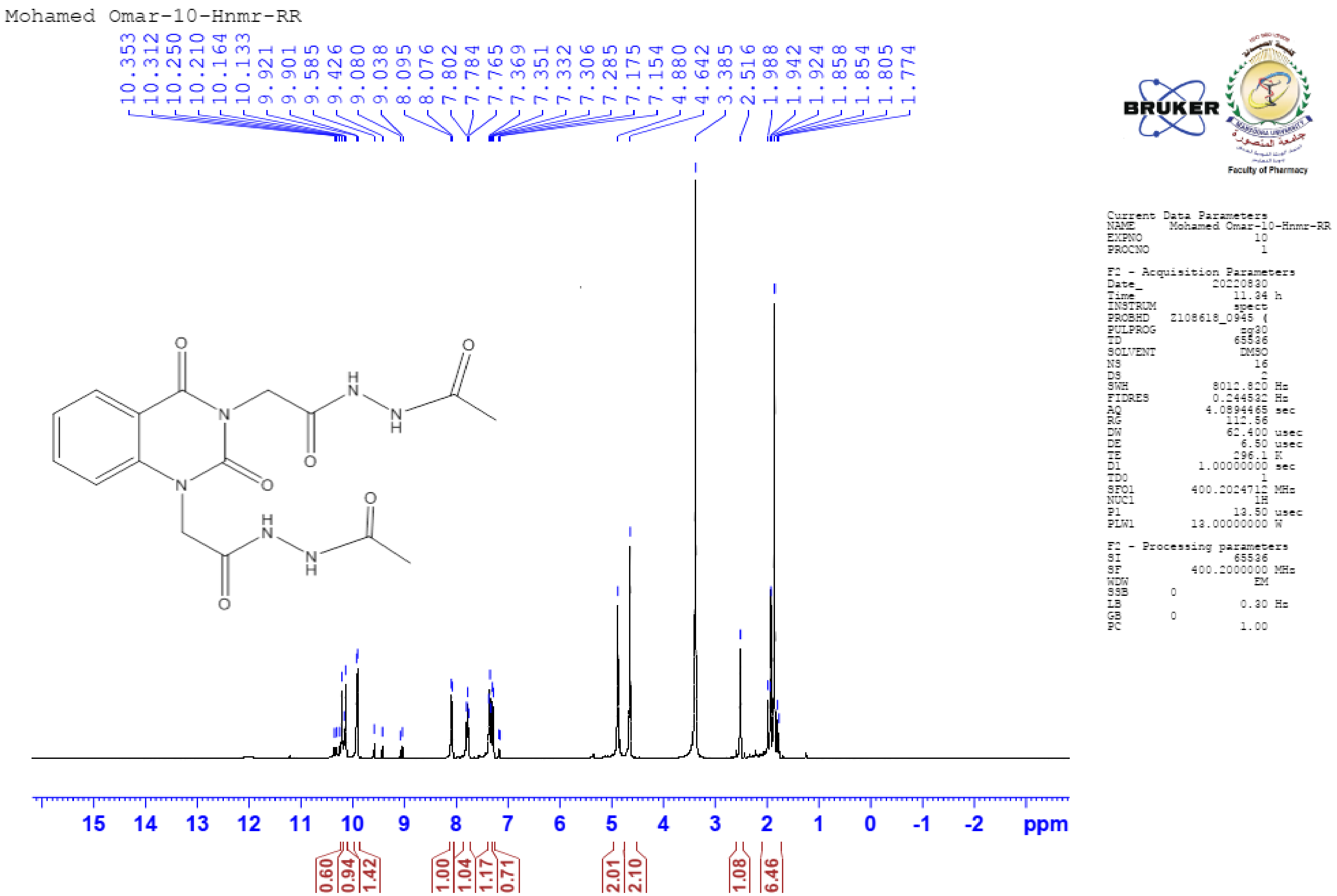

^{1}H-NMR (400 MHz, DMSO-d_6) of compound (6a)

-

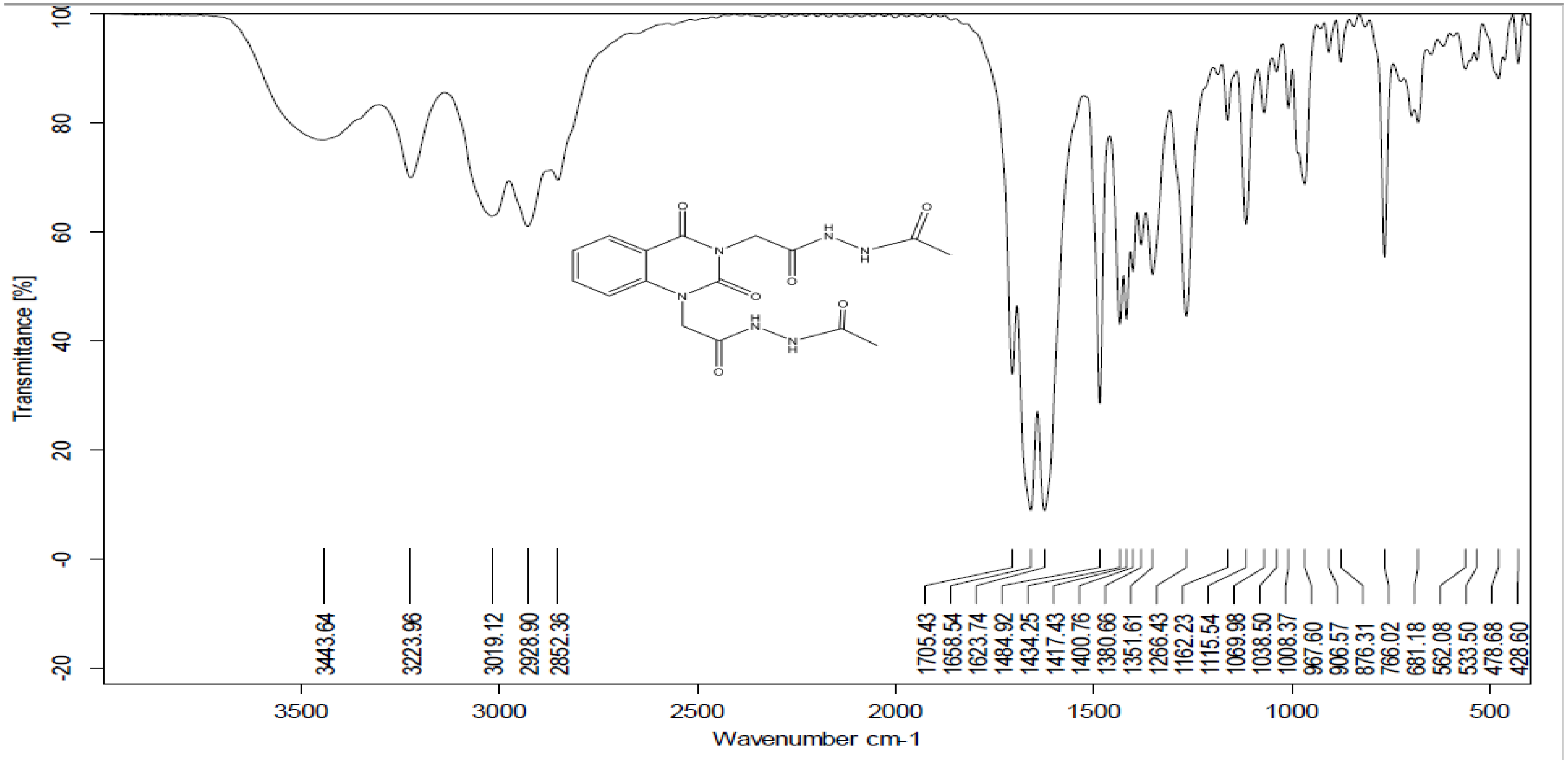

Transmittance [%]
Wavenumber cm-1
3443.64
3223.96
3019.12
2928.90
2852.36
1705.43
1658.54
1623.74
1484.92
1434.25
1417.43
1400.76
1380.66
1351.61
1266.43
1162.23
1115.54
1069.98
1038.50
1008.37
967.60
906.57
876.31
766.02
681.18
562.08
533.50
478.68
428.60

-

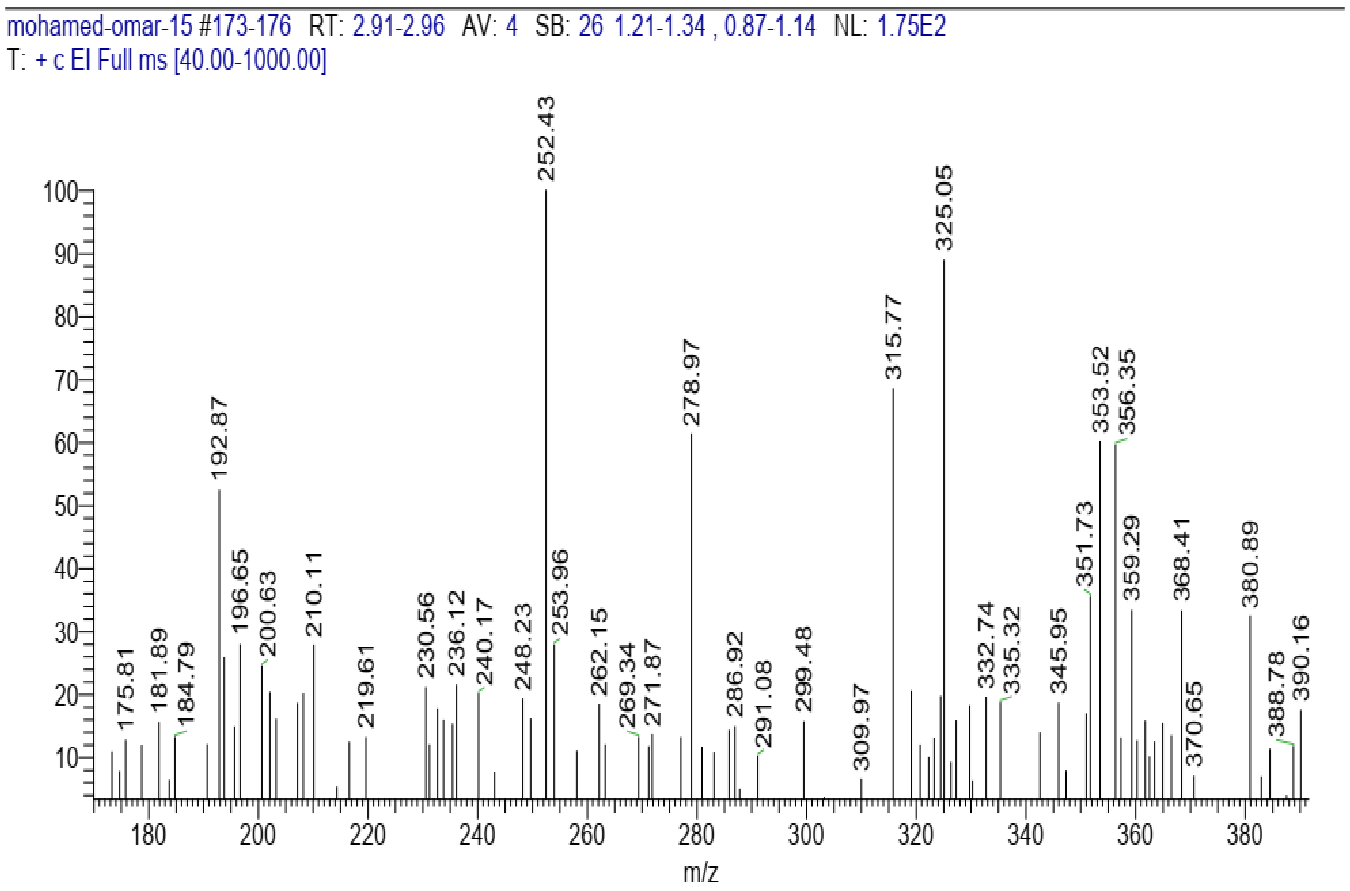

m/z Intensity Relative

MS of compound (6a)

-

-Compound (6b)

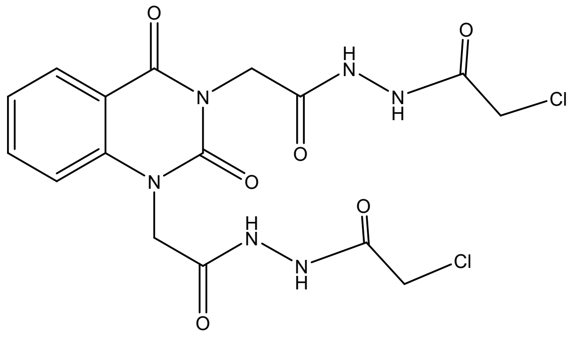

6b

-

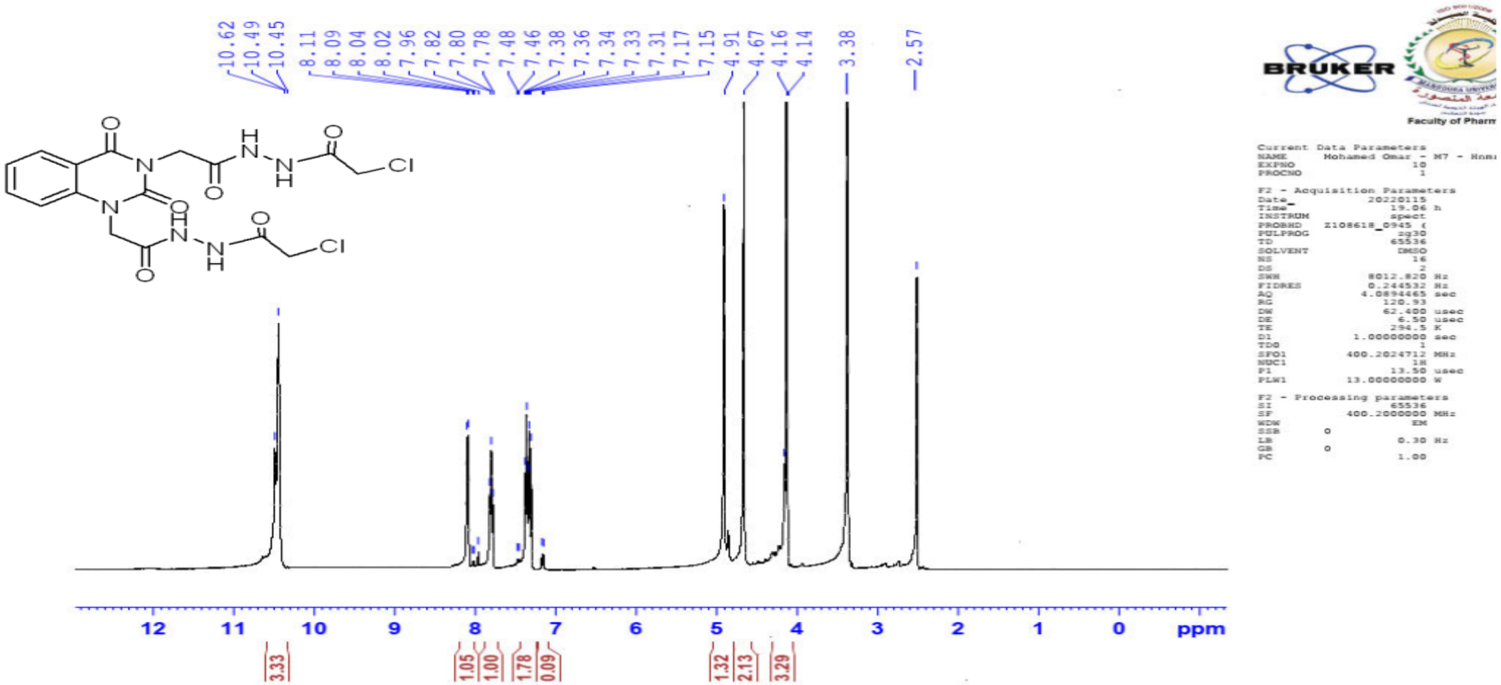

10.62
10.49
10.45
8.11
8.09
8.04
8.02
7.96
7.82
7.80
7.78
7.48
7.46
7.38
7.36
7.34
7.33
7.31
7.17
7.15
4.91
4.67
4.16
4.14
3.38
2.57
O
N
H
Cl
BRUKER
Faculty of Pharm
Current Data Parameters
NAME Mohamed Omar - M7 - Hnm
EXPNO 10
PROCNO 1
F2 - Acquisition Parameters
Date_ 20220115
Time 19.06 h
INSTRUM spect
PROBHD Z108618_0945 (
PULPROG zg30
TD 65536
SOLVENT DMSO
NS 16
DS 2
SWH 8012.820 Hz
FIDRES 0.244532 Hz
AQ 4.0894465 sec
RG 120.93
DW 62.400 usec
DE 6.50 usec
TE 294.5 K
D1 1.00000000 sec
TD0 1
SFO1 400.2024712 MHz
NUC1 1H
P1 13.50 usec
PLW1 13.00000000 W
F2 - Processing parameters
SI 65536
SF 400.2000000 MHz
WDW EM
SSB 0
LB 0.30 Hz
GB 0
PC 1.00
12 11 10 9 8 7 6 5 4 3 2 1 0 ppm
3.33
1.05
1.00
1.78
0.09
1.32
2.13
3.29

-

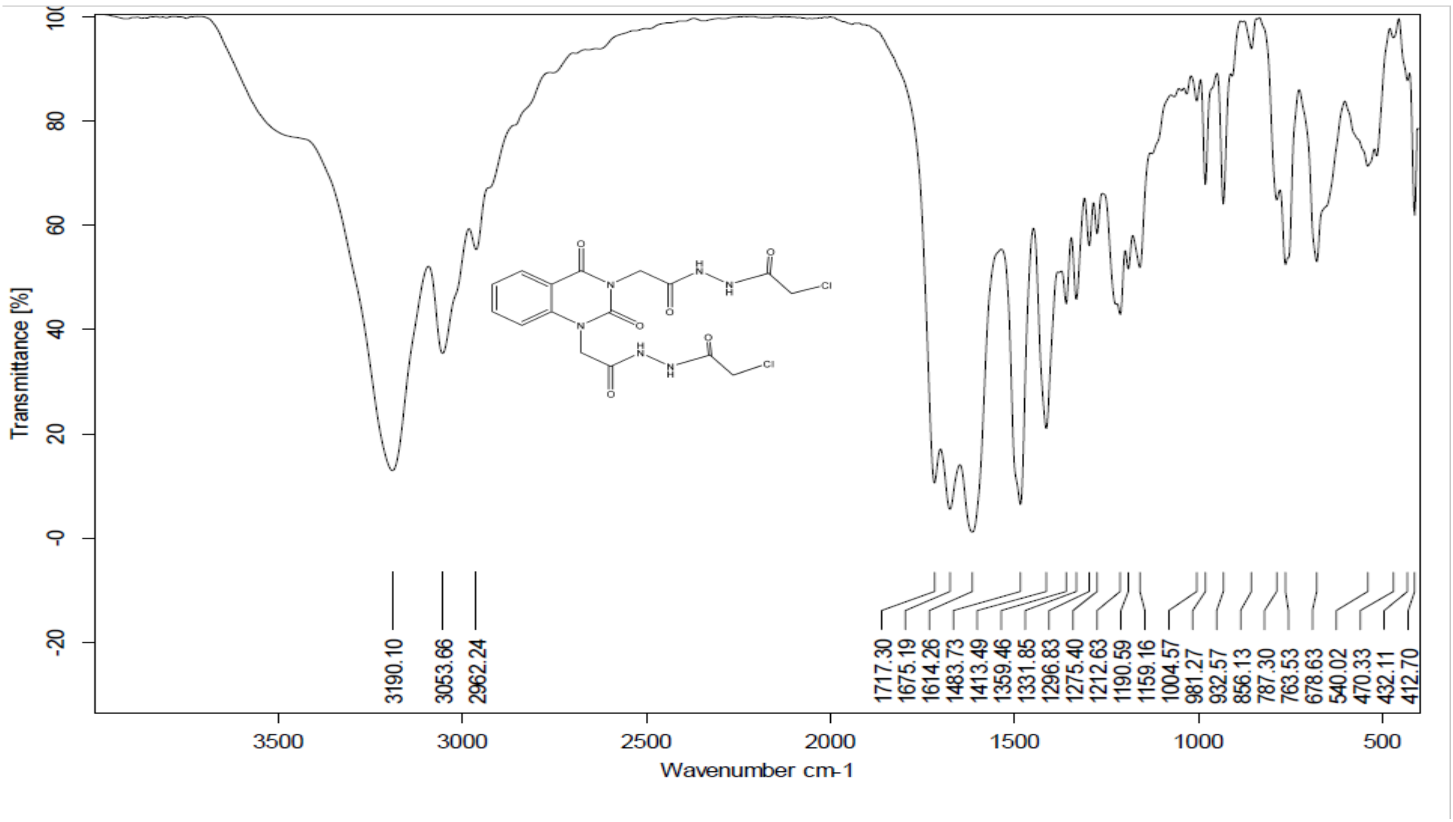

FT-IR of compound (6b)

-

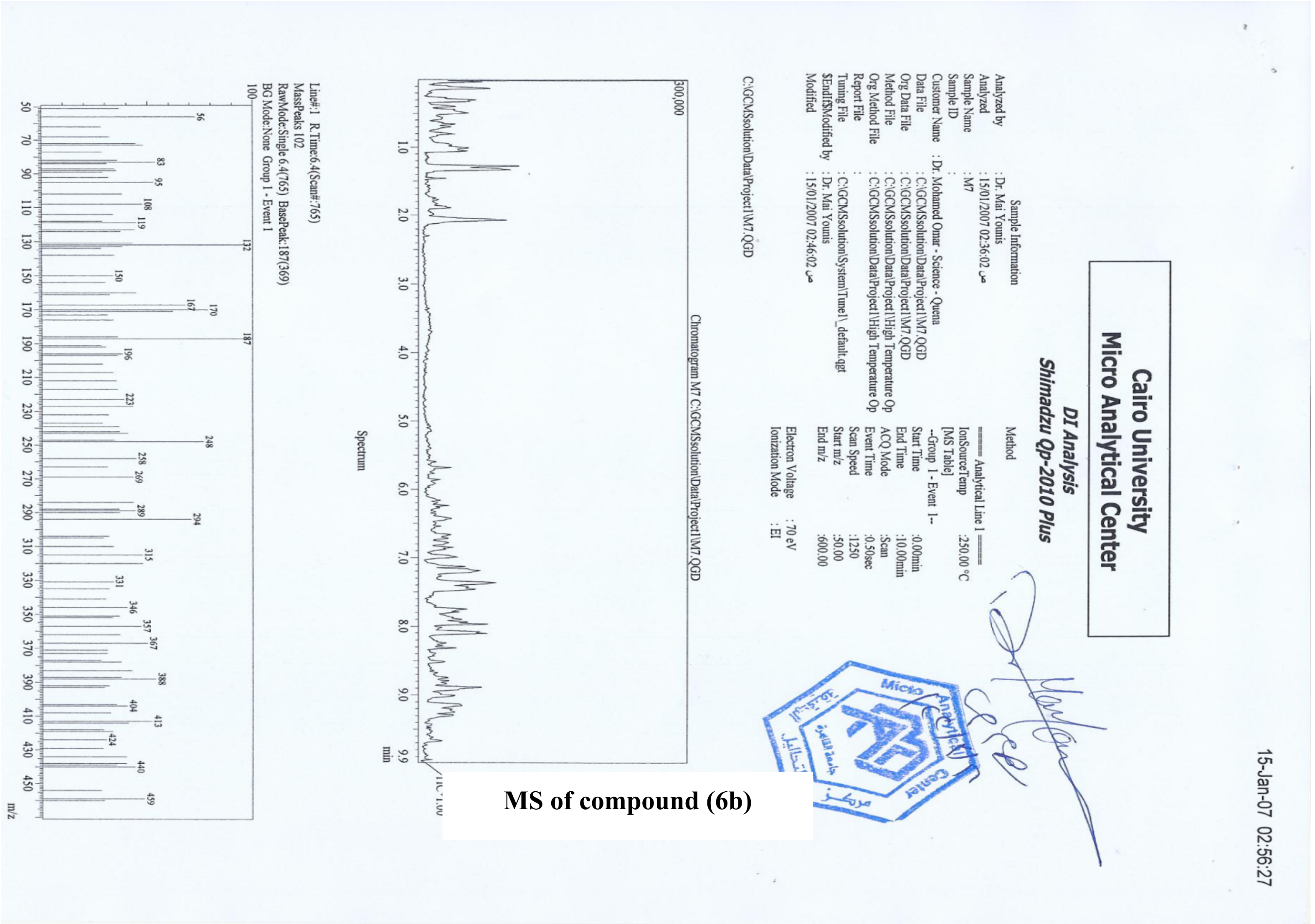

MS of compound (6b)

-

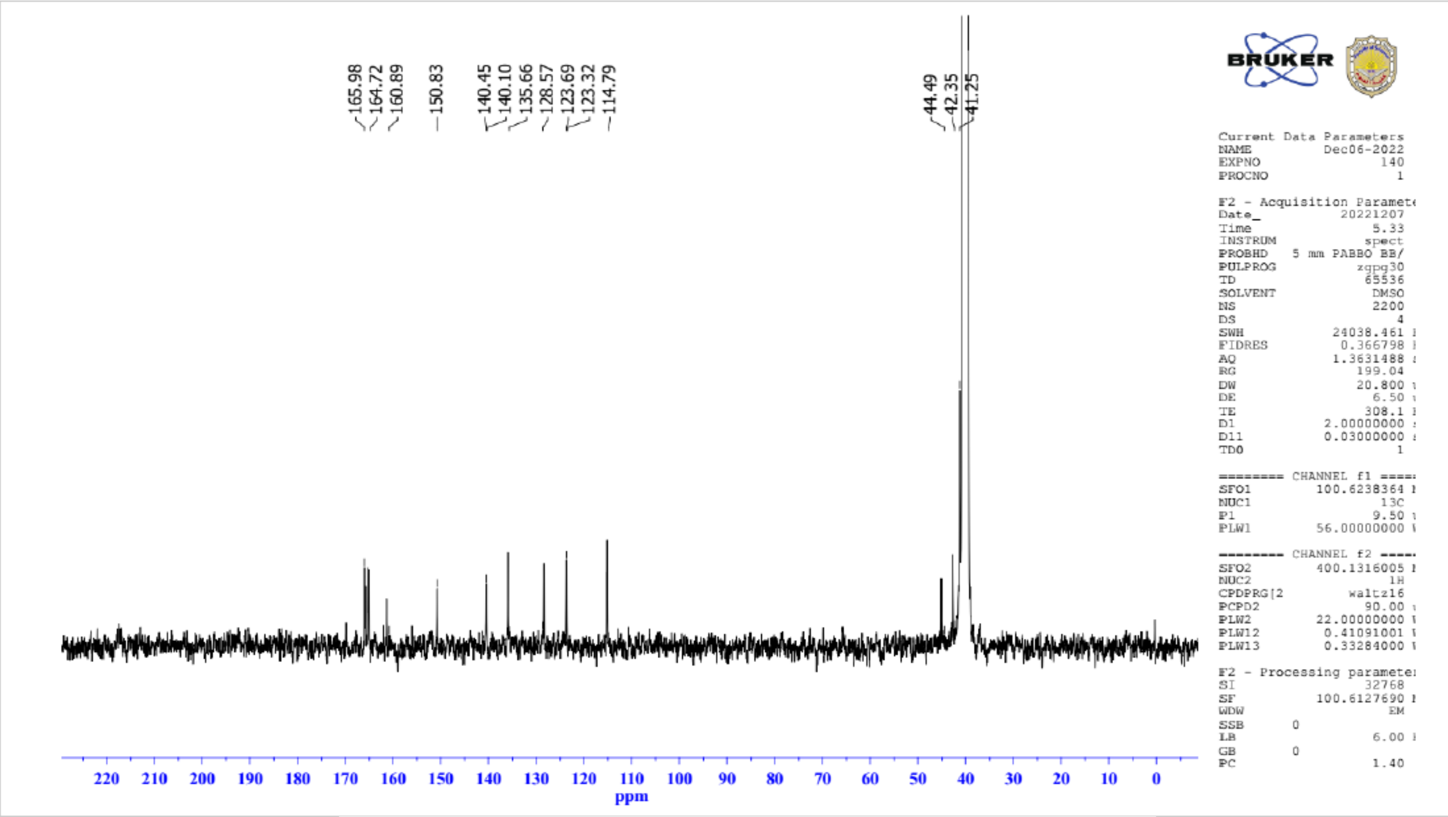

^{13}C-NMR (100 MHz, DMSO) of compound (6b)

-

-Compound (6c)

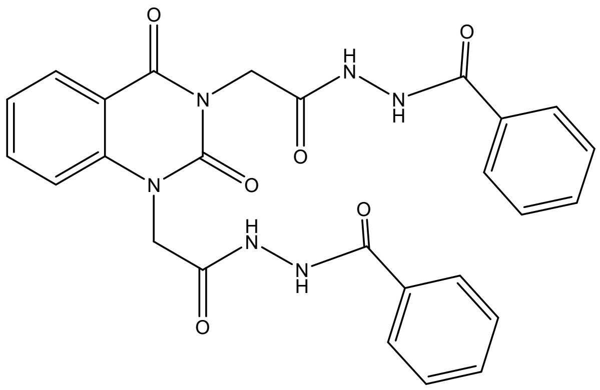

6c

-

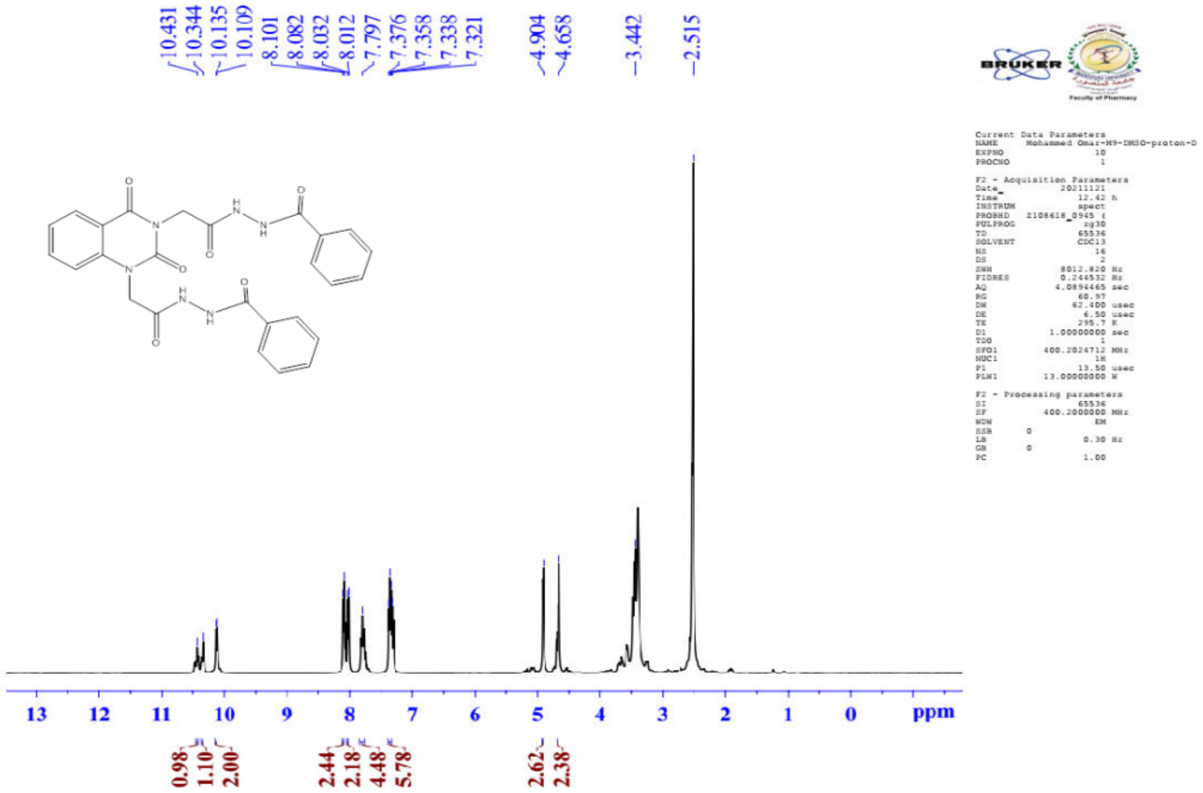

^{1}H-NMR (400 MHz, DMSO-d_6) of compound (6c)

-

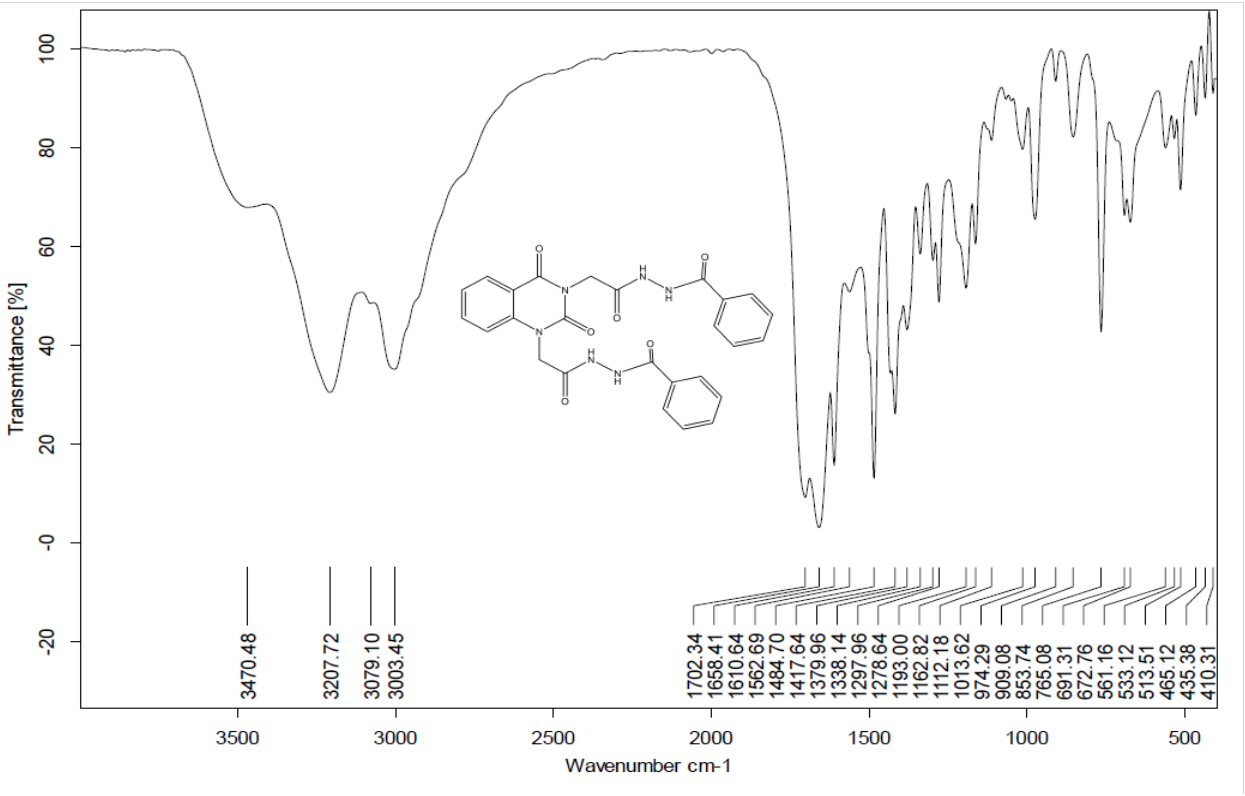

FT-IR of compound (6c)

-

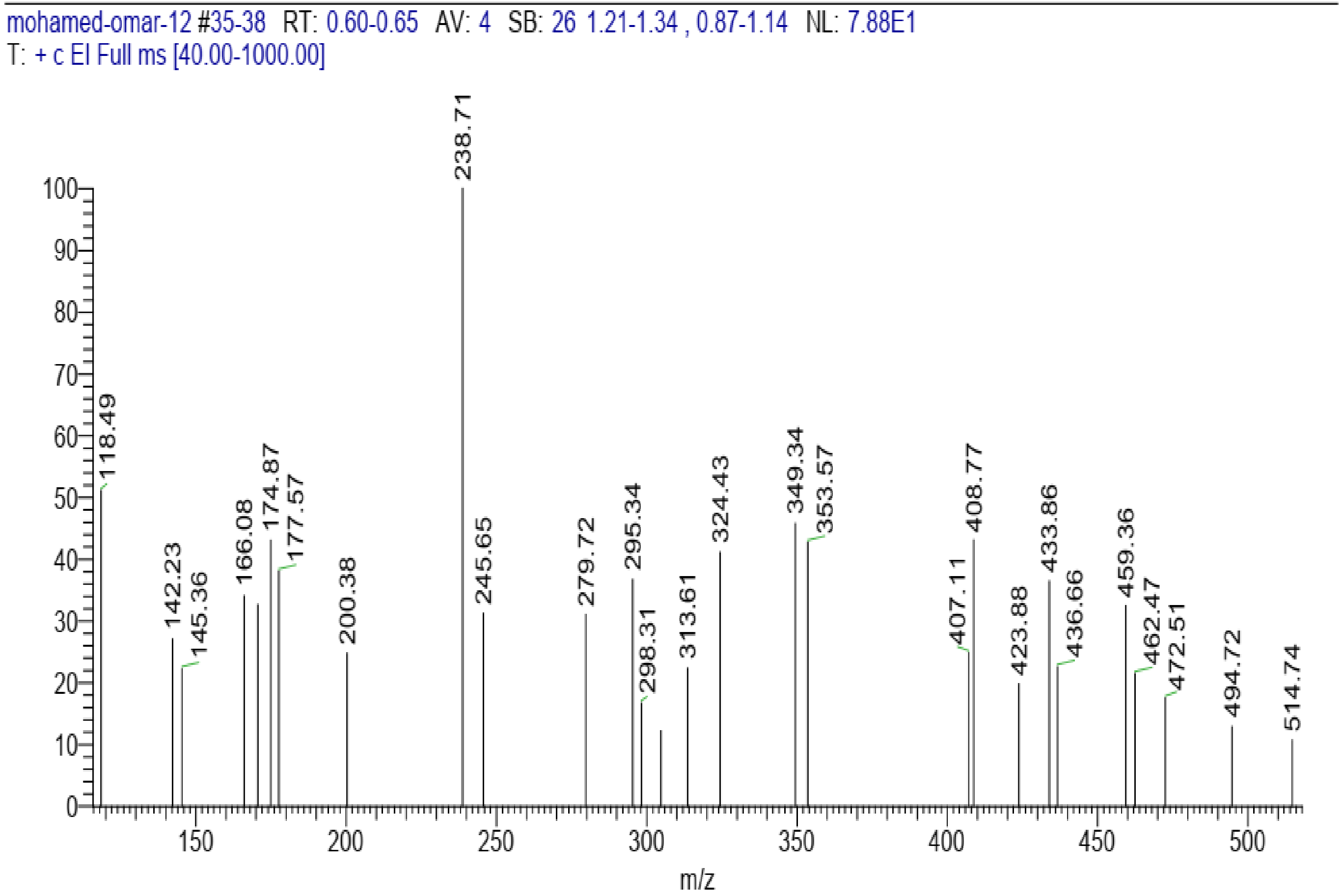

m/z Intensity Relative

MS of compound (6c)

-

-Compound (6d)

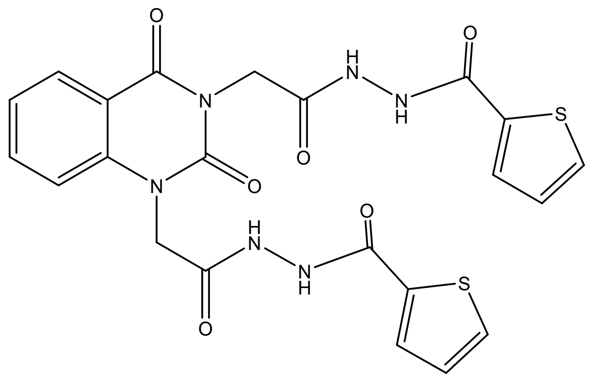

6d

-

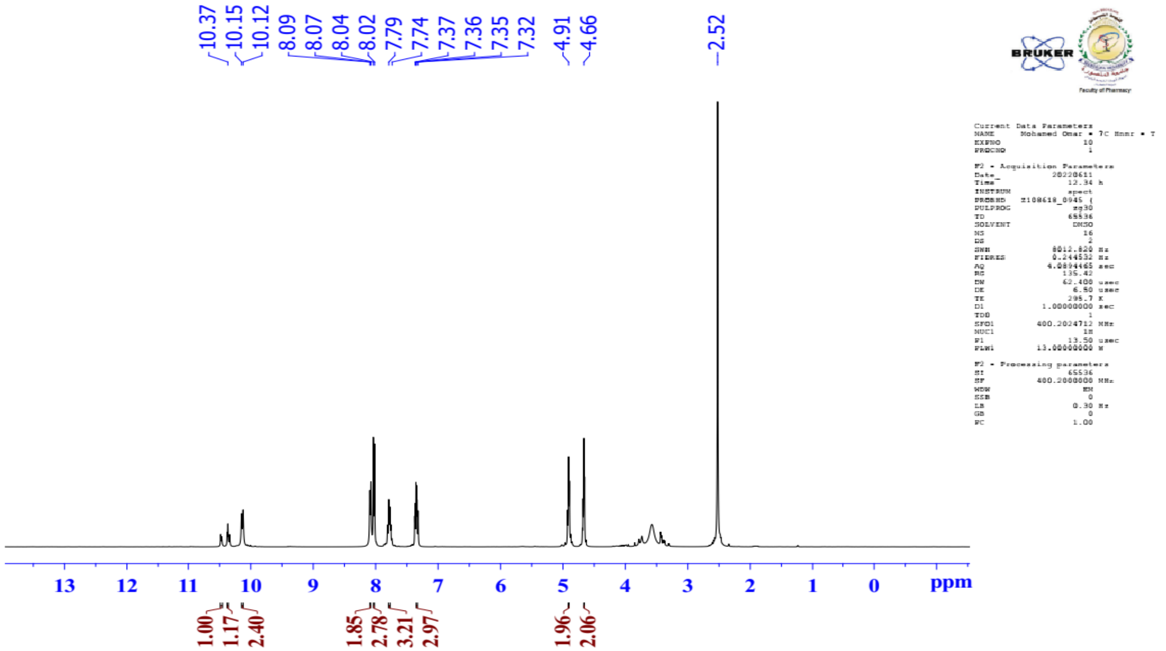

^{1}H-NMR (400 MHz, DMSO-d_6) of compound (6d)

-

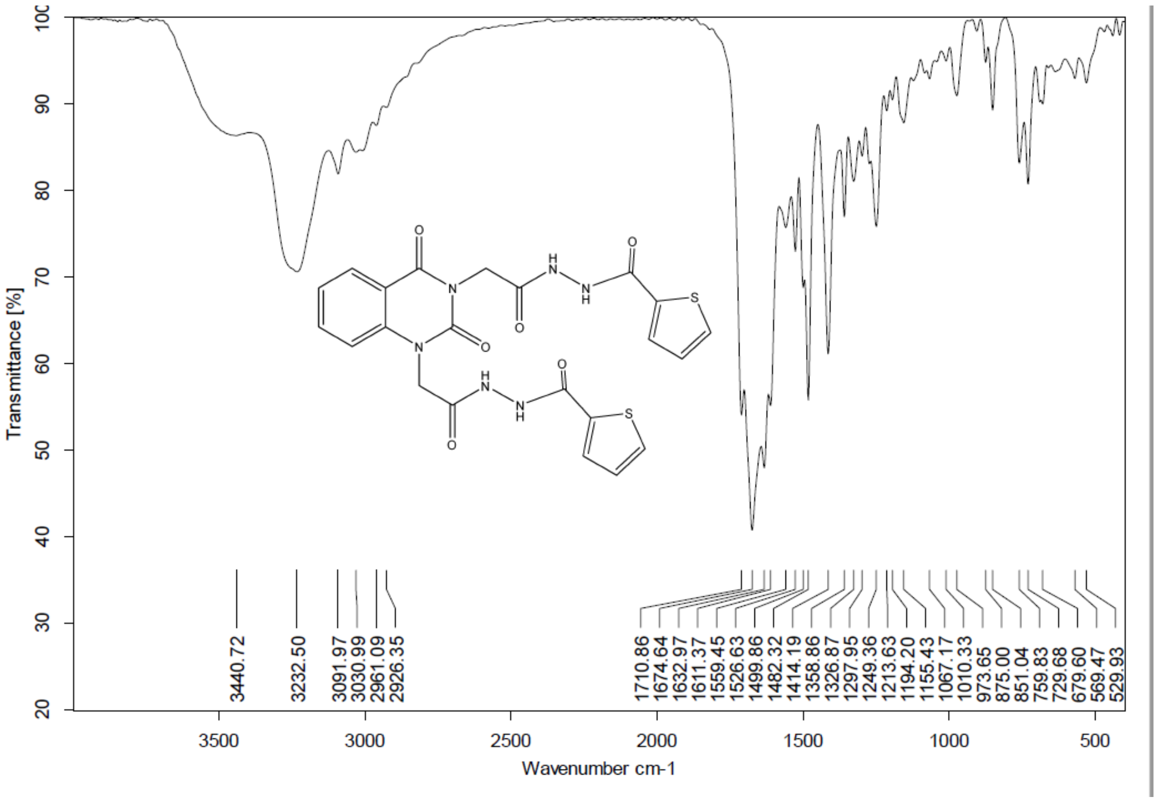

FT-IR of compound (6d)

-

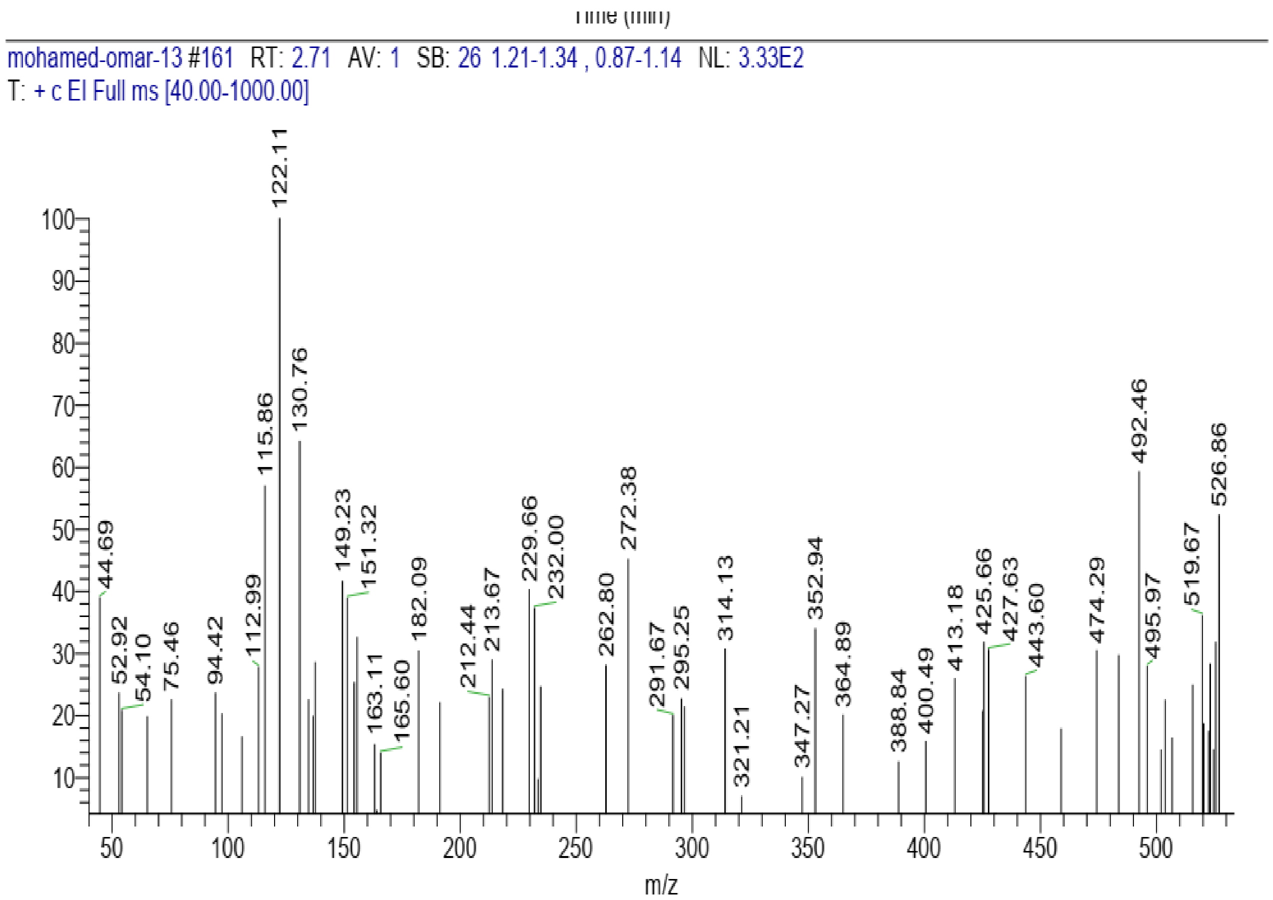

m/z Intensity Relative

MS of compound (6d)

-

-Compound (6e)

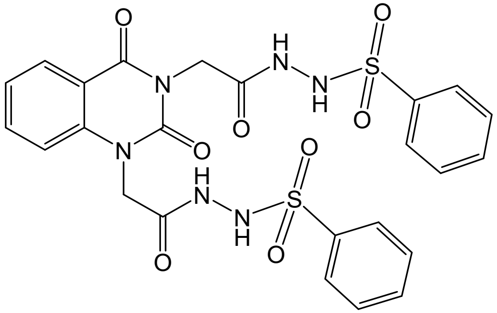

6e

-

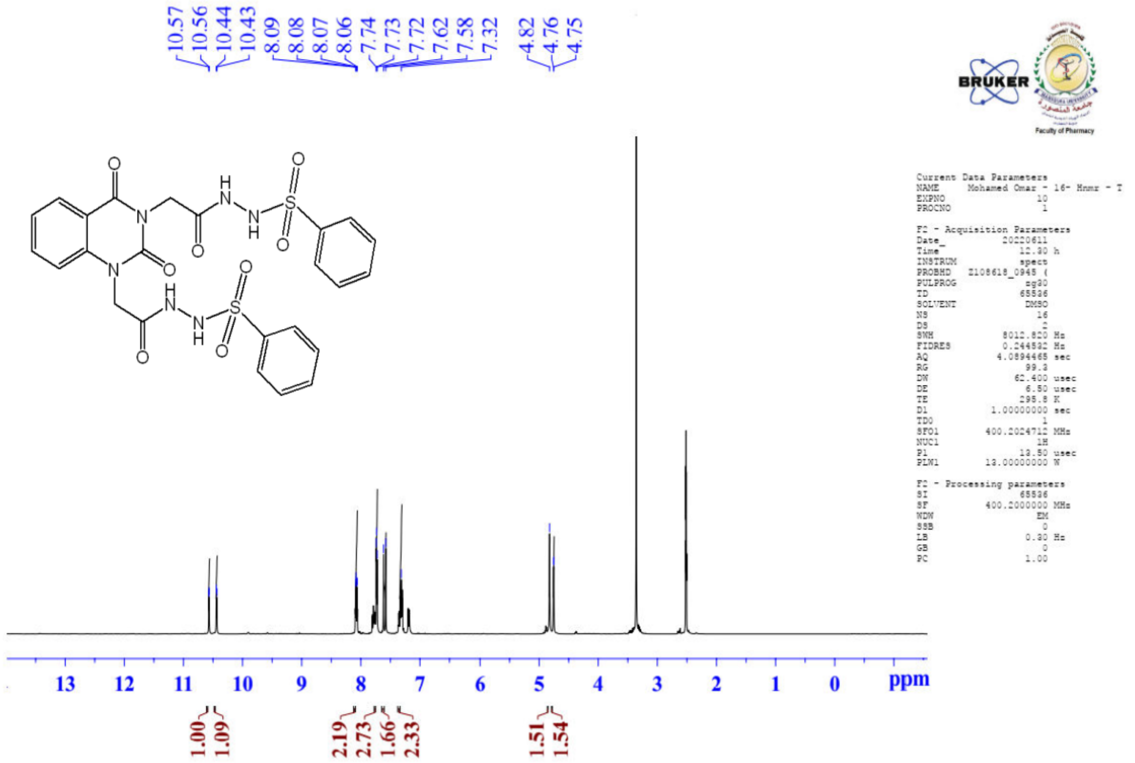

^{1}H-NMR (400 MHz, DMSO-d_6) of compound (6e)

-

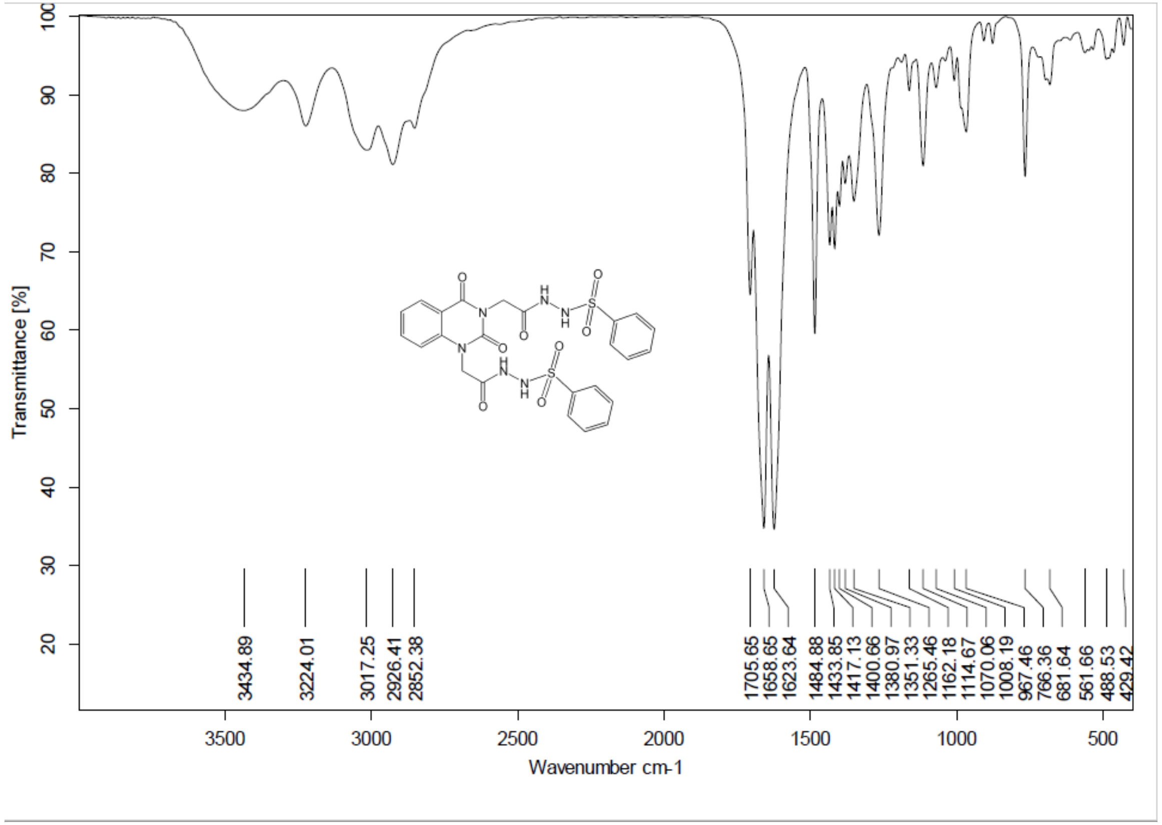

FT-IR of compound (6e)

-

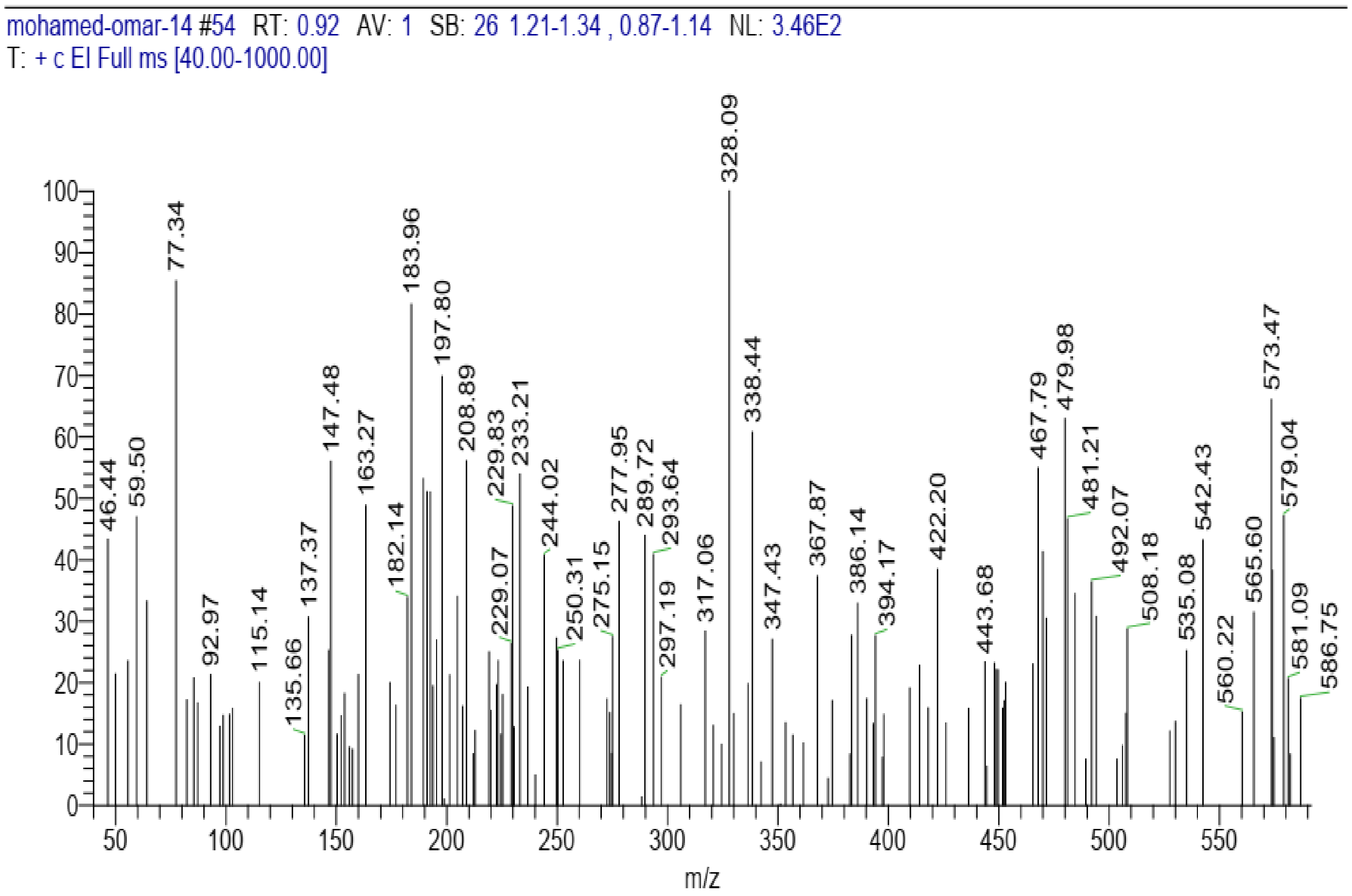

m/z Intensity Relative

MS of compound (6e)

-

-Compound (7a)

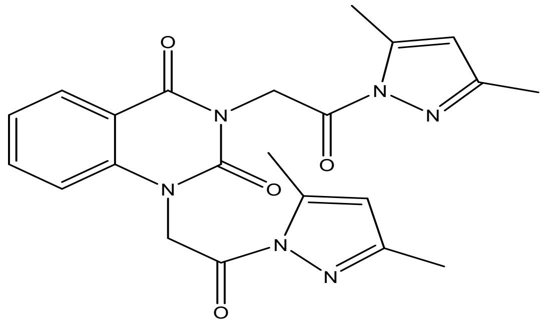

7a

-

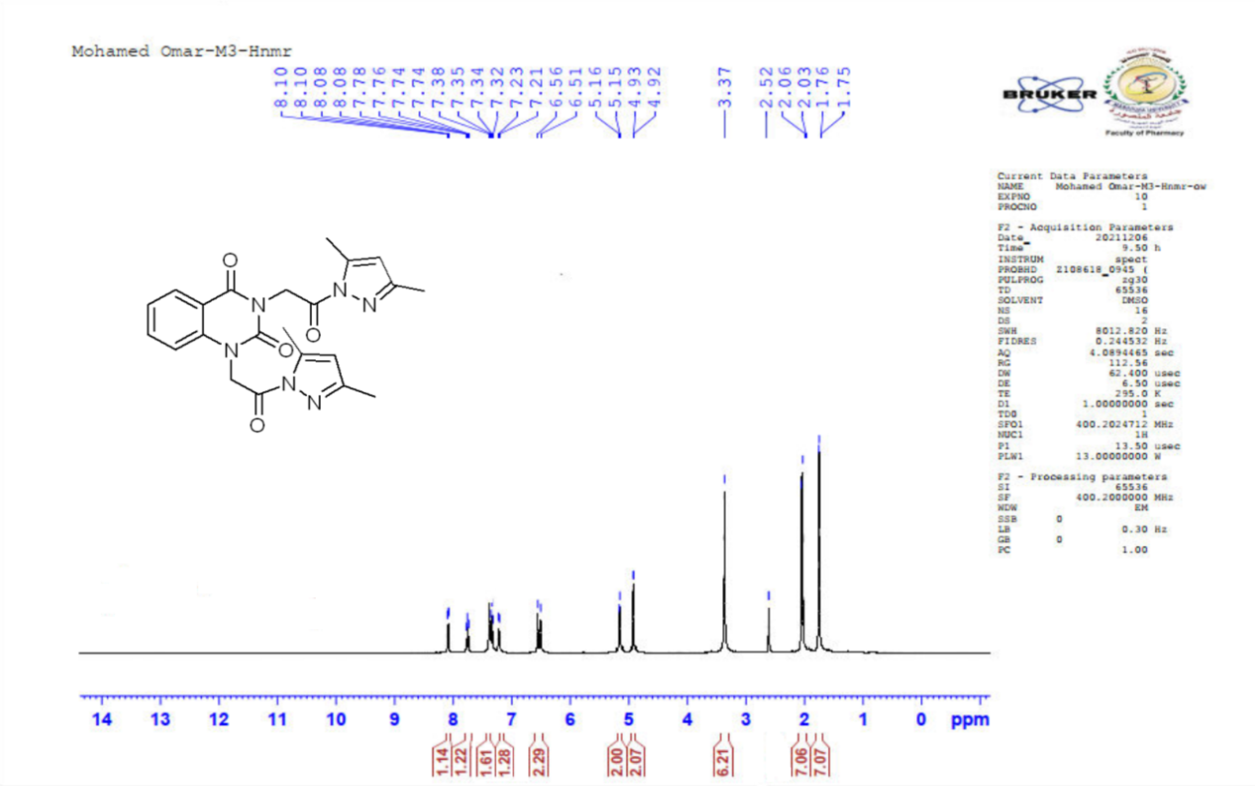

^{1}H-NMR (400 MHz, DMSO-d$_6$) of compound (7a)

-

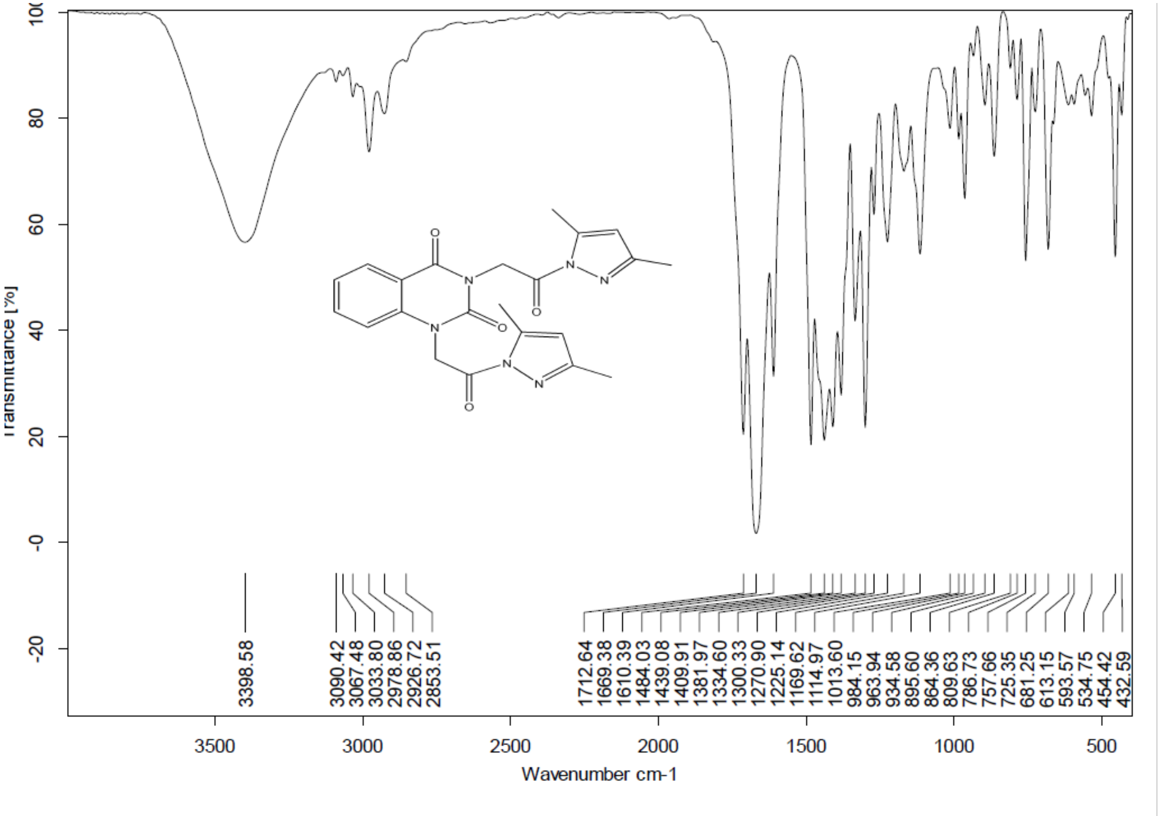

FT-IR of compound (7a)

-

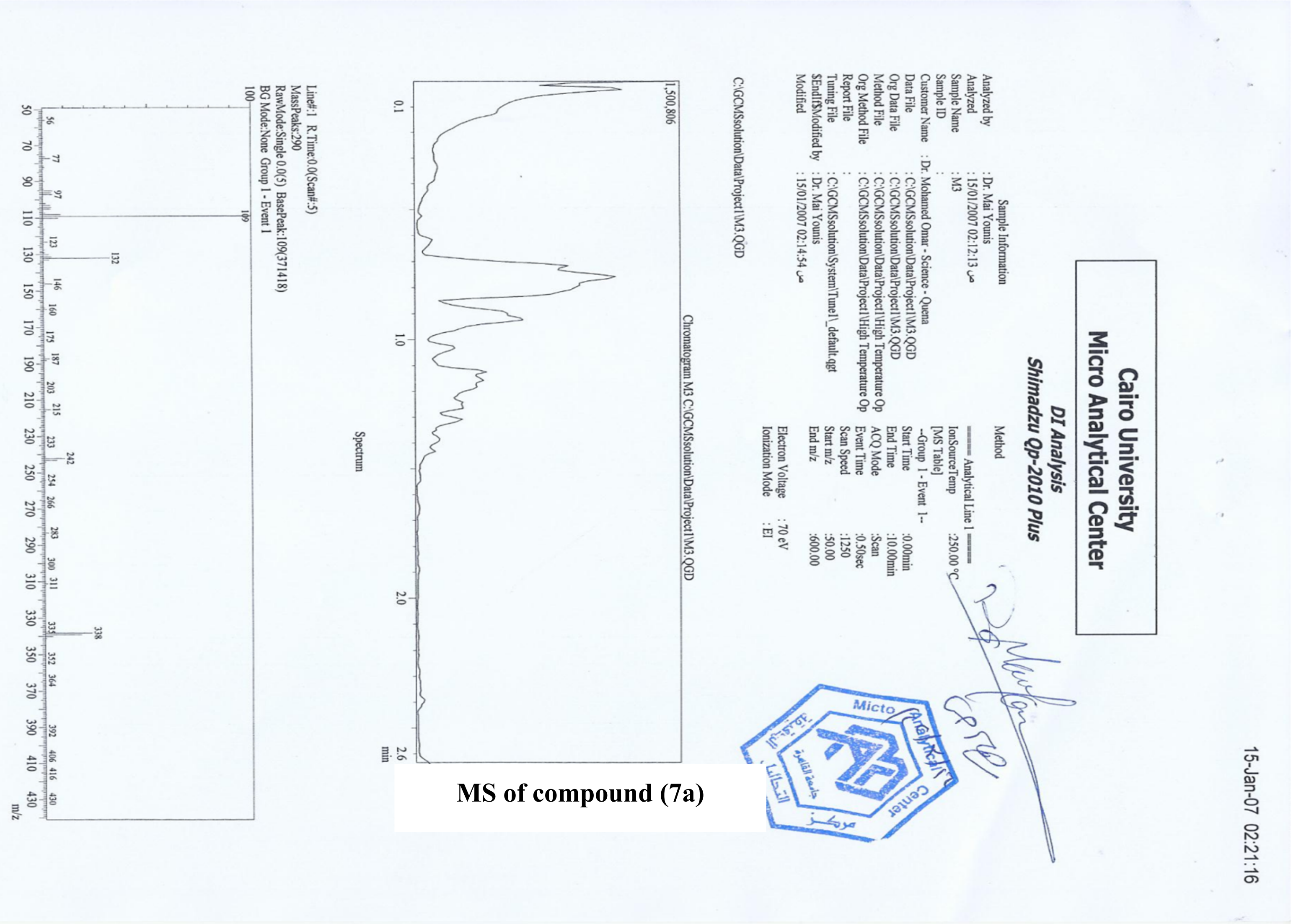

MS of compound (7a)

-

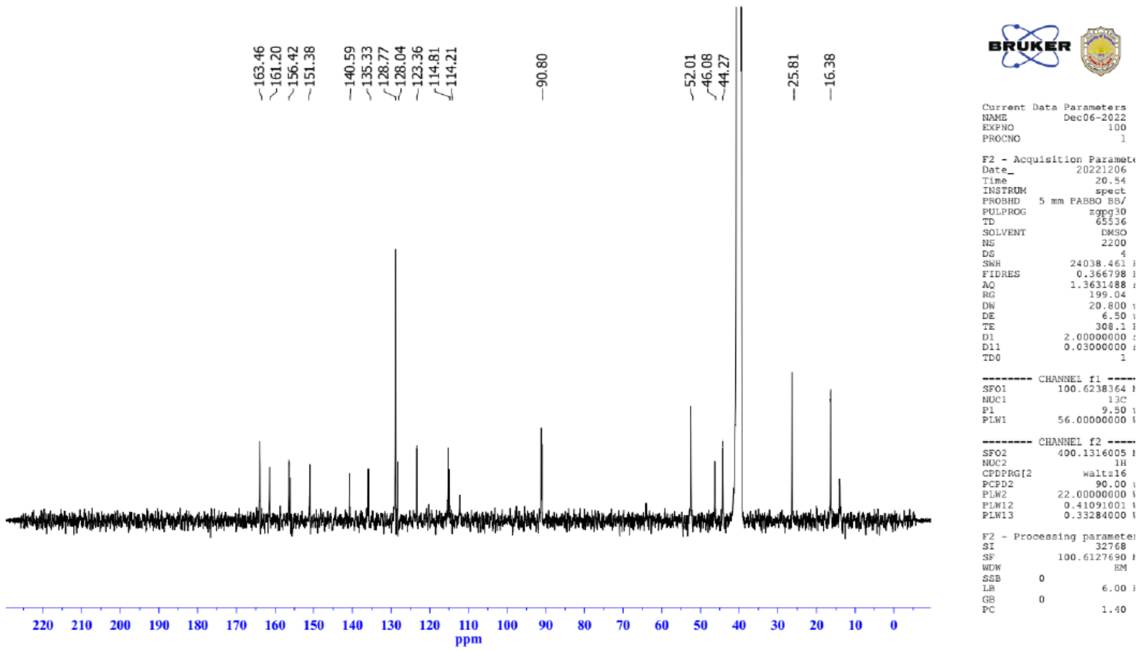

^{13}C-NMR (100 MHz, DMSO) of compound (7a)

-

Compound (7b)

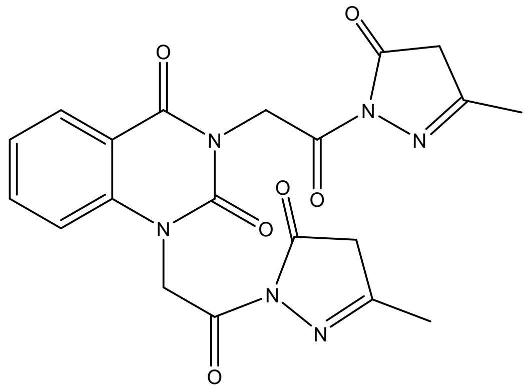

7b

-

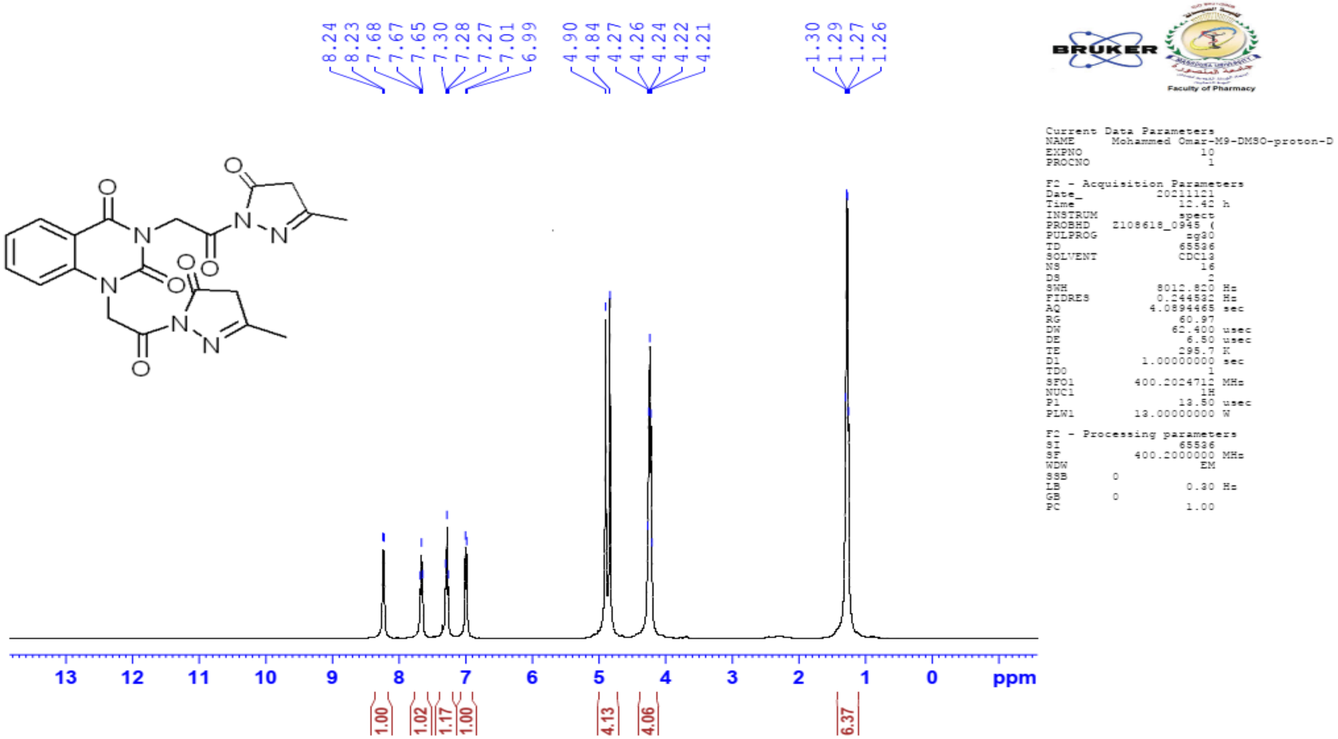

8.24 8.23 7.68 7.67 7.65 7.30 7.28 7.27 7.01 6.99
4.90 4.84 4.27 4.26 4.24 4.22 4.21
1.30 1.29 1.27 1.26
BRUKER
Faculty of Pharmacy
Current Data Parameters
NAME Mohammed Omar-M9-DMSO-proton-D
EXPNO 10
PROCNO 1
F2 - Acquisition Parameters
Date_ 20211121
Time 12.42 h
INSTRUM spect
PROBHD Z108618_0945 (
PULPROG zg30
TD 65536
SOLVENT CDCl3
NS 16
DS 2
SWH 8012.820 Hz
FIDRES 0.244532 Hz
AQ 4.0894465 sec
RG 60.97
DW 62.400 usec
DE 6.50 usec
TE 295.7 K
D1 1.00000000 sec
TD0 1
SFO1 400.2024712 MHz
NUC1 1H
P1 13.50 usec
PLW1 13.00000000 W
F2 - Processing parameters
SI 65536
SF 400.2000000 MHz
WDW EM
SSB 0
LB 0.30 Hz
GB 0
PC 1.00
13 12 11 10 9 8 7 6 5 4 3 2 1 0 ppm
1.00 1.02 1.17 1.00 4.13 4.06 6.37

-

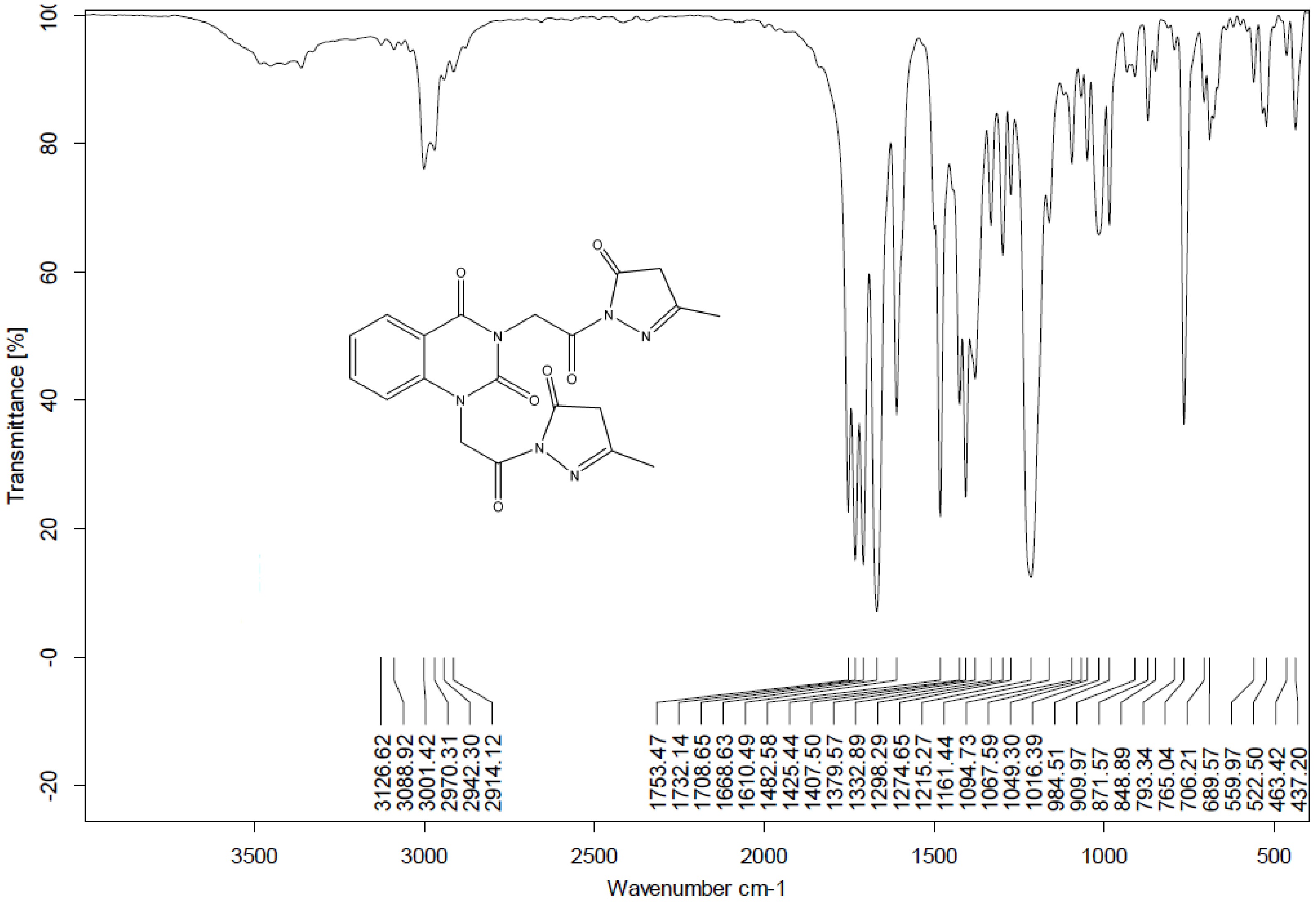

FT-IR of compound (7b)

-

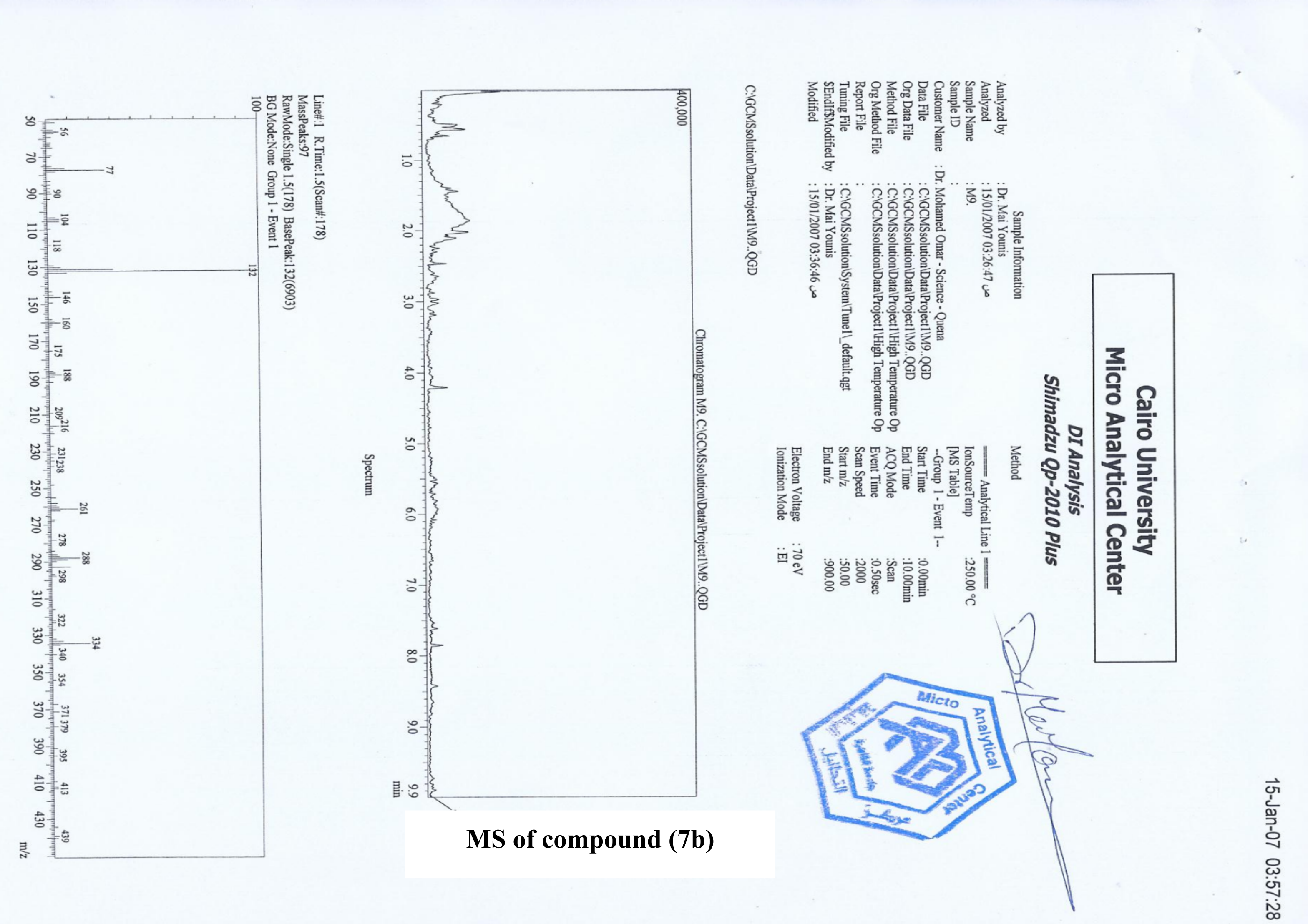

MS of compound (7b)

-

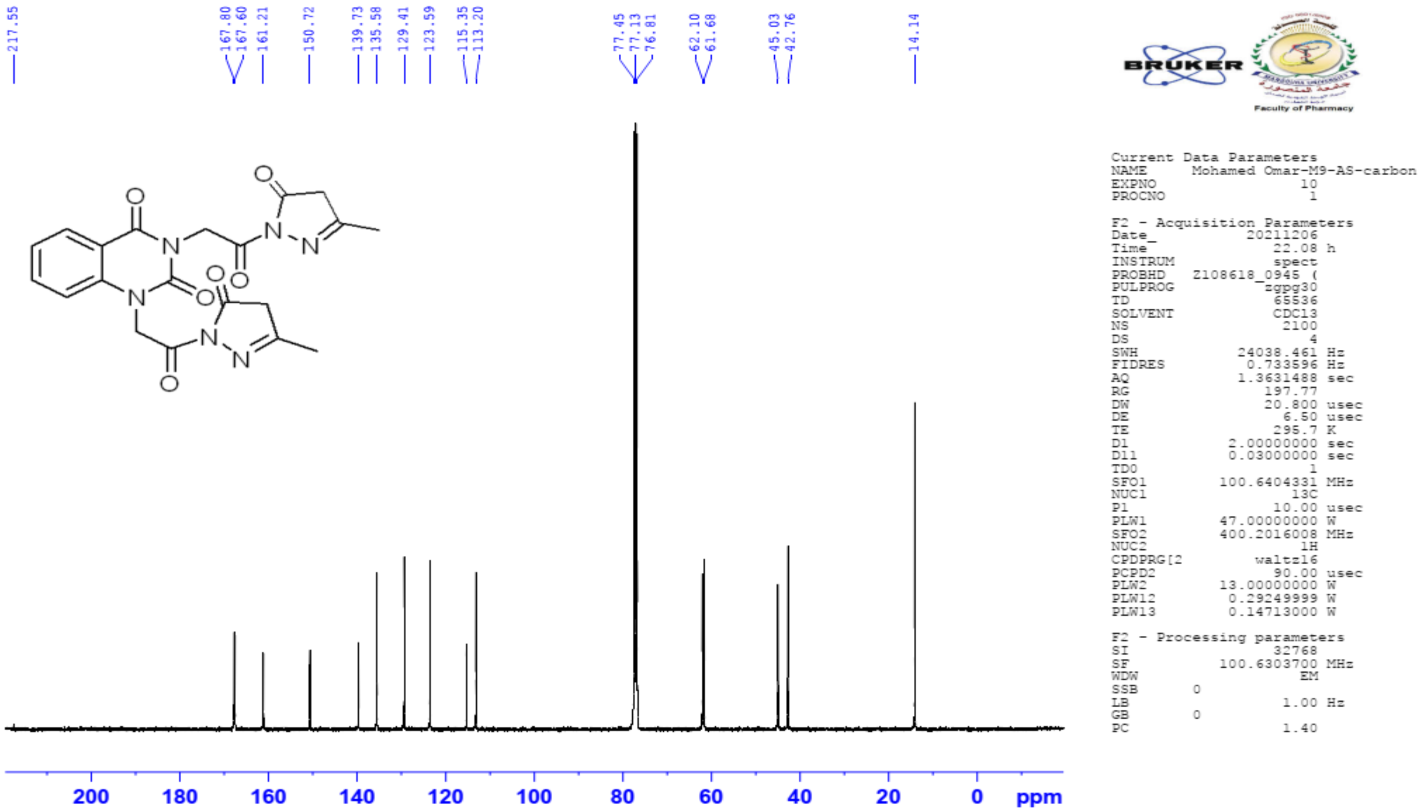

^{13}C-NMR (100 MHz, $CDCl_3$) of compound (7b)

-

-Compound (8)

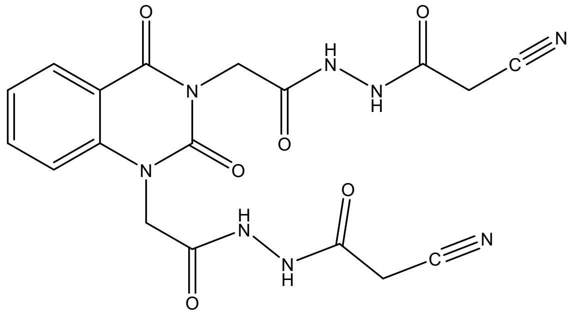

8

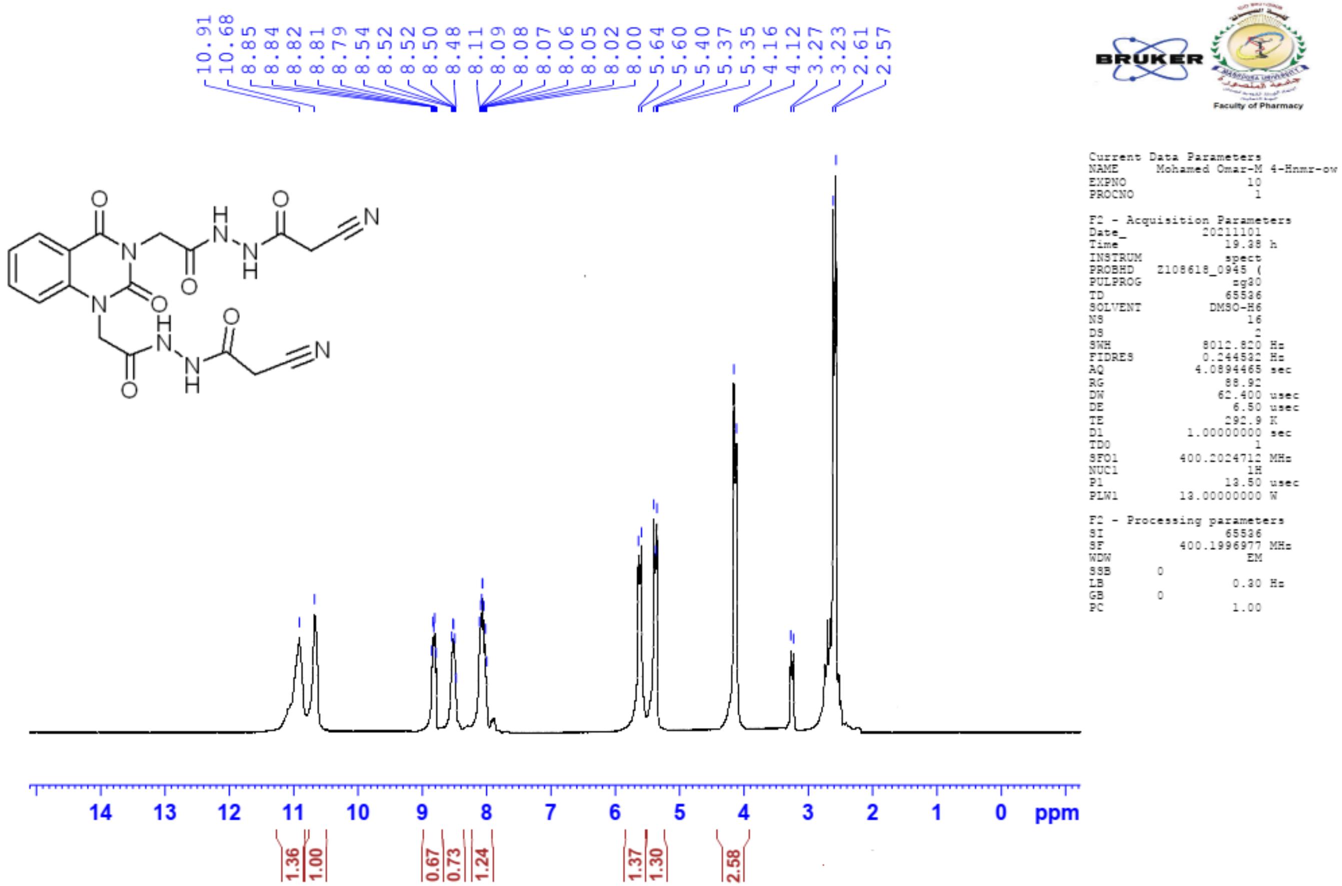

^{1}H-NMR (400 MHz, DMSO-d_6) of compound (8)

-

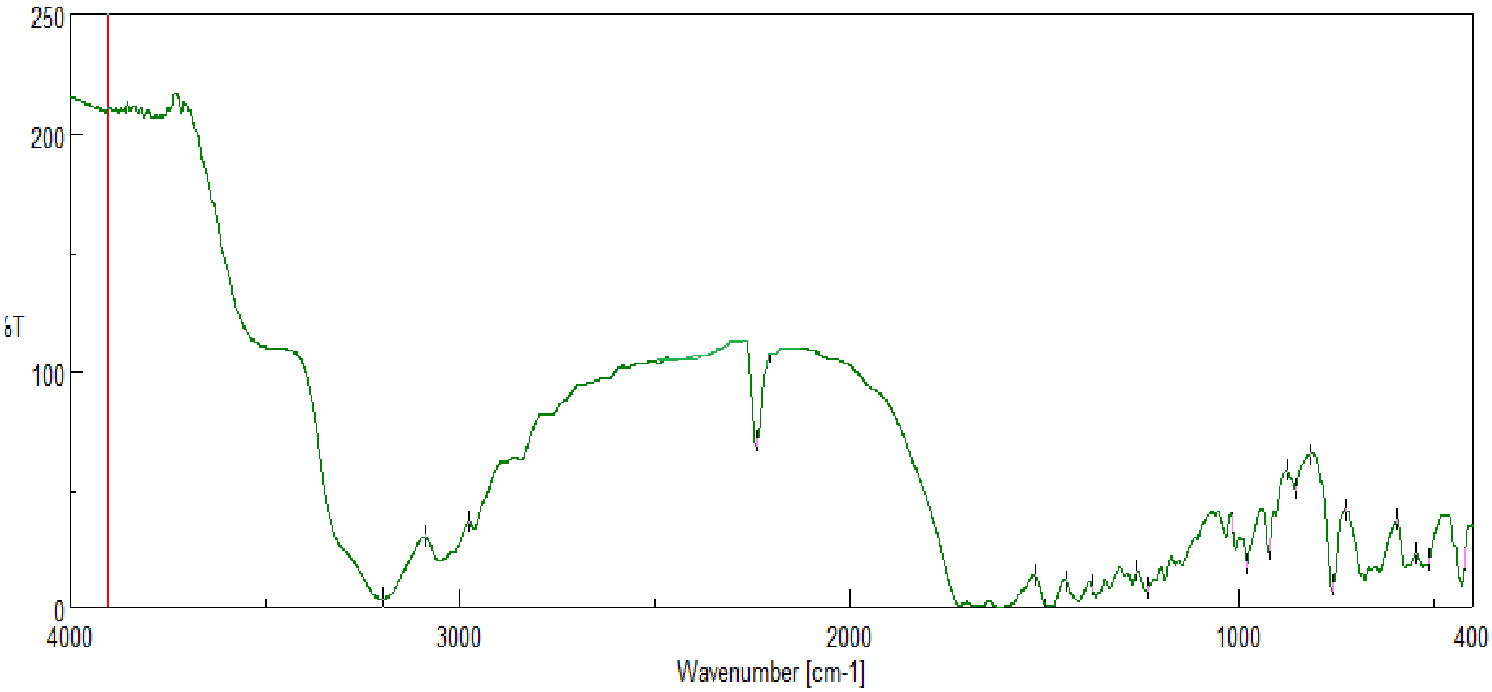

FT-IR of compound (8)

-

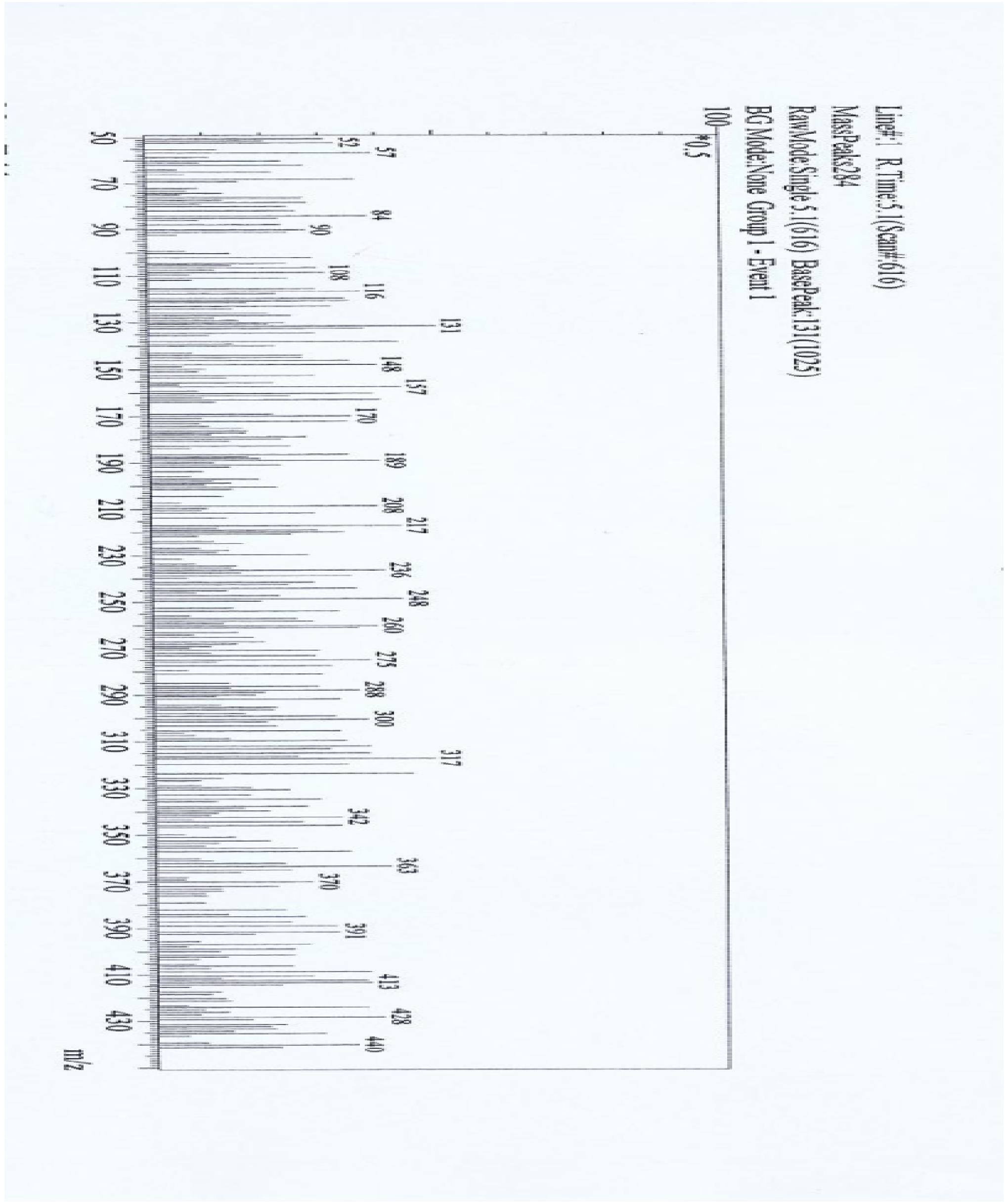

MS of compound (8)

-

-Compound (9a):-

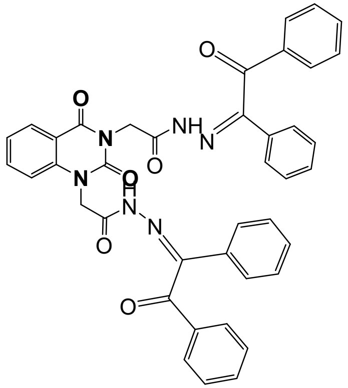

9a

-

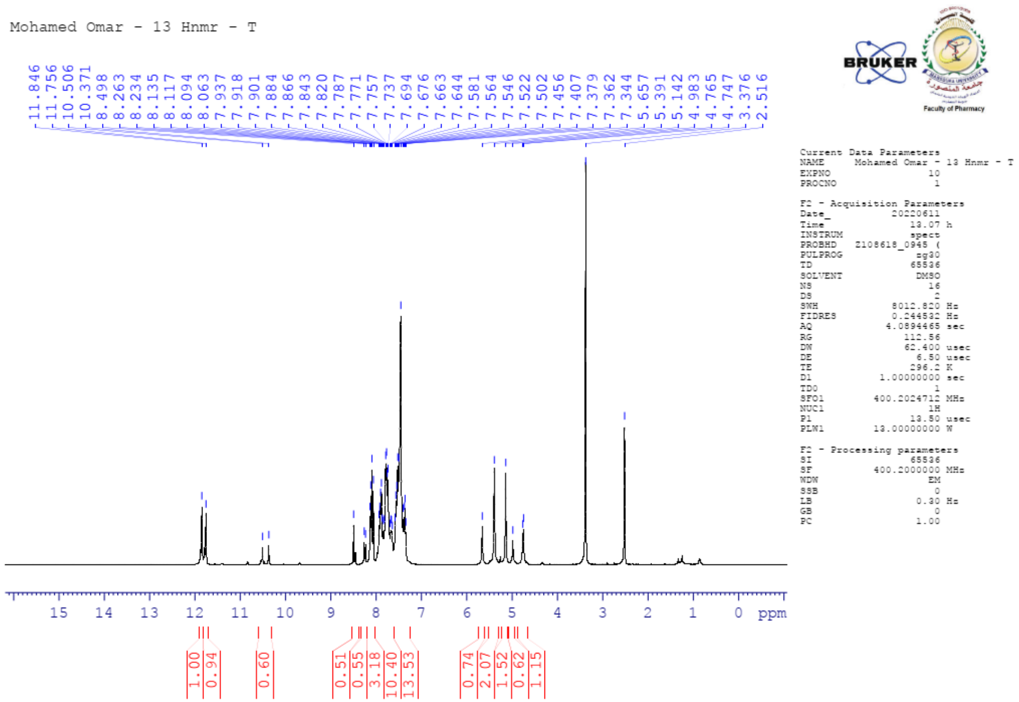

^{1}H-NMR (400 MHz, DMSO-d_6) of compound (9a)

-

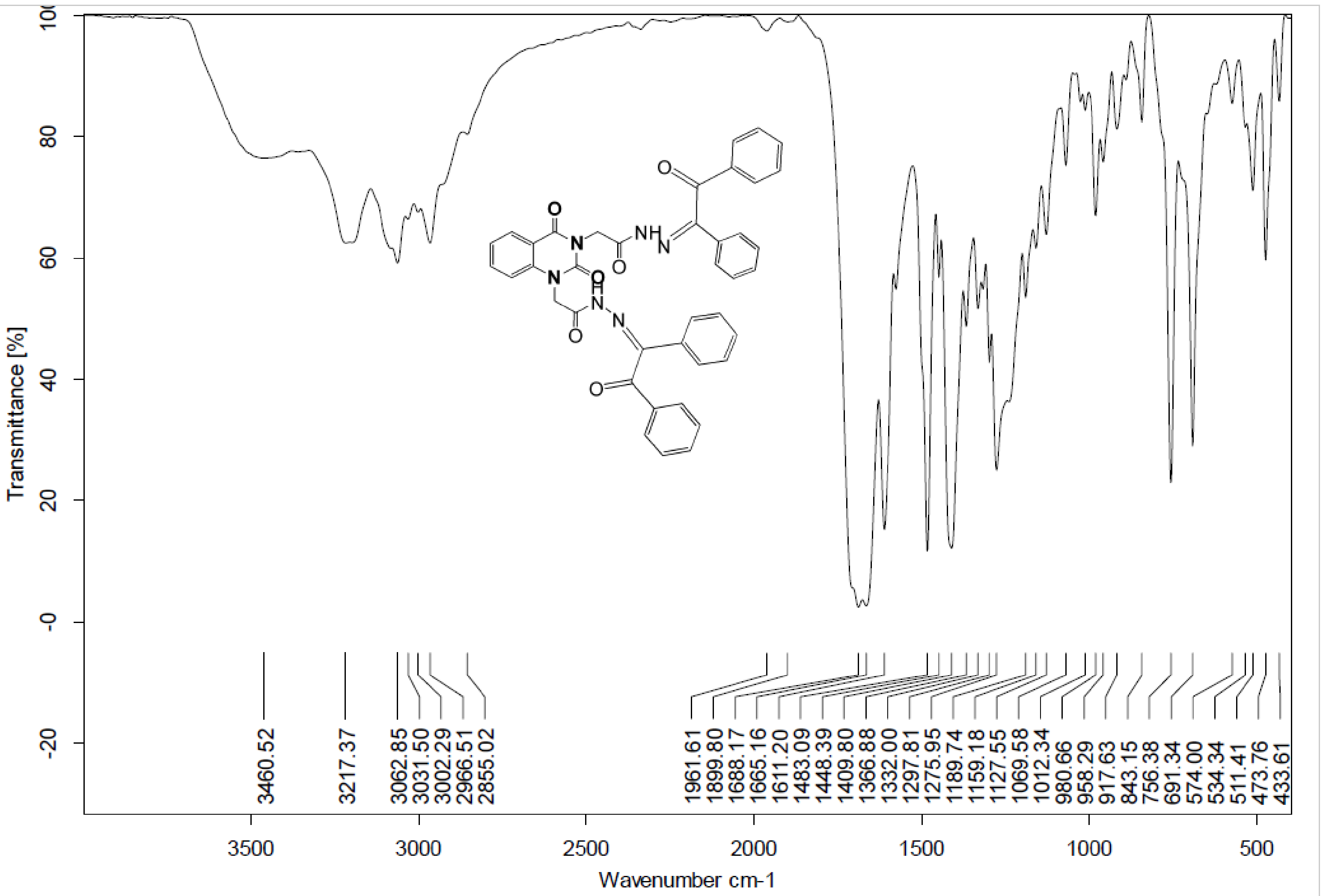

FT-IR of compound (9a)

-

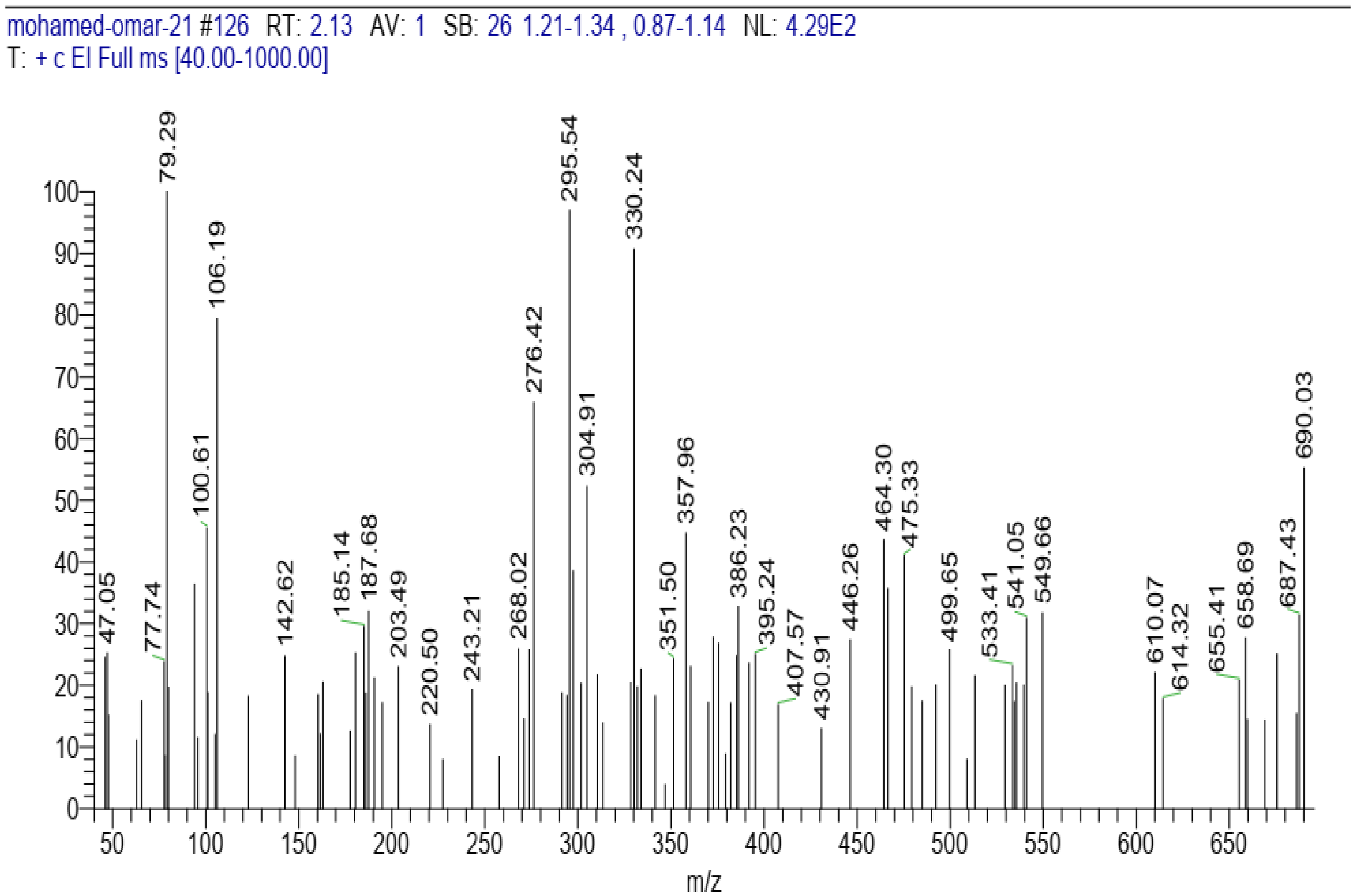

m/z Intensity Relative

MS of compound (9a)

-

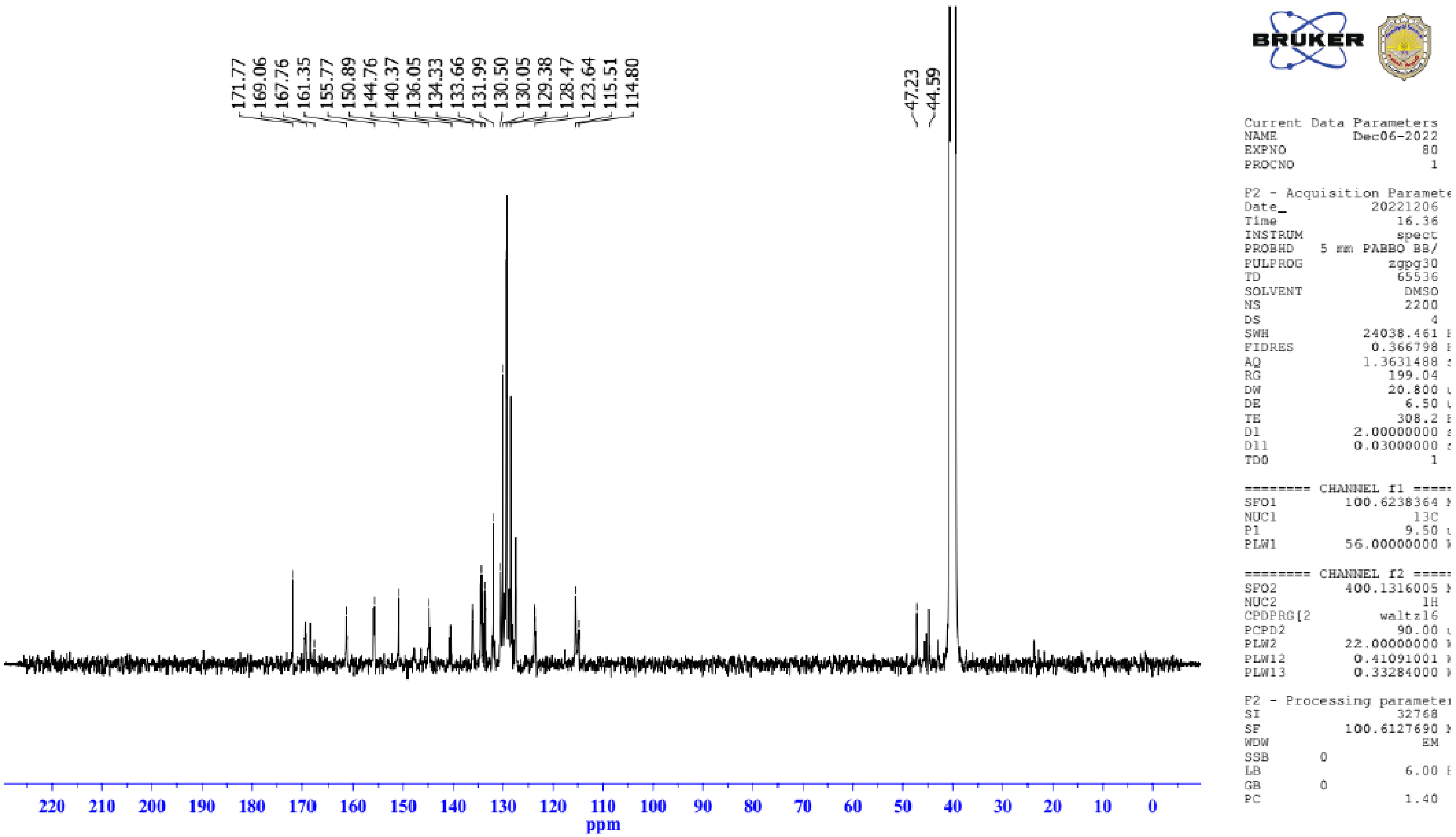

^{13}C-NMR (100 MHz, DMSO) of compound (9a)

-

Compounds Code, InChI, and SMILES

Compound code	InChI	SMILES
**1**	InChI=1S/C16H18N2O6/c1-3-23-13(19)9-17-12-8-6-5-7-11(12)15(21)18(16(17)22)10-14(20)24-4-2/h5-8H,3-4,9-10H2,1-2H3	O=C(C1=CC=CC=C1N2CC(OCC)=O)N(CC(OCC)=O)C2=O
**2**	InChI=1S/C12H14N6O4/c13-15-9(19)5-17-8-4-2-1-3-7(8)11(21)18(12(17)22)6-10(20)16-14/h1-4H,5-6,13-14H2,(H,15,19)(H,16,20)	O=C(N2CC(NN)=O)N(CC(NN)=O)C1=CC=CC=C1C2=O
**3a**	InChI=1S/C26H22N6O4/c33-23(29-27-15-19-9-3-1-4-10-19)17-31-22-14-8-7-13-21(22)25(35)32(26(31)36)18-24(34)30-28-16-20-11-5-2-6-12-20/h1-16H,17-18H2,(H,29,33)(H,30,34)/b27-15+,28-16+	O=C(C1=CC=CC=C1N2CC(N/N=C/C4=CC=CC=C4)=O)N(CC(N/N=C/C3=CC=CC=C3)=O)C2=O
**3b**	InChI=1S/C26H20Cl2N6O4/c27-19-9-5-17(6-10-19)13-29-31-23(35)15-33-22-4-2-1-3-21(22)25(37)34(26(33)38)16-24(36)32-30-14-18-7-11-20(28)12-8-18/h1-14H,15-16H2,(H,31,35)(H,32,36)/b29-13+,30-14+	O=C(C1=CC=CC=C1N2CC(N/N=C/C4=CC=C(Cl)C=C4)=O)N(CC(N/N=C/C3=CC=C(Cl)C=C3)=O)C2=O
**3c**	InChI=1S/C26H22N6O6/c33-19-9-5-17(6-10-19)13-27-29-23(35)15-31-22-4-2-1-3-21(22)25(37)32(26(31)38)16-24(36)30-28-14-18-7-11-20(34)12-8-18/h1-14,33-34H,15-16H2,(H,29,35)(H,30,36)/b27-13+,28-14+	O=C(C1=CC=CC=C1N2CC(N/N=C/C4=CC=C(O)C=C4)=O)N(CC(N/N=C/C3=CC=C(O)C=C3)=O)C2=O
**3d**	InChI=1S/C26H20N8O8/c35-23(29-27-13-17-5-9-19(10-6-17)33(39)40)15-31-22-4-2-1-3-21(22)25(37)32(26(31)38)16-24(36)30-28-14-18-7-11-20(12-8-18)34(41)42/h1-14H,15-16H2,(H,29,35)(H,30,36)/b27-13+,28-14+	O=C(C1=CC=CC=C1N2CC(N/N=C/C4=CC=C([N+]([O-])=O)C=C4)=O)N(CC(N/N=C/C3=CC=C([N+]([O-])=O)C=C3)=O)C2=O
**3e**	InChI=1S/C22H18N6O6/c29-19(25-23-11-15-5-3-9-33-15)13-27-18-8-2-1-7-17(18)21(31)28(22(27)32)14-20(30)26-24-12-16-6-4-10-34-16/h1-12H,13-14H2,(H,25,29)(H,26,30)/b23-11+,24-12+	O=C(C1=CC=CC=C1N2CC(N/N=C/C4=CC=CO4)=O)N(CC(N/N=C/C3=CC=CO3)=O)C2=O
**4a**	InChI=1S/C22H26N6O4/c29-19(25-23-15-7-1-2-8-15)13-27-18-12-6-5-11-17(18)21(31)28(22(27)32)14-20(30)26-24-16-9-3-4-10-16/h5-6,11-12H,1-4,7-10,13-14H2,(H,25,29)(H,26,30)	O=C(C1=CC=CC=C1N2CC(N/N=C4/CCCC4)=O)N(CC(N/N=C3/CCCC3)=O)C2=O
**4b**	InChI=1S/C24H30N6O4/c31-21(27-25-17-9-3-1-4-10-17)15-29-20-14-8-7-13-19(20)23(33)30(24(29)34)16-22(32)28-26-18-11-5-2-6-12-18/h7-8,13-14H,1-6,9-12,15-16H2,(H,27,31)(H,28,32)	O=C(C1=CC=CC=C1N2CC(N/N=C4/CCCCC4)=O)N(CC(N/N=C3/CCCCC3)=O)C2=O
**4c**	InChI=1S/C28H26N6O4/c1-19(21-11-5-3-6-12-21)29-31-25(35)17-33-24-	O=C(C1=CC=CC=C1N2CC(N/N=C(C)/C4=CC=CC=C4)=O)N(CC(N/N=C(C)/C3

	16-10-9-15-23(24)27(37)34(28(33)38)18-26(36)32-30-20(2)22-13-7-4-8-14-22/h3-16H,17-18H2,1-2H3,(H,31,35)(H,32,36)/b29-19+,30-20+	=CC=CC=C3)=O)C2=O
**4d**	InChI=1S/C30H30N6O4/c1-19-9-13-23(14-10-19)21(3)31-33-27(37)17-35-26-8-6-5-7-25(26)29(39)36(30(35)40)18-28(38)34-32-22(4)24-15-11-20(2)12-16-24/h5-16H,17-18H2,1-4H3,(H,33,37)(H,34,38)/b31-21+,32-22+	O=C(C1=CC=CC=C1N2CC(N/N=C(C)/C4=CC=C(C)C=C4)=O)N(CC(N/N=C(C)/C3=CC=C(C)C=C3)=O)C2=O
**4e**	InChI=1S/C24H22N6O4S2/c1-15(19-9-5-11-35-19)25-27-21(31)13-29-18-8-4-3-7-17(18)23(33)30(24(29)34)14-22(32)28-26-16(2)20-10-6-12-36-20/h3-12H,13-14H2,1-2H3,(H,27,31)(H,28,32)/b25-15+,26-16+	O=C(C1=CC=CC=C1N2CC(N/N=C(C)/C4=CC=CS4)=O)N(CC(N/N=C(C)/C3=CC=CS3)=O)C2=O
**4f**	InChI=1S/C28H20N8O6/c37-21(31-33-23-15-7-1-4-10-18(15)29-25(23)39)13-35-20-12-6-3-9-17(20)27(41)36(28(35)42)14-22(38)32-34-24-16-8-2-5-11-19(16)30-26(24)40/h1-12H,13-14H2,(H,31,37)(H,32,38)(H,29,33,39)(H,30,34,40)	O=C(C1=CC=CC=C1N2CC(N/N=C4/C(C=CC=C5)=C5NC4=O)=O)N(CC(N/N=C3/C(C=CC=C6)=C6NC3=O)=O)C2=O
**5a**	InChI=1S/C28H18N6O8/c35-21(29-33-24(38)15-7-1-2-8-16(15)25(33)39)13-31-20-12-6-5-11-19(20)23(37)32(28(31)42)14-22(36)30-34-26(40)17-9-3-4-10-18(17)27(34)41/h1-12H,13-14H2,(H,29,35)(H,30,36)	O=C(C1=CC=CC=C1N2CC(NN4C(C3=CC=CC=C3C4=O)=O)=O)N(CC(NN6C(C5=CC=CC=C5C6=O)=O)=O)C2=O
**5b**	InChI=1S/C48H34N6O8/c55-33(49-53-44(58)39-35-23-11-1-2-12-24(23)36(40(39)45(53)59)26-14-4-3-13-25(26)35)21-51-32-20-10-9-19-31(32)43(57)52(48(51)62)22-34(56)50-54-46(60)41-37-27-15-5-6-16-28(27)38(42(41)47(54)61)30-18-8-7-17-29(30)37/h1-20,35-42H,21-22H2,(H,49,55)(H,50,56)	O=C(C1=CC=CC=C1N2CC(NN3C(C(C7C5=CC=CC=C5C6C8=C7C=CC=C8)C6C3=O)=O)=O)N(CC(NN4C(C(C%11C9=CC=CC=C9C%10C%12=C%11C=CC=C%12)C%10C4=O)=O)=O)C2=O
**5c**	InChI=1S/C28H26N6O8/c35-21(29-33-24(38)15-7-1-2-8-16(15)25(33)39)13-31-20-12-6-5-11-19(20)23(37)32(28(31)42)14-22(36)30-34-26(40)17-9-3-4-10-18(17)27(34)41/h1-6,11-12,15-18H,7-10,13-14H2,(H,29,35)(H,30,36)	O=C(C1=CC=CC=C1N2CC(NN3C(C(CC=CC6)C6C3=O)=O)=O)N(CC(NN4C(C(CC=CC5)C5C4=O)=O)=O)C2=O
**6a**	InChI=1S/C16H18N6O6/c1-9(23)17-19-13(25)7-21-12-6-4-3-5-11(12)15(27)22(16(21)28)8-14(26)20-18-10(2)24/h3-6H,7-8H2,1-2H3,(H,17,23)(H,18,24)(H,19,25)(H,20,26)	O=C(C1=CC=CC=C1N2CC(NNC(C)=O)=O)N(CC(NNC(C)=O)=O)C2=O
**6b**	InChI=1S/C16H16Cl2N6O6/c17-5-11(25)19-21-13(27)7-23-10-4-2-1-3-9(10)15(29)24(16(23)30)8-14(28)22-20-12(26)6-18/h1-4H,5-8H2,(H,19,25)(H,20,26)(H,21,27)(H,22,28)	O=C(C1=CC=CC=C1N2CC(NNC(CCl)=O)=O)N(CC(NNC(CCl)=O)=O)C2=O
**6c**	InChI=1S/C26H22N6O6/c33-21(27-29-23(35)17-9-3-1-4-10-17)15-31-20-14-8-7-13-19(20)25(37)32(26(31)38)16-22(34)28-30-24(36)18-11-5-2-6-12-18/h1-14H,15-16H2,(H,27,33)(H,28,34)(H,29,35)(H,30,36)	O=C(C1=CC=CC=C1N2CC(NNC(C3=CC=CC=C3)=O)=O)N(CC(NNC(C4=CC=CC=C4)=O)=O)C2=O
**6d**	InChI=1S/C22H18N6O6S2/c29-17(23-25-19(31)15-7-3-9-35-15)11-27-14-	O=C(C1=CC=CC=C1N2CC(NNC(C3=CC=CS3)=O)=O)N(CC(NNC(C4=CC=CS

	6-2-1-5-13(14)21(33)28(22(27)34)12-18(30)24-26-20(32)16-8-4-10-36-16/h1-10H,11-12H2,(H,23,29)(H,24,30)(H,25,31)(H,26,32)	4)=O)=O)C2=O
**7a**	InChI=1S/C22H22N6O4/c1-13-9-15(3)27(23-13)19(29)11-25-18-8-6-5-7-17(18)21(31)26(22(25)32)12-20(30)28-16(4)10-14(2)24-28/h5-10H,11-12H2,1-4H3	O=C(C1=CC=CC=C1N2CC(N4N=C(C)C=C4C)=O)N(CC(N3N=C(C)C=C3C)=O)C2=O
**7b**	InChI=1S/C20H18N6O6/c1-11-7-15(27)25(21-11)17(29)9-23-14-6-4-3-5-13(14)19(31)24(20(23)32)10-18(30)26-16(28)8-12(2)22-26/h3-6H,7-10H2,1-2H3	O=C(C1=CC=CC=C1N2CC(N4N=C(C)CC4=O)=O)N(CC(N3N=C(C)CC3=O)=O)C2=O
**8**	InChI=1S/C18H16N8O6/c19-7-5-13(27)21-23-15(29)9-25-12-4-2-1-3-11(12)17(31)26(18(25)32)10-16(30)24-22-14(28)6-8-20/h1-4H,5-6,9-10H2,(H,21,27)(H,22,28)(H,23,29)(H,24,30)	O=C(C1=CC=CC=C1N2CC(NNC(CC#N)=O)=O)N(CC(NNC(CC#N)=O)=O)C2=O
**9a**	InChI=1S/C40H30N6O6.CH4/c47-33(41-43-35(27-15-5-1-6-16-27)37(49)29-19-9-3-10-20-29)25-45-32-24-14-13-23-31(32)39(51)46(40(45)52)26-34(48)42-44-36(28-17-7-2-8-18-28)38(50)30-21-11-4-12-22-30;/h1-24H,25-26H2,(H,41,47)(H,42,48);1H4/b43-35-,44-36-;	O=C(N2CC(N/N=C(C(C5=CC=CC=C5)=O)/C3=CC=CC=C3)=O)N(CC(NN=C(C(C6=CC=CC=C6)=O)C4=CC=CC=C4)=O)C1=CC=CC=C1C2=O

-

The solubility of compounds in water, acidic and/or basic medium

Solubility / Sample	Cold water	Hot water	Acidic medium	Basic medium
**1**	+ve	+ve	+ve	+ve
**2**	+ve	+ve	+ve	+ve
**3a**	-ve	-ve	-ve	-ve
**3b**	-ve	-ve	-ve	-ve
**3c**	-ve	-ve	-ve	-ve
**3d**	-ve	-ve	-ve	-ve
**3e**	-ve	-ve	-ve	-ve
**4a**	-ve	-ve	-ve	-ve
**4b**	-ve	-ve	-ve	-ve
**4c**	-ve	-ve	-ve	-ve
**4d**	-ve	-ve	-ve	-ve
**4e**	-ve	-ve	-ve	-ve
**4f**	-ve	-ve	-ve	-ve
**5a**	-ve	-ve	-ve	+ve
**5b**	-ve	-ve	-ve	-ve
**5c**	-ve	-ve	-ve	-ve
**6a**	-ve	-ve	-ve	-ve

-

**6b**	-ve	-ve	-ve	-ve
**6c**	-ve	-ve	-ve	-ve
**6d**	-ve	-ve	-ve	-ve
**7a**	-ve	-ve	-ve	-ve
**7b**	-ve	-ve	-ve	-ve
**8**	-ve	-ve	-ve	-ve
**9a**	-ve	-ve	-ve	-ve

-

Supplementary data file

-Compound (1):

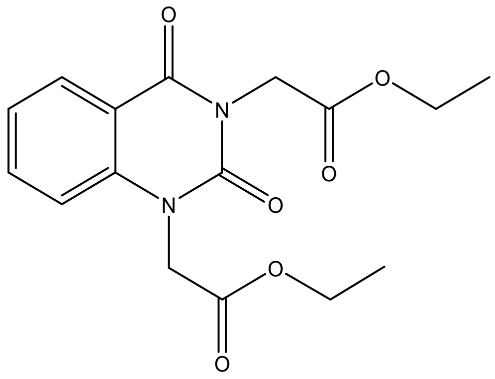

1

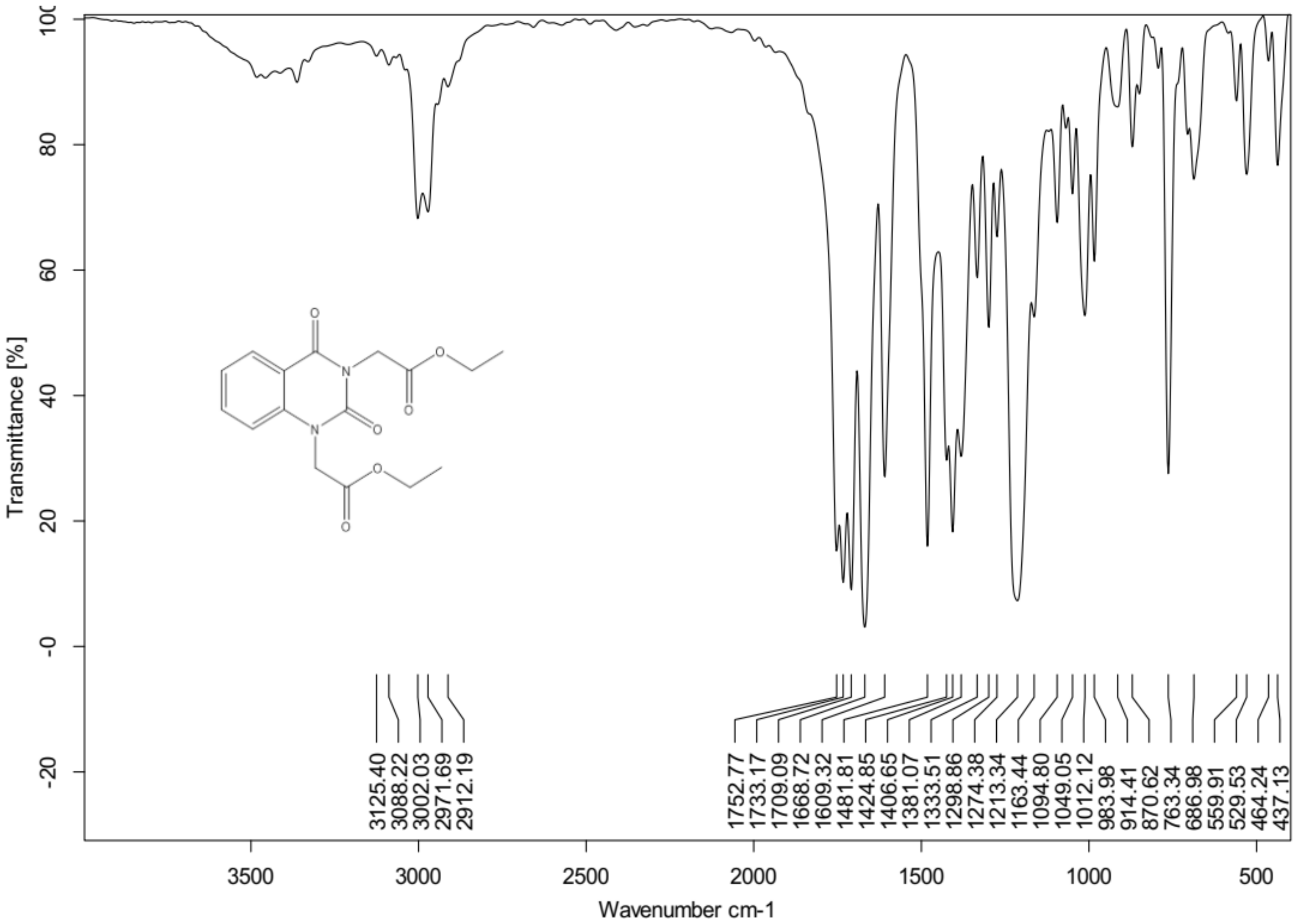

Figure . ^{1}H-NMR of compound 1 .

-

-

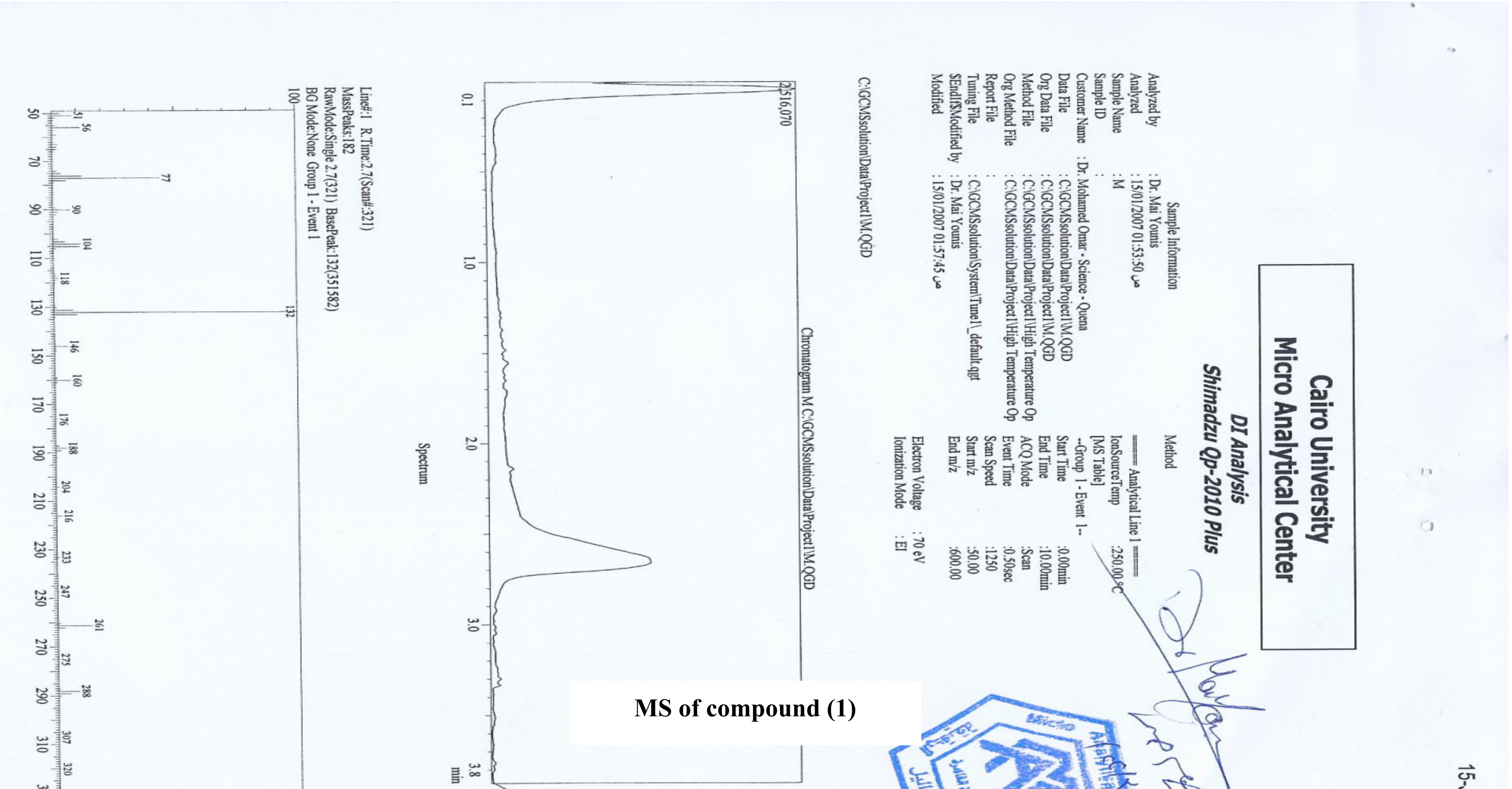

MS of compound (1)

-

-Compound (2):

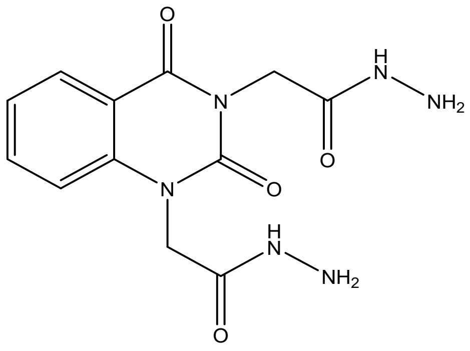

2

2

-

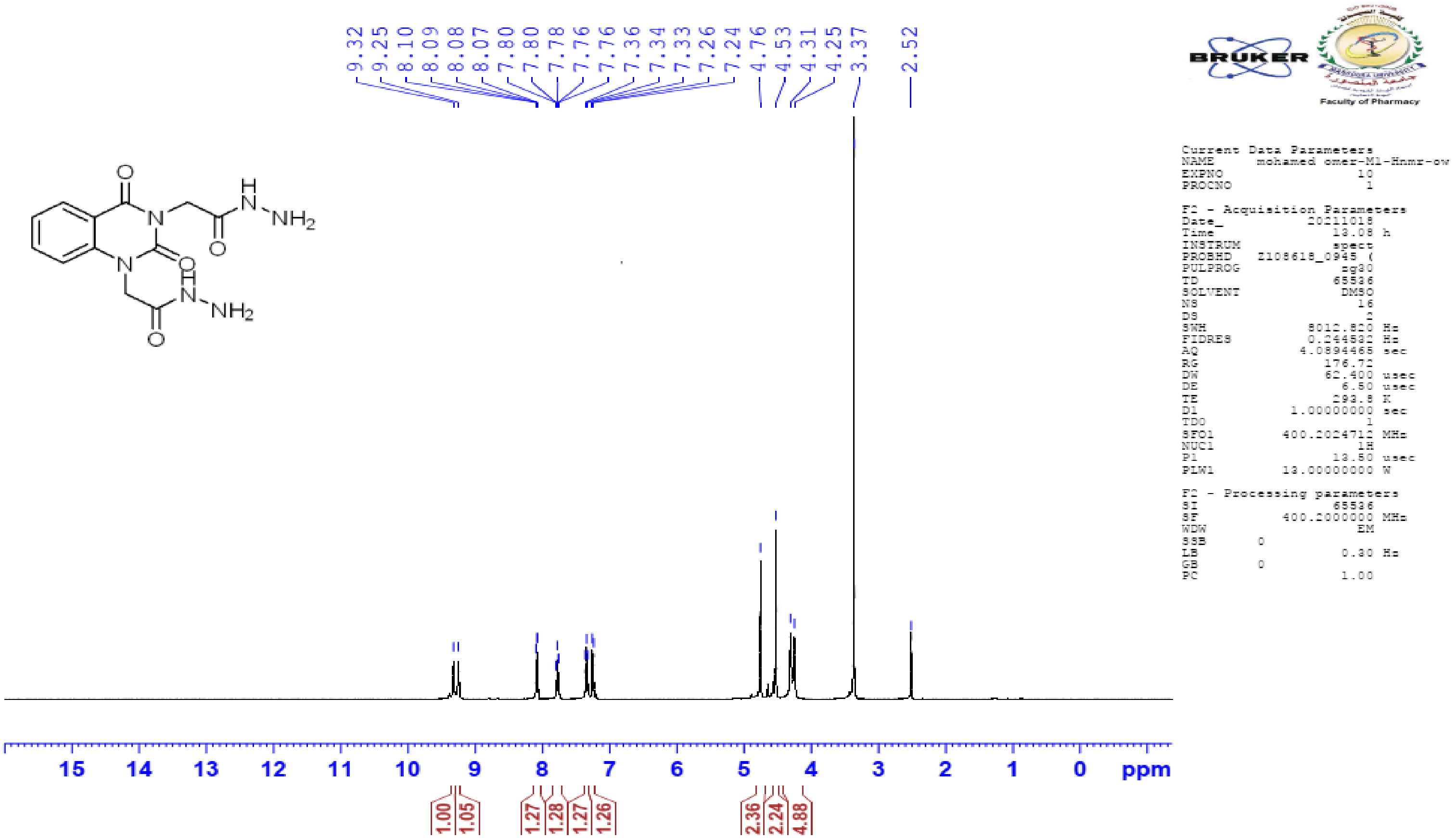

^{1}H-NMR of compound (2)

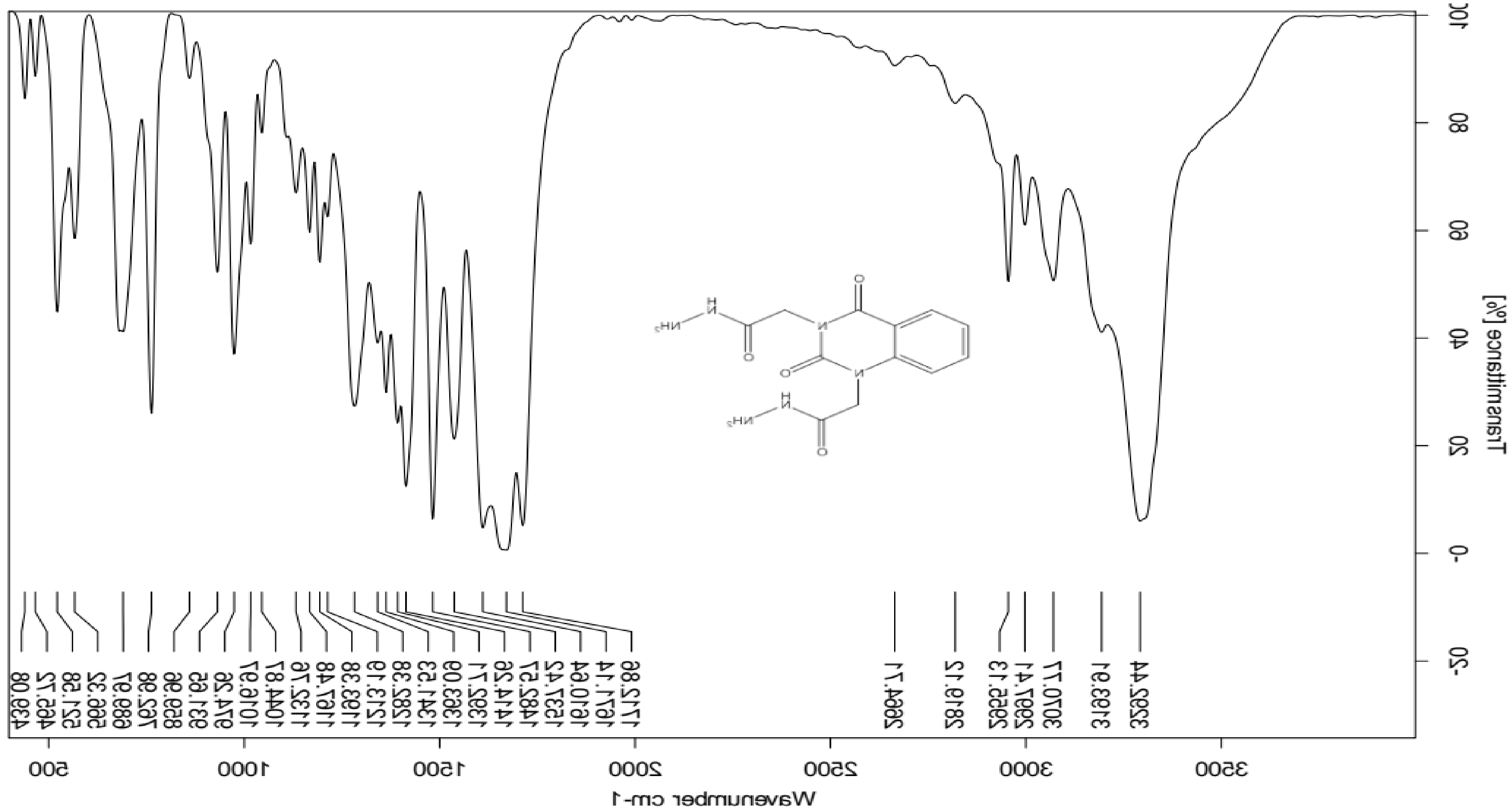

FT-IR of compound (2)

-

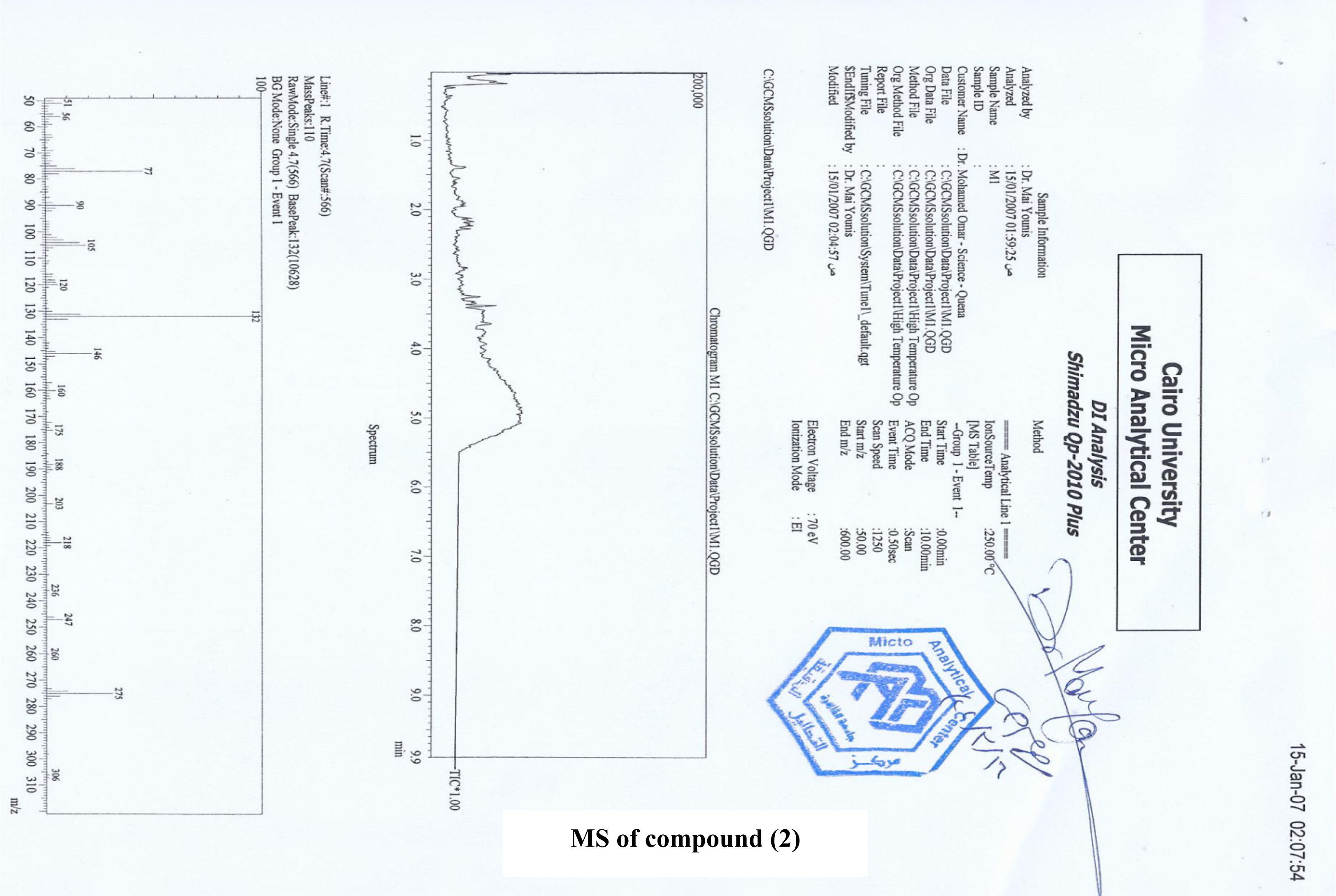

MS of compound (2)

-

-Compound (3a)

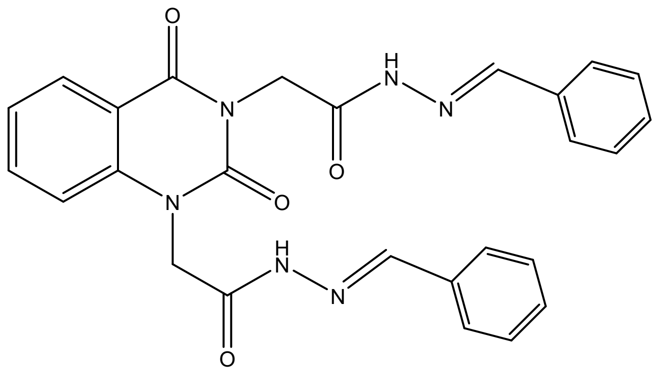

-

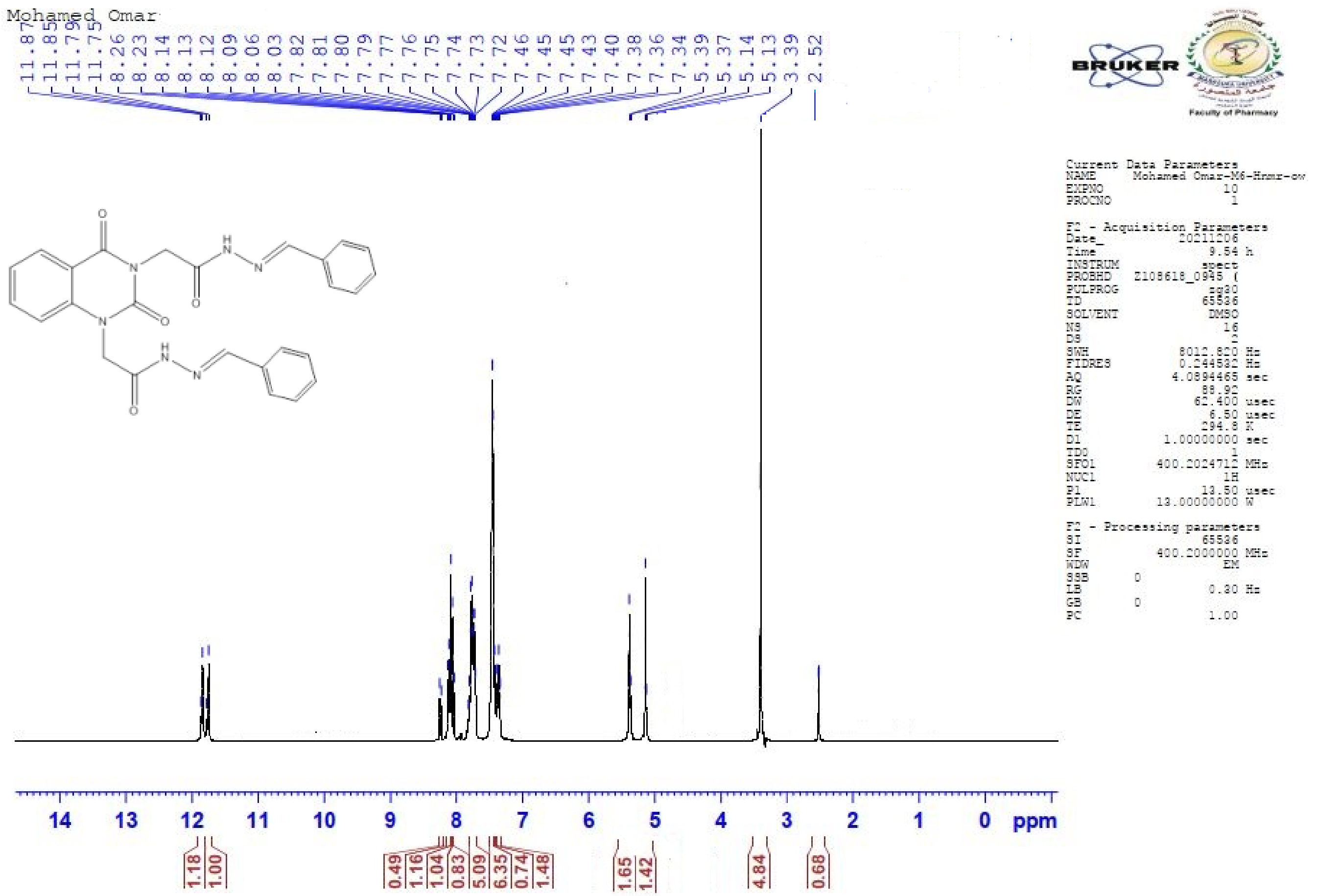

^{1}H-NMR of compound (3a)

-

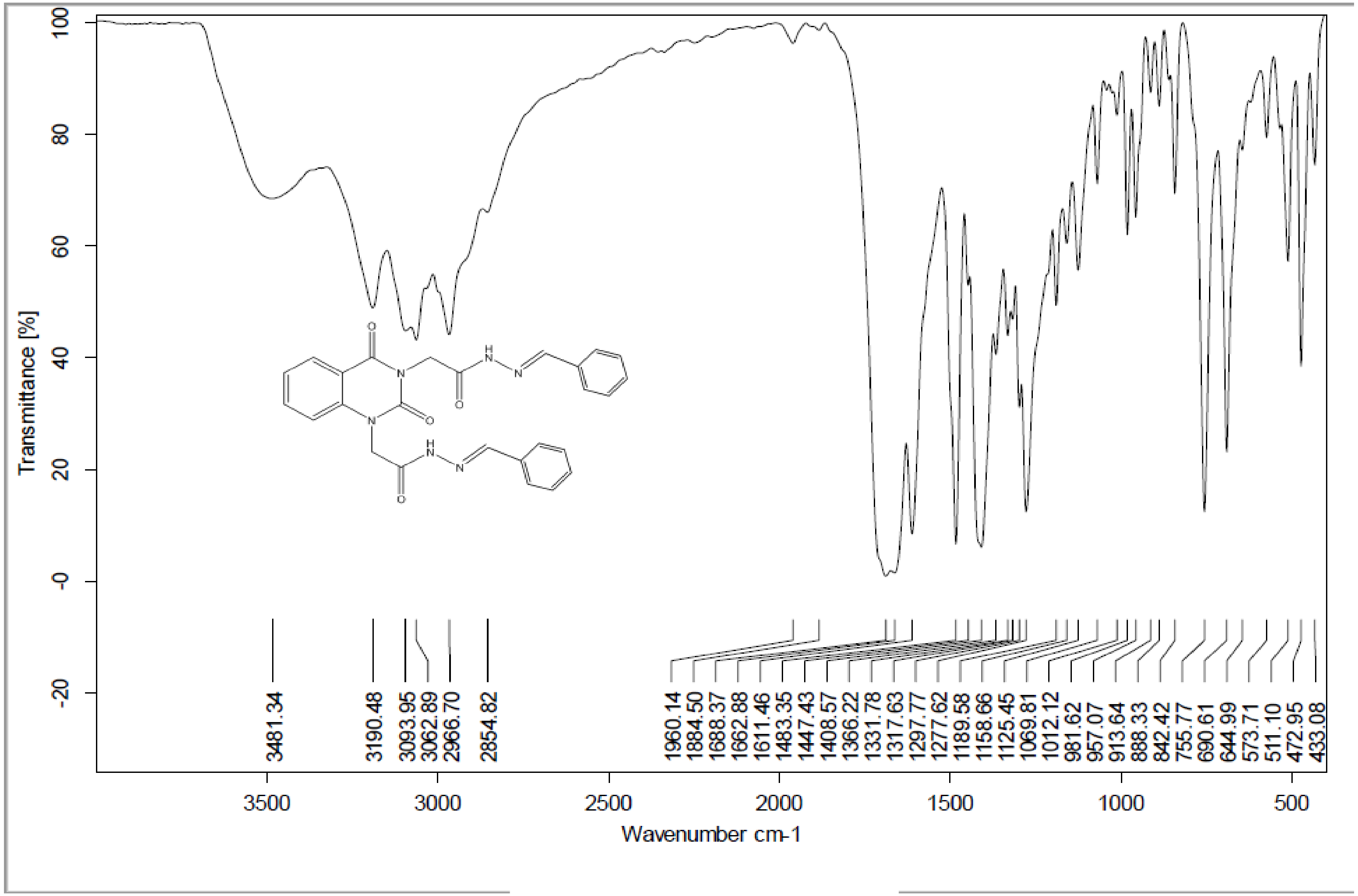

FT-IR of compound (3a)

-

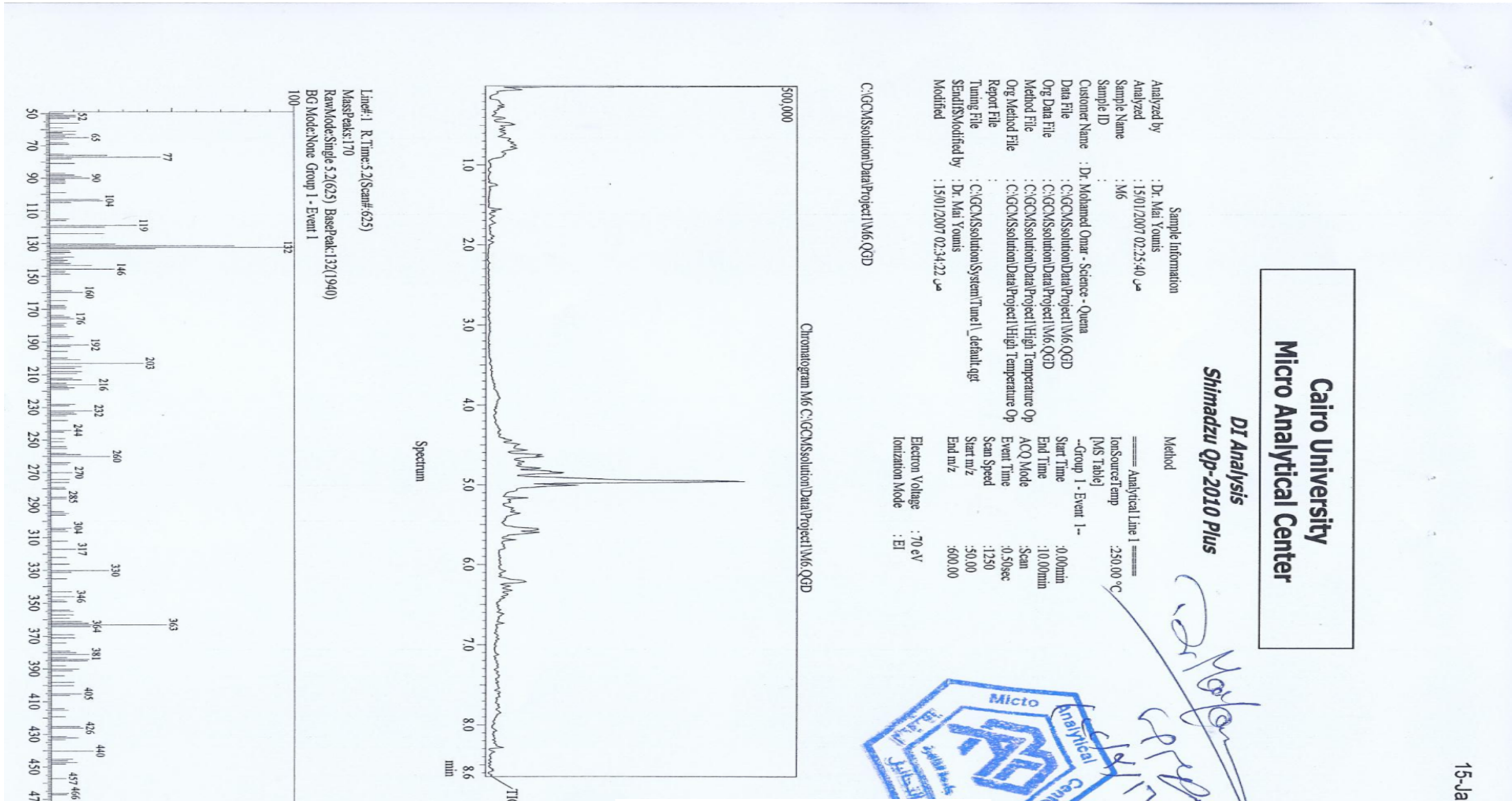

MS of compound (3a)

-

-Compound (3b): -

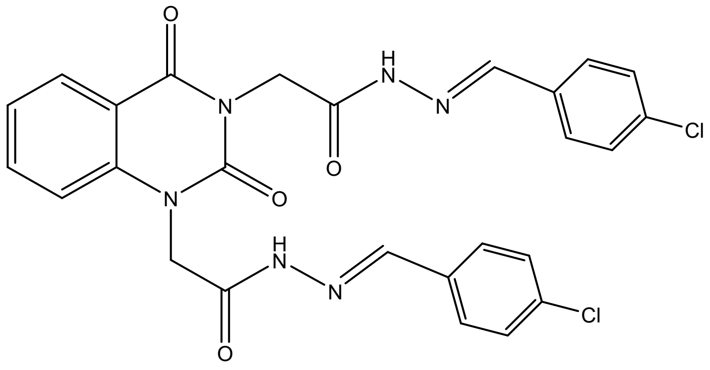

3b

-

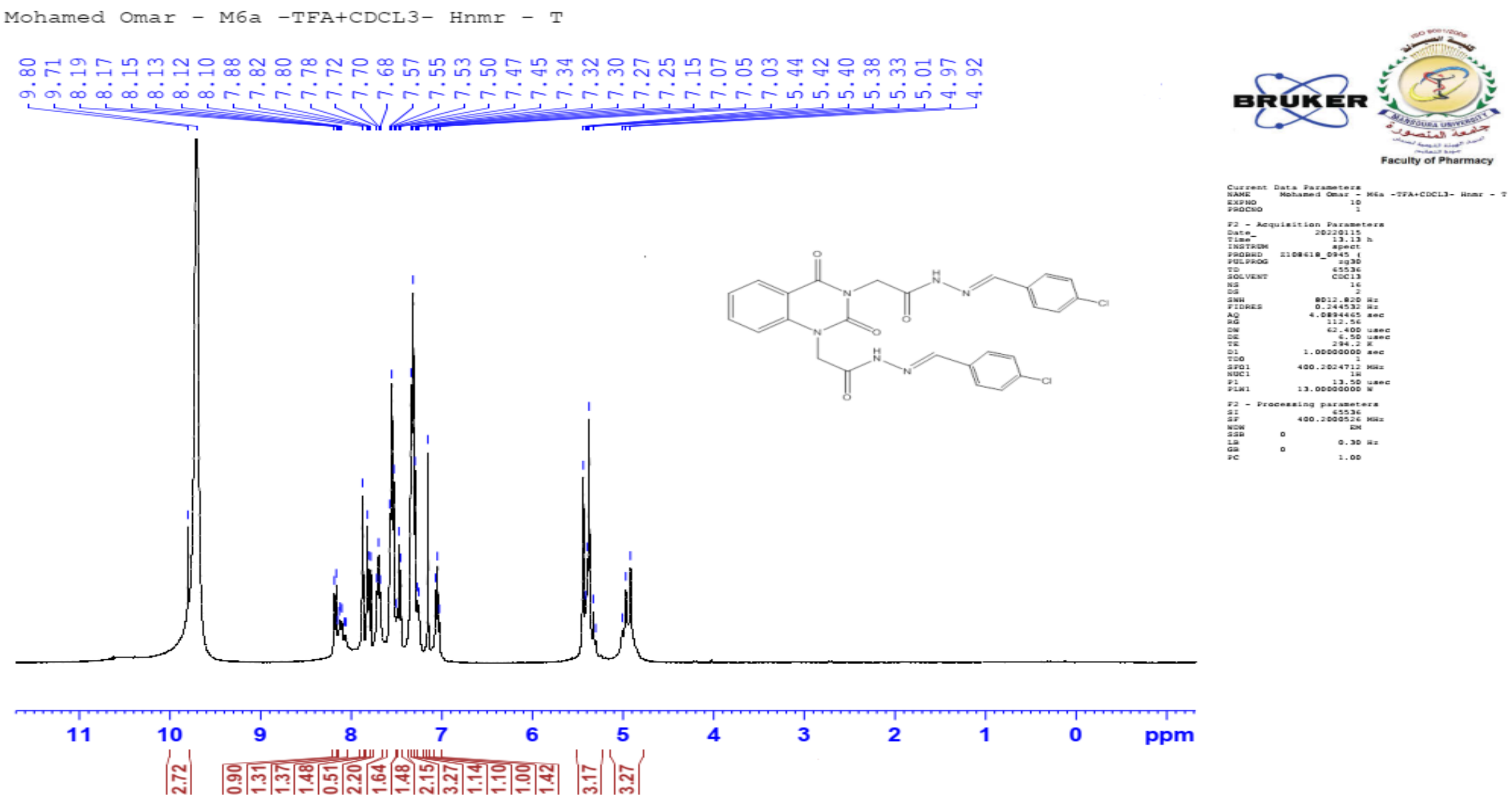

^{1}H-NMR of compound (3b)

-

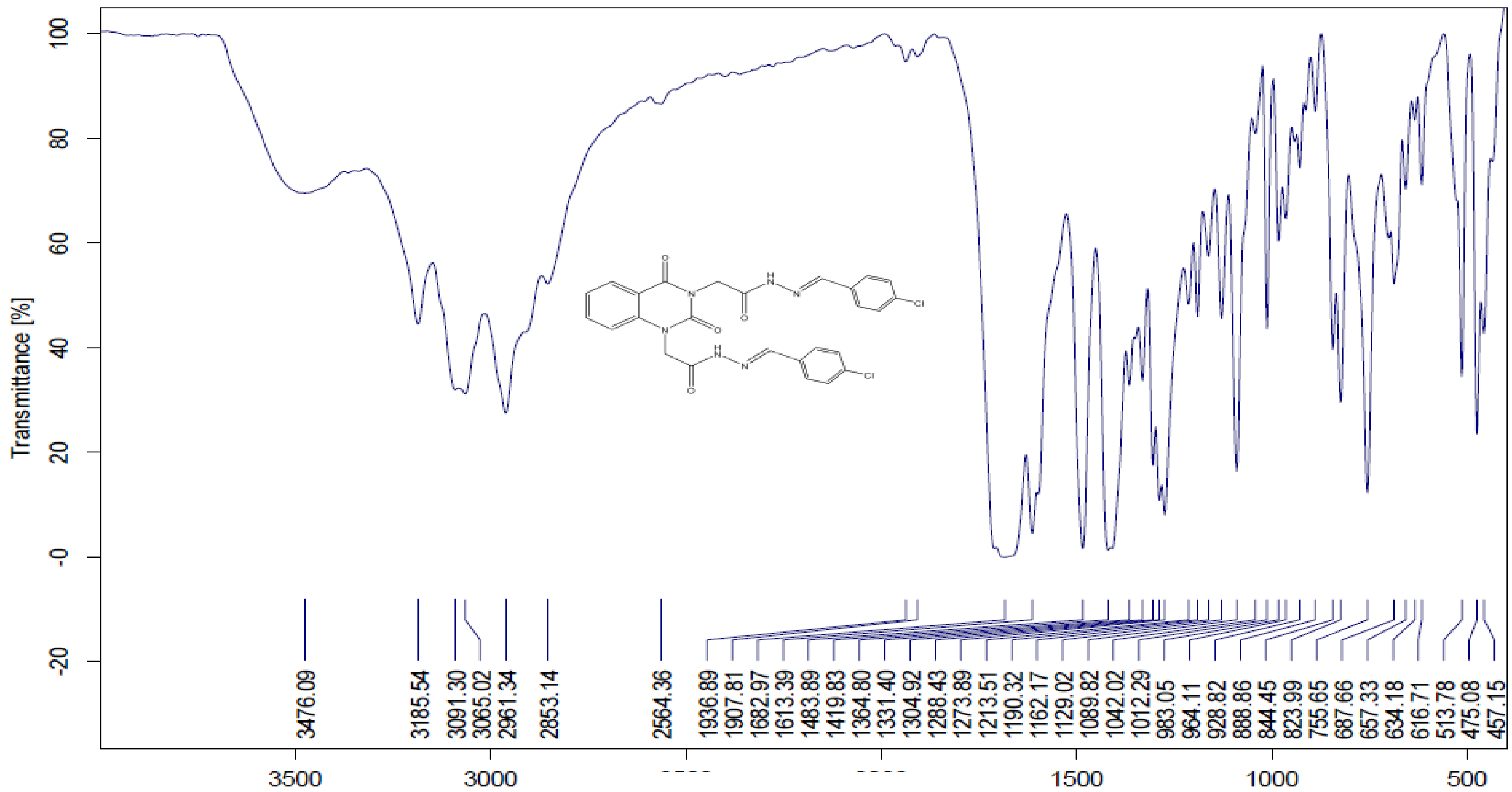

FT-IR of compound (3b)

15-Jan-07 04:21:51

Cairo University
Micro Analytical Center

DI Analysis
Shimadzu Qp-2010 Plus

Sample Information

Analyzed by	: Dr. Mai Younis
Analyzed	: 15/01/2007 04:07:21 ص
Sample Name	: 6a
Sample ID	:
Customer Name	: Dr. Mohamed Omar - Science - Quena
Data File	: C:\GCMSsolution\Data\Project1\6a.QGD
Org Data File	: C:\GCMSsolution\Data\Project1\6a.QGD
Method File	: C:\GCMSsolution\Data\Project1\High Temperature Op
Org Method File	: C:\GCMSsolution\Data\Project1\High Temperature Op
Report File	:
Tuning File	: C:\GCMSsolution\System\Tune1_default.qgt
$EndIf$Modified by	: Dr. Mai Younis
Modified	: 15/01/2007 04:14:59 ص

Method

===== Analytical Line 1 =====

IonSourceTemp	:250.00 °C
[MS Table]	
--Group 1 - Event 1--	
Start Time	:0.00min
End Time	:10.00min
ACQ Mode	:Scan
Event Time	:0.50sec
Scan Speed	:2000
Start m/z	:50.00
End m/z	:900.00
Electron Voltage	: 70 eV
Ionization Mode	: EI

C:\GCMSsolution\Data\Project1\6a.QGD

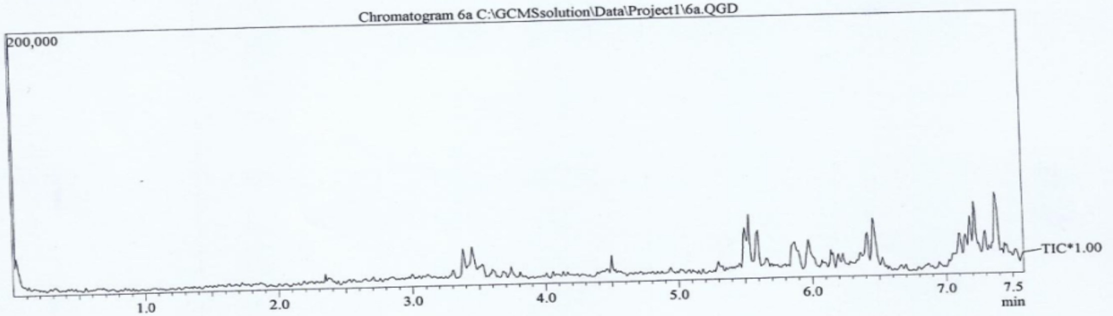

Spectrum

Line#:1 R.Time:6.0(Scan#:715)
MassPeaks:33
RawMode:Single 6.0(715) BasePeak:132(466)
BG Mode:None Group 1 - Event 1

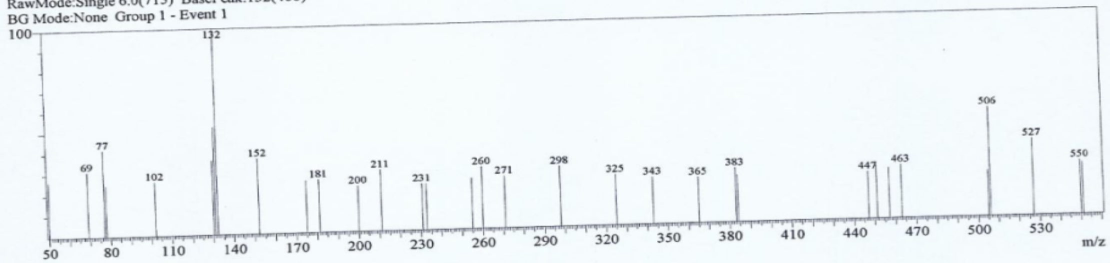

MS of compound (3b)

-

-Compound (3c):

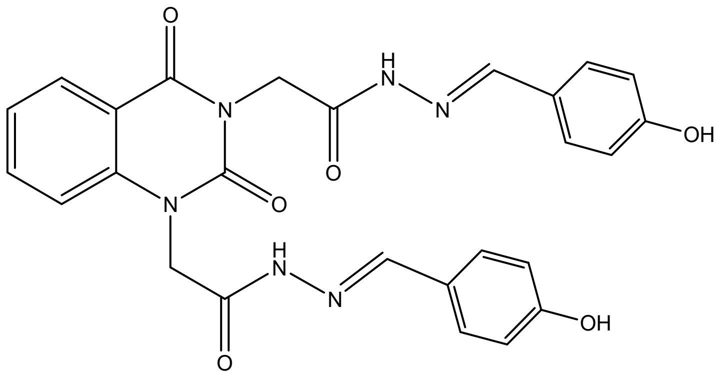

3c

-

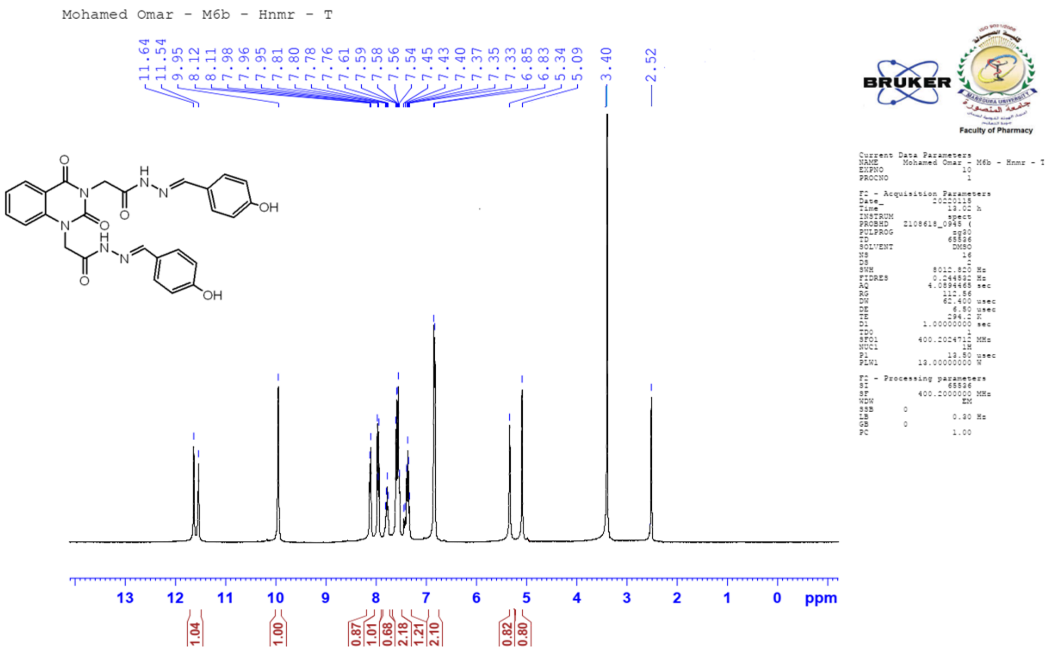

^{1}H-NMR of compound (3c)

-

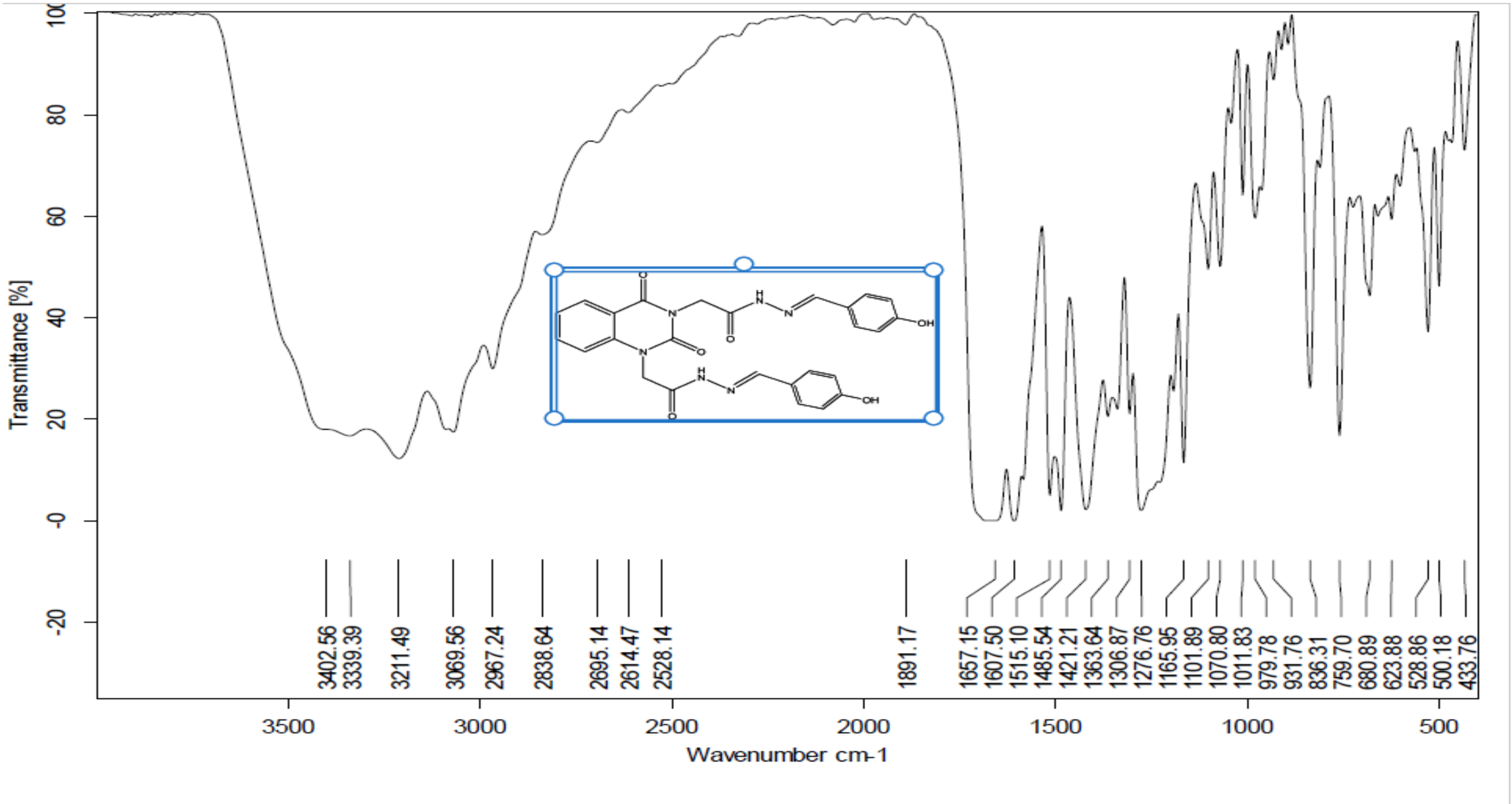

FT-IR of compound (3c)

-

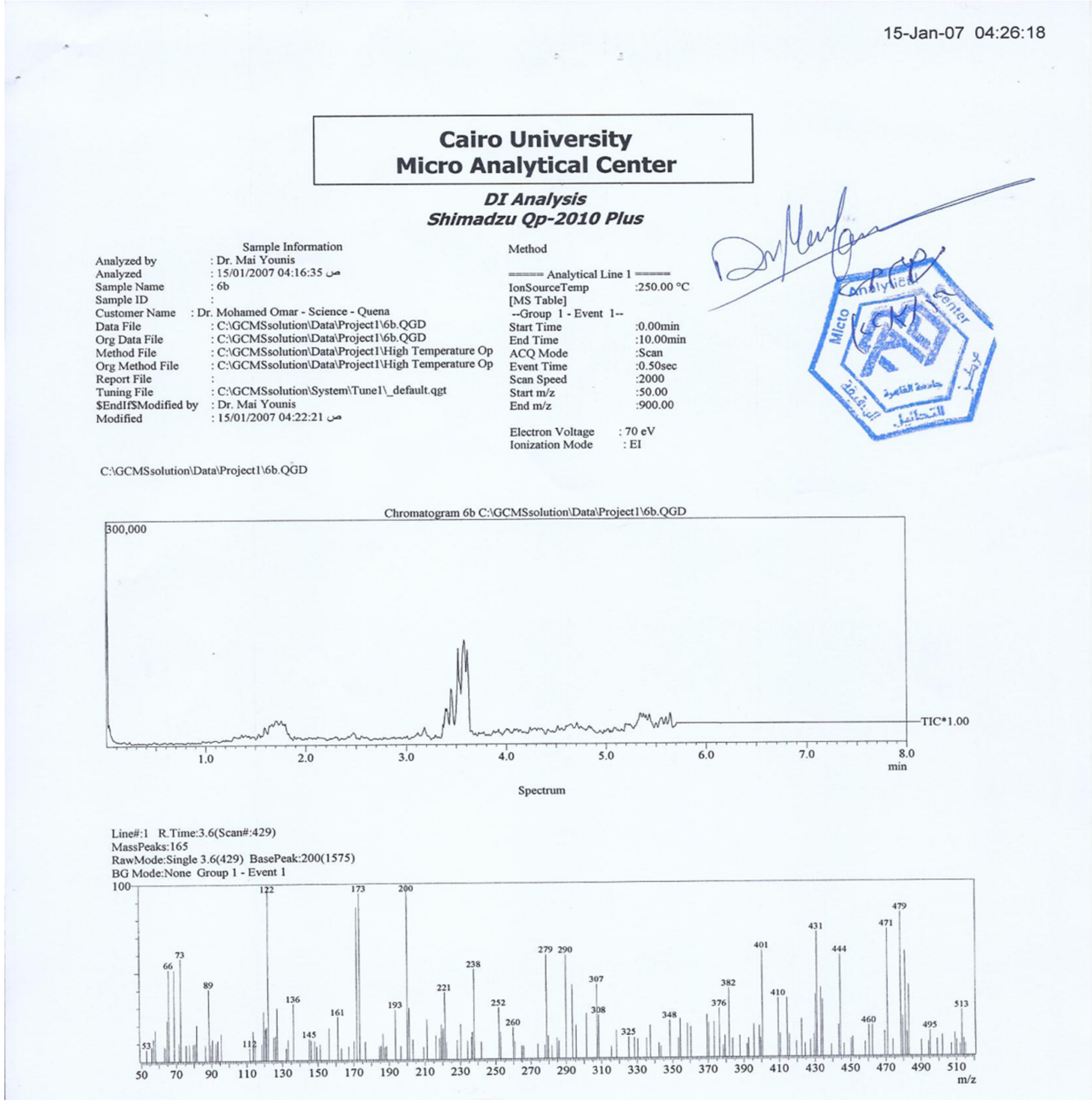

15-Jan-07 04:26:18

Cairo University
Micro Analytical Center

DI Analysis
Shimadzu Qp-2010 Plus

Sample Information

Analyzed by	: Dr. Mai Younis
Analyzed	: 15/01/2007 04:16:35 ص
Sample Name	: 6b
Sample ID	:
Customer Name	: Dr. Mohamed Omar - Science - Quena
Data File	: C:\GCMSsolution\Data\Project1\6b.QGD
Org Data File	: C:\GCMSsolution\Data\Project1\6b.QGD
Method File	: C:\GCMSsolution\Data\Project1\High Temperature Op
Org Method File	: C:\GCMSsolution\Data\Project1\High Temperature Op
Report File	:
Tuning File	: C:\GCMSsolution\System\Tune1_default.qgt
$EndIf$Modified by	: Dr. Mai Younis
Modified	: 15/01/2007 04:22:21 ص

Method

===== Analytical Line 1 =====

IonSourceTemp	:250.00 °C
[MS Table]	
--Group 1 - Event 1--	
Start Time	:0.00min
End Time	:10.00min
ACQ Mode	:Scan
Event Time	:0.50sec
Scan Speed	:2000
Start m/z	:50.00
End m/z	:900.00
Electron Voltage	: 70 eV
Ionization Mode	: EI

C:\GCMSsolution\Data\Project1\6b.QGD

MS of compound (3c)

-

-Compound (3d):

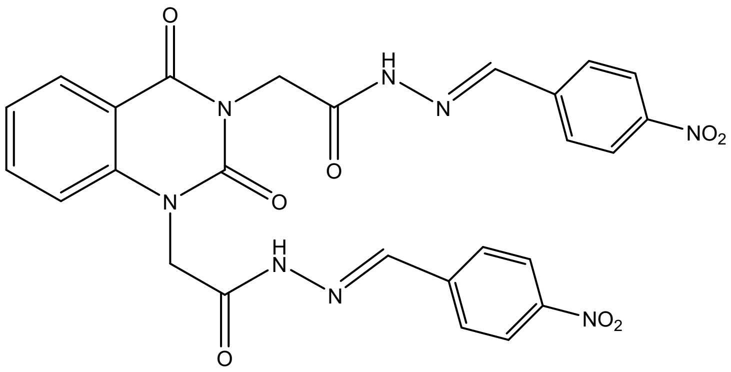

3d

-

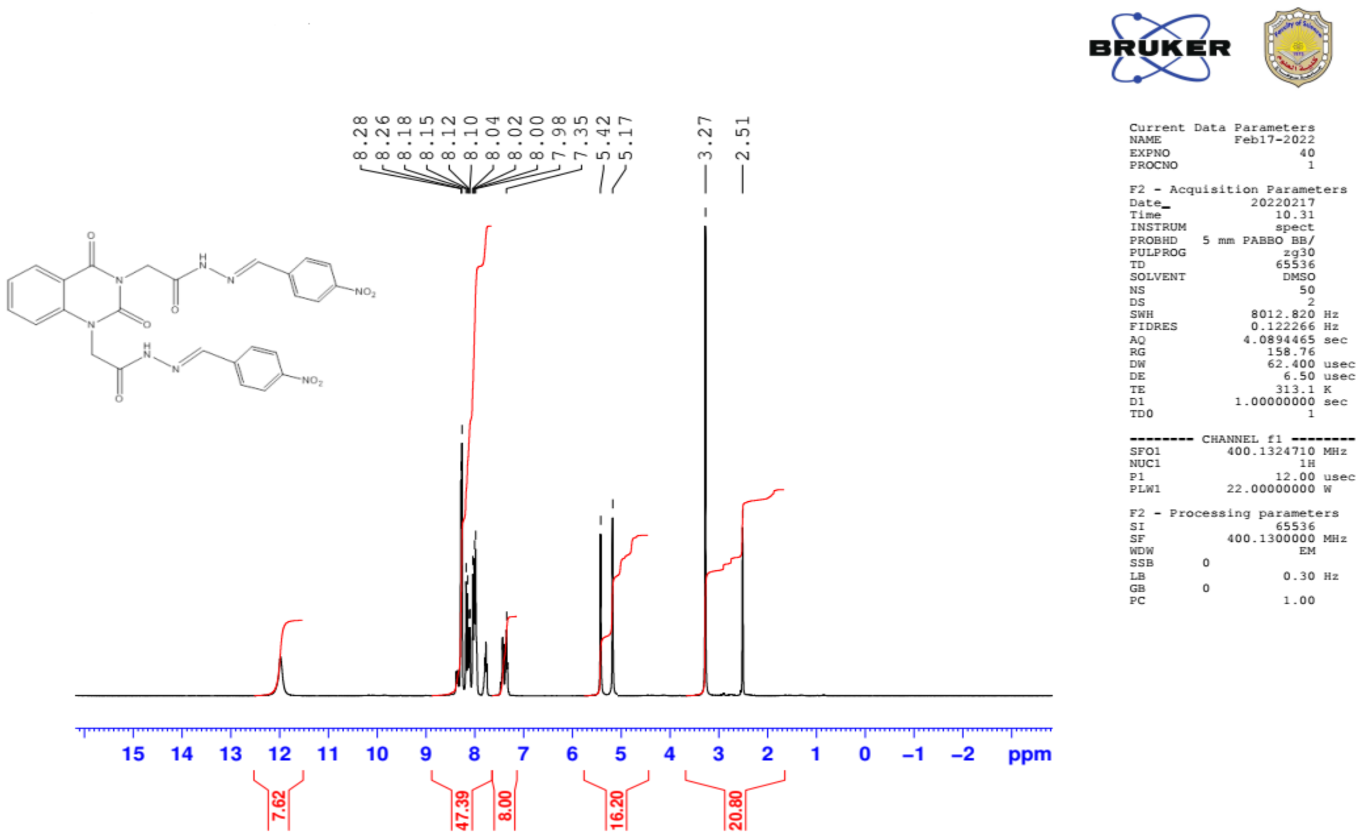

^{1}H-NMR of compound (3d)

-

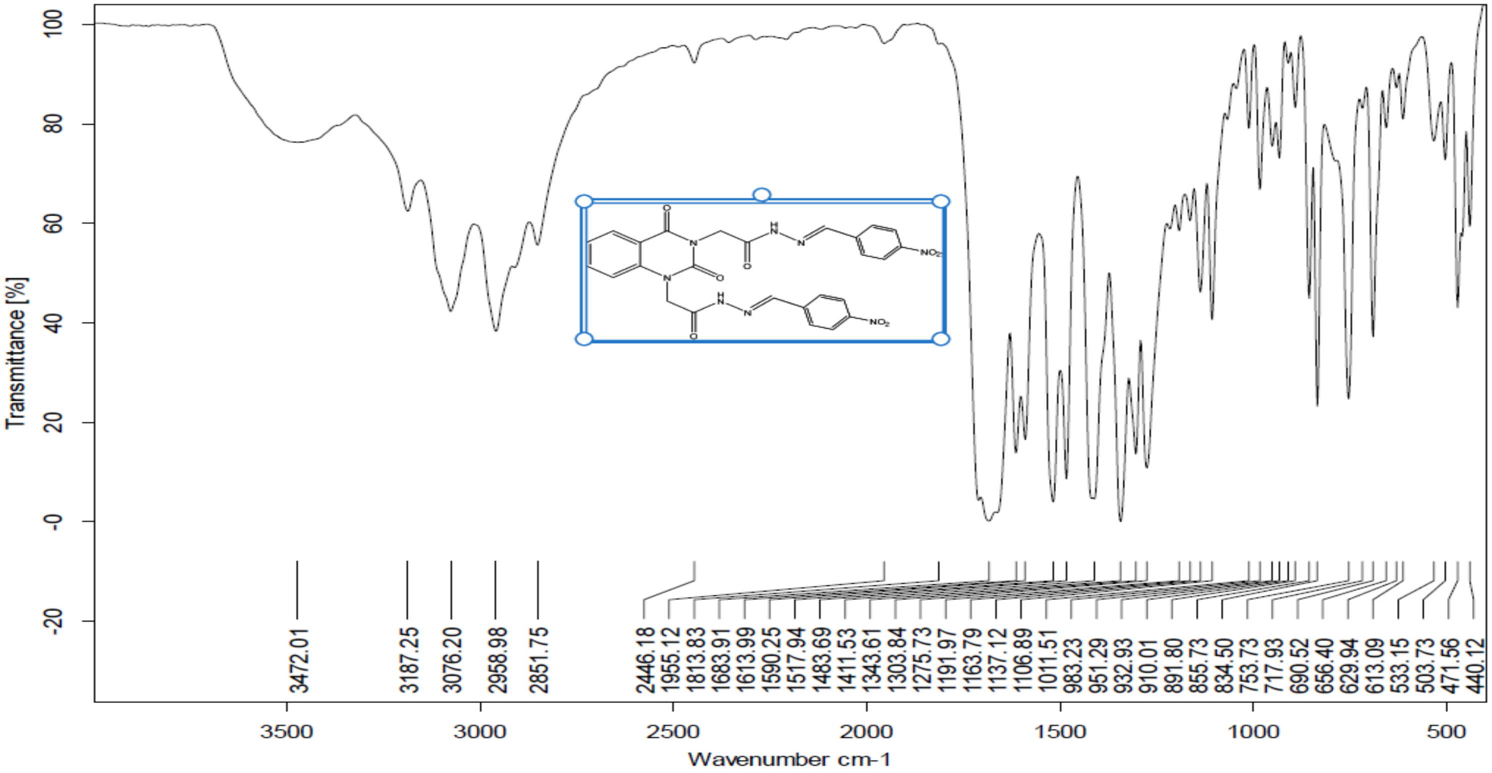

FT-IR of compound (3d)

-

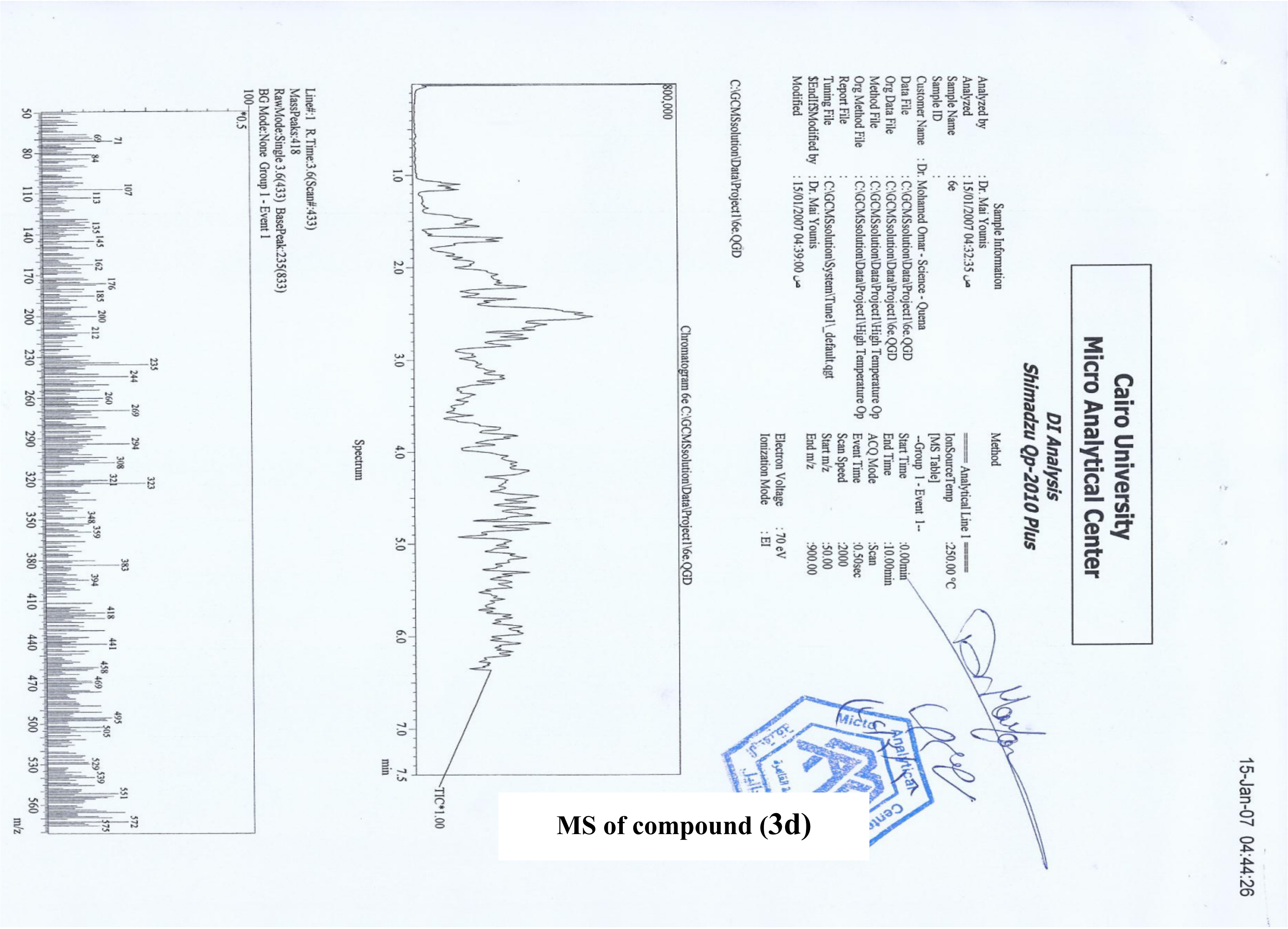

MS of compound (3d)

-

Compound (3e):

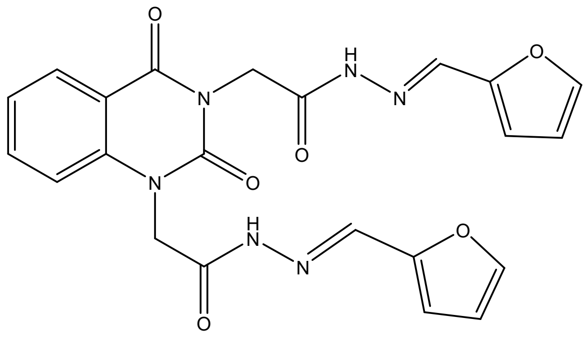

3e

-

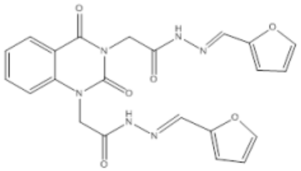

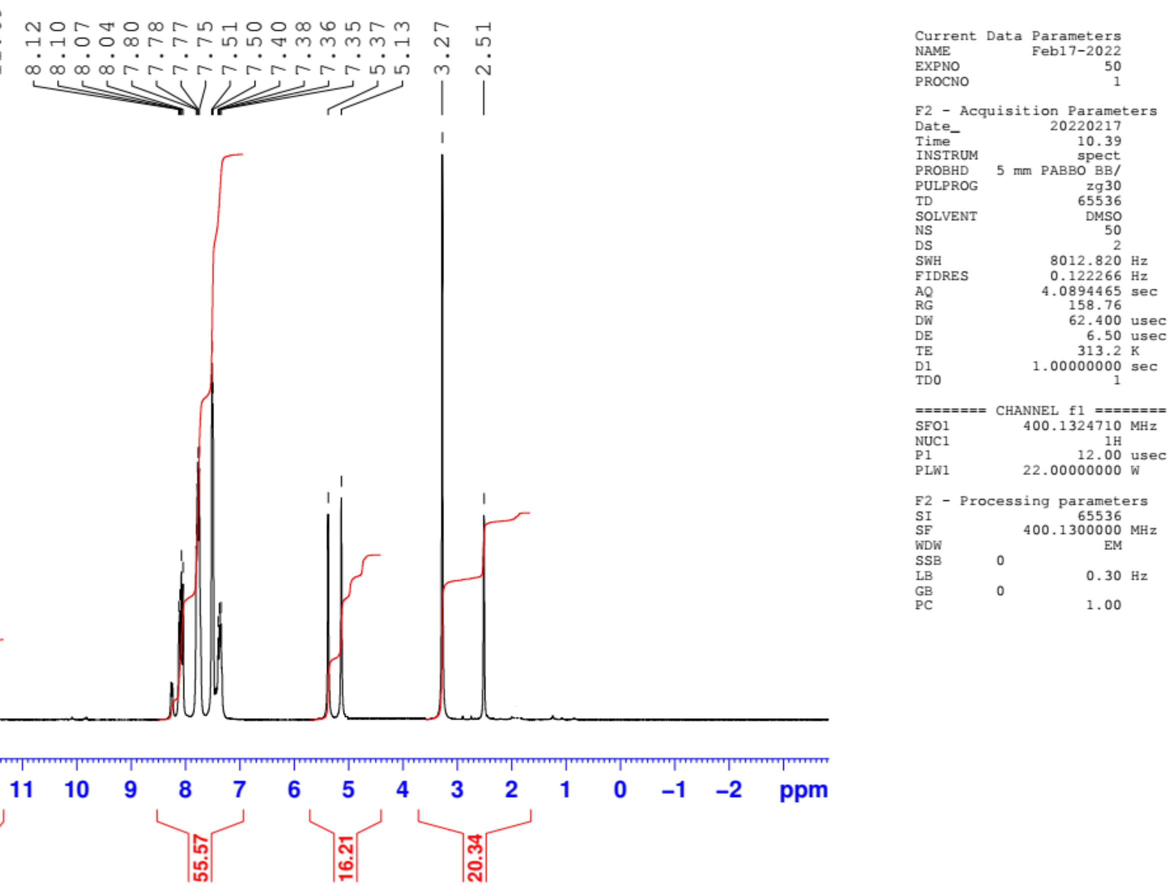

^{1}H-NMR of compound (3e)

-

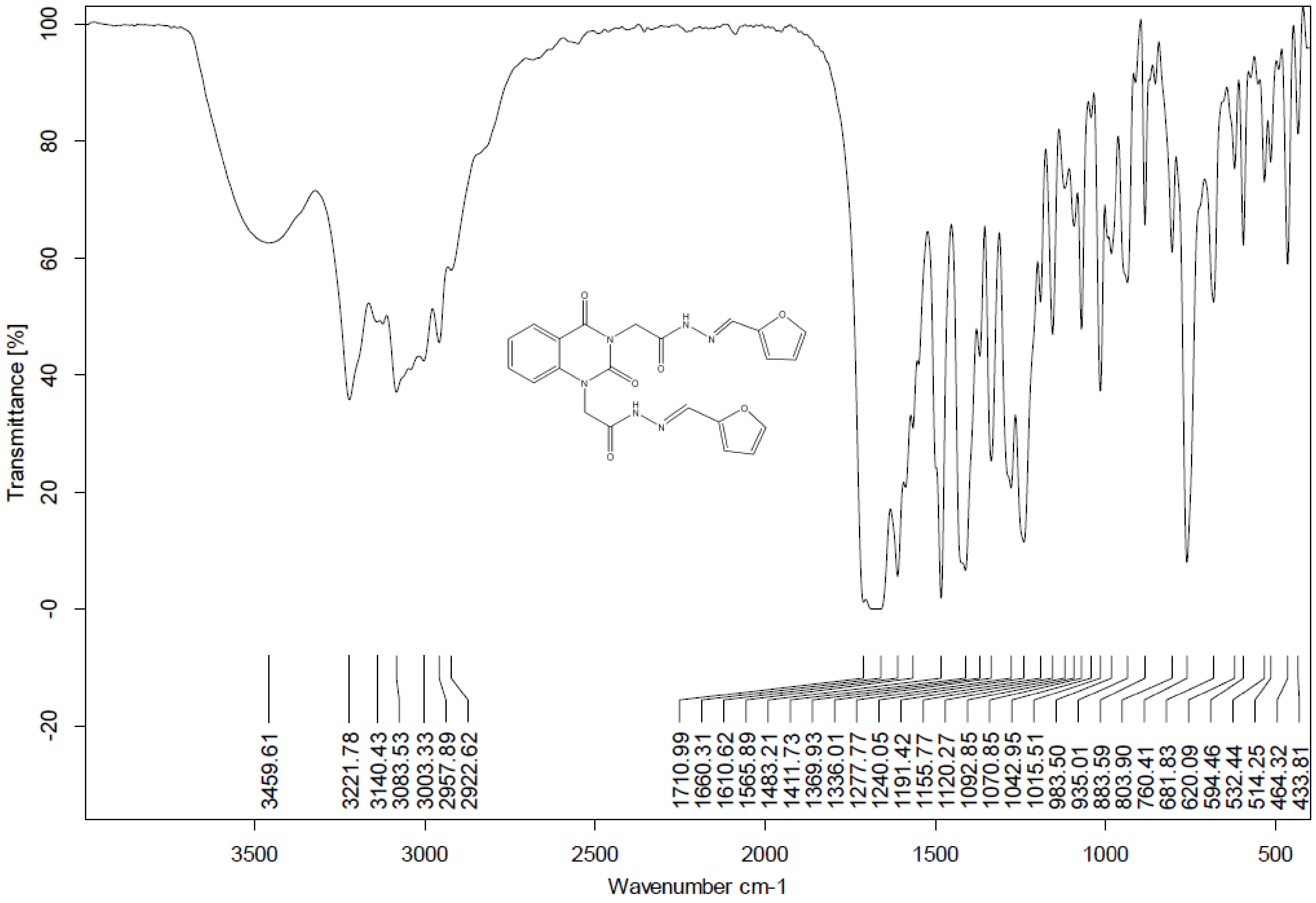

FT-IR of compound (3e)

-

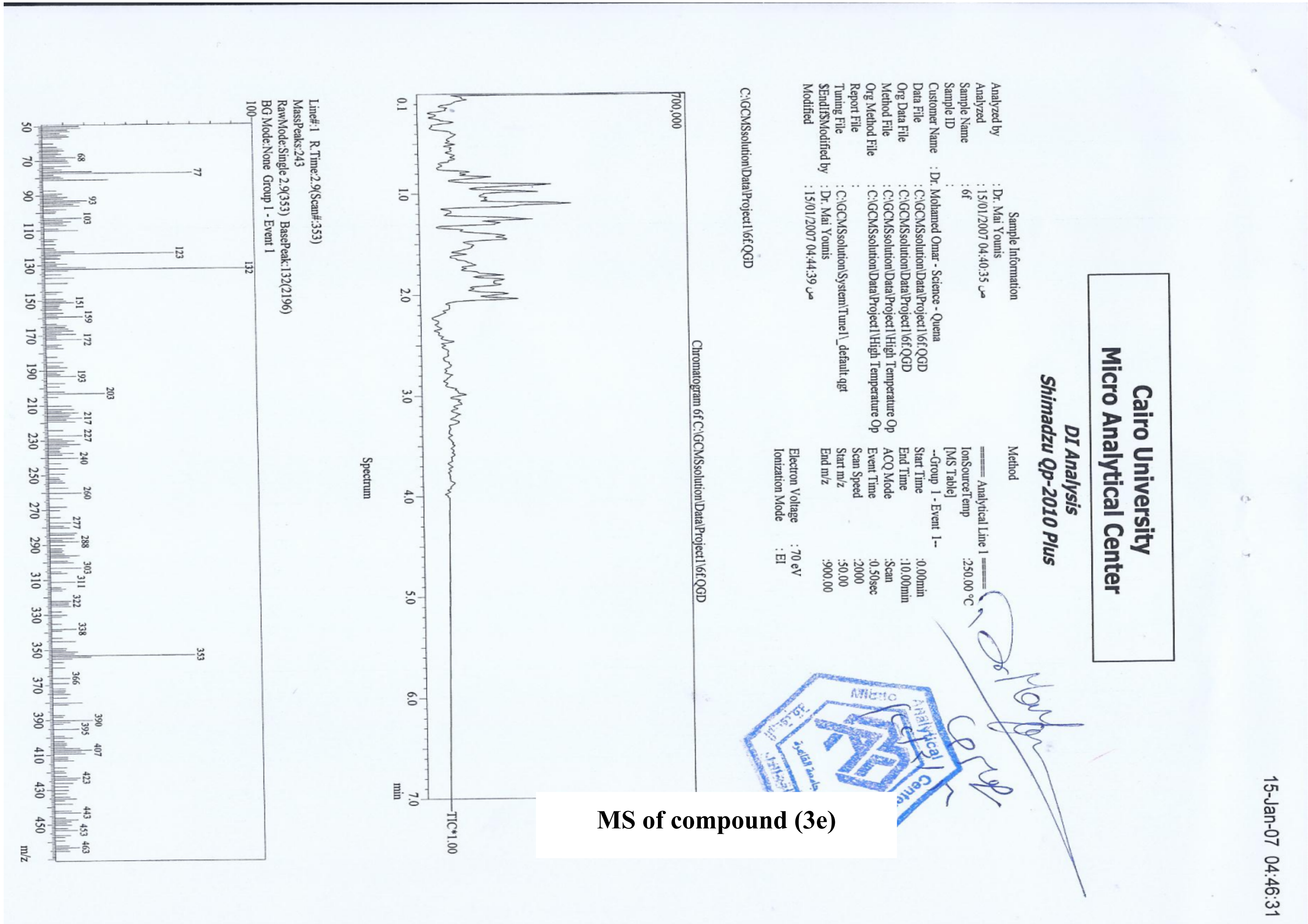

MS of compound (3e)

-

Compound (4a): -

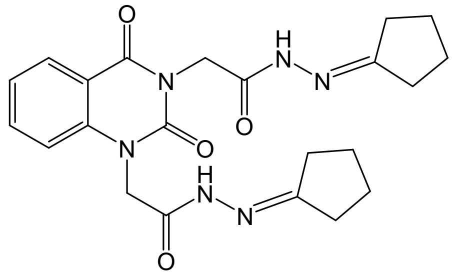

-

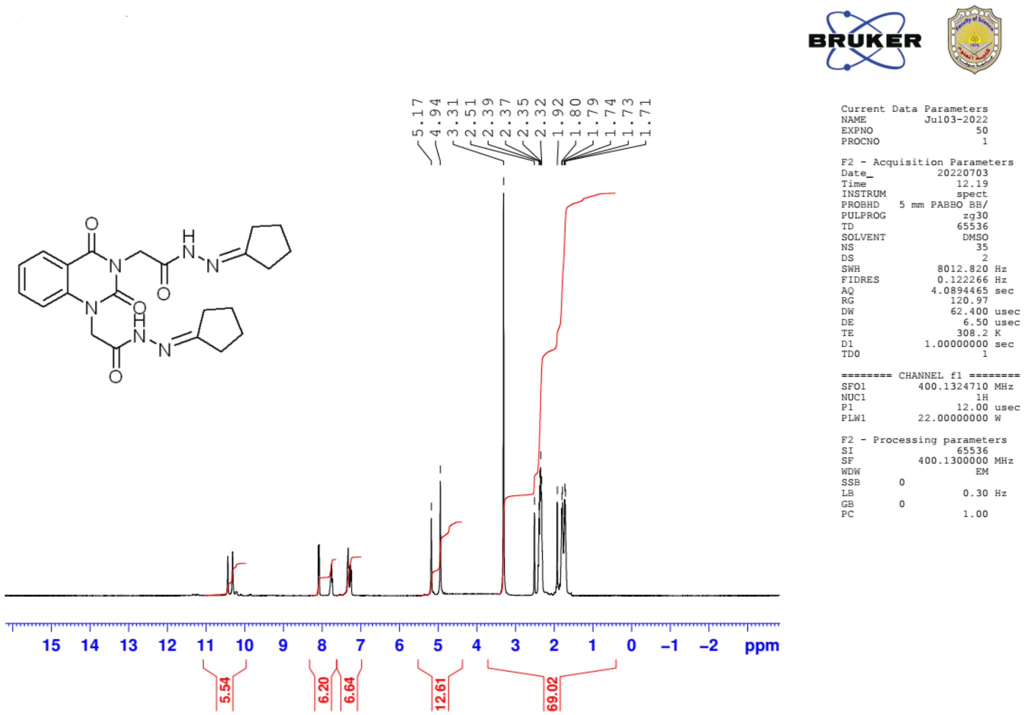

^{1}H-NMR of compound (4a)

-

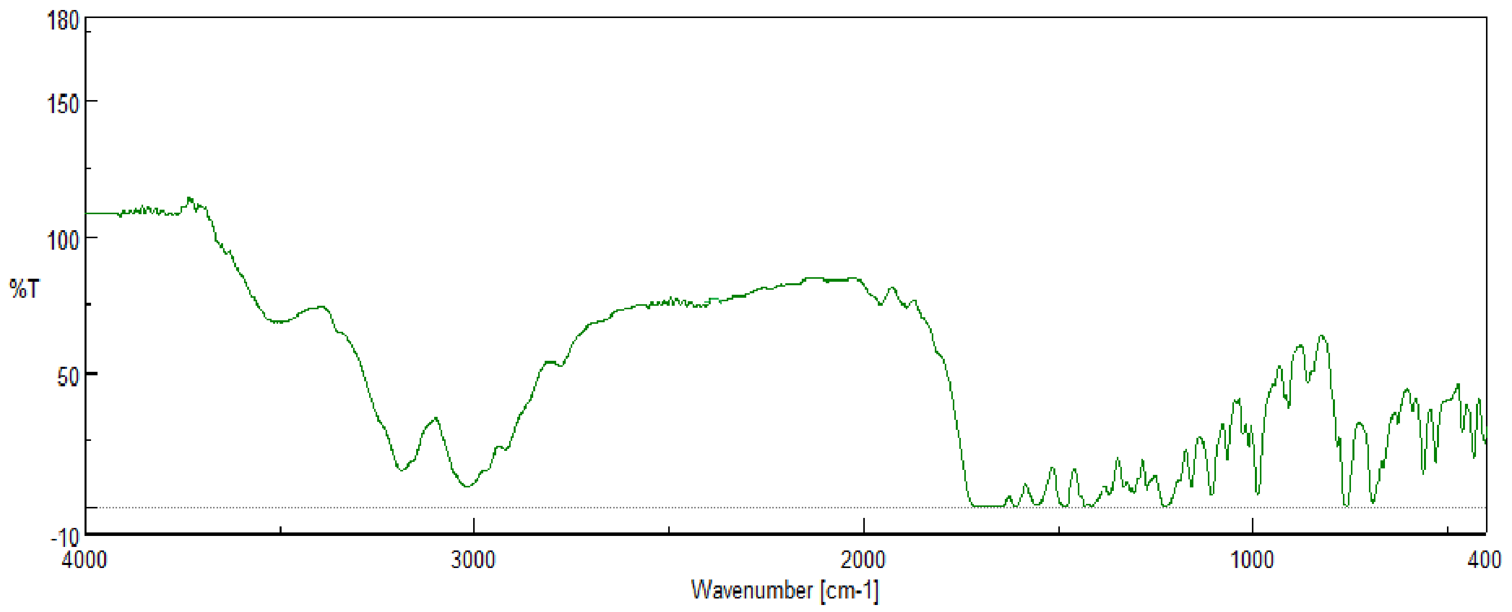

FT-IR of compound (4a)

-

Compound (4b): -

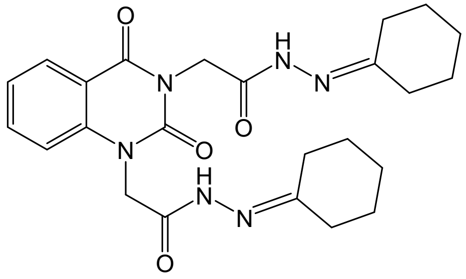

4b

-

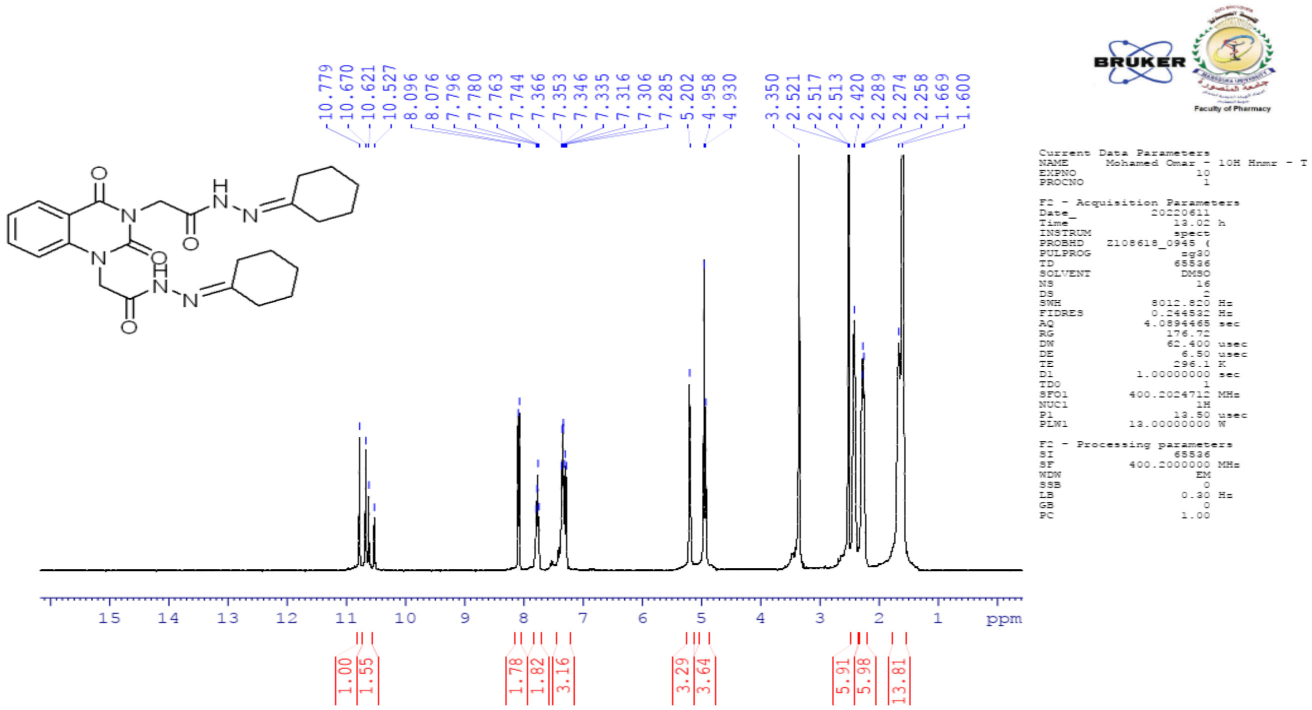

^{1}H-NMR of compound (4b)

-

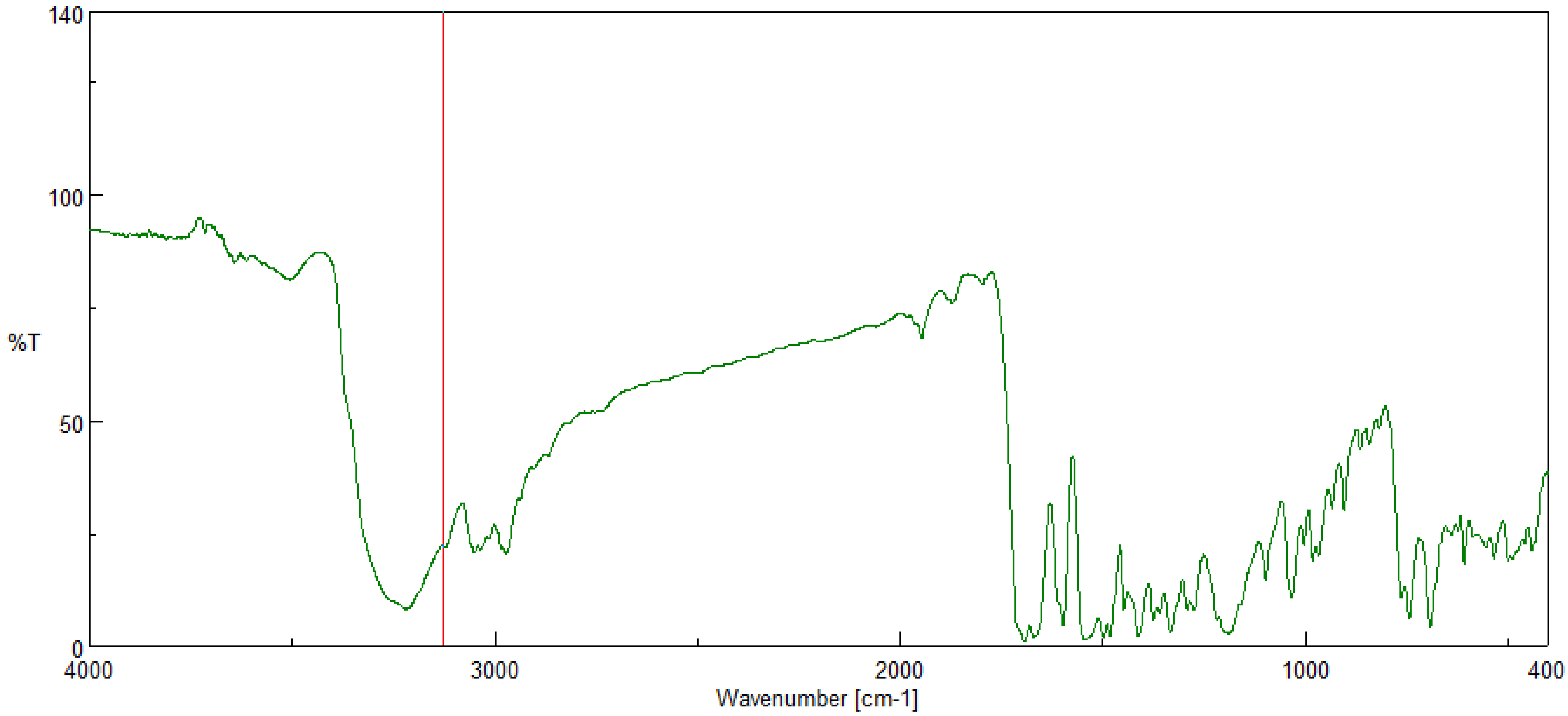

FT-IR of compound (4b)

-

- compound (4c):

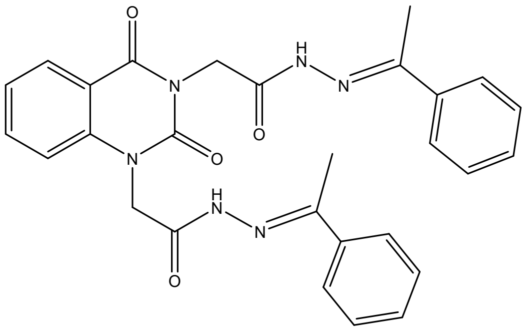

4c

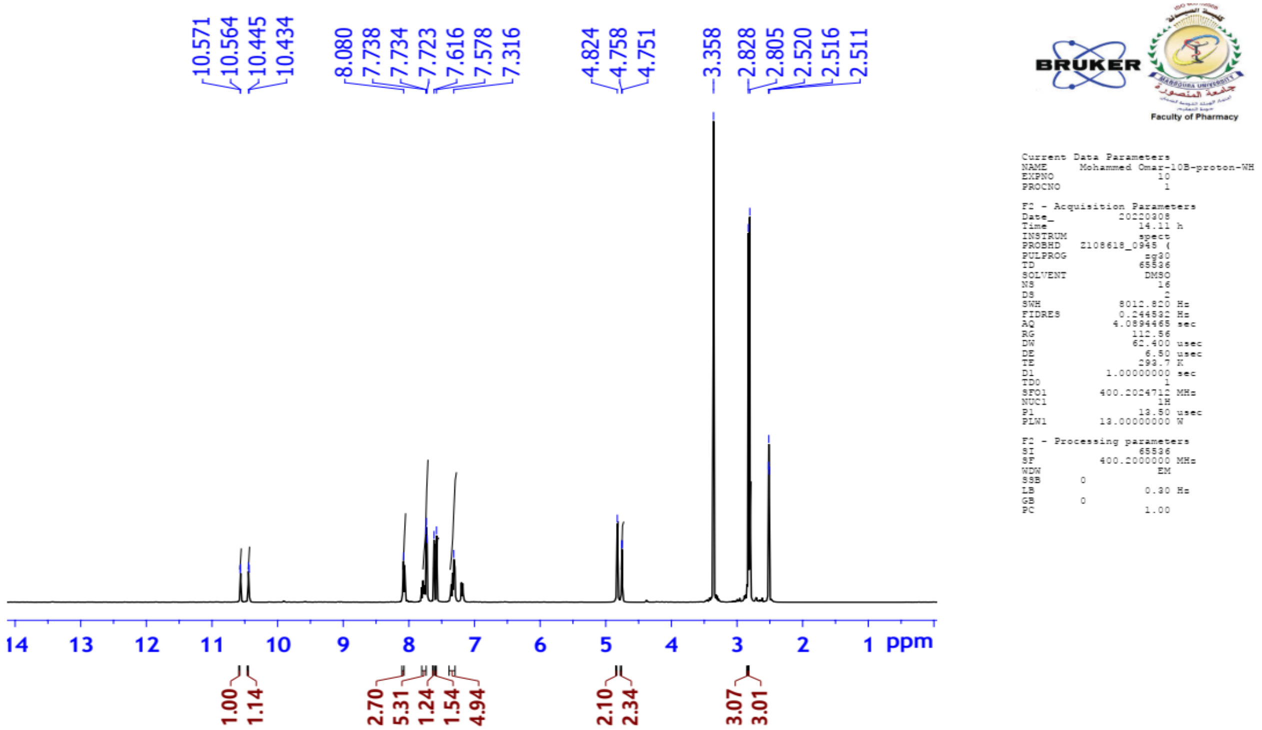

^{1}H-NMR of compound (4c)

-

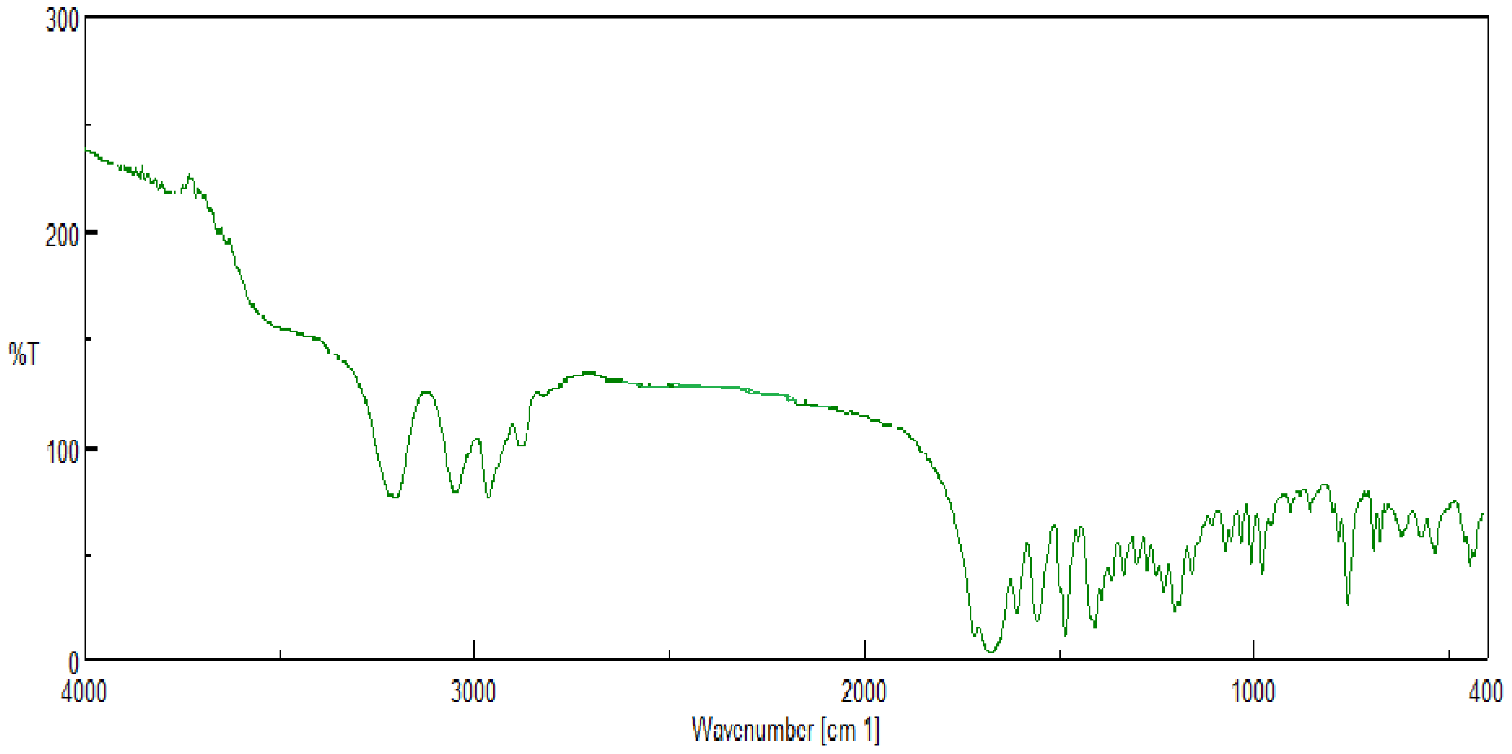

FT-IR of compound (4c)

Compound (4d): -

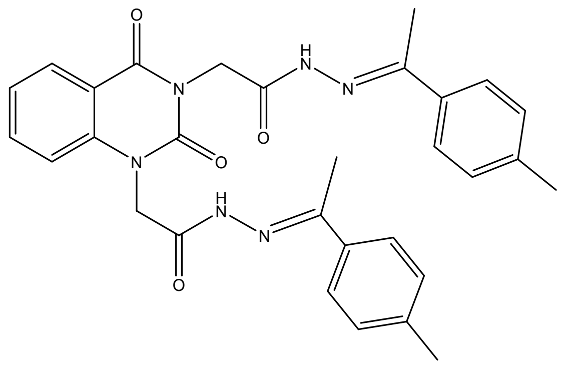

4d

-

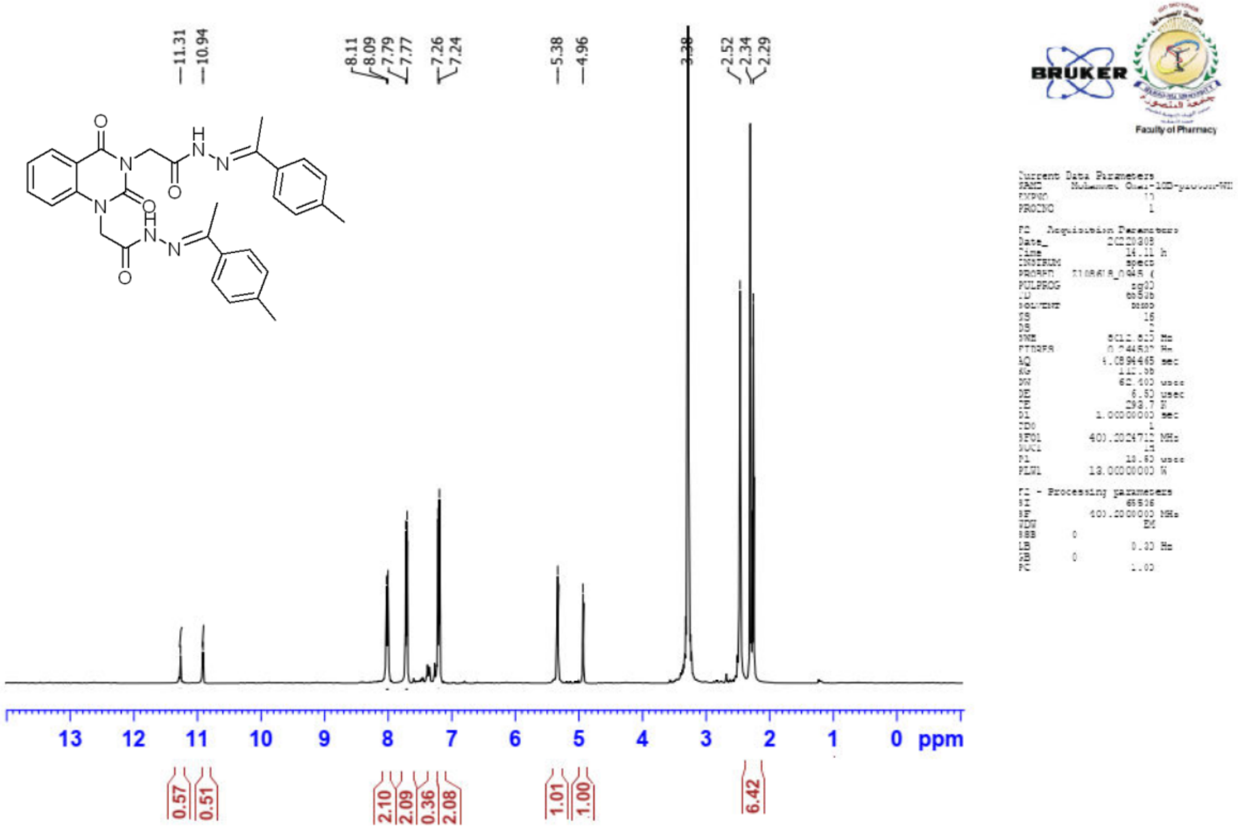

^{1}H-NMR of compound (4d)

-

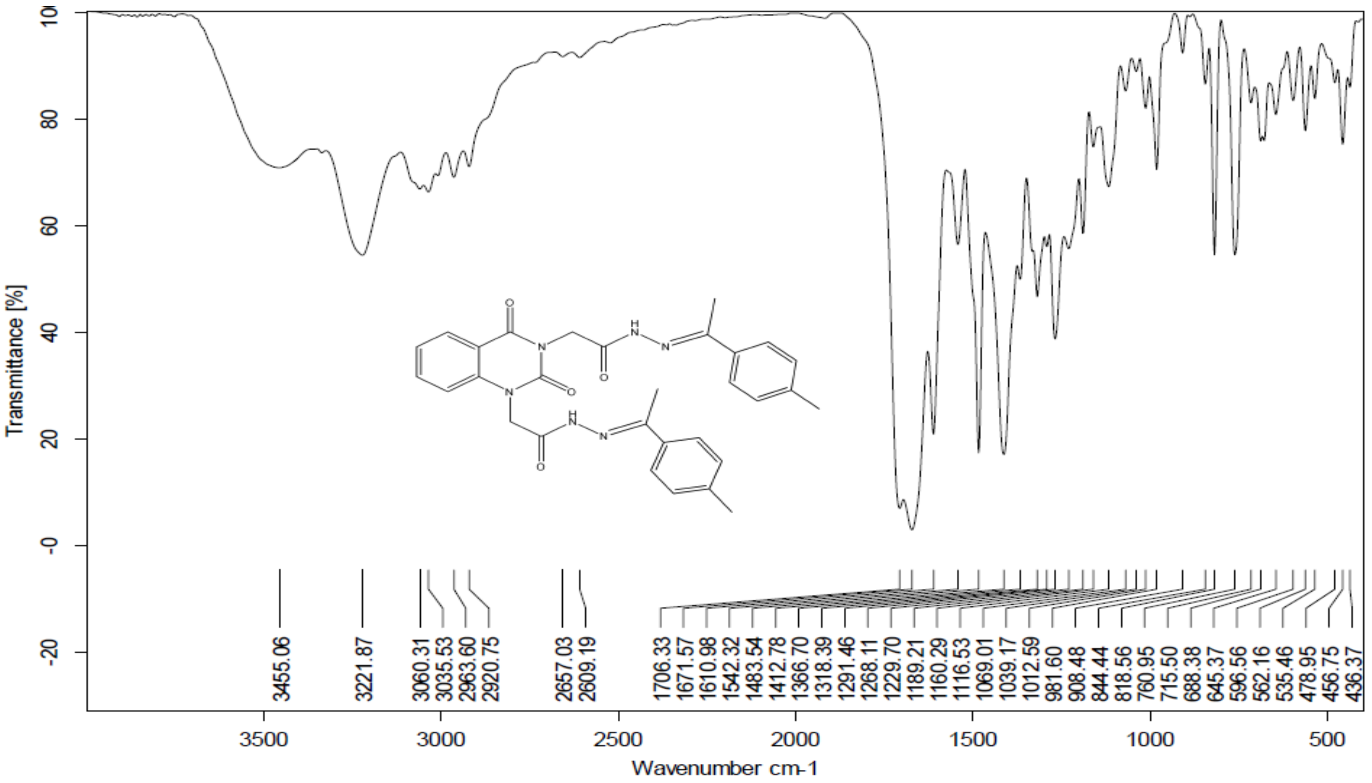

FT-IR of compound (4d)

-

Compound (4e):-

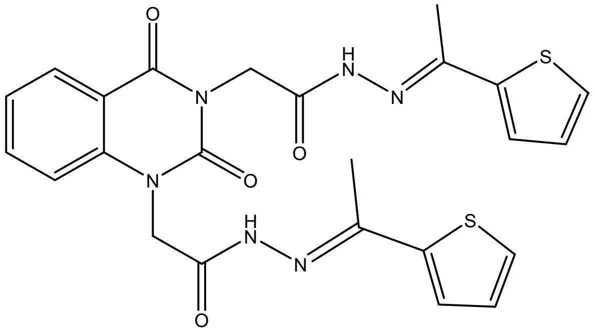

4e

-

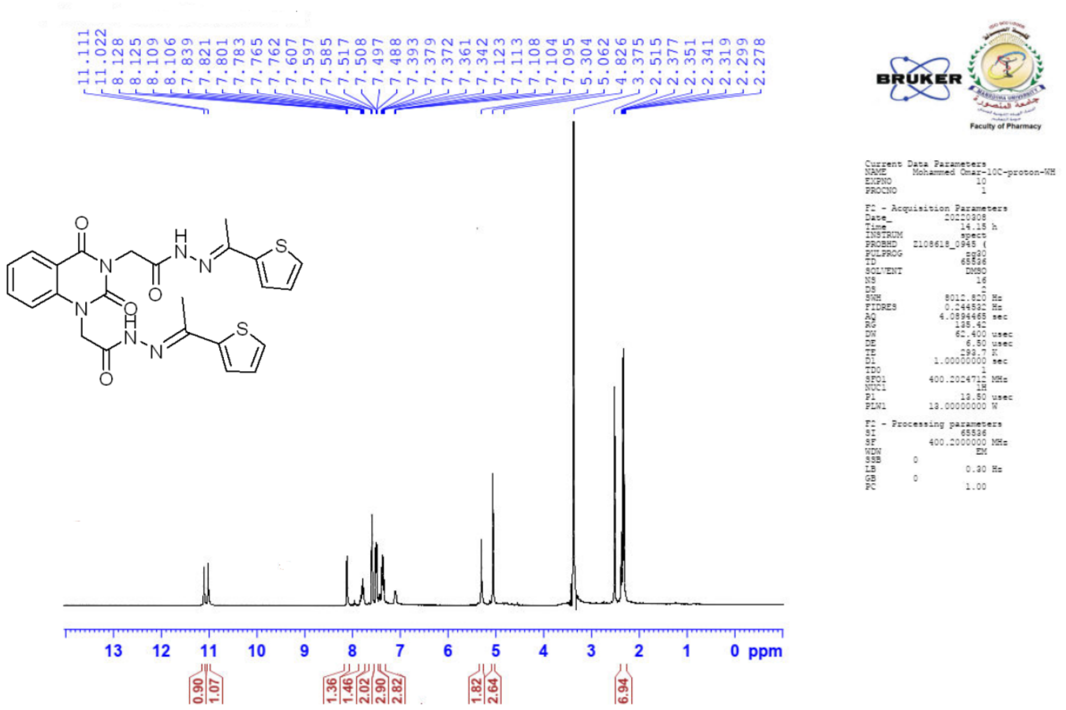

^{1}H-NMR of compound (4e)

-

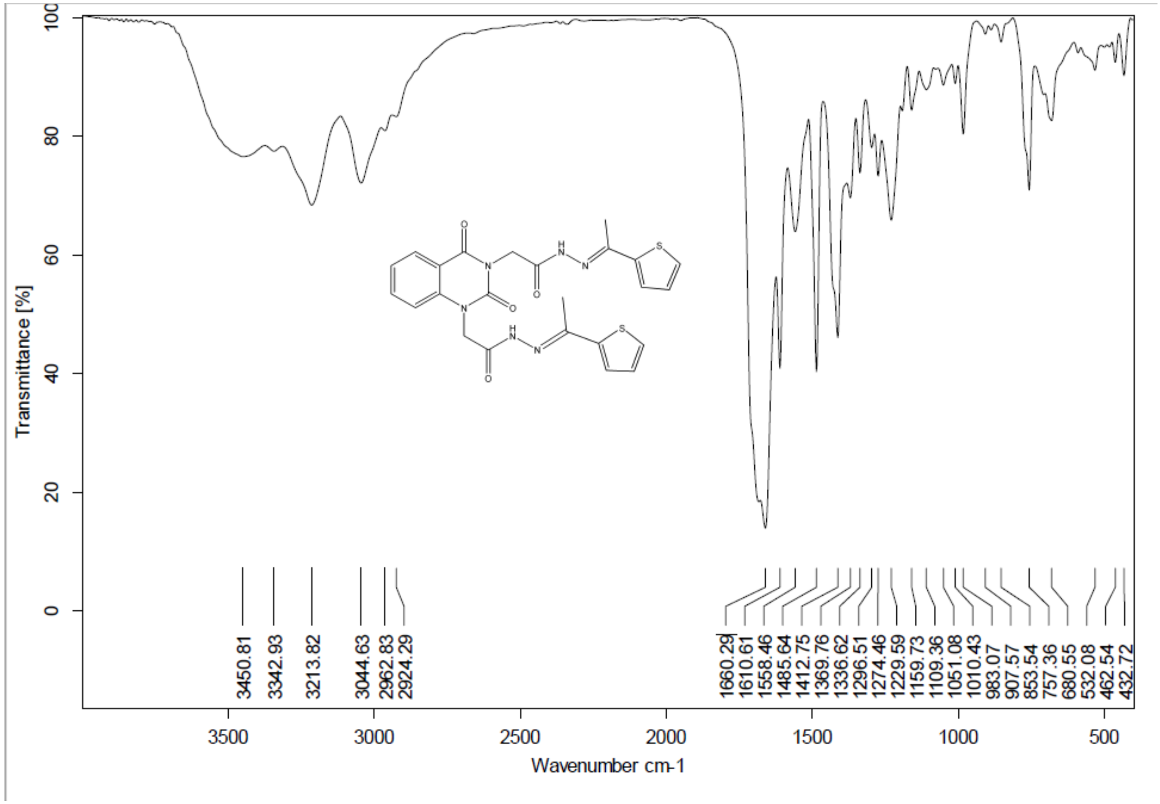

FT-IR of compound (4e)

-

Compound (4f)

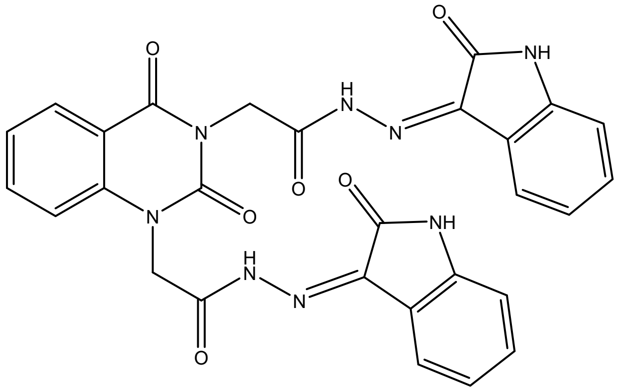

4f

-

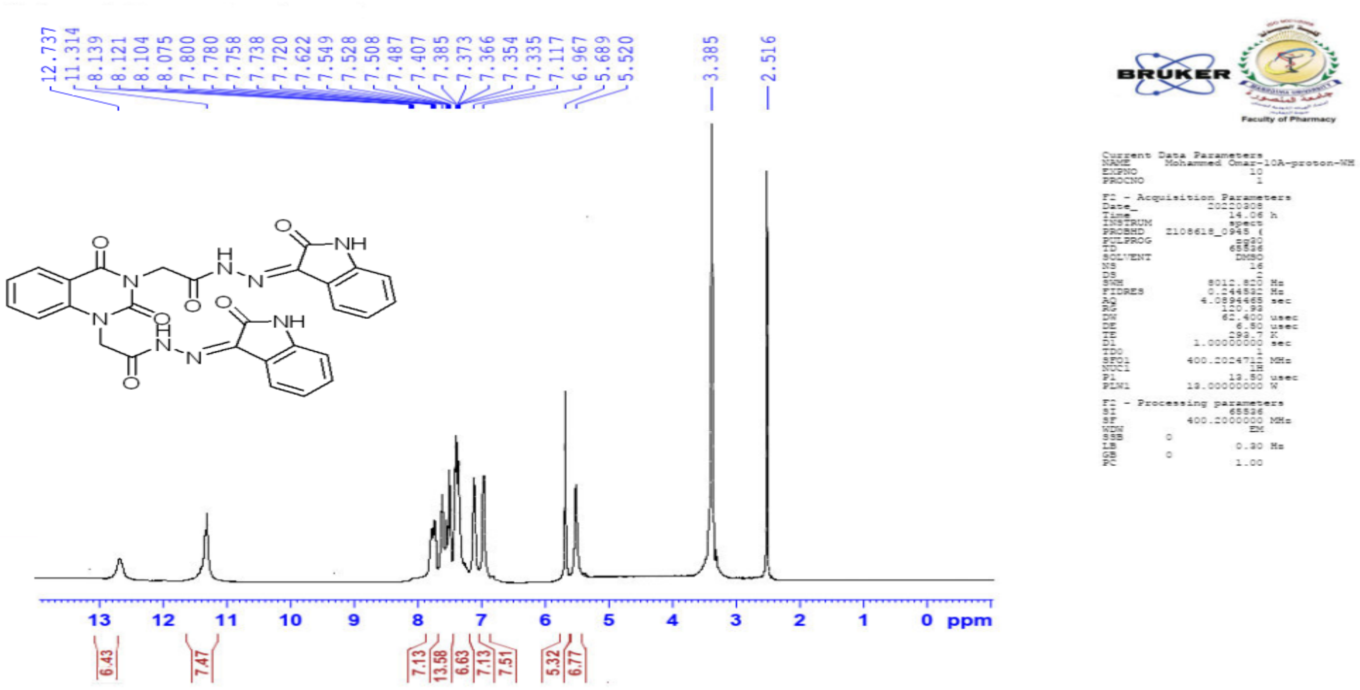

^{1}H-NMR of compound (4f)

-

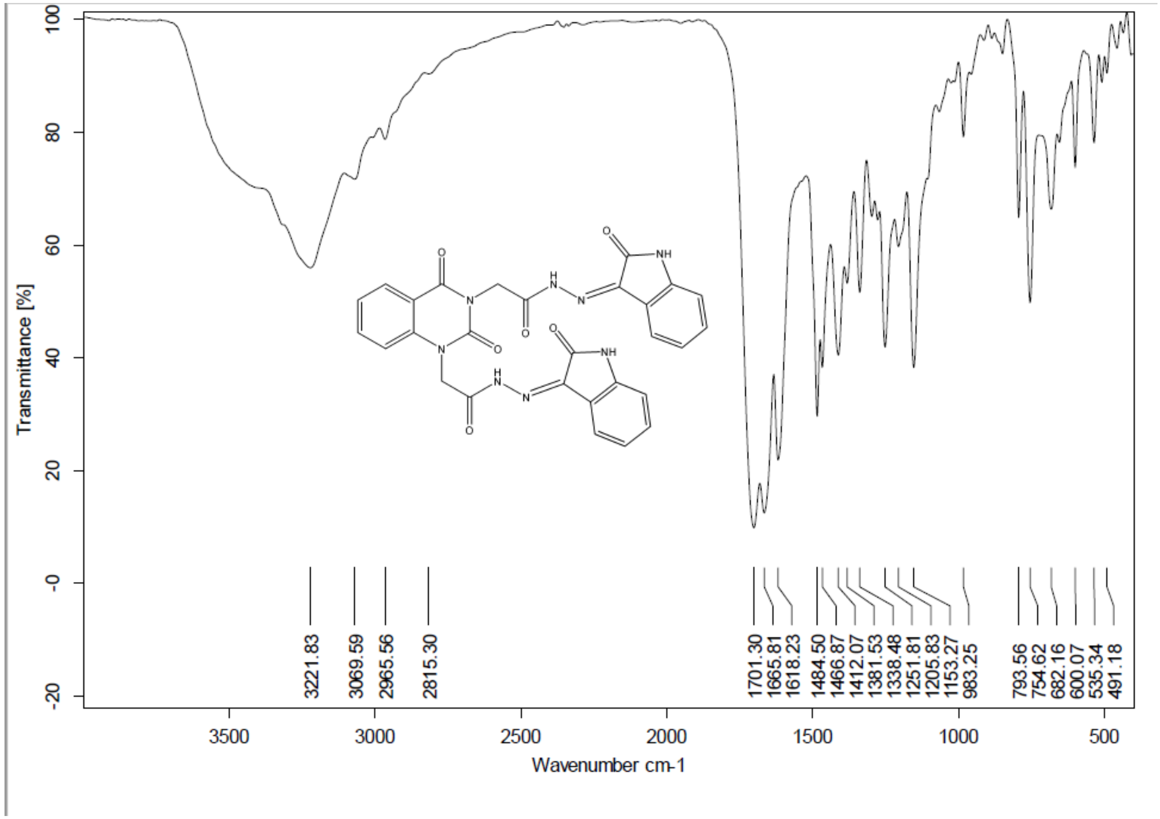

FT-IR of compound (4f)

-

compound (5a)

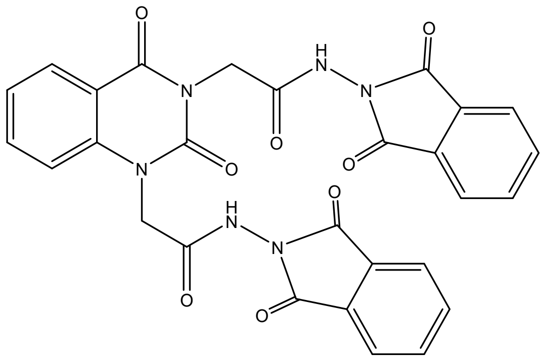

-

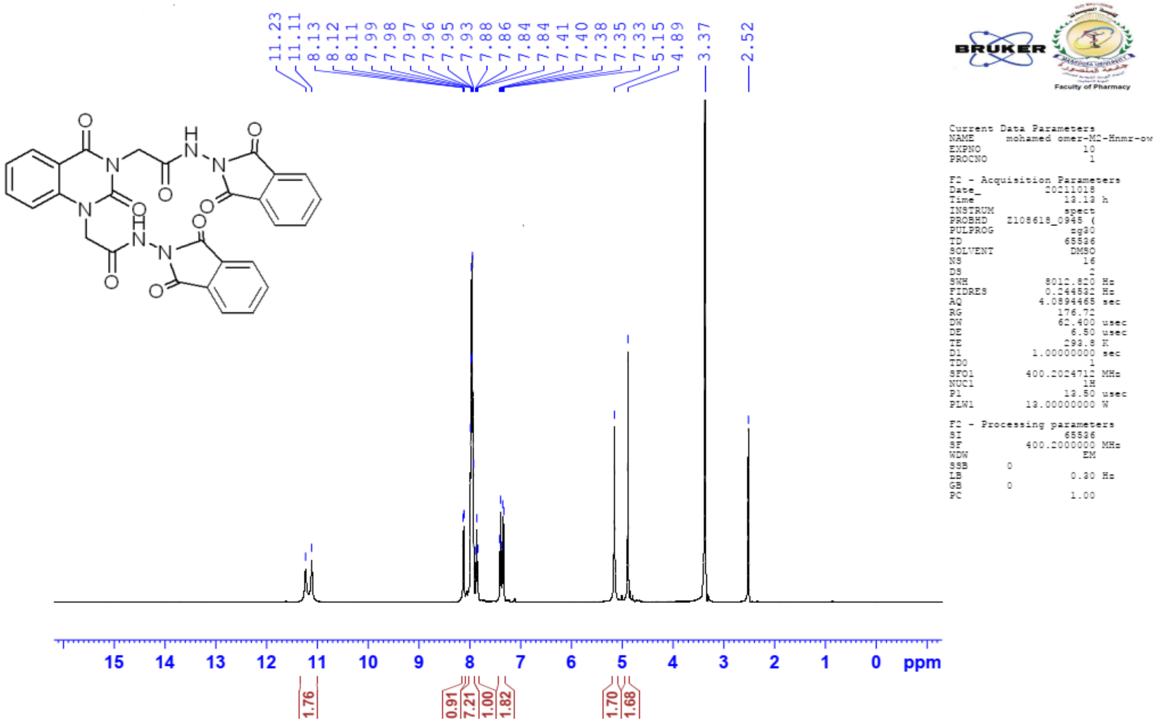

11.23
11.11
8.13
8.12
8.11
7.99
7.98
7.97
7.96
7.95
7.93
7.88
7.86
7.84
7.84
7.41
7.40
7.38
7.35
7.33
5.15
4.89
3.37
2.52
15 14 13 12 11 10 9 8 7 6 5 4 3 2 1 0 ppm
1.76
0.91
7.21
1.00
1.82
1.70
1.68
BRUKER
Faculty of Pharmacy

-

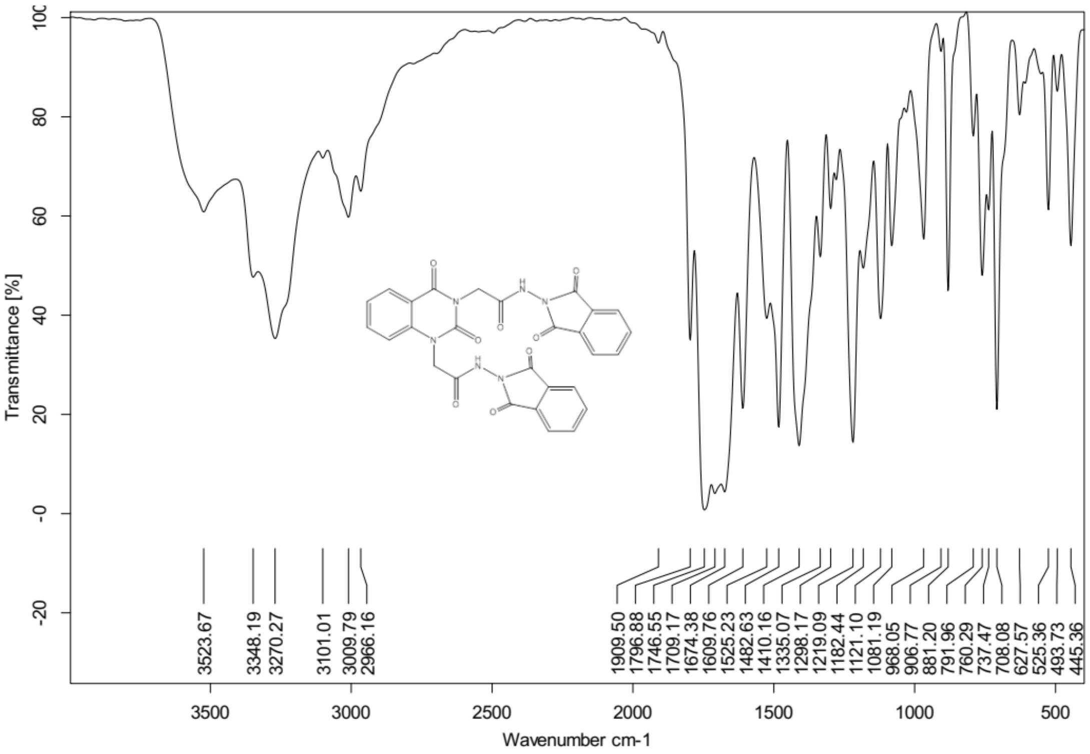

FT-IR of compound (5a)

15-Jan-07 04:04:06

Cairo University
Micro Analytical Center

DI Analysis
Shimadzu Qp-2010 Plus

Sample Information

Analyzed by	: Dr. Mai Younis
Analyzed	: 15/01/2007 03:55:58 ص
Sample Name	: 2a
Sample ID	:
Customer Name	: Dr. Mohamed Omar - Science - Quena
Data File	: C:\GCMSsolution\Data\Project1\2a.QGD
Org Data File	: C:\GCMSsolution\Data\Project1\2a.QGD
Method File	: C:\GCMSsolution\Data\Project1\High Temperature Op
Org Method File	: C:\GCMSsolution\Data\Project1\High Temperature Op
Report File	:
Tuning File	: C:\GCMSsolution\System\Tune1_default.qgt
$EndIf$Modified by	: Dr. Mai Younis
Modified	: 15/01/2007 04:00:33 ص

Method

===== Analytical Line 1 =====

IonSourceTemp	:250.00 °C
[MS Table]	
--Group 1 - Event 1--	
Start Time	:0.00min
End Time	:10.00min
ACQ Mode	:Scan
Event Time	:0.50sec
Scan Speed	:2000
Start m/z	:50.00
End m/z	:900.00
Electron Voltage	: 70 eV
Ionization Mode	: EI

C:\GCMSsolution\Data\Project1\2a.QGD

Chromatogram 2a C:\GCMSsolution\Data\Project1\2a.QGD

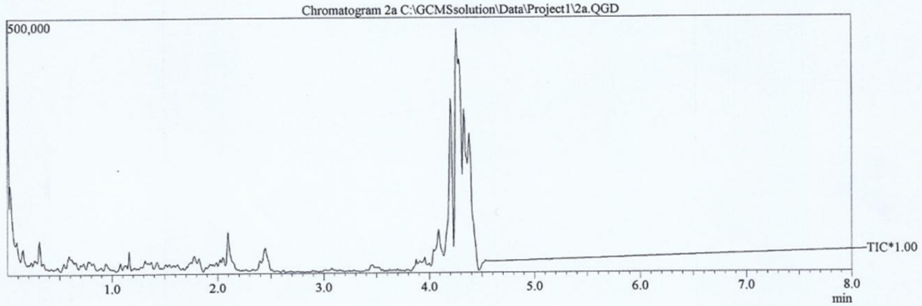

Spectrum

Line#:1 R.Time:4.4(Scan#:526)
MassPeaks:554
RawMode:Single 4.4(526) BasePeak:736(1159)
BG Mode:None Group 1 - Event 1

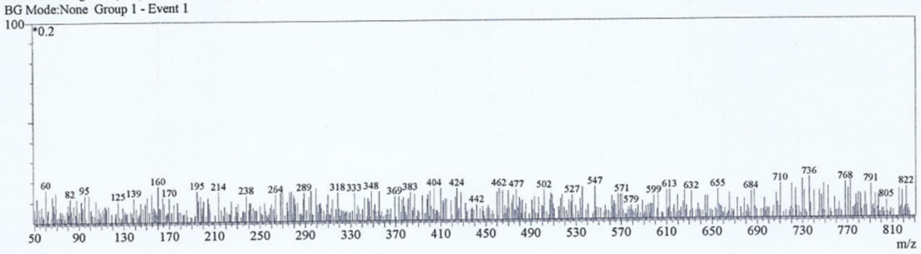

MS of compound (5a)

Compound (5b):-

-

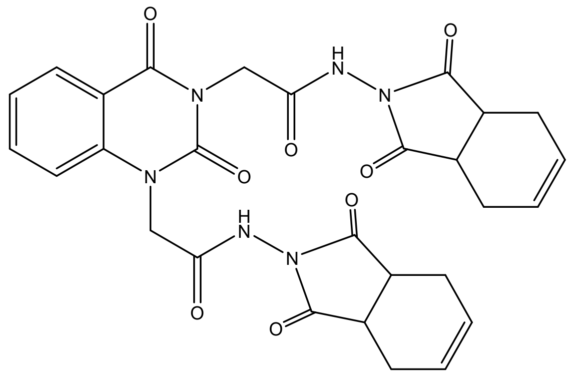

O
N
H
O
O
O
N
H
O
O

-

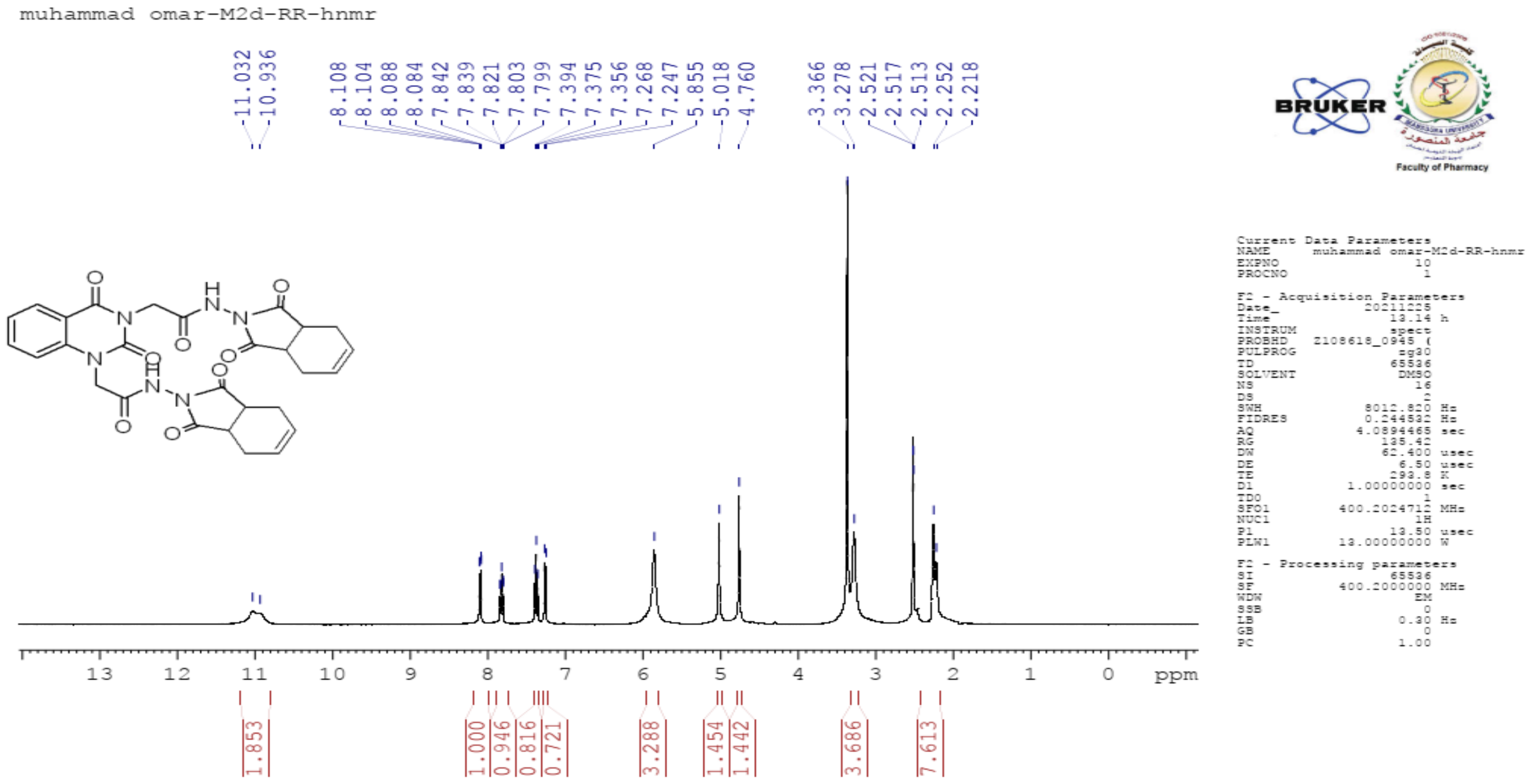

^{1}H-NMR of compound (5b)

-

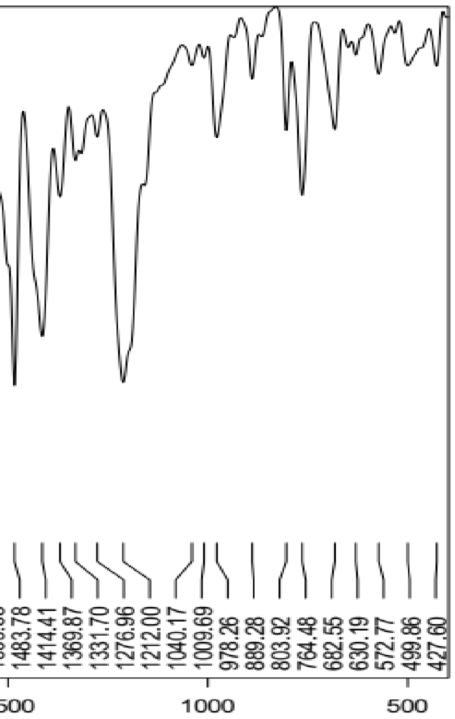

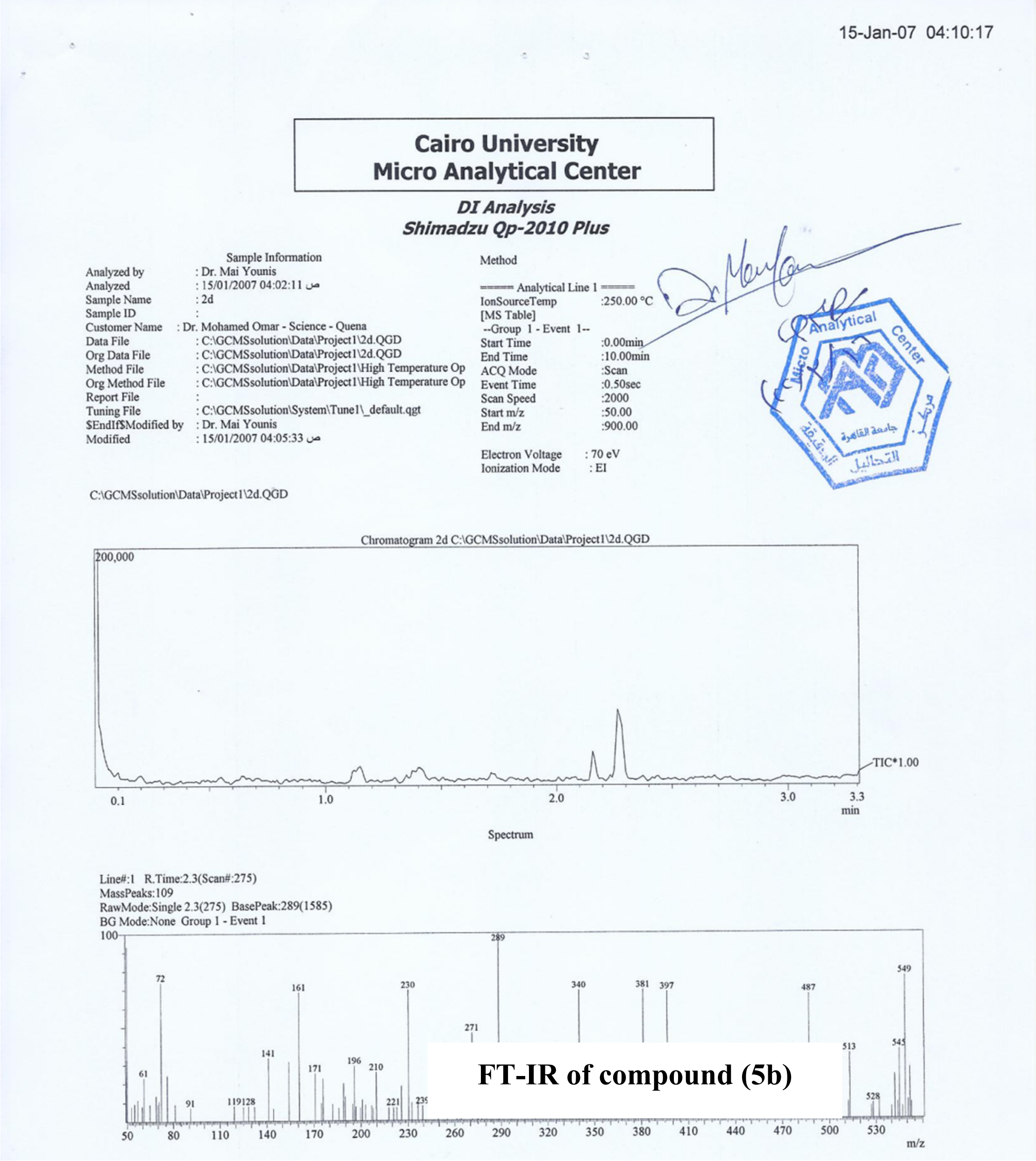

FT-IR of compound (5b)

-

Compound (5c)

MS of compound (5b)

-

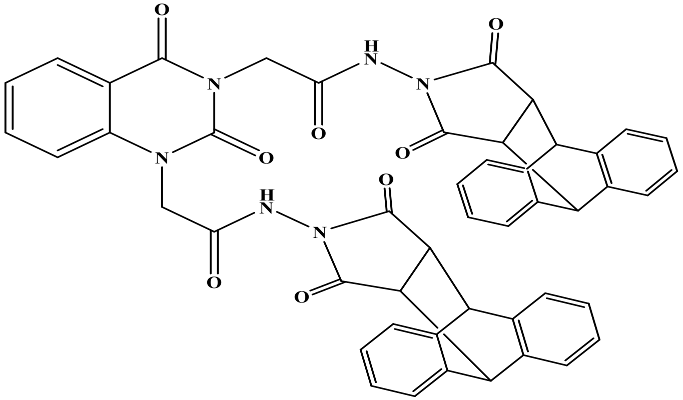

O
N
O
N
O
H
N
N
O
O
O
H
N
N
O
O
O

-

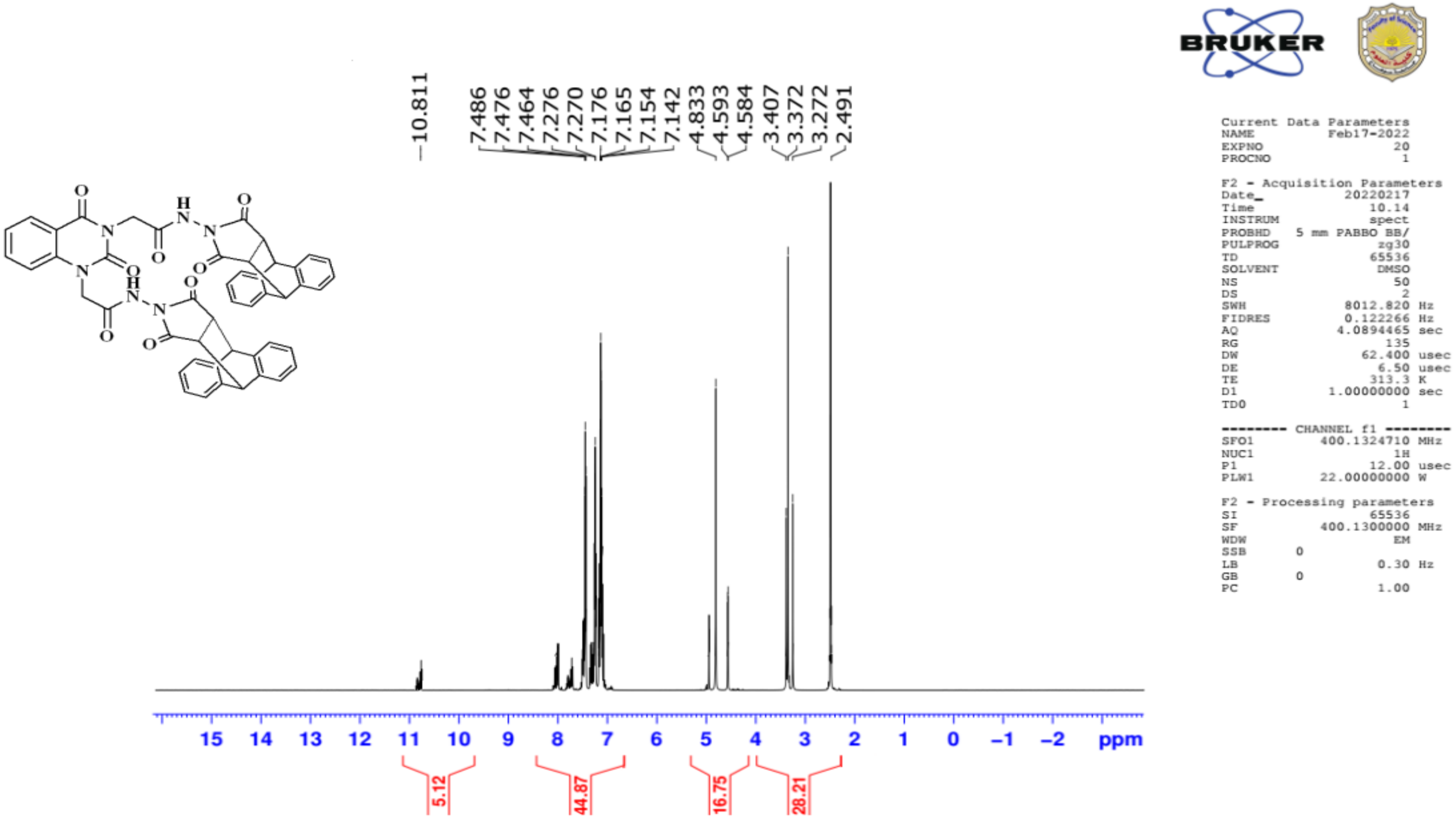

^{1}H-NMR of compound (5c)

-

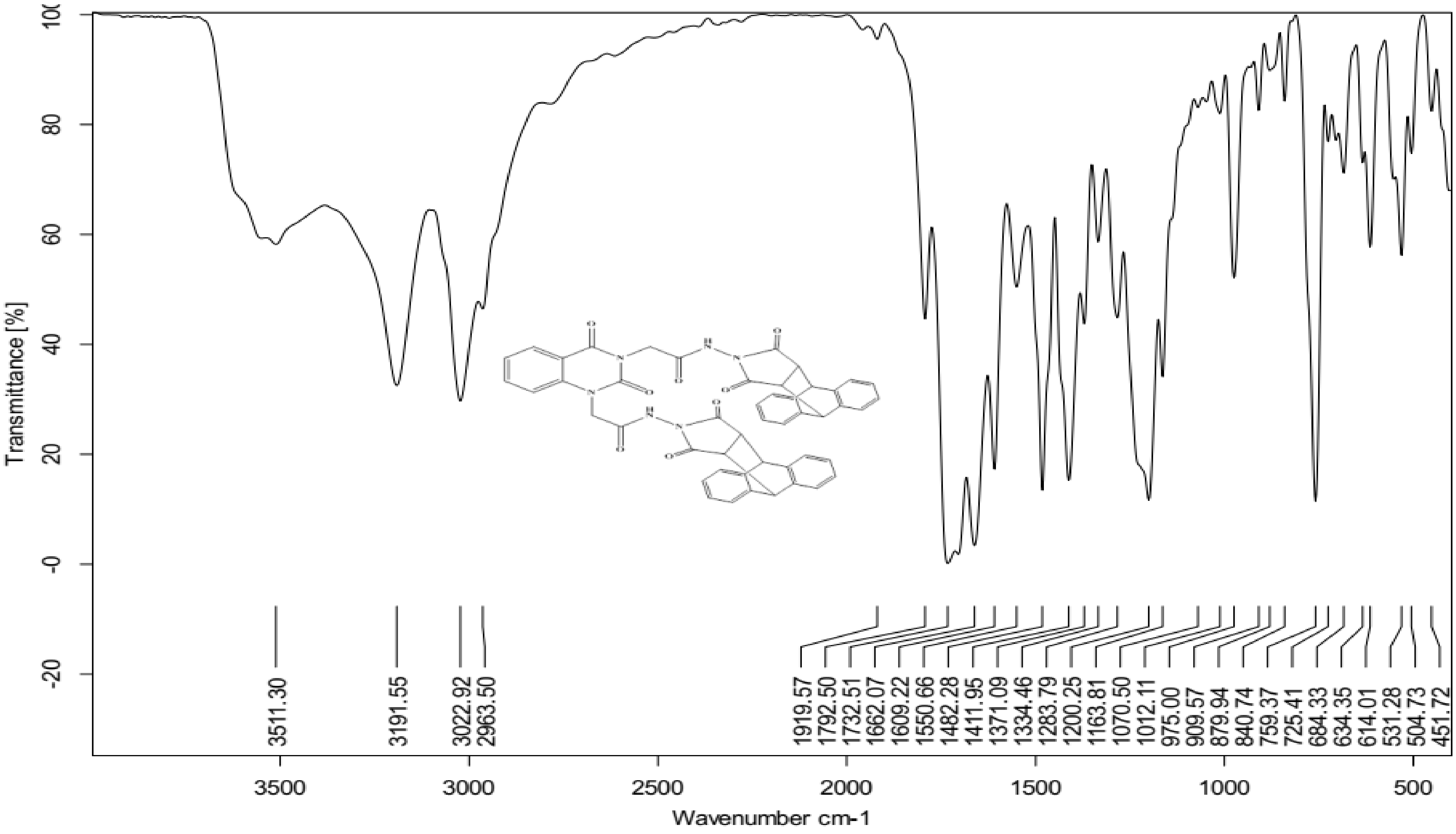

FT-IR of compound (5c)

-

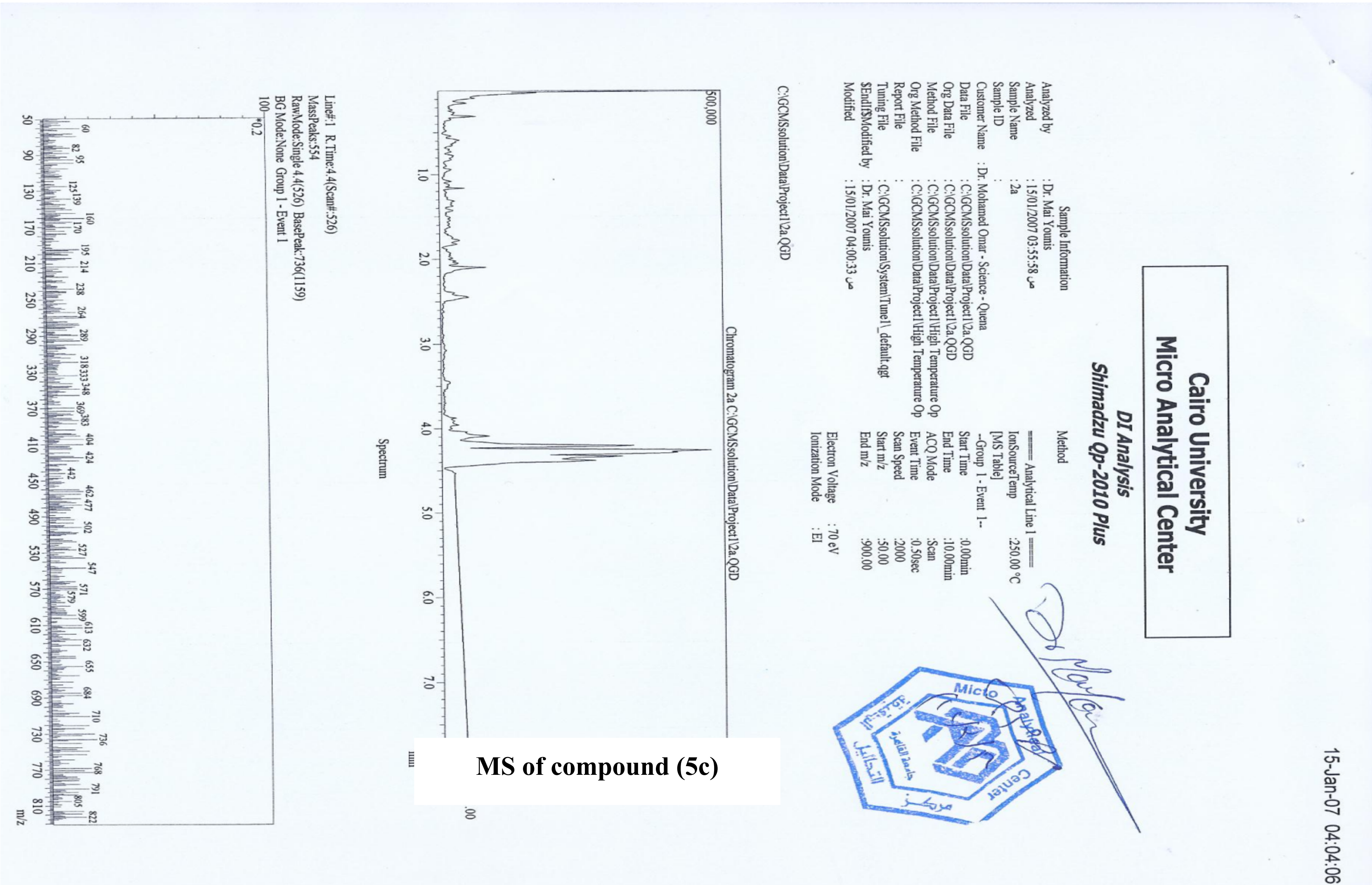

MS of compound (5c)

-

Compound (6a)

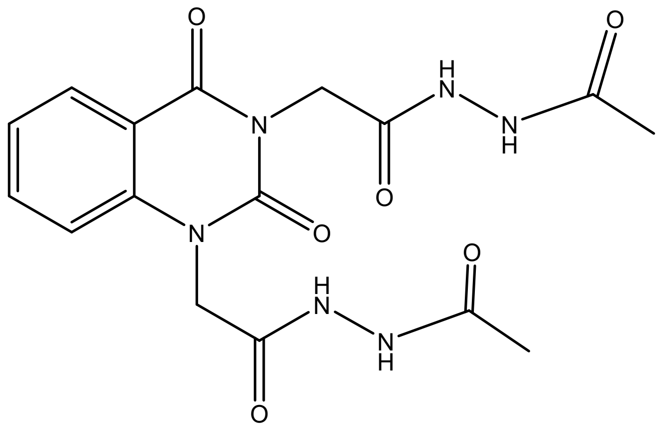

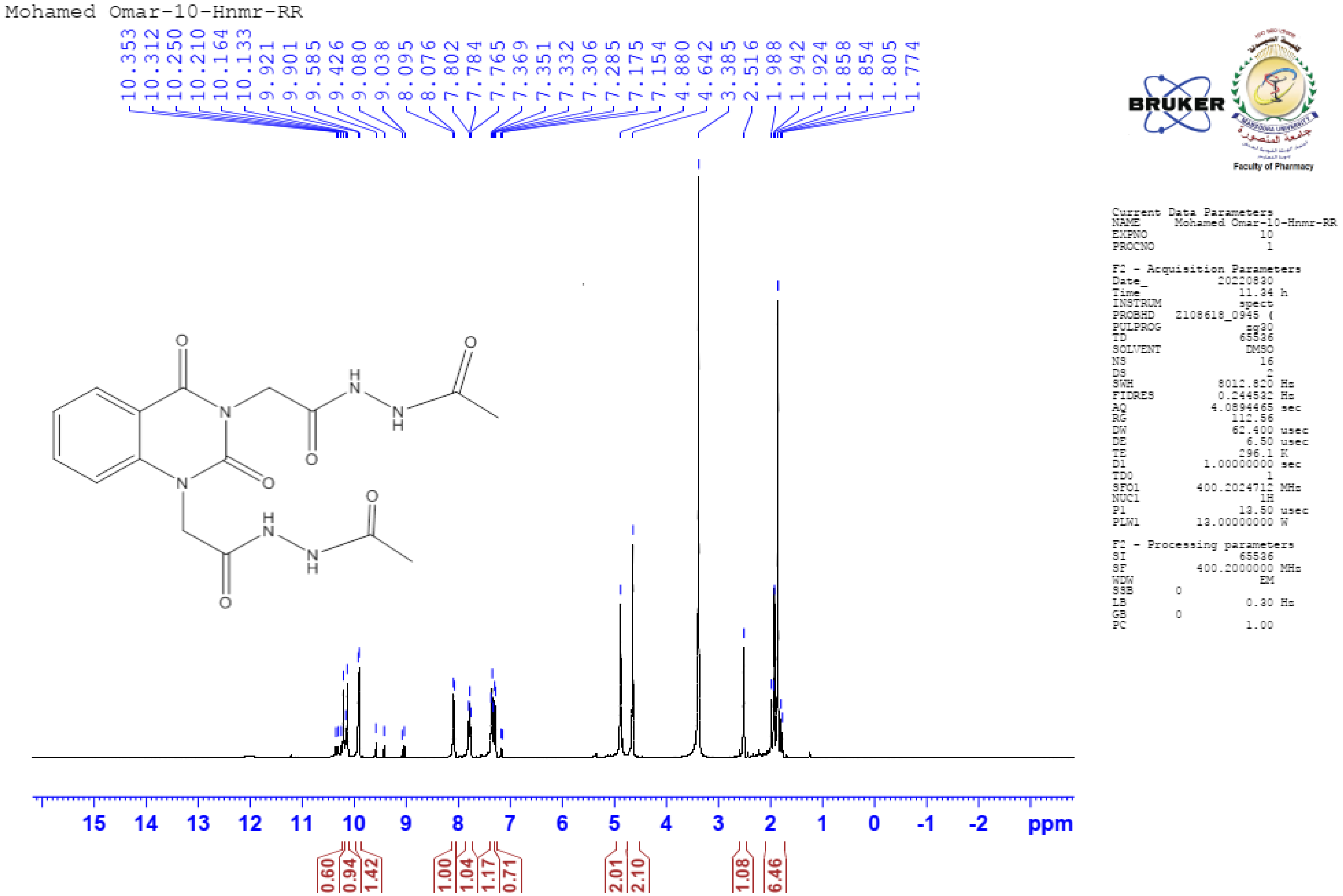

^{1}H-NMR of compound (6a)

-

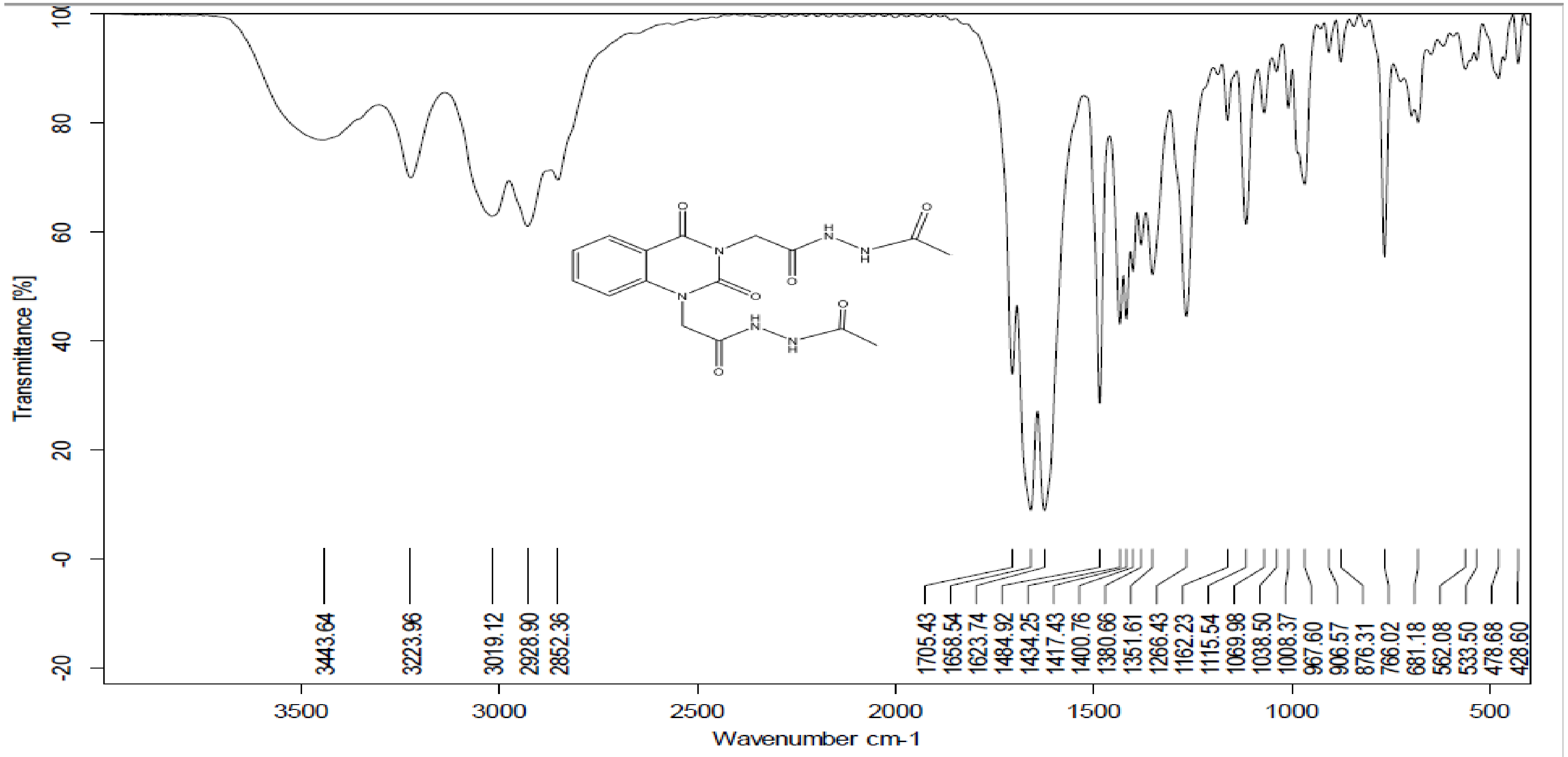

Transmittance [%]
100
80
60
40
20
-0
-20
3443.64
3223.96
3019.12
2928.90
2852.36
1705.43
1658.54
1623.74
1484.92
1434.25
1417.43
1400.76
1380.66
1351.61
1266.43
1162.23
1115.54
1069.98
1038.50
1008.37
967.60
906.57
876.31
766.02
681.18
562.08
533.50
478.68
428.60
3500
3000
2500
2000
1500
1000
500
Wavenumber cm-1

-

[illegible]

FT-IR of compound (6a)

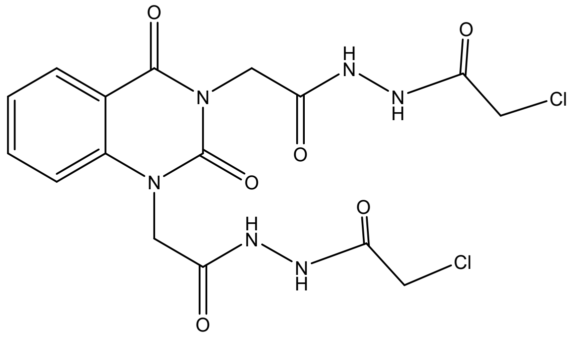

-

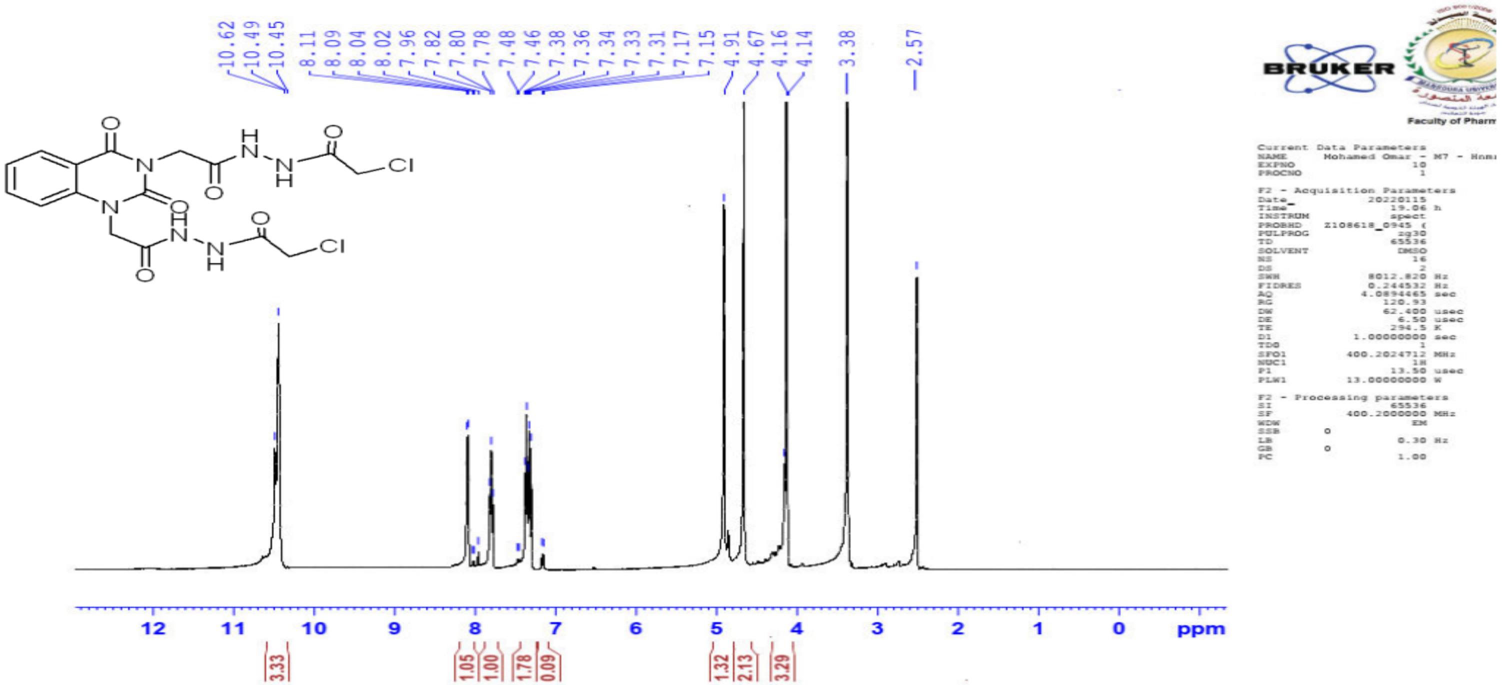

^{1}H-NMR of compound (6b)

-

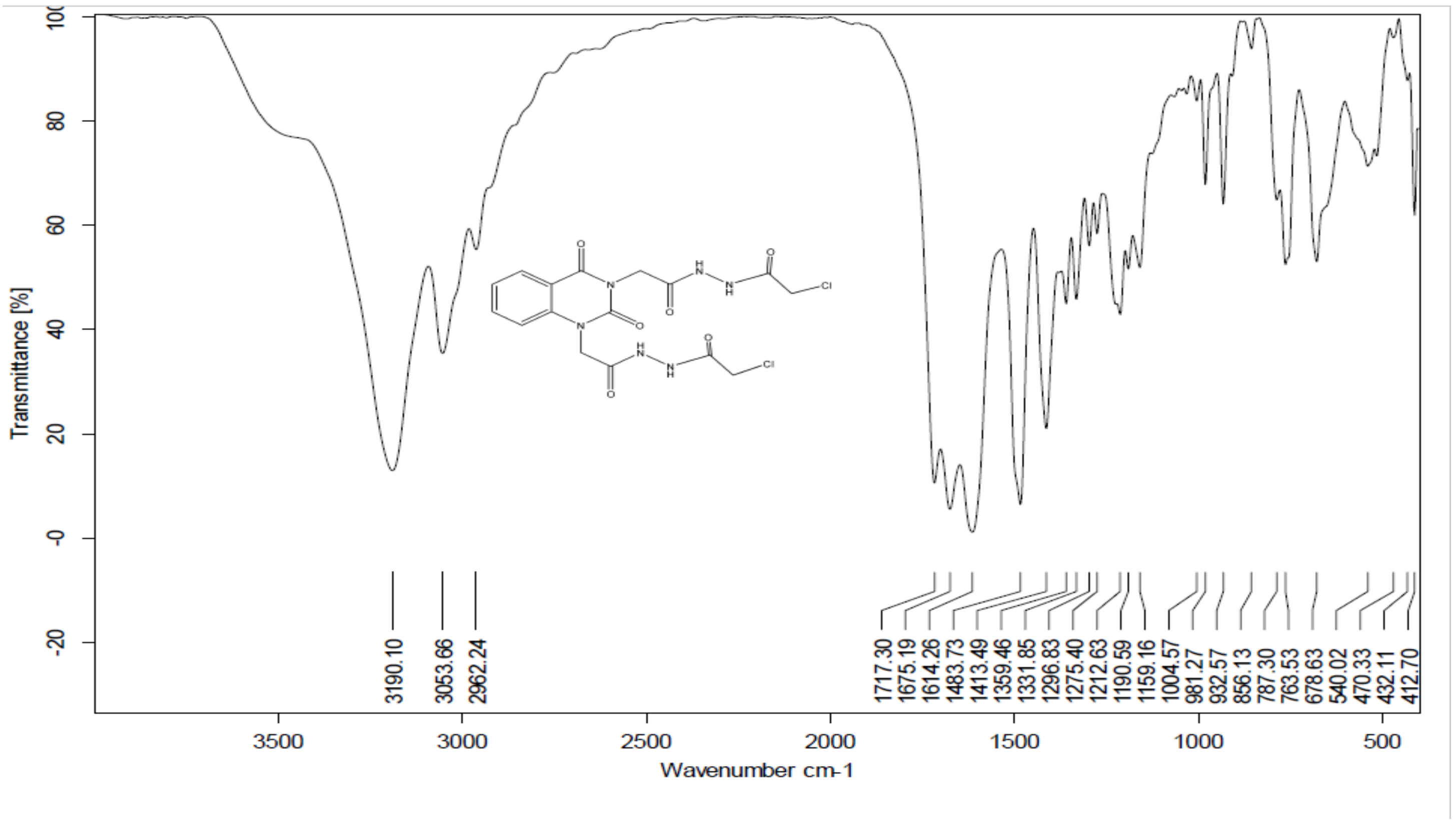

FT-IR of compound (6b)

-

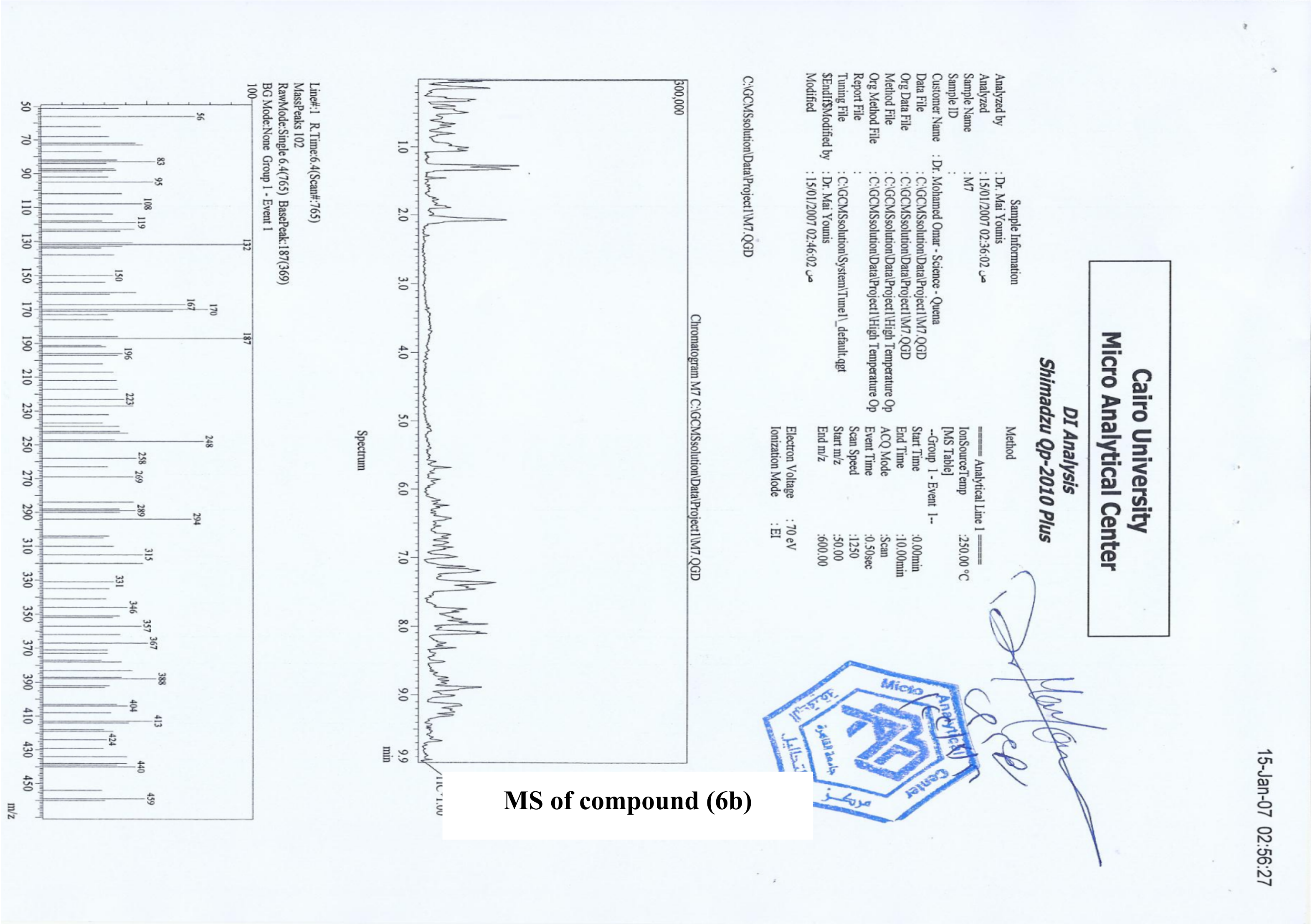

MS of compound (6b)

-

Compound (6c)

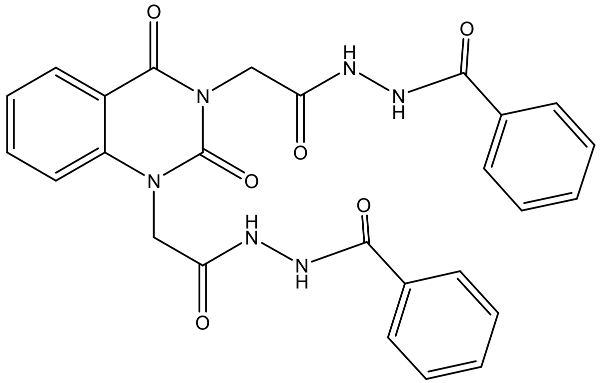

-

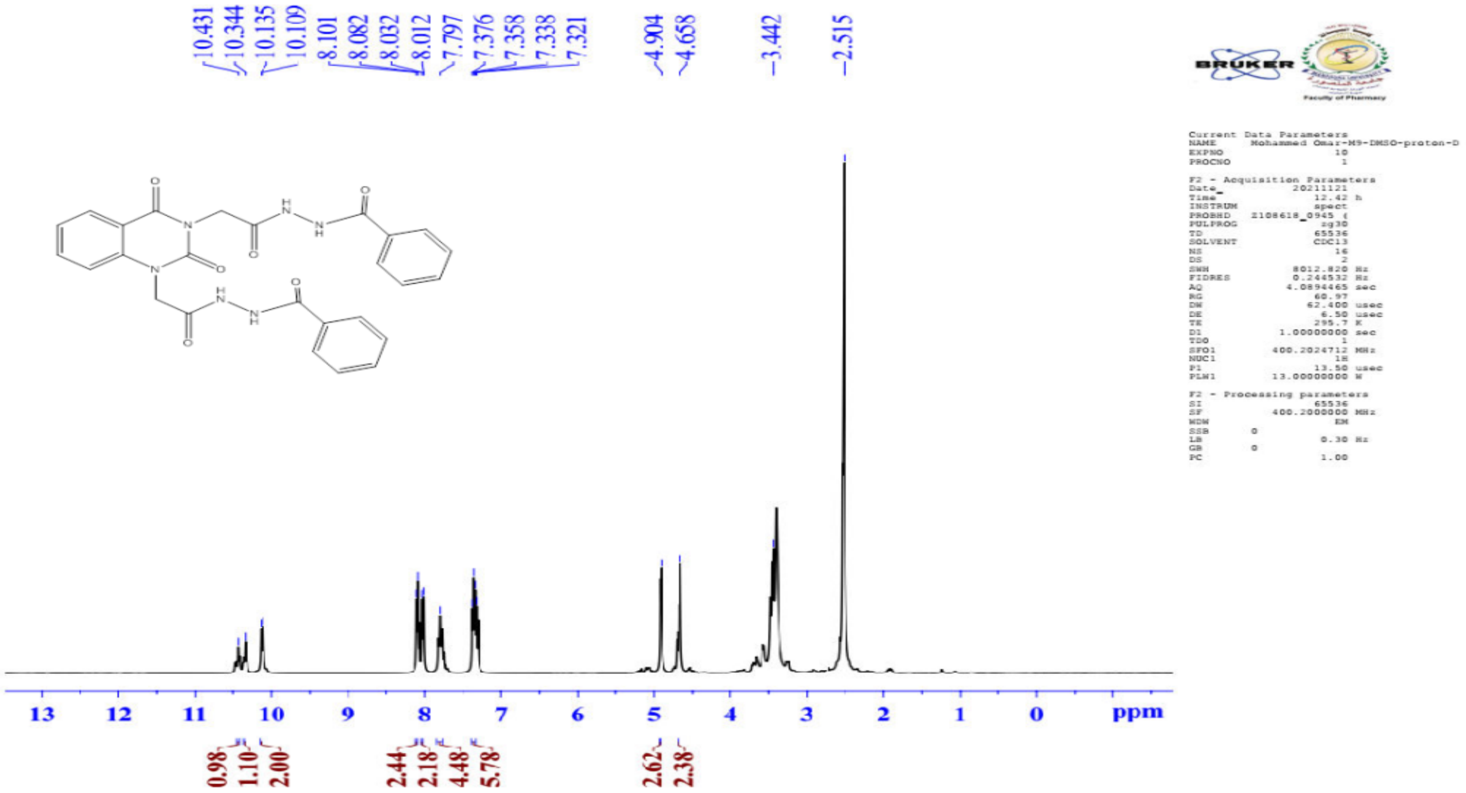

^{1}H-NMR of compound (6c)

-

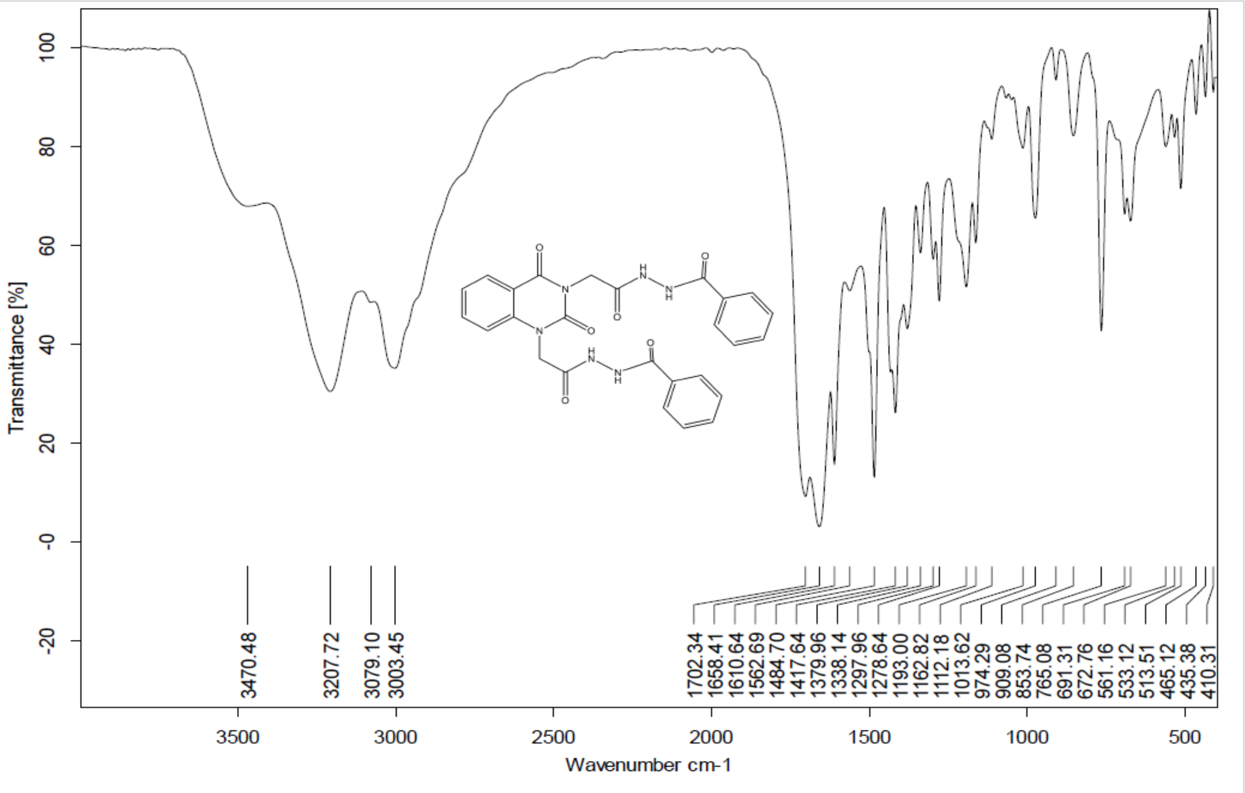

FT-IR of compound (6c)

-

Compound (6d)

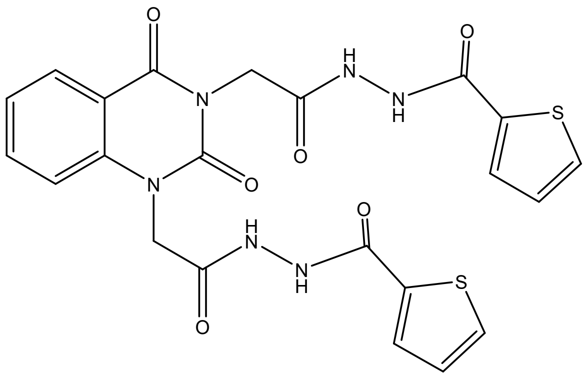

-

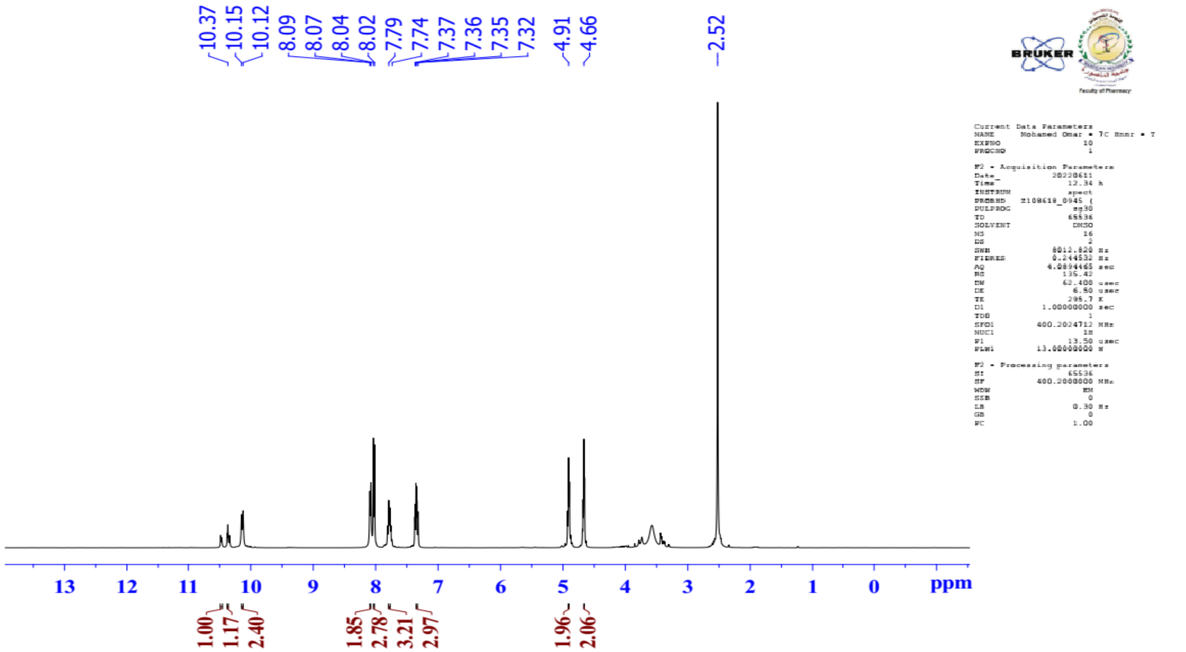

¹H-NMR of compound (6d)

-

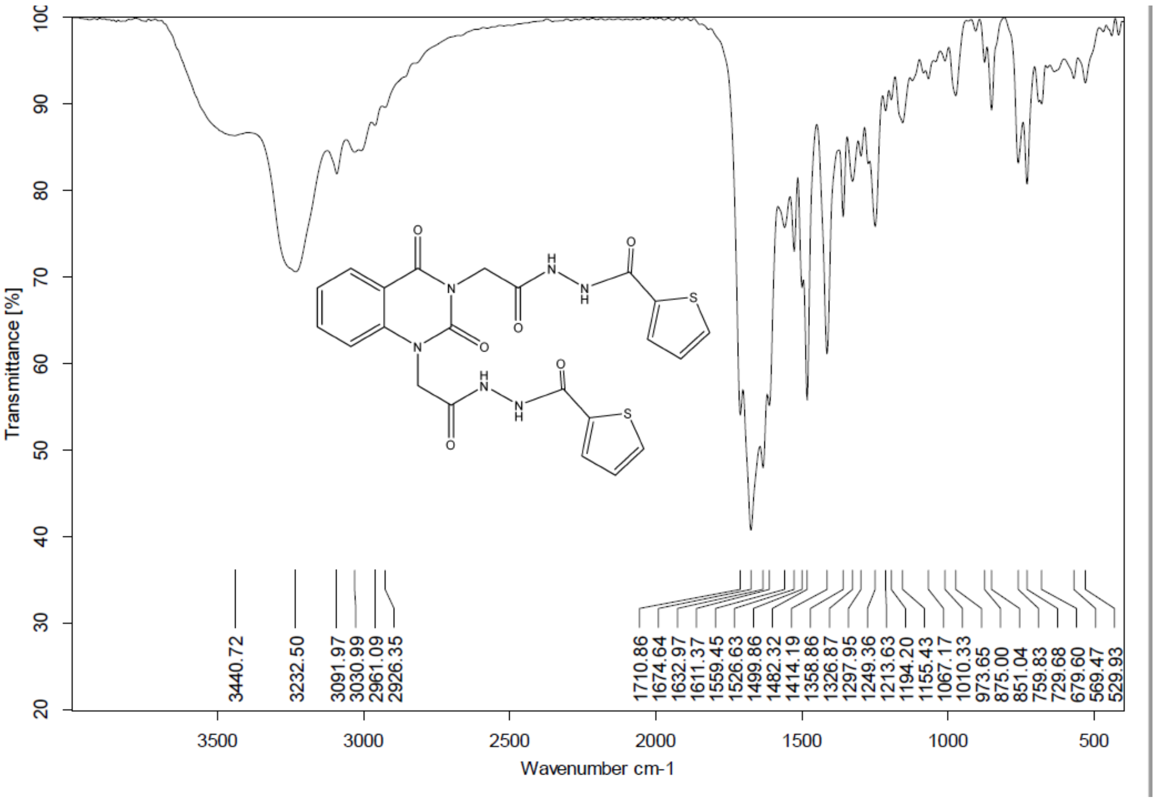

FT-IR of compound (6d)

-

Compound (6e)

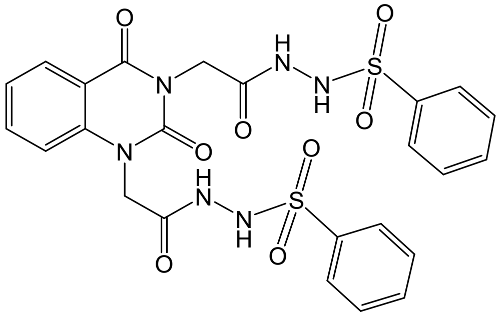

-

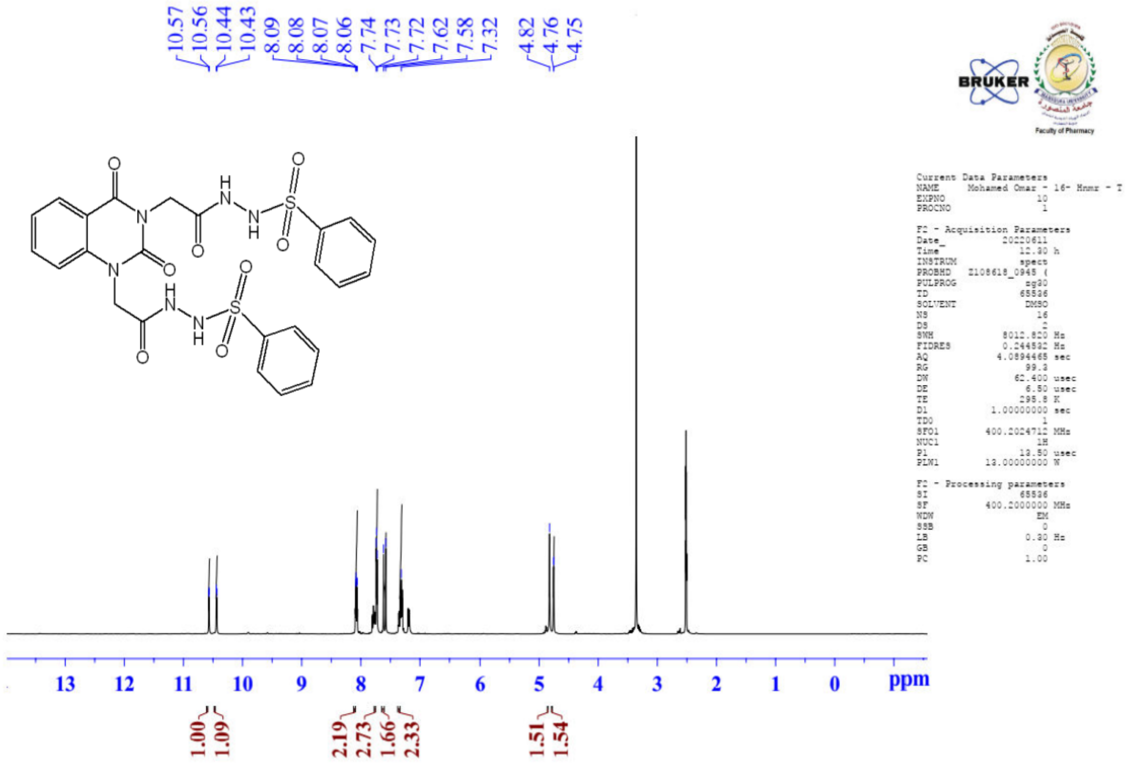

^{1}H-NMR of compound (6e)

-

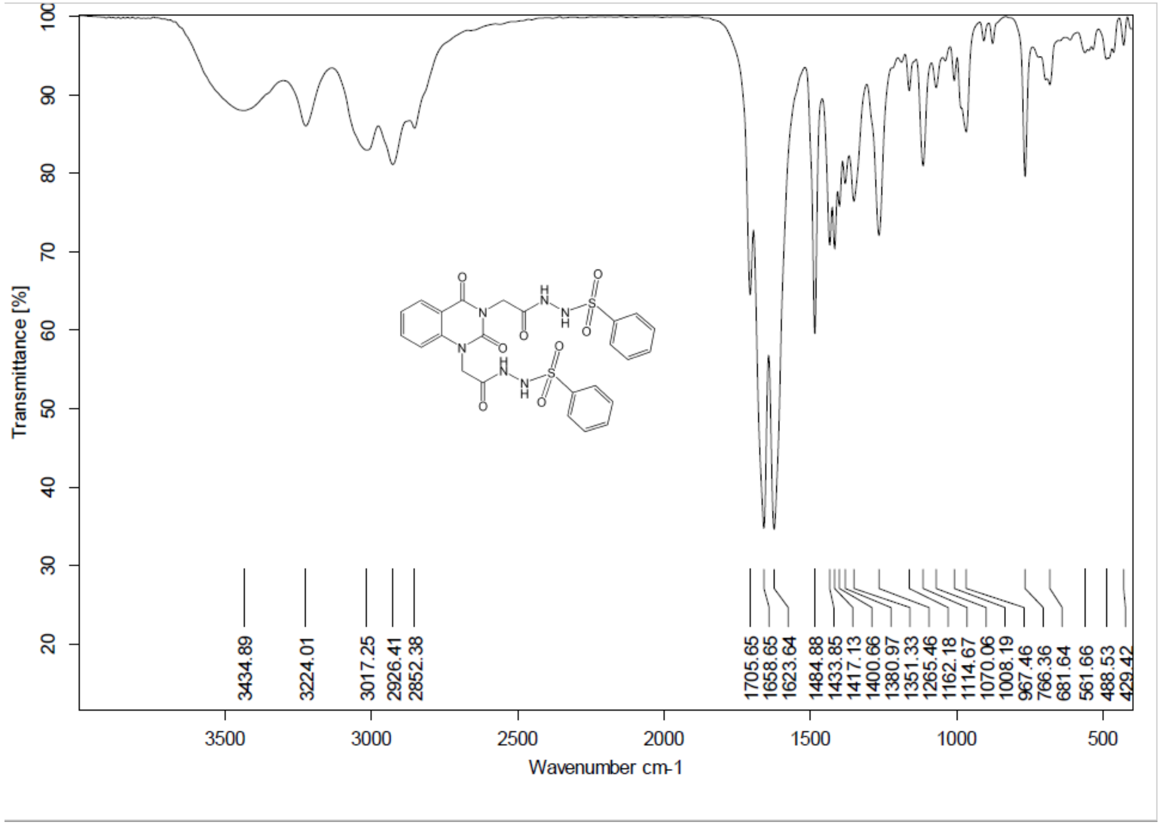

FT-IR of compound (6e)

-

Compound (7a)

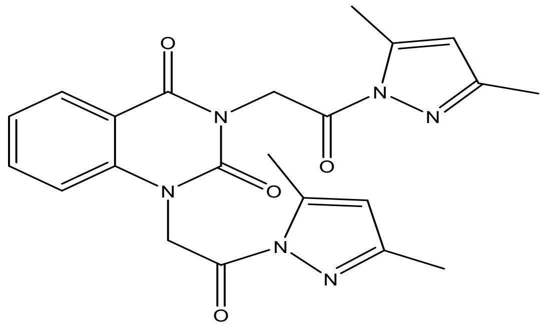

-

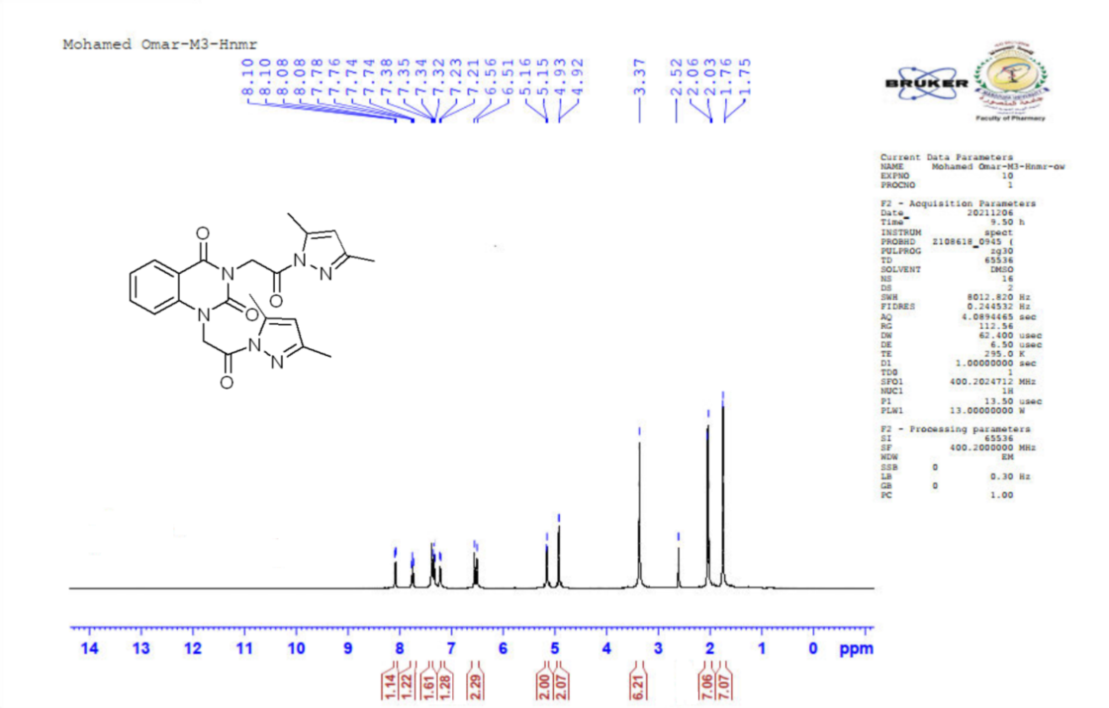

^{1}H-NMR of compound (7a)

-

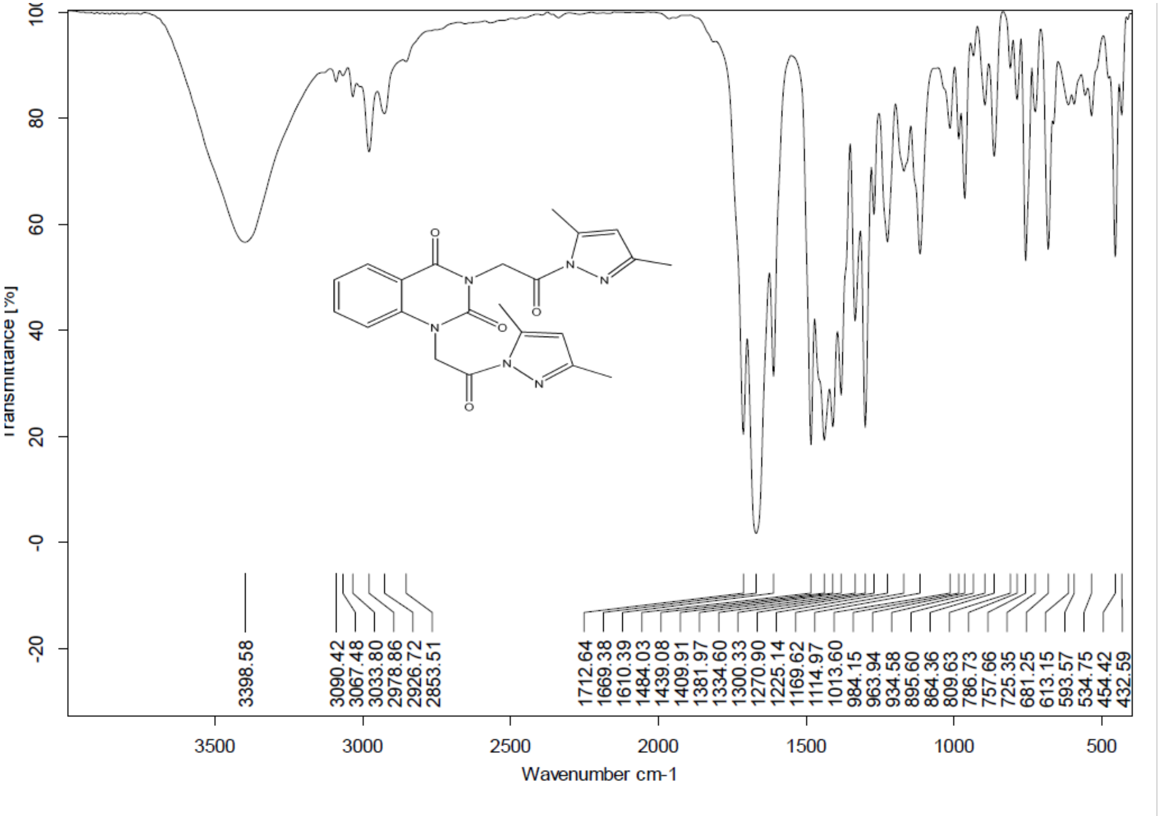

FT-IR of compound (7a)

-

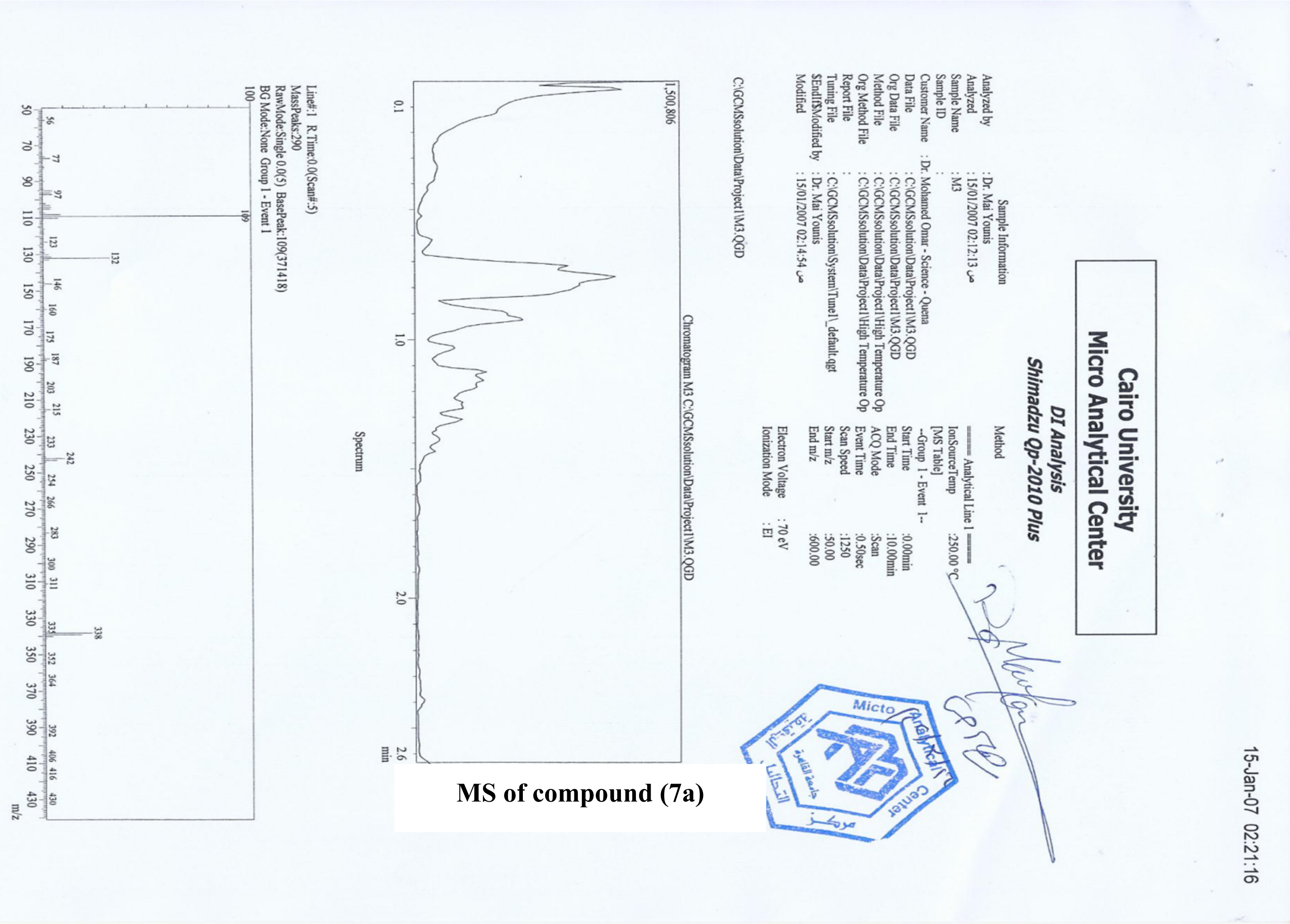

MS of compound (7a)

-

Compound (7b)

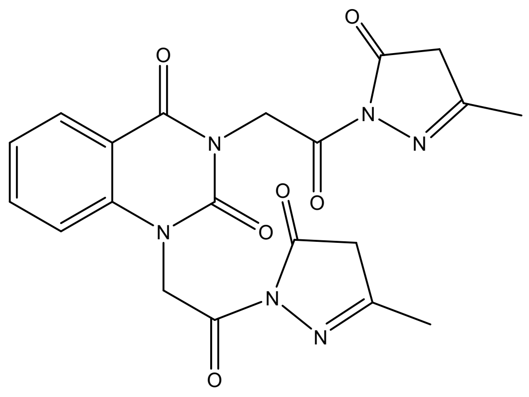

-

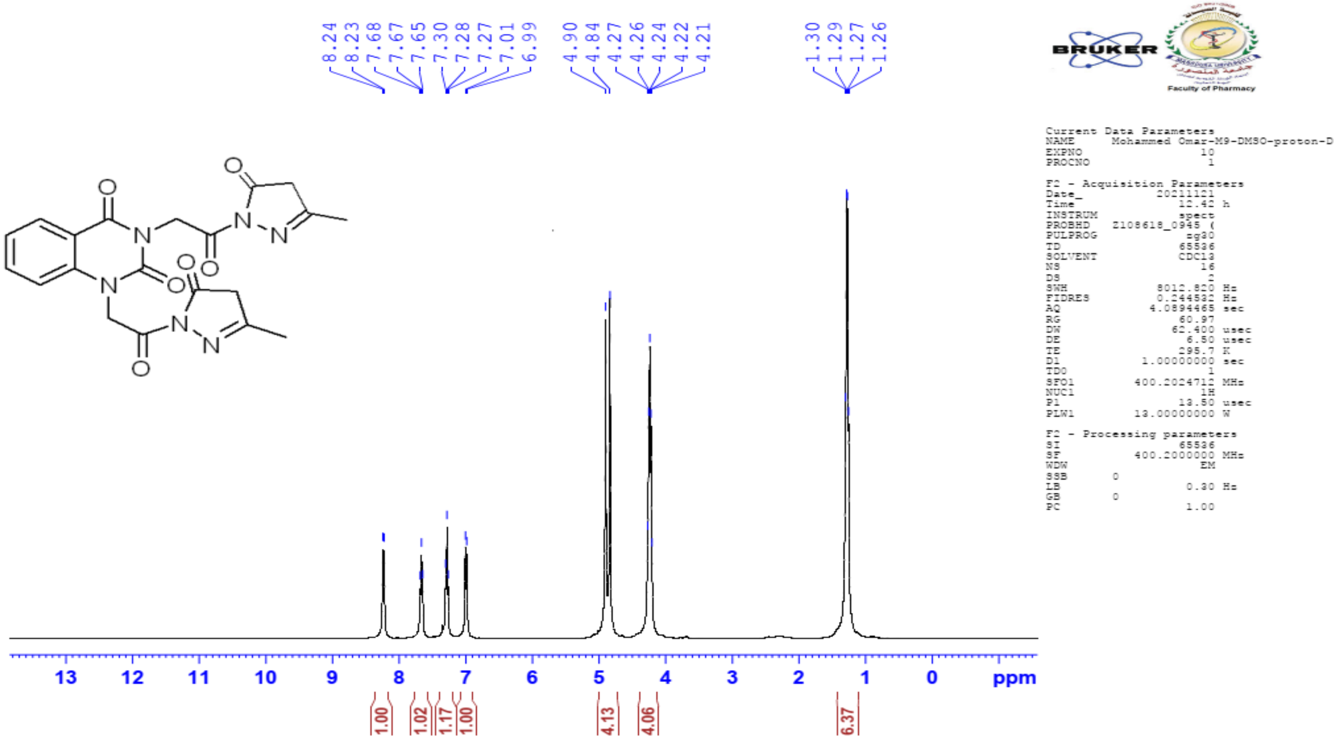

^{1}H-NMR of compound (7b)

-

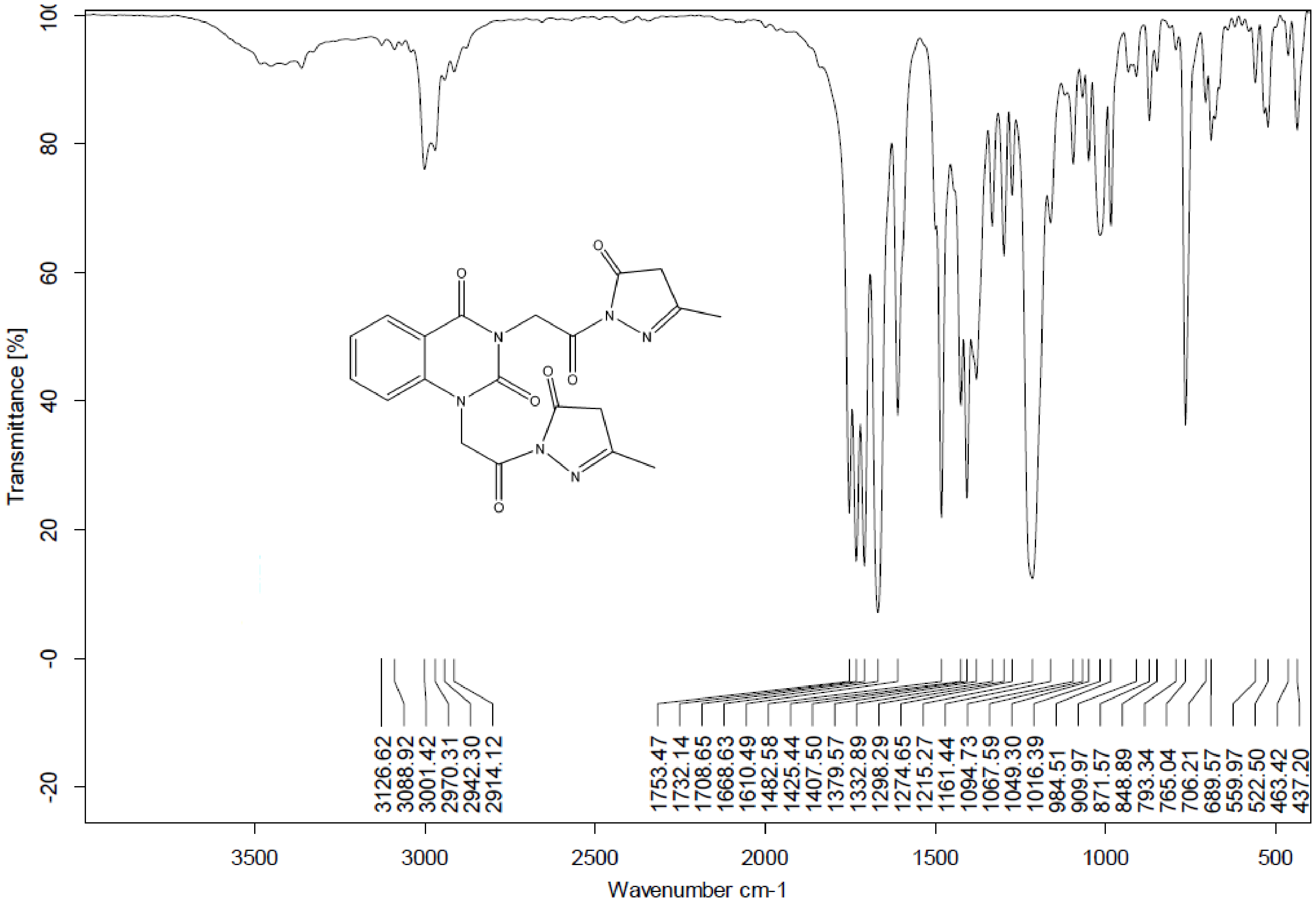

FT-IR of compound (7b)

-

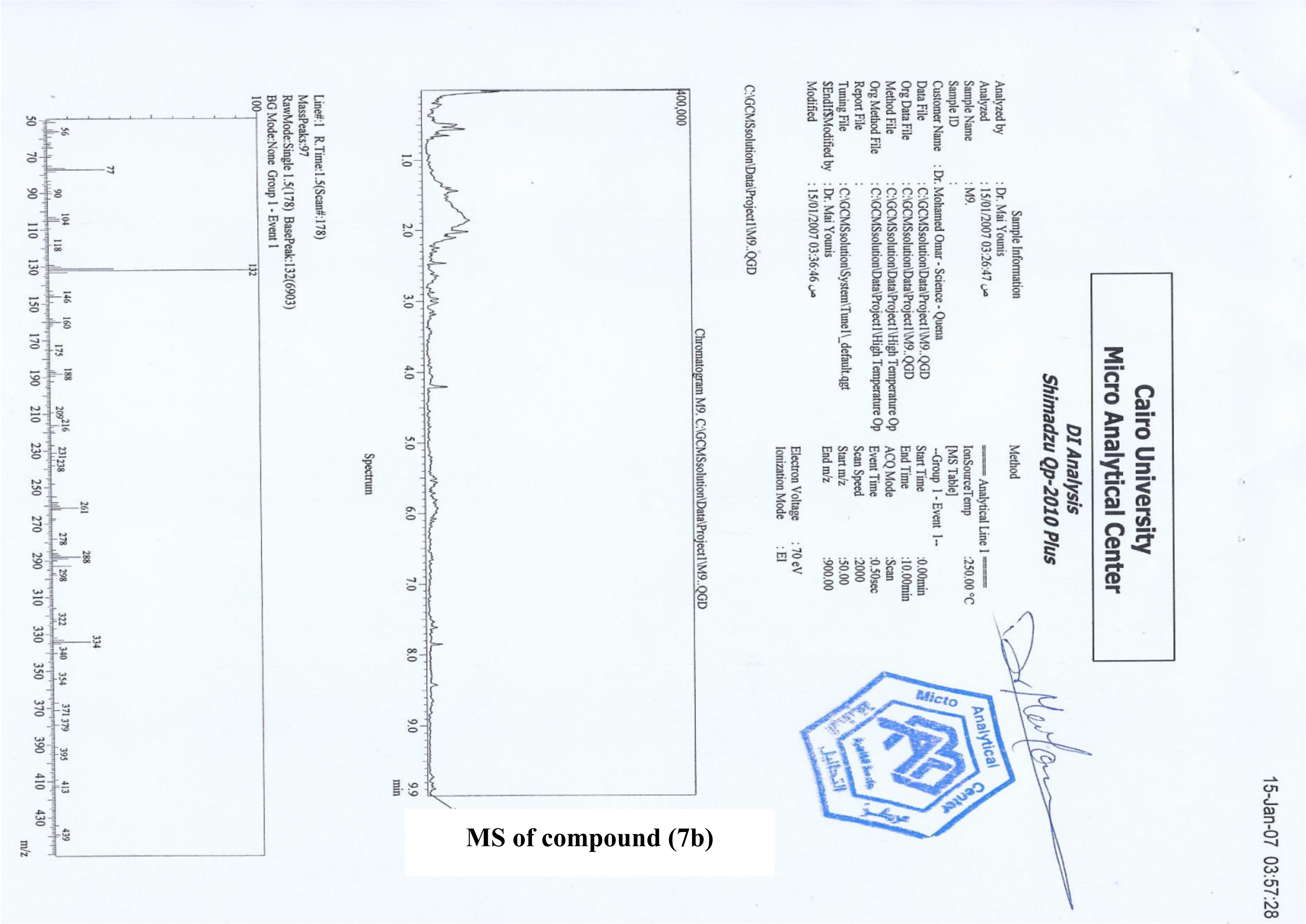

MS of compound (7b)

-

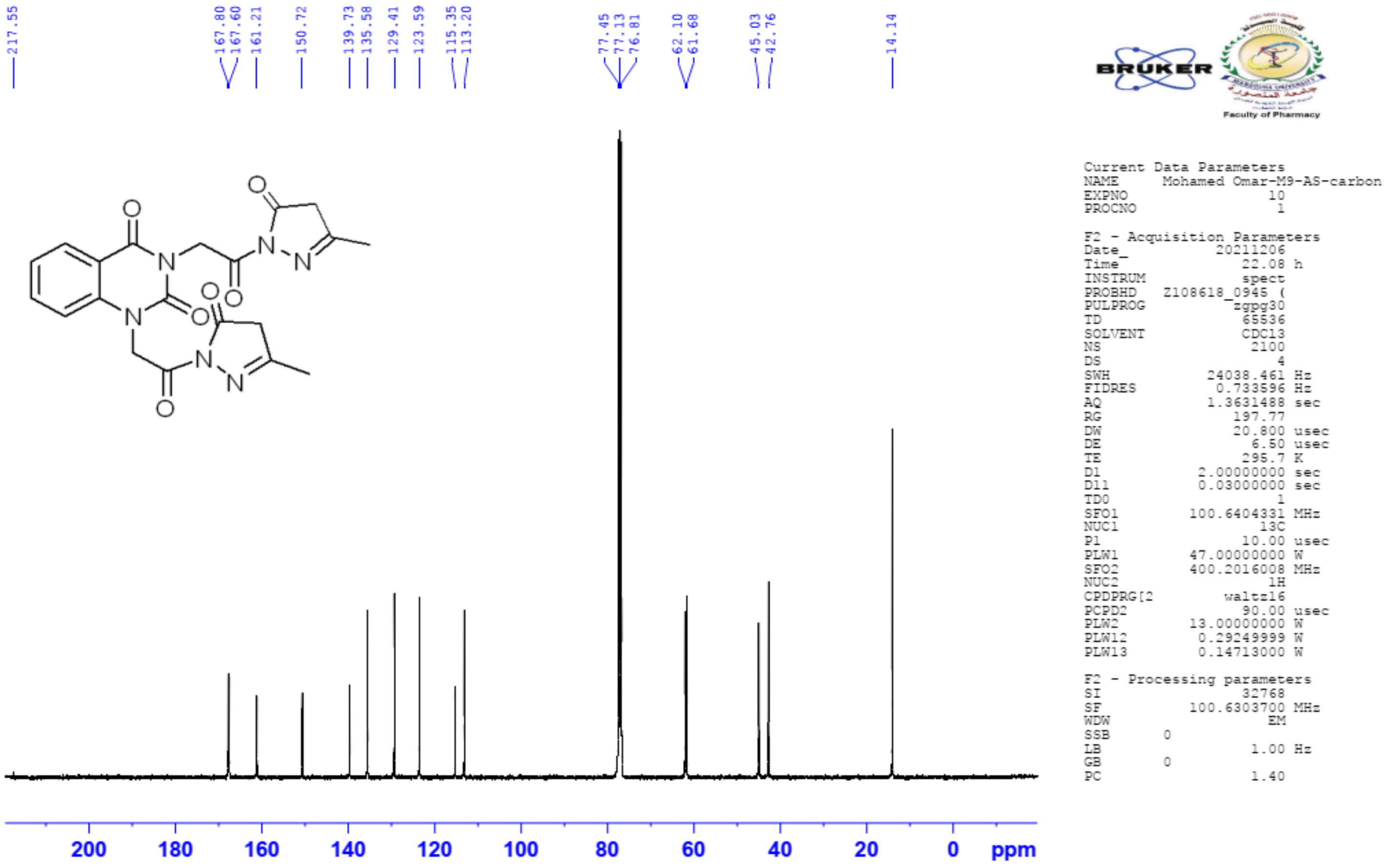

C13-NMR of compound (7b)

-

-

Compound (8)

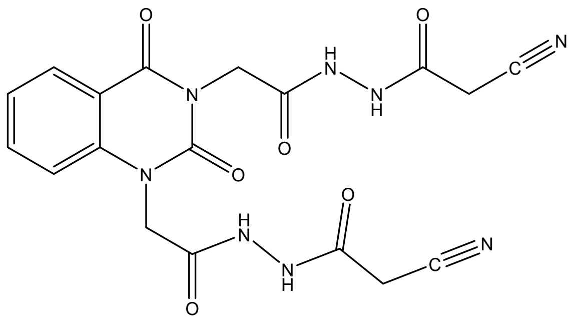

-

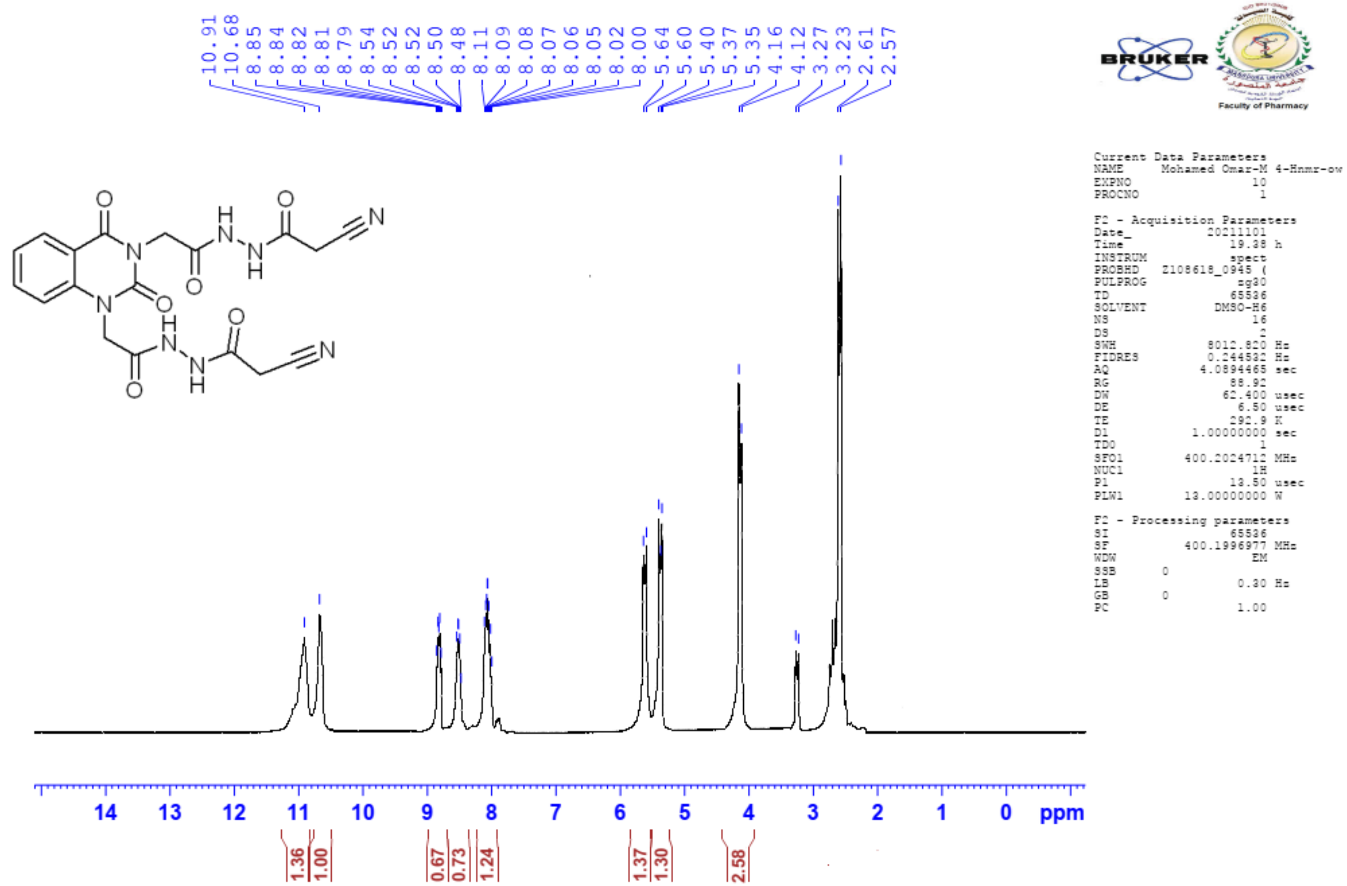

^{1}**H-NMR of compound (8)**

-

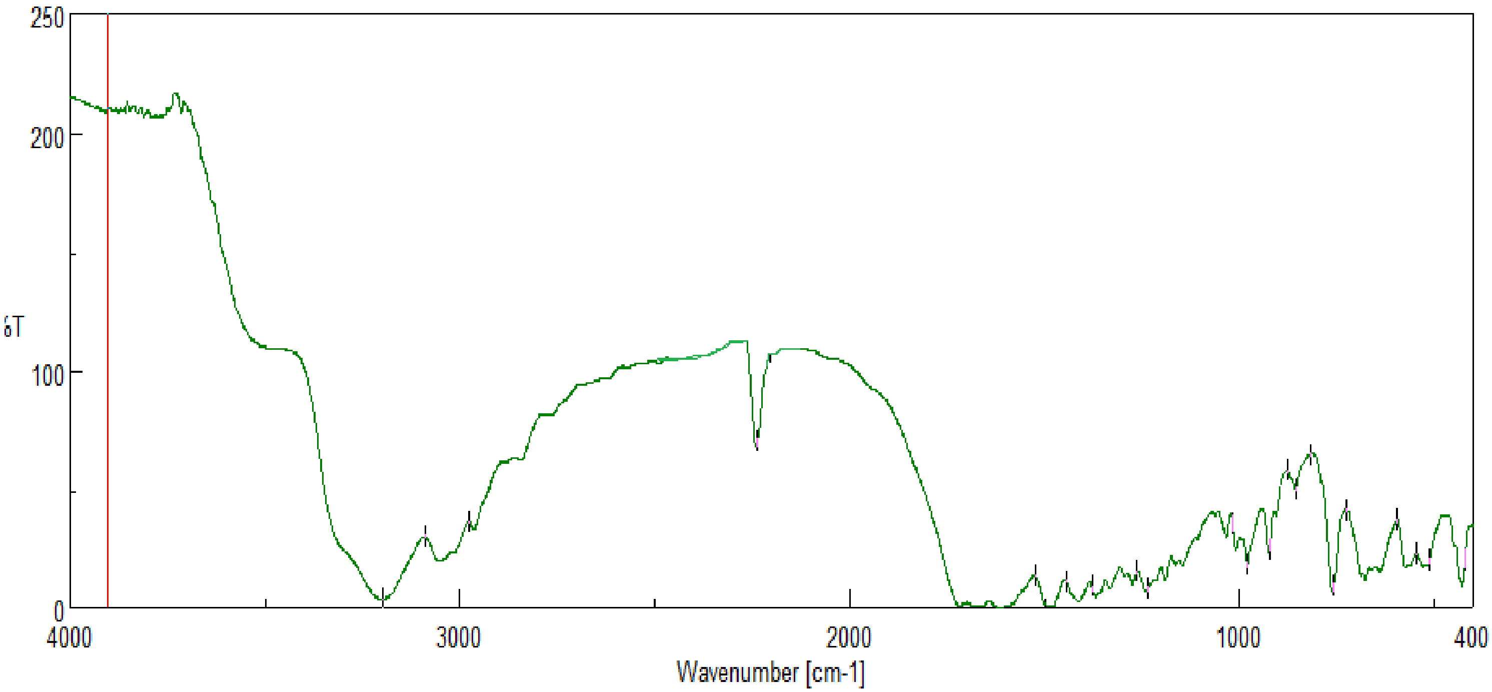

FT-IR of compound (8)

-

Compound (9a)

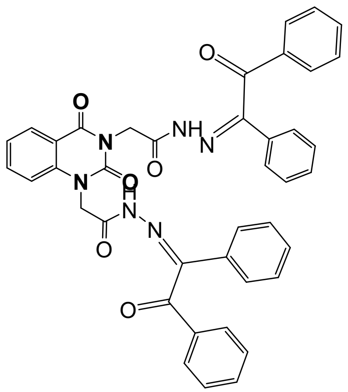

-

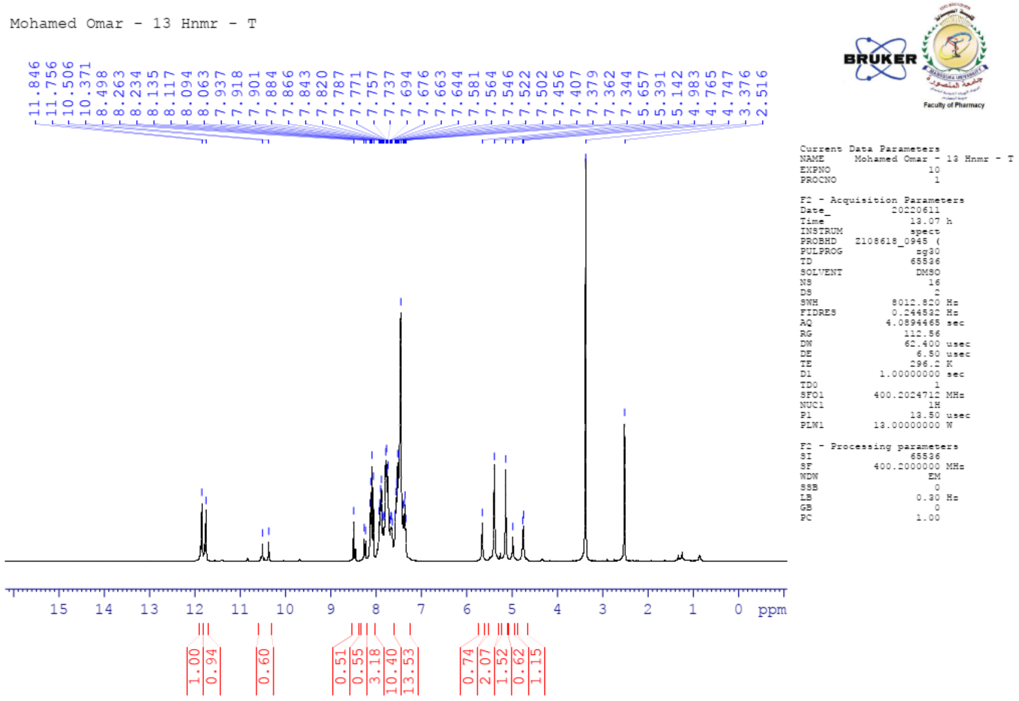

^{1}H-NMR of compound (9a)

-

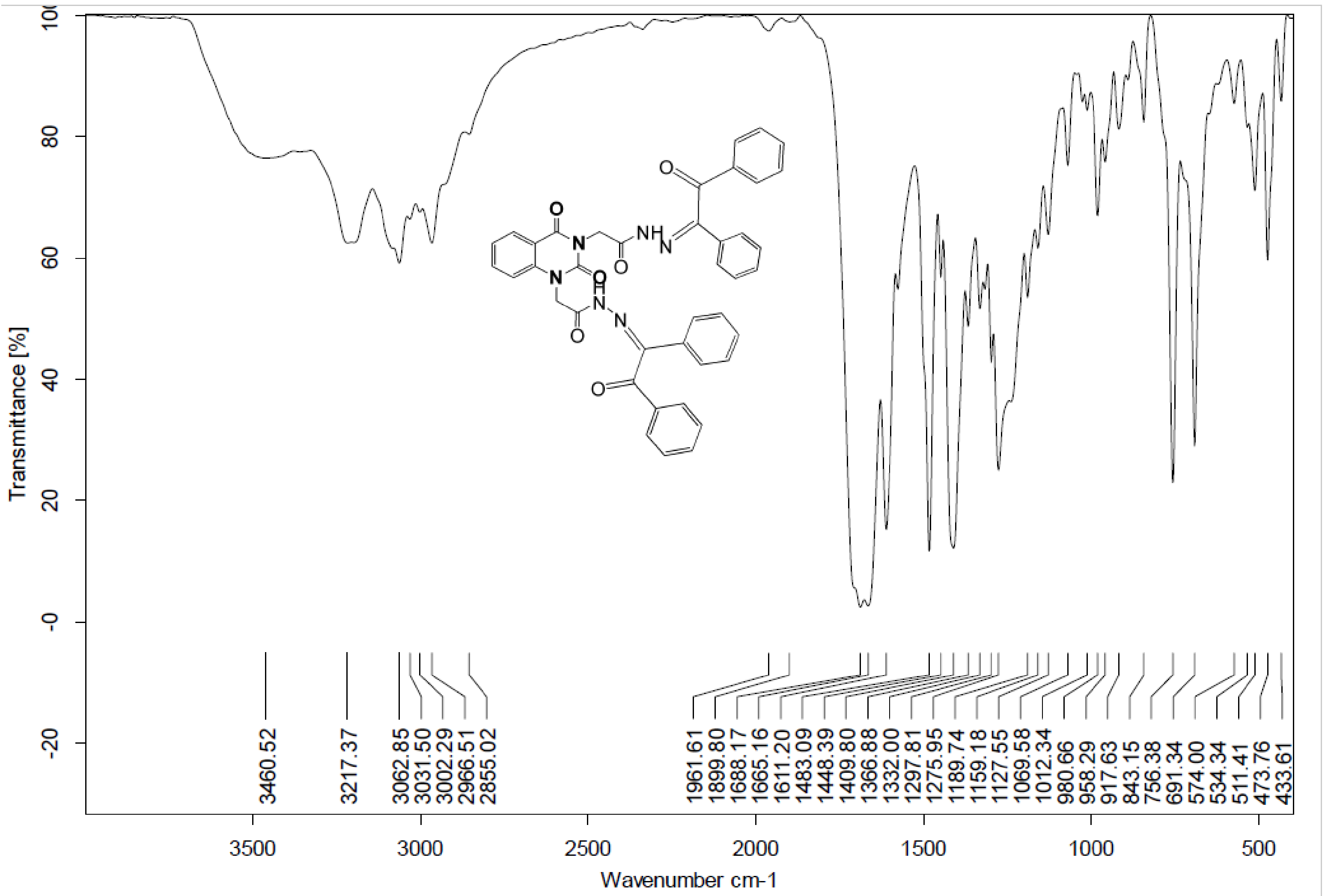

FT-IR of compound (9a)
